# Preventive Home Visits for Mortality, Morbidity, and Institutionalization in Older Adults: A Systematic Review and Meta-Analysis

**DOI:** 10.1371/journal.pone.0089257

**Published:** 2014-03-12

**Authors:** Evan Mayo-Wilson, Sean Grant, Jennifer Burton, Amanda Parsons, Kristen Underhill, Paul Montgomery

**Affiliations:** 1 Centre for Outcomes Research and Effectiveness, Research Department of Clinical, Educational & Health Psychology, University College London, London, United Kingdom; 2 Centre for Evidence-Based Intervention, Department of Social Policy and Intervention, University of Oxford, Oxford, United Kingdom; 3 Yale Law School, New Haven, Connecticut, United States of America; 4 Yale Law School, Yale Center for Interdisciplinary Research on AIDS, New Haven, Connecticut, United States of America; Oregon Health & Science University, United States of America

## Abstract

**Background:**

Home visits for older adults aim to prevent cognitive and functional impairment, thus reducing institutionalization and mortality. Visitors may provide information, investigate untreated problems, encourage medication compliance, and provide referrals to services.

**Methods and Findings:**

*Data Sources*: Ten databases including CENTRAL and Medline searched through December 2012. *Study Selection*: Randomized controlled trials enrolling community-dwelling persons without dementia aged over 65 years. Interventions included visits at home by a health or social care professional that were not related to hospital discharge. *Data Extraction and Synthesis*: Two authors independently extracted data. Outcomes were pooled using random effects. *Main Outcomes and Measures*: Mortality, institutionalization, hospitalization, falls, injuries, physical functioning, cognitive functioning, quality of life, and psychiatric illness.

**Results:**

Sixty-four studies with 28642 participants were included. Home visits were not associated with absolute reductions in mortality at longest follow-up, but some programs may have small relative effects (relative risk = 0.93 [0.87 to 0.99]; absolute risk = 0.00 [−0.01 to 0.00]). There was moderate quality evidence of no overall effect on the number of people institutionalized (RR = 1.02 [0.88 to 1.18]) or hospitalized (RR = 0.96 [0.91 to 1.01]). There was high quality evidence for number of people who fell, which is consistent with no effect or a small effect (odds ratio = 0.86 [0.73 to 1.01]), but there was no evidence that these interventions increased independent living. There was low and very low quality evidence of effects for quality of life (standardised mean difference = −0.06 [−0.11 to −0.01]) and physical functioning (SMD = −0.10 [−0.17 to −0.03]) respectively, but these may not be clinically important.

**Conclusions:**

Home visiting is not consistently associated with differences in mortality or independent living, and investigations of heterogeneity did not identify any programs that are associated with consistent benefits. Due to poor reporting of intervention components and delivery, we cannot exclude the possibility that some programs may be effective.

## Introduction

About 13% of Americans and 15–20% of Europeans are over 65 years old [1], and most wish to remain in their own homes for as long as possible [2], [3]. However, functional decline increases with age and can lead to loss of independence and early death [4].

Preventive home visits by health professionals aim to increase autonomy through primary, secondary, and tertiary prevention activities. These differ from home care interventions to treat or to rehabilitate people with medical problems. Some programs focus on one risk factor, such as falls. Others include multidimensional geriatric assessment (MGA) to evaluate and to improve medical, functional, psychosocial, and environmental problems and resources. Whilst proximal outcomes differ, these interventions all ultimately aim to prevent negative long-term outcomes like institutionalization and mortality.

Several previous reviews have argued that home visits are associated with clinical and economic benefits [5]–[9]; however, authors have questioned their active ingredients [10], suggested that resource-demanding processes be replaced with more efficient services [11], and argued that they should be discontinued altogether [12].

The United States Preventive Services Task Force does not recommend multifactorial risk assessment for all community-dwelling elderly due to uncertainty of evidence, but this position may be revised as more information becomes available [13]. As previous reviews about home visits are now outdated [14], an updated synthesis of relevant studies is required to inform guidelines and ongoing research.

## Methods

### Study selection

We conducted a systematic review and meta-analysis of randomized controlled trials to assess the effectiveness of preventive home visits for community-dwelling older adults (65+ years) without dementia. Through pre-specified subgroup analysis, we also investigated factors that may moderate these effects [15]. Studies that evaluated follow-up home visits directly related to recent hospital discharge (e.g., to assess or to attend a recently treated condition), and studies in which more than 50% of participants had dementia, were excluded.

In December 2012, we searched the following databases from inception and without language restriction: British Nursing Index and Archive, C2-SPECTR, CINAHL, CENTRAL, EMBASE, IBSS, Medline, Nursing Full Text Plus, PsycINFO, and Sociological Abstracts (Text S1). Reference lists from previous reviews and from included studies were examined, and trial authors were contacted for unpublished studies and outcomes.

We analyzed effects on mortality, institutionalization, hospitalization, falls, injuries, physical functioning, cognitive functioning, quality of life, and psychiatric illness. To limit the effects of reporting bias, studies were included based on the characteristics of the participants and interventions rather than the outcomes included in published reports.

### Data analysis

Following methods recommended by the Campbell Collaboration and the Cochrane Collaborations [16], two authors independently reviewed all citations and extracted data from included studies, such as: context, recruitment strategy, study inclusion criteria, demographics, content and delivery of the intervention, and outcomes. We assessed each study using the Cochrane Risk of Bias Tool; risk of bias was judged ‘high’ for blinding of personnel and blinding of participants *per se*.

Authors of included studies were contacted to supply any unreported information and to provide information to permit intention-to-treat analyses. Where possible, dichotomous data were entered directly into Review Manager (RevMan) Version 5.2 [17], and relative risks or rate ratios and 95% confidence intervals (CIs) were calculated using Mantel-Haenszel methods. For dichotomous outcomes that were fully reported in all studies, we also calculated the absolute risk difference. Standardized mean differences (SMDs) and 95% CIs were calculated for continuous measures using Hedges *g* and combined using inverse variance methods. When studies reported more than one measure of a particular outcome (e.g., psychiatric illness measured using two scales), we averaged the results in Comprehensive Meta-Analysis (CMA) Version 2 [18] before entering data in RevMan 5.2. To estimate event rates in studies reporting the number of events but not reporting time at risk, we assumed that (i) all completers were included for the full duration of the study and (ii) dropouts were at risk for 50% of the year in which they died or left the study. Random-effects models were used due to variability in populations and intervention characteristics across studies. In all forest plots, displays extend beyond the range of probable effects (75% reduction to 400% increase; 4 standard deviations difference in means), and studies are ordered by weight.

For critical outcomes included in the Summary of Findings Table (Table S1), we conducted trim-and-fill analyses [19] to investigate the possibility of reporting bias. Overall confidence in the results was assessed using the Grading of Recommendations Assessment, Development and Evaluation Working Group (GRADE) criteria [20], [21].

### Investigation of heterogeneity

Differences among included studies were assessed in terms of their participants, interventions, outcomes, and methods. For each meta-analysis, we also visually inspected forest plots to see if the confidence intervals of individual studies had poor overlap, conducted a Chi^2^ test, and calculated the I^2^ statistic. We considered meta-analyses to have important heterogeneity when the *p* value for Chi^2^ was less than 0.10 and I^2^ was greater than 25%.

The following subgroups were analyzed when 10 or more studies were available:

1. Number of visits (1; 2 to 4; 5 or more);
2. Visitor's professional group
3. Participant age (≤70, 71–75, 76–80, 81–85, >85);
4. Intervention components:
  - Falls only (interventions that exclusively targeted falls prevention, e.g., exercise to improve balance and strength);
  - MGA (a systematic evaluation of at least 3 of these domains—medical, functional, psychosocial, or environmental);
  - Both falls prevention and MGA;
  - Neither falls prevention nor MGA.

Meta-regressions were conducted in STATA [22] for key outcomes (mortality, institutionalization, falls, and functioning) and four moderators (number of visits, participant age, risk of mortality in the control group, and percentage female) using restricted maximum likelihood.

## Results

### Results of the search

We identified 18784 records, and full texts were obtained for 176 records (Figure S1). Thirty papers were secondary reports of a study reported in another paper; thus, 146 studies were assessed for eligibility. Post-hoc, we included two studies (both identified in the search) in which participants were assigned using quasi-random methods that approximated the characteristics of randomization as described below [23], [24]. Sixty-four studies reported in 86 citations were included in the narrative synthesis (Table S2) [23]–[108], but three of these did not report any outcomes that could be included in a meta-analysis [36], [68], [74].

Seventy-six studies (84 citations) [109]–[192] were excluded for reasons that are enumerated (Table S3). We also identified four ongoing studies [193]–[196] and two studies that could not be obtained [197], [198].

### Description of studies

Overall, the 64 included studies assigned 28642 participants, ranging from 59 [44], [73] to 3743 [23], with a median sample size of 299 per study. Follow-up periods ranged from 3 months [44] to 60 months [23].

Studies took place between 1981 [74] and 2012 [56] in 13 countries, but most were conducted in the USA (14), UK (14), or Canada (11). Studies used varying eligibility criteria; some included people at high risk of institutionalization while others recruited from the general population. Between 0% and 33% of control subjects died before the last follow-up. Participants were recruited through primary care providers (24), general population registries (11), community and social service organizations (7), emergency rooms (6), health insurance plan registers (5), advertisements (4), veterans' health organizations (1), and various combinations of the above (3); 3 studies did not report how participants were recruited. Studies included participants aged over 65 years (1), 70 years (10), 75 years (28), 80 years (18), and 85 years (3). In others (4), the mean age was over 70 years, but some participants could have been under 65 years. One of these studies included people aged 17 to 99 years; the mean age was 69 years, and 75% of participants were over 65 years [26].

There was heterogeneity across studies in the number, duration, and focus of visits. Participants received an average of 4.9 (SD = 4.55) visits per study. The number of visits varied by participant in 8 studies. Eleven studies provided one visit per participant, and one study averaged 30 visits per participant. Visitors were nurses alone (27); other professionals, including health visitors, physiotherapists, social workers, and occupational therapists (20); or a combination of health professionals, usually a nurse in combination with another professional (17). Visits had different but overlapping goals, including falls prevention (17), multi-dimensional geriatric assessment (25), both of the above (16), or another prevention (6). Exercise was included in 21 studies. Many studies did not systematically report specific aspects of program design, components that staff actually delivered, or participant take-up.

Comparisons included usual care (50), attention-matched controls including social visits (10), and wait-lists (3); 1 study did not describe the comparison condition. We would have considered comparisons separately, but we could not determine reliably what the comparison groups actually received across different locations, times, and service settings.

### Quality of the evidence

Most studies adequately described randomization and were at low risk of bias (41), but sequence generation was unclear in 20 (Figure S2). Allocation concealment was also adequate in 33 studies at low risk of bias, but unclear in 27. Two quasi-random studies were included post-hoc and were rated high risk for randomization and allocation concealment *per se* [23], [24], but we concluded that the methods of assignment had the desirable characteristics of randomization. One study replaced a few intervention participants and was rated high risk for sequence generation and allocation concealment [92].

Many studies did not describe what happened to participants living in the same household (e.g., husband and wife) and may have randomized small clusters. No study reported that effects were adjusted for clustering; however, studies that explicitly assigned households had cluster sizes close to one.

Subjective outcomes were at high risk of bias for provider and participant blinding; however, mortality, institutionalization, and hospitalization are not likely to have been affected by biased reporting or assessment. Outcome assessors were not blind in 12 studies, which were at high risk of bias for some outcomes, and it was unclear if assessors were blind in 6 studies; other studies were at low risk of bias.

Missing data were unlikely to affect estimates of effects for dichotomous outcomes, including mortality, institutionalization, and hospitalization. As a result, 31 studies were at low risk of bias for incomplete outcome data, 16 studies were unclear, and 17 were at high risk of bias, including two that excluded participants from analyses if they refused visits or did not comply with the protocol [60], [79].

Risk of selective outcome reporting was unclear in 43 studies that did not reference a protocol, and there was high risk of bias in 18 studies that omitted measured outcomes. Only 3 studies were clearly free of selective outcome reporting (i.e., outcomes were registered and reported in full).

In trim-and-fill analyses (Figure S3), there was some evidence of small study bias—studies were trimmed for mortality (3), institutionalization (1), falls (2), and hospitalization (5)—but there was little evidence of benefits, and the adjusted effects were not importantly different from the observed effects.

### Quantitative synthesis

#### Mortality

**Figure 1:**
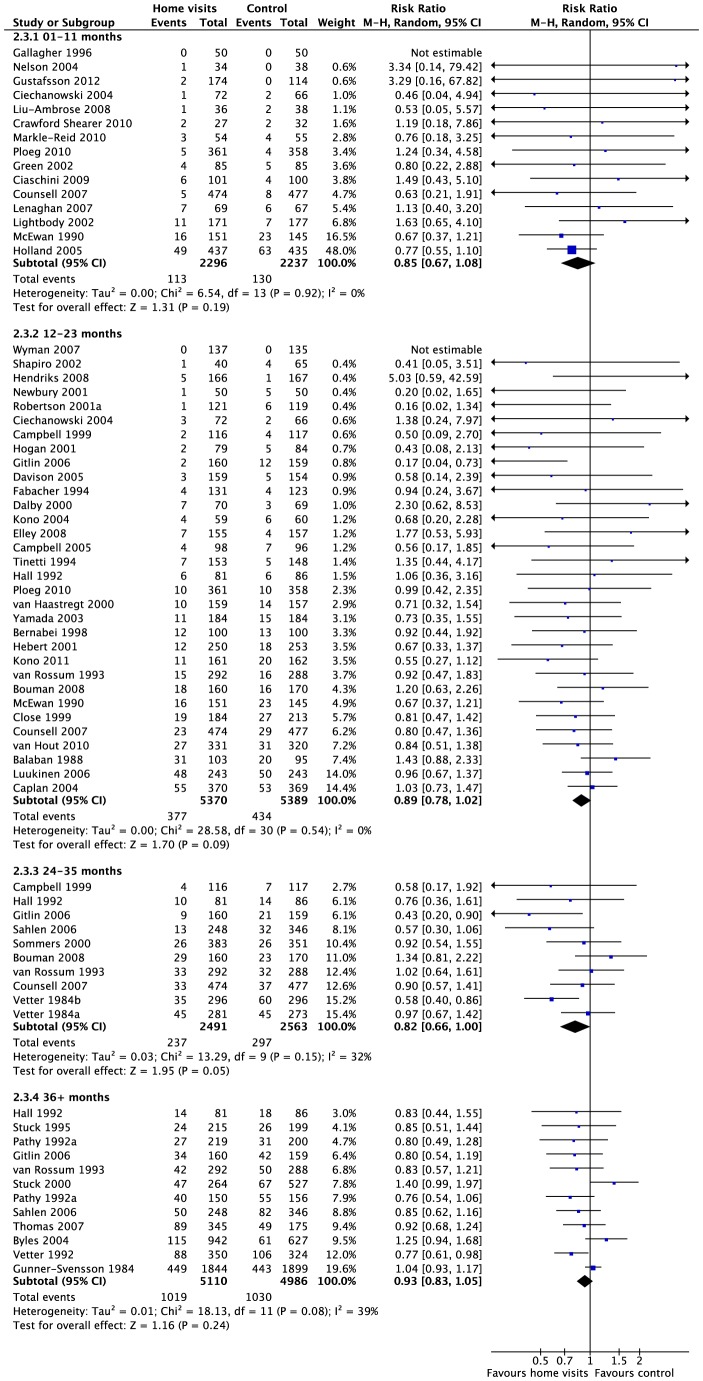
Mortality at each follow-up. All-cause mortality for studies reporting deaths up to 1 year, 2 years, 3 years, and more than 3 years after the start of the trial.

**Figure 2:**
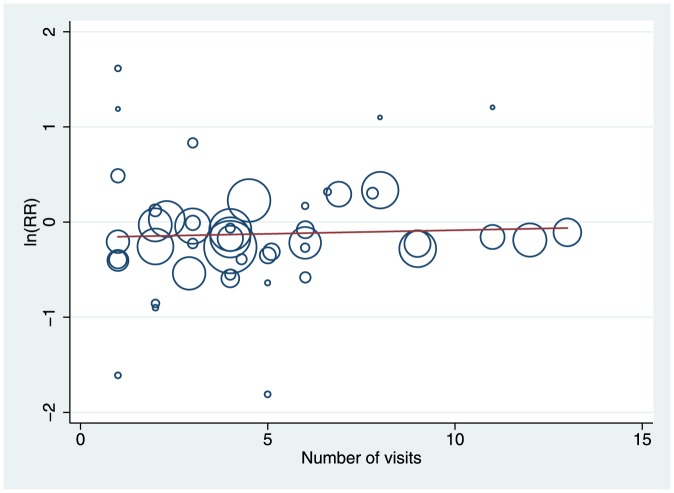
Mortality meta-regression by number of visits. Circles represent studies (N = 47), size represents weight in the analysis. The slope was not significant (0.008 [95% CI −0.02 to 0.04]; t = 0.53, p = 0.60).

Fifty-three studies (83%) with 23826 participants (83%) reported effects for all-cause mortality. There was high quality evidence of a small relative effect (weighted average) at longest follow-up (Risk ratio = 0.93 [0.87, 0.99]; Chi^2^ = 54.89, df = 53, p = 0.40; I^2^ = 3%), but the absolute difference in mortality was close to zero and unlikely to be clinically important (risk difference = 0.00 [−0.01, 0.00]; Chi^2^ = 64.72, df = 55, p = 0.17; I^2^ = 15%). Effects for specified follow-up periods were similar to the effect at longest follow-up (Figure 1). Meta-regression did not identify any effects for age (Figure S4) or number of visits (Figure 2); there was a significant relationship with baseline risk of mortality (i.e. annualized risk in the control group), but the relationship was small and unimportant for most studies within the observed range (Figure S4). There was no difference among subgroups when we compared studies by focus of intervention, average age, or number of visits; however, the effect for interventions including both MGA and falls prevention was larger than the effect for either alone. There was some heterogeneity across types of visitors; there was no overall benefit of interventions delivered exclusively by nurses (Figure S5). The data are available as a RevMan 5.2 file (Dataset S1).

#### Institutionalization

**Figure 3:**
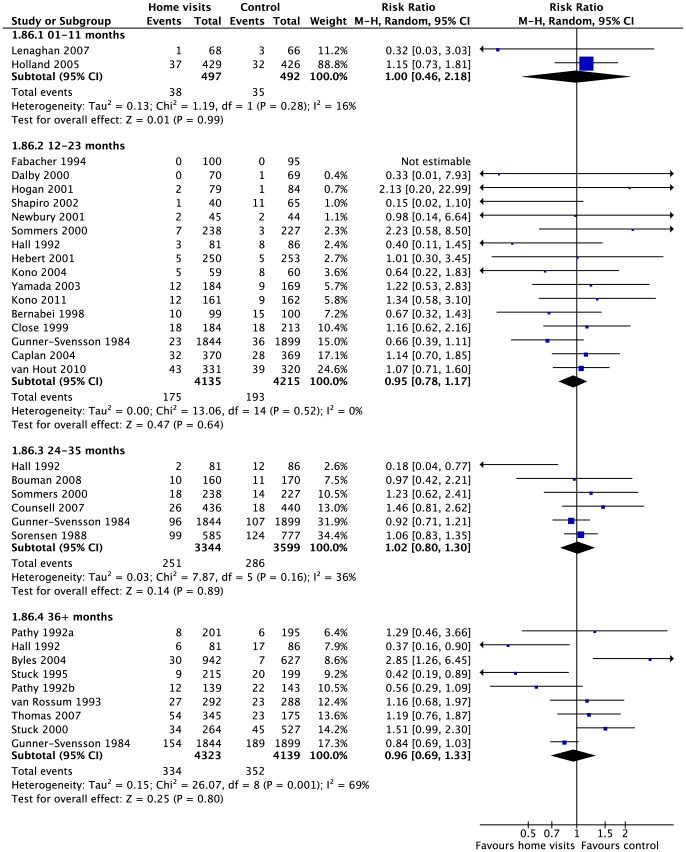
Institutionalisation at each follow-up. Number of people living in institution up to 1 year, 2 years, 3 years, and more than 3 years after the start of the trial.

Twenty-six studies (41%) with 16264 participants (57%) reported effects for the number of participants in each group who were admitted to an institution. There was moderate quality evidence of no clinically important difference at longest follow-up in relative effects (risk ratio = 1.02 [0.88, 1.18]; Chi^2^ = 37.64, df = 26, p = 0.07; I^2^ = 31%) or absolute effects (risk difference = 0.00 [−0.01, 0.01]; Chi^2^ = 45.13, df = 27, p = 0.02; I^2^ = 40%). Effects for specified follow-up periods were similar to the effect at longest follow-up (Figure 3). Meta-regression did not identify any effects for age, number of visits, or risk of mortality. There was no evidence of any differences by time point, focus of visit, age of participants, type of visitor, or number of visits (Table 1).

**Table 1:** Institutionalisation (overall effect and subgroup analyses).

		*Home visits*		*Control*			
*Subgroup*	*Trials*	*Events*	*People*	*Events*	*People*	*Risk Ratio (95% CI), random effects*	*Heterogeneity I^2^; Chi^2^ (p value)*
All studies	26	667	8111	700	8153	1.02 (0.88 to 1.18)	31%; 37.64 (p = 0.07)
*Focus of intervention* (I^2^ = 0%; Chi^2^ = 0.71, p = 0.87)							
*Falls*	*1*	*2*	*79*	*1*	*84*	*2.13 (0.20 to 22.99)*	*Not applicable*
*MGA*	*19*	*552*	*6791*	*596*	*6831*	*0.99 (0.82 to 1.20)*	*46%; 35.34 (p = 0.01)*
*Both*	*4*	*75*	*844*	*68*	*841*	*1.11 (0.81 to 1.51)*	*0%; 0.13 (p = 0.99)*
*Neither*	*2*	*38*	*497*	*35*	*492*	*1.00 (0.46 to 2.18)*	*16%; 1.19 (p = 0.28)*
*Average age* (I^2^ = 0%; Chi^2^ = 1.46, p = 0.69)							
*≤70*	*1*	*8*	*201*	*6*	*195*	*1.29 (0.46 to 3.66)*	*Not applicable*
*71–75*	*1*	*26*	*536*	*18*	*535*	*1.46 (0.81 to 2.62)*	*Not applicable*
*76–80*	*15*	*254*	*3450*	*274*	*3377*	*1.03 (0.81 to 1.31)*	*30%; 19.90 (p = 0.13)*
*81–85*	*9*	*225*	*2180*	*213*	*2242*	*1.02 (0.80 to 1.29)*	*35%; 12.33 (p = 0.14)*
*Type of visitor* (I^2^ = 49.2%; Chi^2^ = 3.94, p = 0.14)							
*Nurse*	*15*	*394*	*4550*	*431*	*4693*	*0.95 (0.77 to 1.16)*	*36%; 21.93 (p = 0.08)*
*Other*	*5*	*86*	*1120*	*96*	*1143*	*0.92 (0.68 to 1.24)*	*9%; 5.48 (p = 0.36)*
*Combined*	*6*	*187*	*2541*	*173*	*2412*	*1.29 (0.99 to 1.69)*	*18%; 6.13 (p = 0.29)*
*Number of visits* (I^2^ = 0%; Chi^2^ = 0.05, p = 0.98)							
*1*	*4*	*124*	*1064*	*149*	*1287*	*1.07 (0.86 to 1.33)*	*0%; 0.08 (p = 0.99)*
*2–4*	*9*	*186*	*2012*	*144*	*1826*	*1.10 (0.90 to 1.36)*	*0%; 3.44 (p = 0.90)*
*5 or more*	*10*	*196*	*3170*	*190*	*3085*	*1.08 (0.80 to 1.45)*	*51%; 20.46 (p = 0.03)*

Legend: Overall effect on institutionalisation, effects for each subgroup, and tests for differences among subgroups.

#### Hospitalization

Fifteen studies (23%) including 6288 participants (22%) reported the number of people admitted to hospital in each group (Table 2). At longest follow-up, there was moderate quality evidence of a small relative effect (risk ratio = 0.96 [0.91, 1.01]; Chi^2^ = 13.70, df = 14, p = 0.47; I^2^ = 0%) that may not be clinically important (risk difference = −0.01 [−0.03, 0.00]; Chi^2^ = 13.89, df = 14, p = 0.46; I^2^ = 0%). There was no evidence of any differences among subgroups, except a statistically significant difference among types of visitors that was consistent with the results for mortality.

**Table 2:** Outcomes at longest follow-up.

		*Participants (events)*			
*Outcomes*	*Trials*	*Home visits*	*Control*	*ES (95% CI), random effects*	*Heterogeneity I^2^; Chi^2^ (p value)*
Mortality	53	12008 (1589)	11818 (1672)	Risk = 0.93 (0.87 to 0.99)	3%; 54.89 (p = 0.40)
Institutionalization					
*People admitted*	26	8111 (667)	8153 (700)	Risk = 1.02 (0.88 to 1.18)	31%; 37.64 (p = 0.07)
*Days in institution*	4	766	757	Rate = 0.78 (0.76 to 0.80)	100%; 2198.79 (p<0.001)
Hospitalization					
*People admitted*	15	3167	3121	Risk = 0.96 (0.91 to 1.01)	0%; 13.70 (p = 0.47)
*Admissions*	11	2476	2467	Rate = 0.93 (0.81 to 1.06)	61%; 28.07 (p = 0.003)
*Days in hospital*	12	2303	2270	Rate = 0.85 (0.71 to 1.02)	99%; 909.84 (p<0.001)
*People attending ER*	12	2180	2141	Risk = 0.91 (0.81 to 1.03)	32%; 16.29 (p = 0.13)
*ER visits*	10	2632	3238	Rate = 0.92 (0.81 to 1.04)	75%; 35.81 (p<0.001)
Falls					
*People who fell*	23	3407	4048	Odds = 0.86 (0.73 to 1.01)	50%; 43.59 (p = 0.004)
*Number of falls*	15	2344	2975	Rate = 0.74 (0.63 to 0.86)	88%; 113.04 (p<0.001)
*Fear of falling*	14	1349	1225	SMD = −0.16 (−0.26 to −0.07)	29%; 18.26 (p = 0.15)
Injuries					
*People injured*	10	1524	1531	Risk = 0.77 (0.63 to 0.95)	0%; 7.24 (p = 0.61)
*Number of injuries*	7	1558	2160	Rate = 0.98 (0.87 to 1.11)	0%; 4.32 (p = 0.63)
Physical functioning	27	4296	4473	SMD = −0.10 (−0.17 to −0.03)	53%; 55.40 (p<0.001)
Cognitive functioning	8	852	756	SMD = −0.06 (−0.21 to 0.09)	44%; 12.49 (p = 0.09)
Quality of life	29	5136	4756	SMD = −0.06 (−0.11 to −0.01)	22%; 35.69 (p = 0.15)
Psychiatric illness	15	1676	1642	SMD = −0.10 (−0.18 to −0.02)	22%; 18.06 (p = 0.20)

Legend: Overall effects at longest follow-up. Rate ratio (Rate); Risk ratio (Risk); Odds Ratio (Odds); Standardised Mean Difference (SMD).

Eleven studies (17%) including 4943 participants (17%) reported the number of admissions to hospital. There was low quality evidence at longest follow-up, which would be consistent with no effect or a small effect (rate ratio = 0.93 [0.81, 1.06]; Chi^2^ = 28.07, df = 11, p = 0.003; I^2^ = 61%). There was no evidence of any differences among subgroups except a significant difference among types of visitors that was not consistent with the results for people admitted to hospital or mortality.

Twelve studies (19%) including 4321 participants (15%) reported the number of people who visited an emergency room in each group. There was moderate quality evidence at longest follow-up, which would be consistent with no effect or a small relative effect (risk ratio = 0.91 [0.81, 1.03]; Chi^2^ = 16.29, df = 11, p = 0.13; I^2^ = 32%). There was no evidence of any differences among subgroups.

Ten studies (16%) including 5870 participants (20%) reported the number of emergency room visits. There was low quality evidence at longest follow-up, which would be consistent with no effect or a small effect (rate ratio = 0.92 [0.81, 1.04]; Chi^2^ = 35.81, df = 9, p<0.0001; I^2^ = 75%). Several differences across subgroups were statistically significant because one group in several analyses included only one study that was inconsistent with others; there were no meaningful differences.

#### Falls

Twenty-three studies (36%) including 7455 (26%) participants reported the number of people who fell. One study reported an adjusted effect that could not be combined with other measures to estimate a relative risk, so an overall odds ratio was calculated [96]. There was moderate quality evidence of a small effect at longest follow-up, but it was not statistically significant (odds ratio = 0.86 [0.73, 1.01]; Chi^2^ = 43.59, df = 22, p = 0.004; I^2^ = 50%). Most effects were measured after about 12 months; two studies reporting longer follow-up found no evidence of extended benefits. Meta-regression did not identify any effects for age, number of visits, or risk of mortality. There was no evidence of any differences among subgroups, though only one study reported falls but did not explicitly target falls prevention.

Fifteen studies (23%) including 5319 (19%) participants reported the number of falls. There was low quality evidence of a small effect at longest follow-up (rate ratio = 0.74 [0.58, 0.93]), but as with days in hospital or days in institution, the results were extremely inconsistent (Chi^2^ = 4574.87, df = 14, p<0.00001; I^2^ = 100%). Some subgroups analyses suggested statistical differences among groups, but studies within these groups were also highly heterogeneous; that is, differences between subgroups did not appear to explain the observed heterogeneity.

#### Physical and cognitive functioning

Twenty-seven studies (42%) including 8769 (31%) participants reported a measure of functioning activities of daily living (ADL) or instrumental activities of daily living (IADL). Several studies reported the number of people dependent or independent (or having difficulty) in specific activities (e.g., eating or dressing), but did not report an estimate of overall functioning [79]. There was very low quality evidence of a small effect on ADLs and IADLs at longest follow-up (SMD = −0.10 [−0.17, −0.03]; Chi^2^ = 55.40, df = 26, p<0.001; I^2^ = 53%). Meta-regression did not identify any effects for age, number of visits, or risk of mortality. There was no evidence of any differences among subgroups.

Eight studies (13%) including 1608 (6%) of participants reported a measure of cognitive functioning. There was low quality evidence of no clinically important difference at longest follow-up (SMD = −0.06 [−0.21, 0.09]; Chi^2^ = 12.49, df = 7, p = 0.09; I^2^ = 44%). We did not compare subgroups due to a lack of studies.

#### Quality of life

Twenty-nine studies (45%) including 9892 participants (35%) reported any measure of health-related quality of life. There was low quality evidence of no clinically important difference at longest follow-up (SMD = −0.06 [−0.11, −0.01], Chi^2^ = 35.69, df = 28, p = 0.15; I^2^ = 22%). There was no evidence of any significant differences among subgroups.

#### Psychiatric illness (anxiety and depression)

Fifteen studies (23%) including 3318 participants (12%) reported psychiatric illness (anxiety or depression). There was low quality evidence of a small effect at longest follow-up (SMD = −0.10 [−0.18, 0.02]; Chi^2^ = 18.06, df = 14, p = 0.20; I^2^ = 22%). There was no evidence of any differences among subgroups.

#### Additional analyses

Additional analyses identified no evidence of important benefits for: days in institution, days in hospital, fear of falling, people injured, and number of injuries. These outcomes were infrequently reported and many were heterogeneous (Table 2); subgroup analyses did not reveal any patterns that were inconsistent with the results above (Figure S5).

## Discussion

Over the past 20 years, many reviews have investigated the effects of preventive home visiting. Some analyses conclude that comprehensive geriatric assessment may have several benefits [14], [199], but other reviews have come to conflicting conclusions [6], [7], [9], [12], [200], [201] guidelines reflect this uncertainty [13], but this review finds high quality evidence that preventive home visits do not have important effects on mortality or on independent living overall (Table S1). Future guidelines might recommend against these interventions as they do not have proven effects.

Including 64 randomized trials conducted over the three decades, this review is the most comprehensive in scope, and it identified several studies not included in previous analyses. The results include a wide range of outcomes, the main results are statistically precise with little evidence of statistical heterogeneity, and broad inclusion criteria facilitated several pre-specified subgroup analyses. The quality of evidence was high for mortality and moderate for other critical outcomes, and we conclude that these interventions are ineffective overall; however, we cannot exclude the possibility that some programs may be associated with benefits.

Some home visits are part of larger programs that might have positive effects, including exercise, improved assessment by medical professionals, or falls prevention. However, no specific components appeared to distinguish effective programs from ineffective programs for mortality and institutionalization. Consistent with a recent review of interventions to prevent falls [7], we find some evidence that home visits may reduce risk of falling; however, many studies did not have a pre-defined primary outcome, and most studies were not prospectively registered. We interpret these results cautiously because other included studies may have measured proximal outcomes and failed to report them. Furthermore, there was no evidence of effects on distal outcomes among those studies that reported reductions in falls.

An overview of reviews identified a need for further analyses to investigate differences related to the focus of visits, number of visits, characteristics of participants, and characteristics of providers [202]. To the extent possible, this review investigated these variables and failed to identify any patterns across outcomes that would be consistent with benefits overall or consistent with benefits for any defined subgroup. Limited reporting of intervention implementation prevented further investigation into potential mediators and moderators (Figure S6). It is possible that some combination of components in particular populations and settings could yield benefits; however, most studies failed to describe a clear program theory [10]. Some interventions included mostly assessment and recommendations; the efficacy of such interventions depends on adherence to these recommendations (e.g., removing fall hazards, exercising, changing medications) and complementary care. Included trials rarely reported participant compliance and use of other services during the trial. As many of the programs required contacts with local health services, it is impossible to assess effects without a description of usual care for each site at each point in time.

Poorly reported trials waste scare resources [203], [204] and can exacerbate difficulties in systematic reviews of complex interventions [203], [204], yet under-reporting of intervention content and delivery is common [205]–[207]. This review cannot demonstrate if home visits delivered as intended will have insignificant effects; null effects could be related to implementation failure. To produce useful evidence about the effects of complex interventions, researchers must adhere to CONSORT guidelines for reporting trials [204], [208]; report all outcomes measured [209]; and clearly describe program design, delivery, and uptake so that intervention components can be fully considered in systematic reviews [210].

Given the size of this review and the number of previous reviews on this topic, further small studies comparing multi-component preventive home visits to usual care will add very little to the knowledge base. Only a very large trial—or a program of research leading to one—could be justified at this time. Funders should not support further trials unless researchers can explain how new studies would change conclusions drawn from a large body of existing evidence.

## Conclusion

Home visiting is not consistently associated with differences in mortality or independent living, and investigations of heterogeneity did not identify any subset of programs that are associated with consistent benefits. Due to poor reporting of intervention components and delivery, we cannot exclude the possibility that some programs may be effective. If researchers continue to evaluate these types of interventions, they should begin with clear theories of change, clearly describe programmes and their implementation, and report all outcomes measured [202], [211], [212].

## Supporting Information

Figure S1 **PRISMA Chart.**
(TIFF)

Figure S2 **Risk of bias.**
(TIFF)

Figure S3 **Main outcomes and tests for reporting bias.**
(TIFF)

Figure S4 **Meta-regression analyses.**
(TIFF)

Figure S5 **Results for each follow-up interval, longest follow-up, and subgroup analyses.**
(PDF)

Figure S6 **Extended table of included studies.**
(PDF)

Table S1 **Summary of findings table.**
(DOCX)

Table S2 **Table of included studies.**
(DOCX)

Table S3 **Table of excluded studies.**
(DOCX)

Text S1 **Complete search strategies.**
(DOCX)

Dataset S1 **RevMan 5.2 file.**
(RM5)

Checklist S1 (DOC)
