# Supplementary figures and images for: Preventive Home Visits for Mortality, Morbidity, and Institutionalization in Older Adults: A Systematic Review and Meta-Analysis
Source: PLoS One. 2014 Mar 12;9(3):e89257. doi: 10.1371/journal.pone.0089257 (PMC3951196; doi:10.1371/journal.pone.0089257)

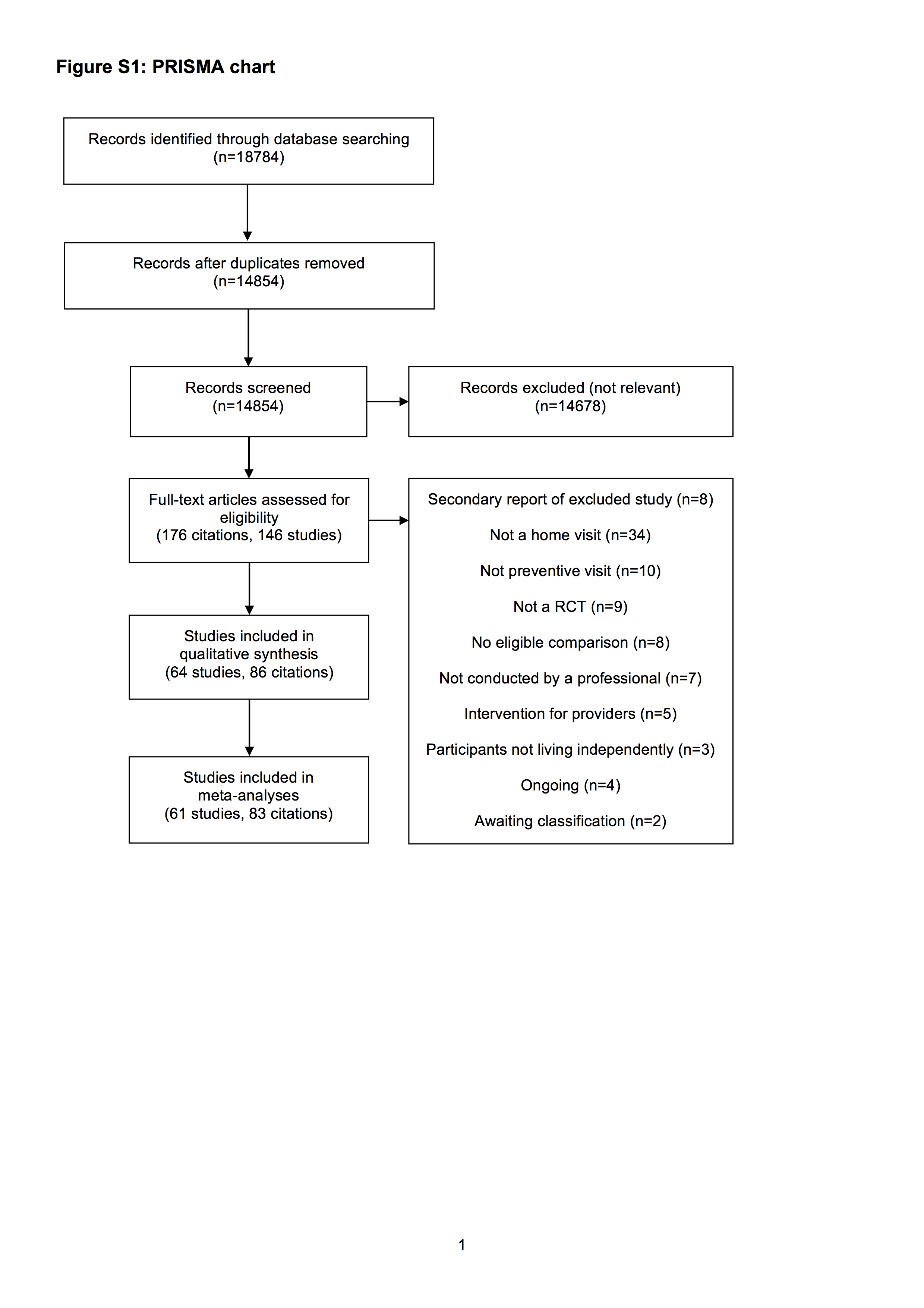

Supplement: Figure S1 — PRISMA Chart. (TIFF) [file pone.0089257.s001.tiff]

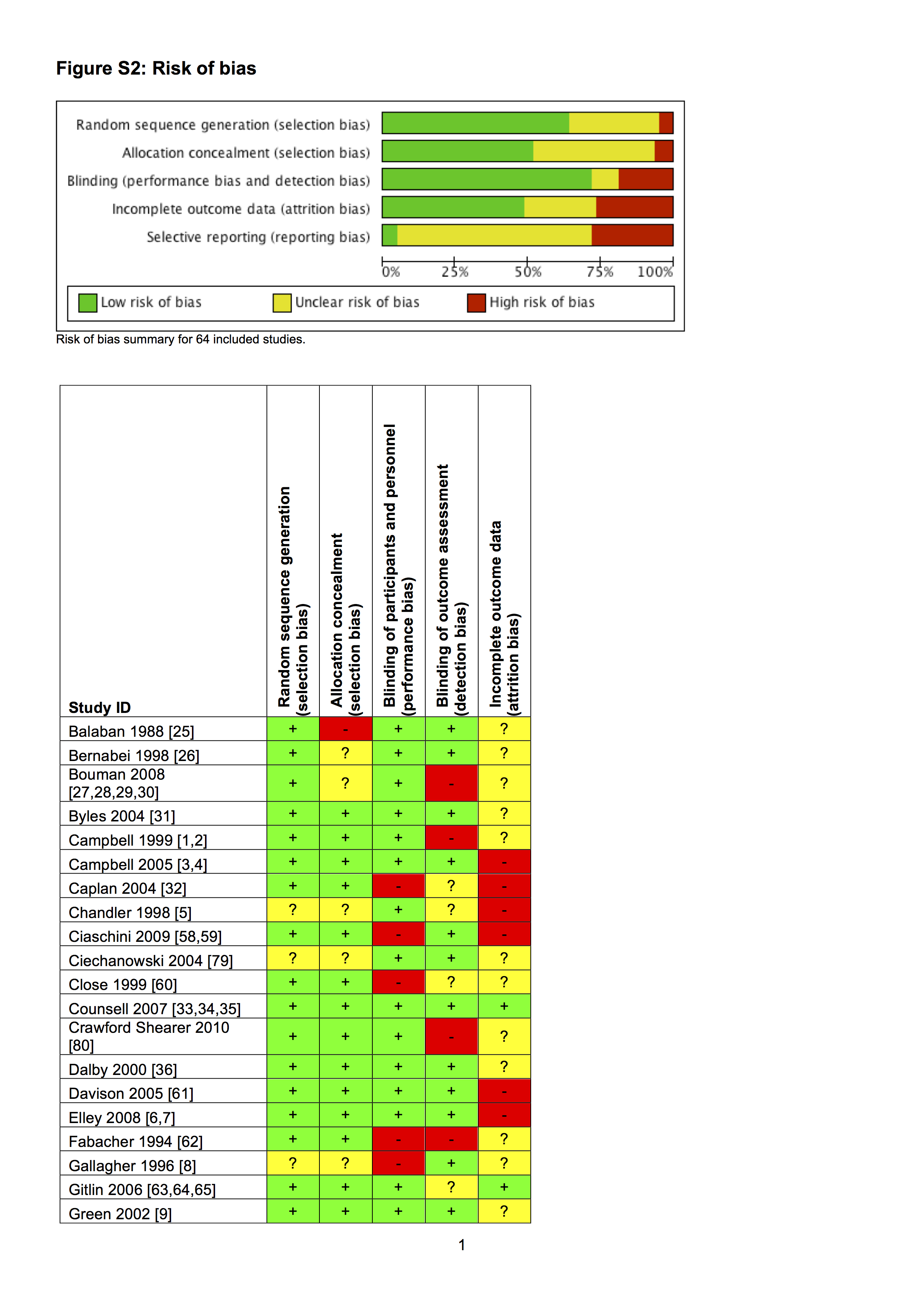

Supplement: Figure S2 — Risk of bias. (TIFF) [file pone.0089257.s002.tiff]

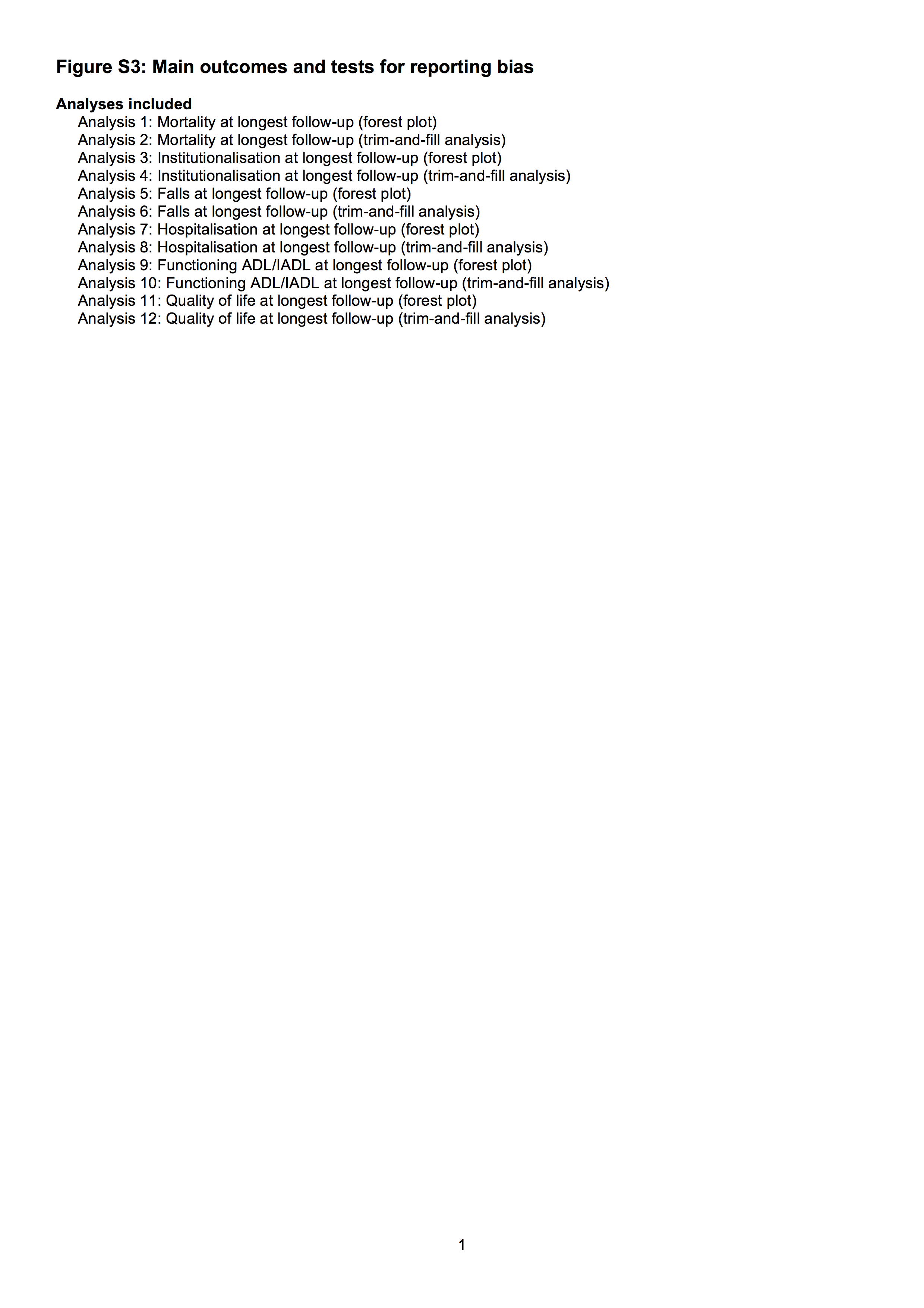

Supplement: Figure S3 — Main outcomes and tests for reporting bias. (TIFF) [file pone.0089257.s003.tiff]

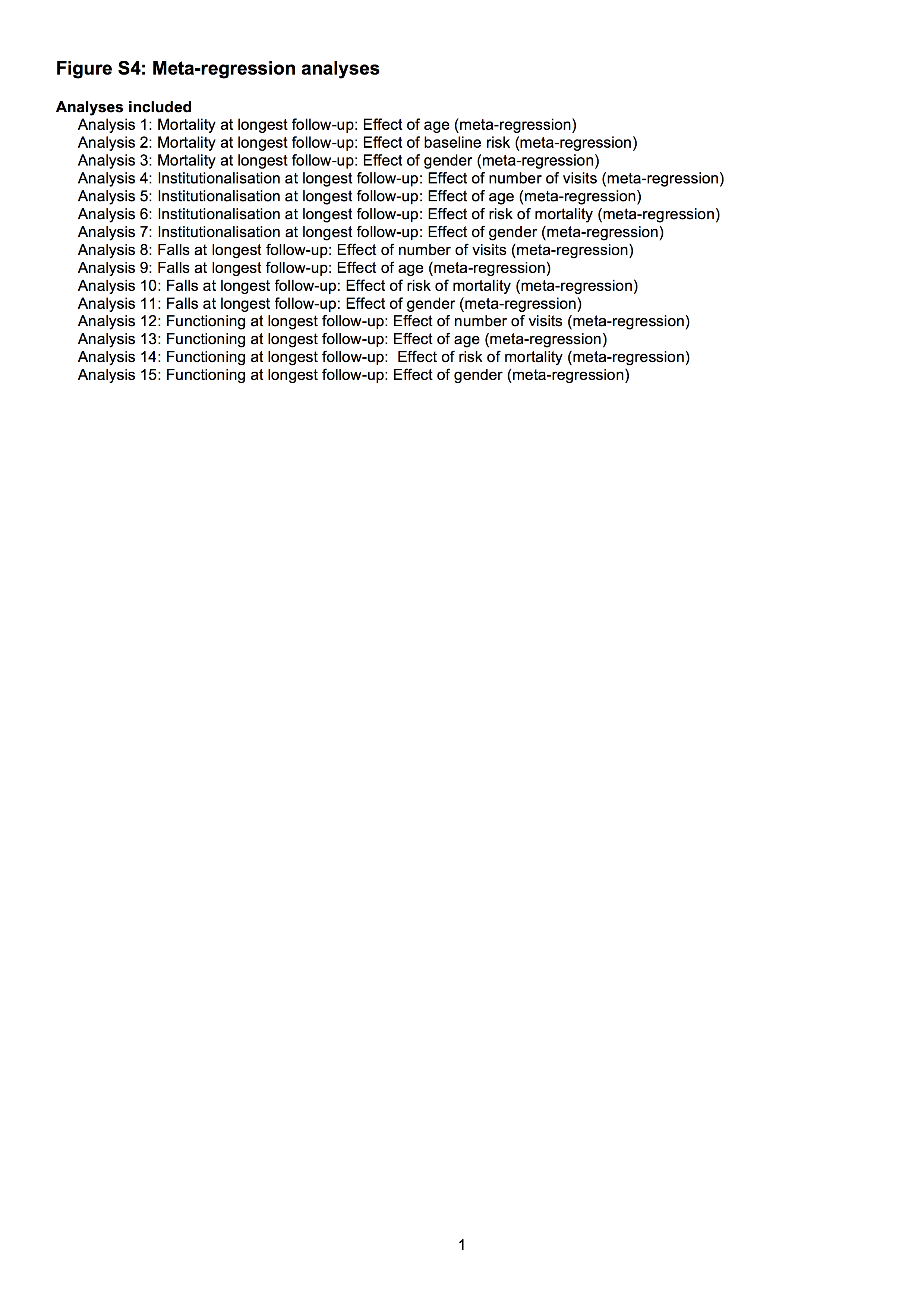

Supplement: Figure S4 — Meta-regression analyses. (TIFF) [file pone.0089257.s004.tiff]
