# Supplementary material for: Preventive Home Visits for Mortality, Morbidity, and Institutionalization in Older Adults: A Systematic Review and Meta-Analysis
Source: PLoS One. 2014 Mar 12;9(3):e89257. doi: 10.1371/journal.pone.0089257 (PMC3951196; doi:10.1371/journal.pone.0089257)

Figure S5: Results for each follow-up interverval, longest follow-up, and subgroup analyses

Analyses included

Analysis 1: ER (number); results at longest follow-up
Analysis 2: ER (number); at each follow-up interval
Analysis 3: ER (number); focus of visit subgroups
Analysis 4: ER (number); age of participants subgroups
Analysis 5: ER (number); type of visitor subgroups
Analysis 6: ER (number); number of visits subgroups
Analysis 7: ER (people); results at longest follow-up
Analysis 8: ER (people); at each follow-up interval
Analysis 9: ER (people); focus of visit subgroups
Analysis 10: ER (people); age of participants subgroups
Analysis 11: ER (people); type of visitor subgroups
Analysis 12: ER (people); number of visits subgroups
Analysis 13: Falls (number); results at longest follow-up
Analysis 14: Falls (number); at each follow-up interval
Analysis 15: Falls (number); focus of visit subgroups
Analysis 16: Falls (number); age of participants subgroups
Analysis 17: Falls (number); type of visitor subgroups
Analysis 18: Falls (number); number of visits subgroups
Analysis 19: Falls (people) at each follow-up interval
Analysis 20: Falls (people); focus of visit subgroups
Analysis 21: Falls (people); age of participants subgroups
Analysis 22: Falls (people); type of visitor subgroups
Analysis 23: Falls (people); number of visits subgroups
Analysis 24: Falls (subjective), results at longest follow-up
Analysis 25: Falls (subjective) at each follow-up interval
Analysis 26: Falls (subjective); focus of visit subgroups
Analysis 27: Falls (subjective); age of participants subgroups
Analysis 28: Falls (subjective); type of visitor subgroups
Analysis 29: Falls (subjective); number of visits subgroups
Analysis 30: Functioning ADL/IADL at each follow-up interval
Analysis 31: Functioning ADL/IADL; focus of visit subgroups
Analysis 32: Functioning ADL/IADL; age of participants subgroups
Analysis 33: Functioning ADL/IADL; type of visitor subgroups
Analysis 34: Functioning ADL/IADL; number of visits subgroups
Analysis 35: Cognitive functioning, results at longest follow-up
Analysis 36: Quality of life at each follow-up interval
Analysis 37: Quality of life; focus of visit subgroups
Analysis 38: Quality of life; age of participants subgroups
Analysis 39: Quality of life; type of visitor subgroups
Analysis 40: Quality of life; number of visits subgroups
Analysis 41: Hospitalisation Admissions, results at longest follow-up
Analysis 42: Hospitalisation Admissions at each follow-up interval
Analysis 43: Hospitalisation Admissions; focus of visit subgroups
Analysis 44: Hospitalisation Admissions; age of participants subgroups
Analysis 45: Hospitalisation Admissions; type of visitor subgroups
Analysis 46: Hospitalisation Admissions; number of visits subgroups
Analysis 47: Hospitalisation (days), results at longest follow-up
Analysis 48: Hospitalisation (days) at each follow-up interval
Analysis 49: Hospitalisation (days); focus of visit subgroups
Analysis 50: Hospitalisation (days); age of participants subgroups
Analysis 51: Hospitalisation (days); type of visitor subgroups
Analysis 52: Hospitalisation (days); number of visits subgroups
Analysis 53: Hospitalisation (people) at each follow-up interval
Analysis 54: Hospitalisation (people); focus of visit subgroups
Analysis 55: Hospitalisation (people); age of participants subgroups
Analysis 56: Hospitalisation (people); type of visitor subgroups
Analysis 57: Hospitalisation (people); number of visits subgroups
Analysis 58: Injuries (number), results at longest follow-up
Analysis 59: Injuries (people), results at longest follow-up
Analysis 60: Injuries (people) at each follow-up interval
Analysis 61: Injuries (people); focus of visit subgroups
Analysis 62: Injuries (people); age of participants subgroups

Analysis 63: Injuries (people); type of visitor subgroups
Analysis 64: Injuries (people); number of visits subgroups
Analysis 65: Institutionalisation (days), results at longest follow-up
Analysis 66: Institutionalisation (people) at each follow-up interval
Analysis 67: Institutionalisation (people); focus of visit subgroups
Analysis 68: Institutionalisation (people); age of participants subgroups
Analysis 69: Institutionalisation (people); type of visitor subgroups
Analysis 70: Institutionalisation (people); number of visits subgroups
Analysis 71: Mortality (Focus of visits subgroups)
Analysis 72: Mortality; age of participants subgroups
Analysis 73: Mortality; type of visitor subgroups
Analysis 74: Mortality; number of visits subgroups
Analysis 75: Psychiatric (anxiety and depression), results at longest follow-up
Analysis 76: Psychiatric (anxiety and depression) at each follow-up interval
Analysis 77: Psychiatric (anxiety and depression); focus of visit subgroups
Analysis 78: Psychiatric (anxiety and depression); age of participants subgroups
Analysis 79: Psychiatric (anxiety and depression); type of visitor subgroups
Analysis 80: Psychiatric (anxiety and depression); number of visits subgroups

Analysis 1: ER (number); results at longest follow-up

Study or Subgroup	log[Rate Ratio]	SE	Home visits Total	Control Total	Weight	Rate Ratio IV, Random, 95% CI
van Haastregt 2000	0.3791478	0.34503278	120	115	3.1%	1.46 [0.74, 2.87]
Robertson 2001a	−0.40578434	0.30662207	121	119	3.8%	0.67 [0.37, 1.22]
Tinetti 1994	−0.39176342	0.26034166	147	144	4.9%	0.68 [0.41, 1.13]
Dalby 2000	−0.23794986	0.25436154	70	69	5.1%	0.79 [0.48, 1.30]
Campbell 2005	−0.10167807	0.25413251	98	96	5.1%	0.90 [0.55, 1.49]
Stevens 2001	−0.02020271	0.20078915	635	1244	7.1%	0.98 [0.66, 1.45]
Holland 2005	0.25147785	0.09945593	437	435	14.3%	1.29 [1.06, 1.56]
Caplan 2004	−0.2555538	0.07224321	370	369	16.8%	0.77 [0.67, 0.89]
Counsell 2007	−0.18817955	0.03555452	474	477	19.9%	0.83 [0.77, 0.89]
Bouman 2008	0	0.03541811	160	170	19.9%	1.00 [0.93, 1.07]
**Total (95% CI)**			**2632**	**3238**	**100.0%**	**0.92 [0.81, 1.04]**

Heterogeneity: $Tau^2 = 0.02$; $Chi^2 = 35.81$, df = 9 ($P < 0.0001$); $I^2 = 75\%$
Test for overall effect: Z = 1.31 (P = 0.19)

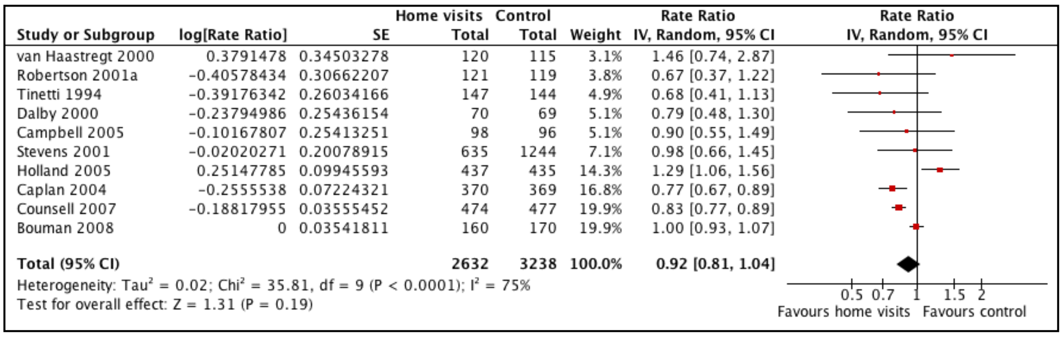

Analysis 2: ER (number); at each follow-up interval

Study or Subgroup	log[Rate Ratio]	SE	Home visits Total	Control Total	Weight	Rate Ratio IV, Random, 95% CI
**1.2.1 01-11 months**						
Holland 2005	0.25147785	0.09945593	437	435	100.0%	1.29 [1.06, 1.56]
**Subtotal (95% CI)**			**437**	**435**	**100.0%**	**1.29 [1.06, 1.56]**
Heterogeneity: Not applicable						
Test for overall effect: Z = 2.53 (P = 0.01)						
**1.2.2 12-23 months**						
van Haastregt 2000	0.3791478	0.34503278	120	115	2.4%	1.46 [0.74, 2.87]
Robertson 2001a	−0.40578434	0.30662207	121	119	2.9%	0.67 [0.37, 1.22]
Tinetti 1994	−0.39176342	0.26034166	147	144	3.9%	0.68 [0.41, 1.13]
Dalby 2000	−0.23794986	0.25436154	70	69	4.1%	0.79 [0.48, 1.30]
Campbell 2005	−0.10167807	0.25413251	98	96	4.1%	0.90 [0.55, 1.49]
Stevens 2001	−0.02020271	0.20078915	635	1244	6.1%	0.98 [0.66, 1.45]
Caplan 2004	−0.2555538	0.07224321	370	369	21.0%	0.77 [0.67, 0.89]
Counsell 2007	−0.125449	0.04777345	474	477	26.4%	0.88 [0.80, 0.97]
Bouman 2008	0	0.03541811	160	170	29.0%	1.00 [0.93, 1.07]
**Subtotal (95% CI)**			**2195**	**2803**	**100.0%**	**0.89 [0.80, 0.99]**
Heterogeneity: $Tau^2 = 0.01$; $Chi^2 = 16.71$, df = 8 ($P = 0.03$); $I^2 = 52\%$						
Test for overall effect: Z = 2.19 (P = 0.03)						
**1.2.3 24-35 months**						
Counsell 2007	−0.18817955	0.03555452	474	477	50.0%	0.83 [0.77, 0.89]
Bouman 2008	0	0.03541811	160	170	50.0%	1.00 [0.93, 1.07]
**Subtotal (95% CI)**			**634**	**647**	**100.0%**	**0.91 [0.76, 1.09]**
Heterogeneity: $Tau^2 = 0.02$; $Chi^2 = 14.06$, df = 1 ($P = 0.0002$); $I^2 = 93\%$						
Test for overall effect: Z = 1.00 (P = 0.32)						

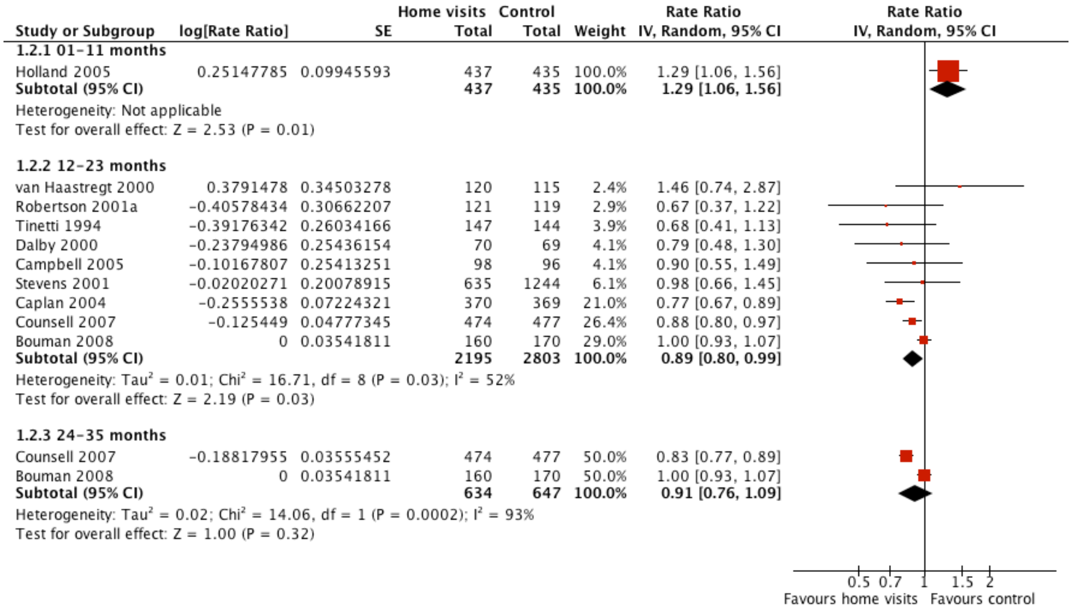

Analysis 3: ER (number); focus of visit subgroups

Study or Subgroup	log[Rate Ratio]	SE	Home visits Total	Control Total	Weight	Rate Ratio IV, Random, 95% CI
**1.3.1 Falls Only**						
Robertson 2001a	−0.40578434	0.30662207	121	119	3.8%	0.67 [0.37, 1.22]
Campbell 2005	−0.10167807	0.25413251	98	96	5.1%	0.90 [0.55, 1.49]
Stevens 2001	−0.02020271	0.20078915	635	1244	7.1%	0.98 [0.66, 1.45]
**Subtotal (95% CI)**			**854**	**1459**	**16.0%**	**0.88 [0.67, 1.16]**
Heterogeneity: Tau² = 0.00; Chi² = 1.12, df = 2 (P = 0.57); I² = 0%						
Test for overall effect: Z = 0.90 (P = 0.37)						
**1.3.2 MGA Only**						
Dalby 2000	−0.23794986	0.25436154	70	69	5.1%	0.79 [0.48, 1.30]
Caplan 2004	−0.2555538	0.07224321	370	369	16.8%	0.77 [0.67, 0.89]
Counsell 2007	−0.18817955	0.03555452	474	477	19.9%	0.83 [0.77, 0.89]
Bouman 2008	0	0.03541811	160	170	19.9%	1.00 [0.93, 1.07]
**Subtotal (95% CI)**			**1074**	**1085**	**61.7%**	**0.86 [0.75, 1.00]**
Heterogeneity: Tau² = 0.01; Chi² = 18.78, df = 3 (P = 0.0003); I² = 84%						
Test for overall effect: Z = 2.01 (P = 0.04)						
**1.3.3 Both**						
van Haastregt 2000	0.3791478	0.34503278	120	115	3.1%	1.46 [0.74, 2.87]
Tinetti 1994	−0.39176342	0.26034166	147	144	4.9%	0.68 [0.41, 1.13]
**Subtotal (95% CI)**			**267**	**259**	**8.0%**	**0.96 [0.45, 2.04]**
Heterogeneity: Tau² = 0.20; Chi² = 3.18, df = 1 (P = 0.07); I² = 69%						
Test for overall effect: Z = 0.10 (P = 0.92)						
**1.3.4 Neither**						
Holland 2005	0.25147785	0.09945593	437	435	14.3%	1.29 [1.06, 1.56]
**Subtotal (95% CI)**			**437**	**435**	**14.3%**	**1.29 [1.06, 1.56]**
Heterogeneity: Not applicable						
Test for overall effect: Z = 2.53 (P = 0.01)						
**Total (95% CI)**			**2632**	**3238**	**100.0%**	**0.92 [0.81, 1.04]**
Heterogeneity: Tau² = 0.02; Chi² = 35.81, df = 9 (P < 0.0001); I² = 75%						
Test for overall effect: Z = 1.31 (P = 0.19)						
Test for subgroup differences: Chi² = 11.03, df = 3 (P = 0.01), I² = 72.8%						

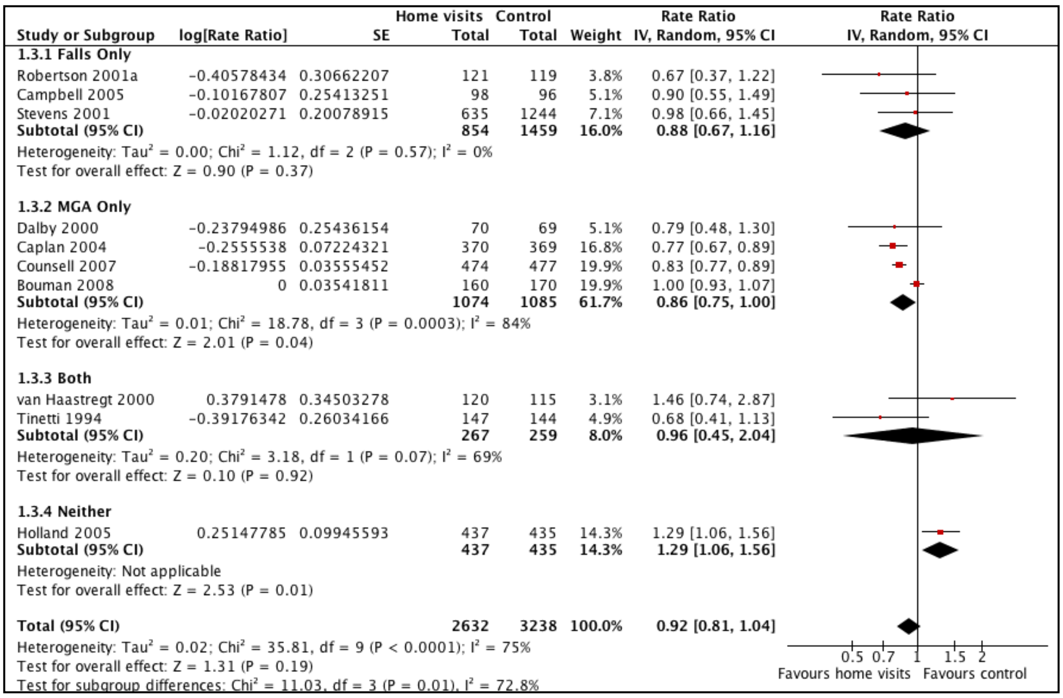

Analysis 4: ER (number); age of participants subgroups

Study or Subgroup	log[Rate Ratio]	SE	Home visits Total	Control Total	Weight	Rate Ratio IV, Random, 95% CI
**1.4.2 71-75**						
Counsell 2007	−0.18817955	0.03555452	474	477	19.9%	0.83 [0.77, 0.89]
**Subtotal (95% CI)**			**474**	**477**	**19.9%**	**0.83 [0.77, 0.89]**
Heterogeneity: Not applicable						
Test for overall effect: Z = 5.29 (P < 0.00001)						
**1.4.3 76-80**						
van Haastregt 2000	0.3791478	0.34503278	120	115	3.1%	1.46 [0.74, 2.87]
Tinetti 1994	−0.39176342	0.26034166	147	144	4.9%	0.68 [0.41, 1.13]
Dalby 2000	−0.23794986	0.25436154	70	69	5.1%	0.79 [0.48, 1.30]
Stevens 2001	−0.02020271	0.20078915	635	1244	7.1%	0.98 [0.66, 1.45]
Bouman 2008	0	0.03541811	160	170	19.9%	1.00 [0.93, 1.07]
**Subtotal (95% CI)**			**1132**	**1742**	**40.1%**	**0.98 [0.88, 1.09]**
Heterogeneity: Tau² = 0.00; Chi² = 4.30, df = 4 (P = 0.37); I² = 7%						
Test for overall effect: Z = 0.36 (P = 0.72)						
**1.4.4 81-85**						
Robertson 2001a	−0.40578434	0.30662207	121	119	3.8%	0.67 [0.37, 1.22]
Campbell 2005	−0.10167807	0.25413251	98	96	5.1%	0.90 [0.55, 1.49]
Holland 2005	0.25147785	0.09945593	437	435	14.3%	1.29 [1.06, 1.56]
Caplan 2004	−0.2555538	0.07224321	370	369	16.8%	0.77 [0.67, 0.89]
**Subtotal (95% CI)**			**1026**	**1019**	**40.0%**	**0.91 [0.65, 1.28]**
Heterogeneity: Tau² = 0.09; Chi² = 18.10, df = 3 (P = 0.0004); I² = 83%						
Test for overall effect: Z = 0.53 (P = 0.59)						
**Total (95% CI)**			**2632**	**3238**	**100.0%**	**0.92 [0.81, 1.04]**
Heterogeneity: Tau² = 0.02; Chi² = 35.81, df = 9 (P < 0.0001); I² = 75%						
Test for overall effect: Z = 1.31 (P = 0.19)						
Test for subgroup differences: Chi² = 6.61, df = 2 (P = 0.04), I² = 69.7%						

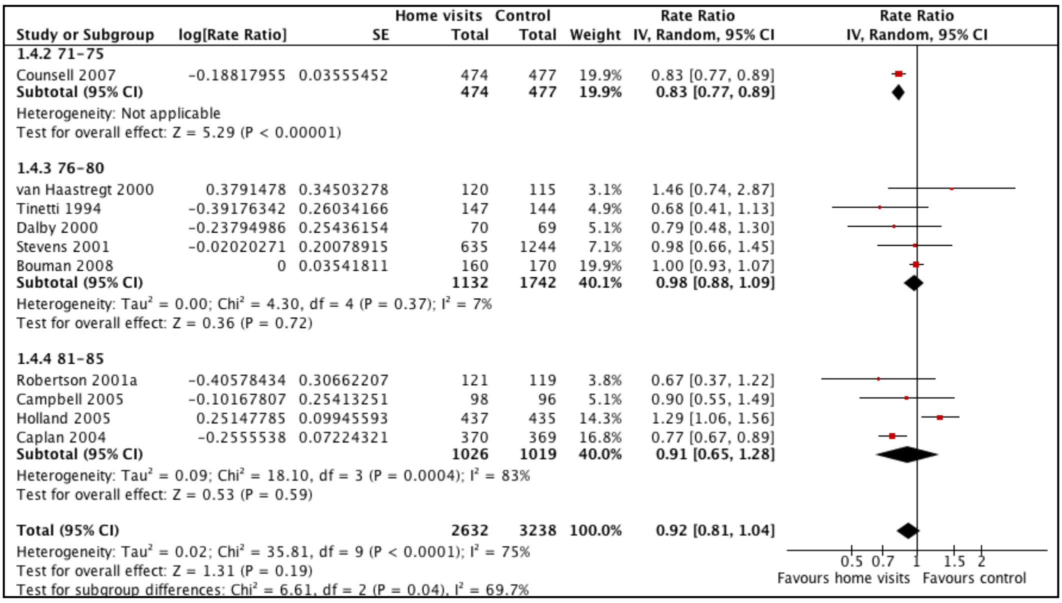

Analysis 5: ER (number); type of visitor subgroups

Study or Subgroup	log[Rate Ratio]	SE	Home visits Total	Control Total	Weight	Rate Ratio IV, Random, 95% CI
**1.5.1 Nurse**						
van Haastregt 2000	0.3791478	0.34503278	120	115	3.1%	1.46 [0.74, 2.87]
Robertson 2001a	−0.40578434	0.30662207	121	119	3.8%	0.67 [0.37, 1.22]
Dalby 2000	−0.23794986	0.25436154	70	69	5.1%	0.79 [0.48, 1.30]
Stevens 2001	−0.02020271	0.20078915	635	1244	7.1%	0.98 [0.66, 1.45]
Caplan 2004	−0.2555538	0.07224321	370	369	16.8%	0.77 [0.67, 0.89]
Bouman 2008	0	0.03541811	160	170	19.9%	1.00 [0.93, 1.07]
**Subtotal (95% CI)**			**1476**	**2086**	**55.8%**	**0.90 [0.75, 1.07]**
Heterogeneity: Tau² = 0.02; Chi² = 13.55, df = 5 (P = 0.02); I² = 63%						
Test for overall effect: Z = 1.21 (P = 0.23)						
**1.5.2 Other**						
Holland 2005	0.25147785	0.09945593	437	435	14.3%	1.29 [1.06, 1.56]
**Subtotal (95% CI)**			**437**	**435**	**14.3%**	**1.29 [1.06, 1.56]**
Heterogeneity: Not applicable						
Test for overall effect: Z = 2.53 (P = 0.01)						
**1.5.3 Combined**						
Tinetti 1994	−0.39176342	0.26034166	147	144	4.9%	0.68 [0.41, 1.13]
Campbell 2005	−0.10167807	0.25413251	98	96	5.1%	0.90 [0.55, 1.49]
Counsell 2007	−0.18817955	0.03555452	474	477	19.9%	0.83 [0.77, 0.89]
**Subtotal (95% CI)**			**719**	**717**	**29.9%**	**0.83 [0.77, 0.89]**
Heterogeneity: Tau² = 0.00; Chi² = 0.72, df = 2 (P = 0.70); I² = 0%						
Test for overall effect: Z = 5.45 (P < 0.00001)						
**Total (95% CI)**			**2632**	**3238**	**100.0%**	**0.92 [0.81, 1.04]**
Heterogeneity: Tau² = 0.02; Chi² = 35.81, df = 9 (P < 0.0001); I² = 75%						
Test for overall effect: Z = 1.31 (P = 0.19)						
Test for subgroup differences: Chi² = 17.69, df = 2 (P = 0.0001), I² = 88.7%						

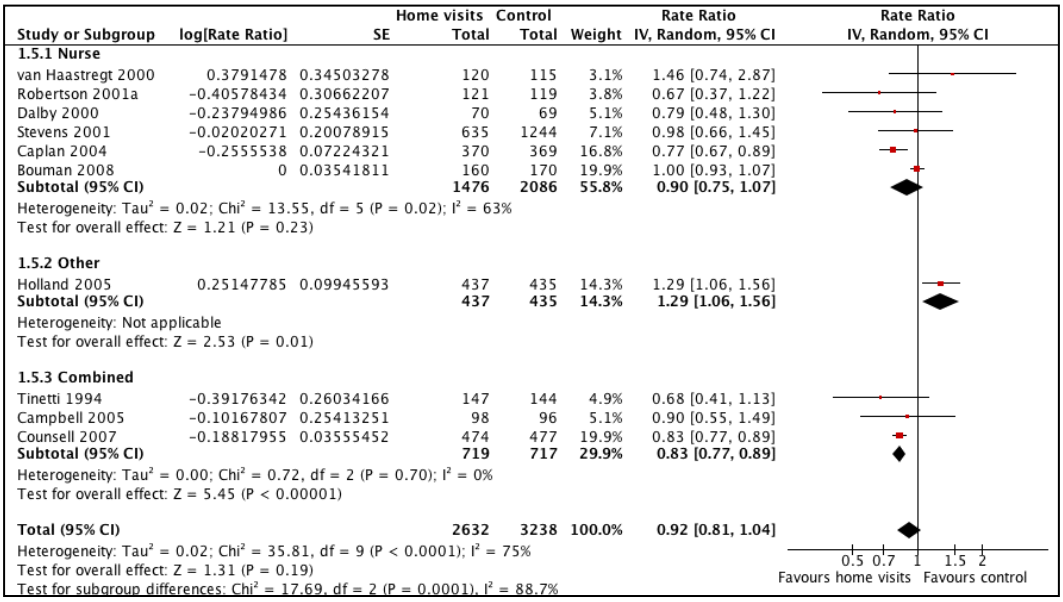

Analysis 6: ER (number); number of visits subgroups

Study or Subgroup	log[Rate Ratio]	SE	Home visits Total	Control Total	Weight	Rate Ratio IV, Random, 95% CI
**1.6.1 1 Visit**						
Stevens 2001	−0.02020271	0.20078915	635	1244	7.1%	0.98 [0.66, 1.45]
**Subtotal (95% CI)**			**635**	**1244**	**7.1%**	**0.98 [0.66, 1.45]**
Heterogeneity: Not applicable						
Test for overall effect: Z = 0.10 (P = 0.92)						
**1.6.2 2-4 Visits**						
Dalby 2000	−0.23794986	0.25436154	70	69	5.1%	0.79 [0.48, 1.30]
Holland 2005	0.25147785	0.09945593	437	435	14.3%	1.29 [1.06, 1.56]
Caplan 2004	−0.2555538	0.07224321	370	369	16.8%	0.77 [0.67, 0.89]
**Subtotal (95% CI)**			**877**	**873**	**36.2%**	**0.94 [0.63, 1.39]**
Heterogeneity: Tau² = 0.10; Chi² = 17.38, df = 2 (P = 0.0002); I² = 88%						
Test for overall effect: Z = 0.32 (P = 0.75)						
**1.6.3 5 or More Visits**						
van Haastregt 2000	0.3791478	0.34503278	120	115	3.1%	1.46 [0.74, 2.87]
Robertson 2001a	−0.40578434	0.30662207	121	119	3.8%	0.67 [0.37, 1.22]
Tinetti 1994	−0.39176342	0.26034166	147	144	4.9%	0.68 [0.41, 1.13]
Campbell 2005	−0.10167807	0.25413251	98	96	5.1%	0.90 [0.55, 1.49]
Counsell 2007	−0.18817955	0.03555452	474	477	19.9%	0.83 [0.77, 0.89]
Bouman 2008	0	0.03541811	160	170	19.9%	1.00 [0.93, 1.07]
**Subtotal (95% CI)**			**1120**	**1121**	**56.7%**	**0.89 [0.77, 1.04]**
Heterogeneity: Tau² = 0.02; Chi² = 18.28, df = 5 (P = 0.003); I² = 73%						
Test for overall effect: Z = 1.43 (P = 0.15)						
**Total (95% CI)**			**2632**	**3238**	**100.0%**	**0.92 [0.81, 1.04]**
Heterogeneity: Tau² = 0.02; Chi² = 35.81, df = 9 (P < 0.0001); I² = 75%						
Test for overall effect: Z = 1.31 (P = 0.19)						
Test for subgroup differences: Chi² = 0.21, df = 2 (P = 0.90), I² = 0%						

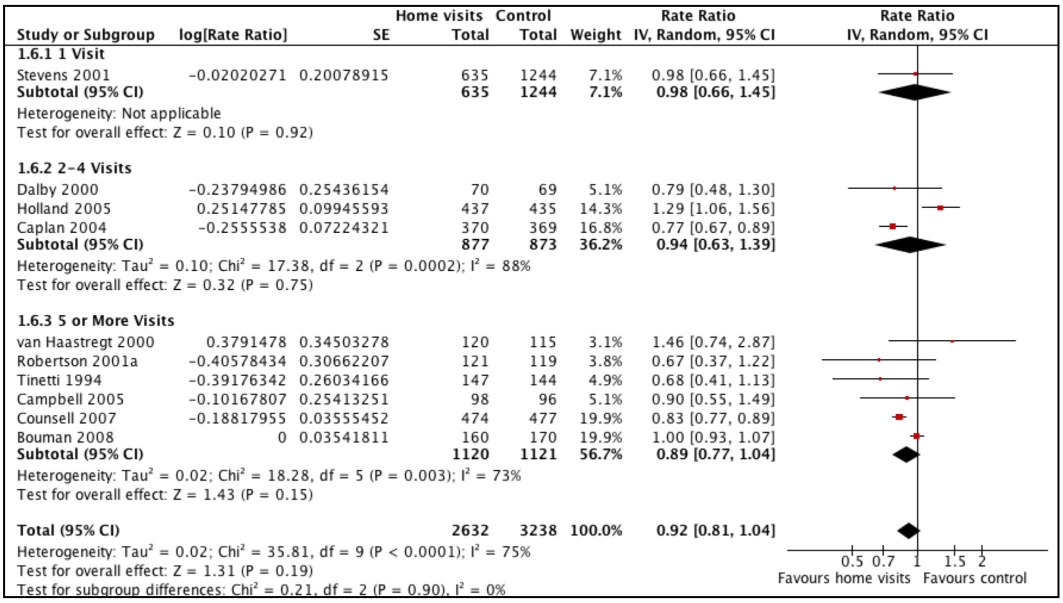

Analysis 7: ER (people); results at longest follow-up

Study or Subgroup	log[Risk Ratio]	SE	Home visits Total	Control Total	Weight	Risk Ratio IV, Random, 95% CI
Hogan 2001	0.17915198	0.45994361	79	84	1.8%	1.20 [0.49, 2.95]
Bernabei 1998	−1.03140354	0.45319884	99	100	1.8%	0.36 [0.15, 0.87]
Vetter 1992	0	0.35355339	240	210	2.9%	1.00 [0.50, 2.00]
Ciechanowski 2004	−0.36480123	0.27139692	69	62	4.6%	0.69 [0.41, 1.18]
Tinetti 1994	−0.23419339	0.26894873	147	144	4.7%	0.79 [0.47, 1.34]
Davison 2005	−0.10891264	0.25348417	159	154	5.2%	0.90 [0.55, 1.47]
Sommers 2000	0.33216897	0.23700287	238	227	5.8%	1.39 [0.88, 2.22]
Luukinen 2006	−0.00048004	0.1715891	133	142	9.4%	1.00 [0.71, 1.40]
Lightbody 2002	−0.27376381	0.16660214	155	159	9.8%	0.76 [0.55, 1.05]
van Hout 2010	0.11724288	0.11861371	331	320	14.7%	1.12 [0.89, 1.42]
Bouman 2008	−0.04585886	0.09594067	160	170	17.9%	0.96 [0.79, 1.15]
Caplan 2004	−0.20614484	0.07523268	370	369	21.3%	0.81 [0.70, 0.94]
**Total (95% CI)**			**2180**	**2141**	**100.0%**	**0.91 [0.81, 1.03]**
Heterogeneity: Tau² = 0.01; Chi² = 16.29, df = 11 (P = 0.13); I² = 32%						
Test for overall effect: Z = 1.45 (P = 0.15)						

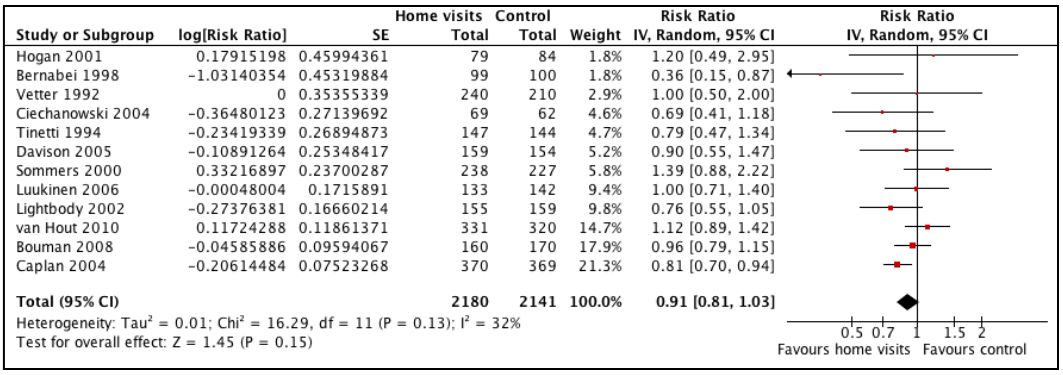

Analysis 8: ER (people); at each follow-up interval

Study or Subgroup	log[Risk Ratio]	SE	Home visits Total	Control Total	Weight	Risk Ratio IV, Random, 95% CI
**1.8.1 01-11 months**						
Ciechanowski 2004	−0.36480123	0.27139692	69	62	17.9%	0.69 [0.41, 1.18]
Caplan 2004	−0.19686238	0.19494778	370	369	34.7%	0.82 [0.56, 1.20]
Lightbody 2002	−0.27376381	0.16660214	155	159	47.5%	0.76 [0.55, 1.05]
**Subtotal (95% CI)**			**594**	**590**	**100.0%**	**0.77 [0.61, 0.96]**
Heterogeneity: Tau² = 0.00; Chi² = 0.26, df = 2 (P = 0.88); I² = 0%						
Test for overall effect: Z = 2.29 (P = 0.02)						
**1.8.2 12-23 months**						
Hogan 2001	0.17915198	0.45994361	79	84	2.4%	1.20 [0.49, 2.95]
Bernabei 1998	−1.03140354	0.45319884	99	100	2.4%	0.36 [0.15, 0.87]
Tinetti 1994	−0.23419339	0.26894873	147	144	6.1%	0.79 [0.47, 1.34]
Sommers 2000	0.19109037	0.26222999	238	227	6.4%	1.21 [0.72, 2.02]
Davison 2005	−0.10891264	0.25348417	159	154	6.7%	0.90 [0.55, 1.47]
Luukinen 2006	−0.00048004	0.1715891	133	142	11.9%	1.00 [0.71, 1.40]
van Hout 2010	0.11724288	0.11861371	331	320	18.0%	1.12 [0.89, 1.42]
Bouman 2008	−0.23947997	0.0981168	160	170	21.1%	0.79 [0.65, 0.95]
Caplan 2004	−0.20614484	0.07523268	370	369	25.0%	0.81 [0.70, 0.94]
**Subtotal (95% CI)**			**1716**	**1710**	**100.0%**	**0.89 [0.77, 1.03]**
Heterogeneity: Tau² = 0.02; Chi² = 13.24, df = 8 (P = 0.10); I² = 40%						
Test for overall effect: Z = 1.52 (P = 0.13)						
**1.8.3 24-35 months**						
Sommers 2000	0.33216897	0.23700287	238	227	33.6%	1.39 [0.88, 2.22]
Bouman 2008	−0.04585886	0.09594067	160	170	66.4%	0.96 [0.79, 1.15]
**Subtotal (95% CI)**			**398**	**397**	**100.0%**	**1.08 [0.76, 1.54]**
Heterogeneity: Tau² = 0.04; Chi² = 2.19, df = 1 (P = 0.14); I² = 54%						
Test for overall effect: Z = 0.45 (P = 0.65)						
**1.8.4 36+ months**						
Vetter 1992	0	0.35355339	240	210	100.0%	1.00 [0.50, 2.00]
**Subtotal (95% CI)**			**240**	**210**	**100.0%**	**1.00 [0.50, 2.00]**
Heterogeneity: Not applicable						
Test for overall effect: Z = 0.00 (P = 1.00)						

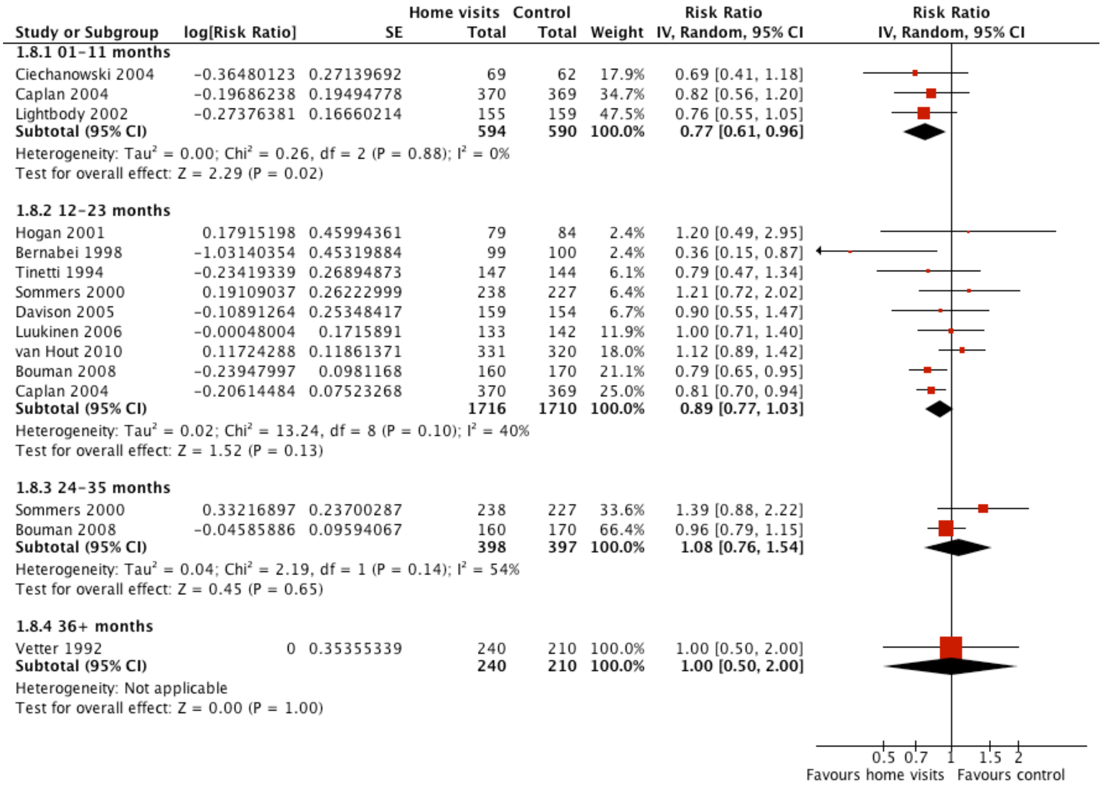

Analysis 9: ER (people); focus of visit subgroups

Study or Subgroup	log[Risk Ratio]	SE	Home visits Total	Control Total	Weight	Risk Ratio IV, Random, 95% CI
**1.9.1 Falls Only**						
Hogan 2001	0.17915198	0.45994361	79	84	1.8%	1.20 [0.49, 2.95]
Luukinen 2006	−0.00048004	0.1715891	133	142	9.4%	1.00 [0.71, 1.40]
**Subtotal (95% CI)**			**212**	**226**	**11.2%**	**1.02 [0.75, 1.40]**
Heterogeneity: Tau² = 0.00; Chi² = 0.13, df = 1 (P = 0.71); I² = 0%						
Test for overall effect: Z = 0.13 (P = 0.89)						
**1.9.2 MGA Only**						
Bernabei 1998	−1.03140354	0.45319884	99	100	1.8%	0.36 [0.15, 0.87]
Sommers 2000	0.33216897	0.23700287	238	227	5.8%	1.39 [0.88, 2.22]
Bouman 2008	−0.04585886	0.09594067	160	170	17.9%	0.96 [0.79, 1.15]
Caplan 2004	−0.20614484	0.07523268	370	369	21.3%	0.81 [0.70, 0.94]
**Subtotal (95% CI)**			**867**	**866**	**46.9%**	**0.90 [0.69, 1.16]**
Heterogeneity: Tau² = 0.04; Chi² = 9.55, df = 3 (P = 0.02); I² = 69%						
Test for overall effect: Z = 0.83 (P = 0.41)						
**1.9.3 Both**						
Vetter 1992	0	0.35355339	240	210	2.9%	1.00 [0.50, 2.00]
Tinetti 1994	−0.23419339	0.26894873	147	144	4.7%	0.79 [0.47, 1.34]
Davison 2005	−0.10891264	0.25348417	159	154	5.2%	0.90 [0.55, 1.47]
Lightbody 2002	−0.27376381	0.16660214	155	159	9.8%	0.76 [0.55, 1.05]
van Hout 2010	0.11724288	0.11861371	331	320	14.7%	1.12 [0.89, 1.42]
**Subtotal (95% CI)**			**1032**	**987**	**37.3%**	**0.95 [0.79, 1.13]**
Heterogeneity: Tau² = 0.00; Chi² = 4.33, df = 4 (P = 0.36); I² = 8%						
Test for overall effect: Z = 0.61 (P = 0.54)						
**1.9.4 Neither**						
Ciechanowski 2004	−0.36480123	0.27139692	69	62	4.6%	0.69 [0.41, 1.18]
**Subtotal (95% CI)**			**69**	**62**	**4.6%**	**0.69 [0.41, 1.18]**
Heterogeneity: Not applicable						
Test for overall effect: Z = 1.34 (P = 0.18)						
**Total (95% CI)**			**2180**	**2141**	**100.0%**	**0.91 [0.81, 1.03]**
Heterogeneity: Tau² = 0.01; Chi² = 16.29, df = 11 (P = 0.13); I² = 32%						
Test for overall effect: Z = 1.45 (P = 0.15)						
Test for subgroup differences: Chi² = 1.62, df = 3 (P = 0.65), I² = 0%						

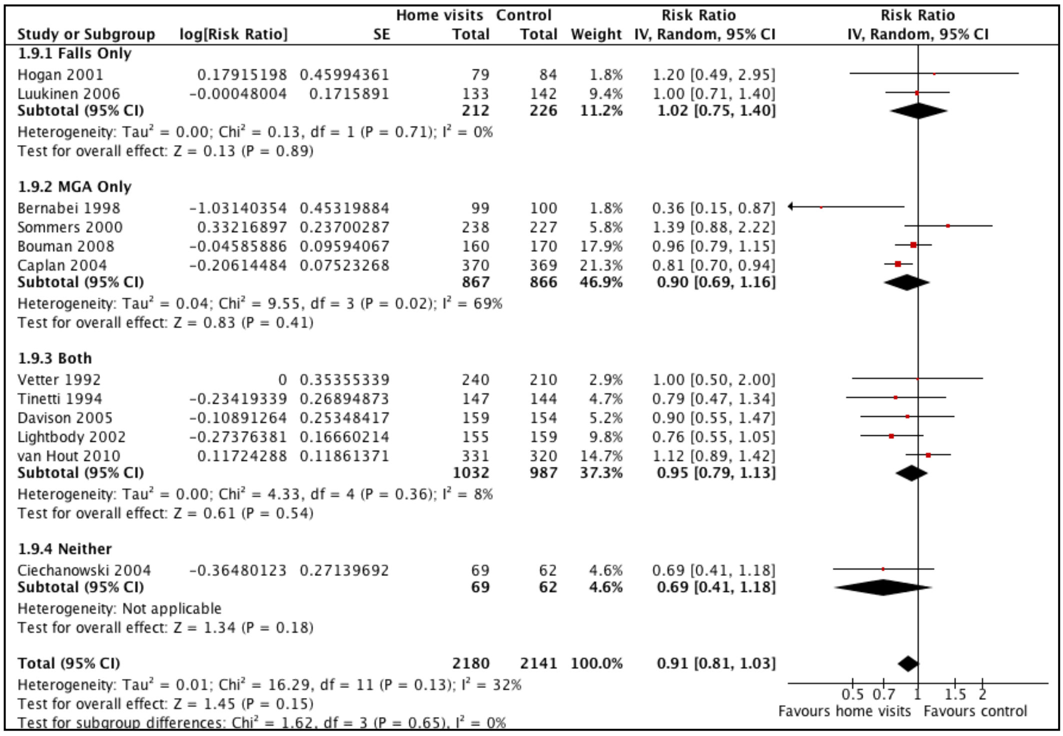

Analysis 10: ER (people); age of participants subgroups

Study or Subgroup	log[Risk Ratio]	SE	Home visits Total	Control Total	Weight	Risk Ratio IV, Random, 95% CI
**1.10.2 71-75**						
Ciechanowski 2004	−0.36480123	0.27139692	69	62	4.6%	0.69 [0.41, 1.18]
Lightbody 2002	−0.27376381	0.16660214	155	159	9.8%	0.76 [0.55, 1.05]
**Subtotal (95% CI)**			**224**	**221**	**14.4%**	**0.74 [0.56, 0.98]**
Heterogeneity: Tau² = 0.00; Chi² = 0.08, df = 1 (P = 0.77); I² = 0%						
Test for overall effect: Z = 2.10 (P = 0.04)						
**1.10.3 76-80**						
Hogan 2001	0.17915198	0.45994361	79	84	1.8%	1.20 [0.49, 2.95]
Vetter 1992	0	0.35355339	240	210	2.9%	1.00 [0.50, 2.00]
Tinetti 1994	−0.23419339	0.26894873	147	144	4.7%	0.79 [0.47, 1.34]
Davison 2005	−0.10891264	0.25348417	159	154	5.2%	0.90 [0.55, 1.47]
Sommers 2000	0.33216897	0.23700287	238	227	5.8%	1.39 [0.88, 2.22]
Bouman 2008	−0.04585886	0.09594067	160	170	17.9%	0.96 [0.79, 1.15]
**Subtotal (95% CI)**			**1023**	**989**	**38.3%**	**0.98 [0.84, 1.14]**
Heterogeneity: Tau² = 0.00; Chi² = 3.23, df = 5 (P = 0.66); I² = 0%						
Test for overall effect: Z = 0.24 (P = 0.81)						
**1.10.4 81-85**						
Bernabei 1998	−1.03140354	0.45319884	99	100	1.8%	0.36 [0.15, 0.87]
van Hout 2010	0.11724288	0.11861371	331	320	14.7%	1.12 [0.89, 1.42]
Caplan 2004	−0.20614484	0.07523268	370	369	21.3%	0.81 [0.70, 0.94]
**Subtotal (95% CI)**			**800**	**789**	**37.9%**	**0.84 [0.59, 1.20]**
Heterogeneity: Tau² = 0.07; Chi² = 9.33, df = 2 (P = 0.009); I² = 79%						
Test for overall effect: Z = 0.95 (P = 0.34)						
**1.10.5 86+**						
Luukinen 2006	−0.00048004	0.1715891	133	142	9.4%	1.00 [0.71, 1.40]
**Subtotal (95% CI)**			**133**	**142**	**9.4%**	**1.00 [0.71, 1.40]**
Heterogeneity: Not applicable						
Test for overall effect: Z = 0.00 (P = 1.00)						
**Total (95% CI)**			**2180**	**2141**	**100.0%**	**0.91 [0.81, 1.03]**
Heterogeneity: Tau² = 0.01; Chi² = 16.29, df = 11 (P = 0.13); I² = 32%						
Test for overall effect: Z = 1.45 (P = 0.15)						
Test for subgroup differences: Chi² = 3.48, df = 3 (P = 0.32), I² = 13.8%						

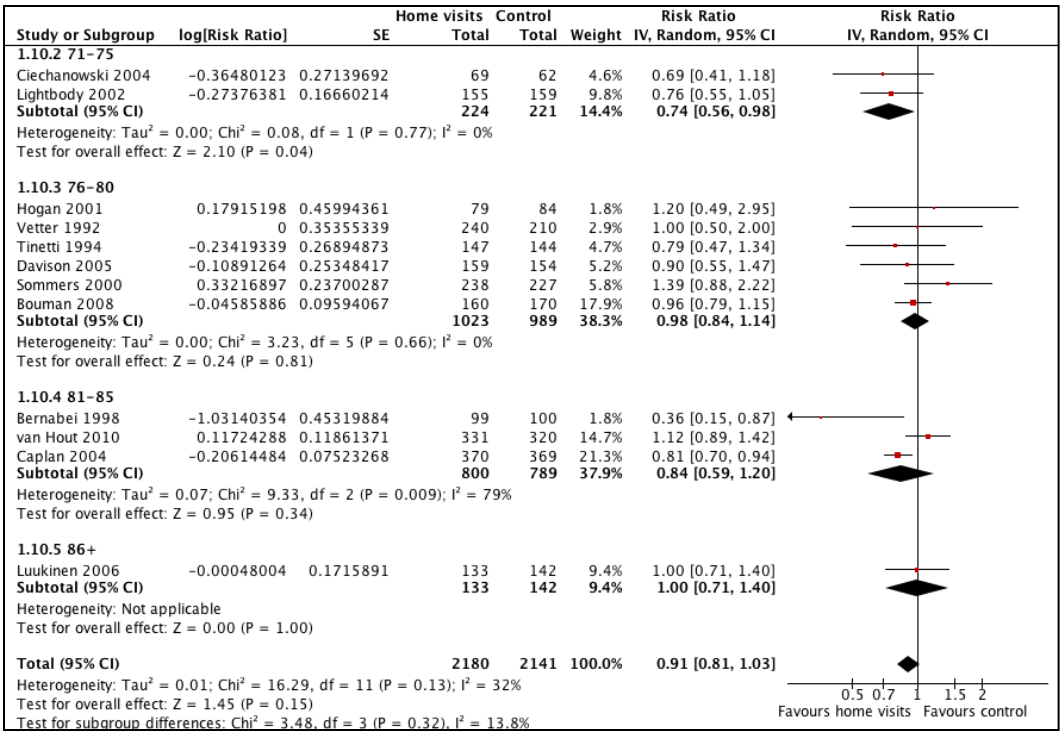

Analysis 11: ER (people); type of visitor subgroups

Study or Subgroup	log[Risk Ratio]	SE	Home visits Total	Control Total	Weight	Risk Ratio IV, Random, 95% CI
**1.11.1 Nurse**						
Lightbody 2002	−0.27376381	0.16660214	155	159	9.8%	0.76 [0.55, 1.05]
van Hout 2010	0.11724288	0.11861371	331	320	14.7%	1.12 [0.89, 1.42]
Bouman 2008	−0.04585886	0.09594067	160	170	17.9%	0.96 [0.79, 1.15]
Caplan 2004	−0.20614484	0.07523268	370	369	21.3%	0.81 [0.70, 0.94]
**Subtotal (95% CI)**			**1016**	**1018**	**63.8%**	**0.91 [0.78, 1.06]**
Heterogeneity: Tau² = 0.01; Chi² = 6.72, df = 3 (P = 0.08); I² = 55%						
Test for overall effect: Z = 1.20 (P = 0.23)						
**1.11.2 Other**						
Bernabei 1998	−1.03140354	0.45319884	99	100	1.8%	0.36 [0.15, 0.87]
Vetter 1992	0	0.35355339	240	210	2.9%	1.00 [0.50, 2.00]
Ciechanowski 2004	−0.36480123	0.27139692	69	62	4.6%	0.69 [0.41, 1.18]
**Subtotal (95% CI)**			**408**	**372**	**9.4%**	**0.67 [0.41, 1.11]**
Heterogeneity: Tau² = 0.08; Chi² = 3.22, df = 2 (P = 0.20); I² = 38%						
Test for overall effect: Z = 1.57 (P = 0.12)						
**1.11.3 Combined**						
Hogan 2001	0.17915198	0.45994361	79	84	1.8%	1.20 [0.49, 2.95]
Tinetti 1994	−0.23419339	0.26894873	147	144	4.7%	0.79 [0.47, 1.34]
Davison 2005	−0.10891264	0.25348417	159	154	5.2%	0.90 [0.55, 1.47]
Sommers 2000	0.33216897	0.23700287	238	227	5.8%	1.39 [0.88, 2.22]
Luukinen 2006	−0.00048004	0.1715891	133	142	9.4%	1.00 [0.71, 1.40]
**Subtotal (95% CI)**			**756**	**751**	**26.8%**	**1.02 [0.83, 1.26]**
Heterogeneity: Tau² = 0.00; Chi² = 3.02, df = 4 (P = 0.55); I² = 0%						
Test for overall effect: Z = 0.20 (P = 0.85)						
**Total (95% CI)**			**2180**	**2141**	**100.0%**	**0.91 [0.81, 1.03]**
Heterogeneity: Tau² = 0.01; Chi² = 16.29, df = 11 (P = 0.13); I² = 32%						
Test for overall effect: Z = 1.45 (P = 0.15)						
Test for subgroup differences: Chi² = 2.49, df = 2 (P = 0.29), I² = 19.5%						

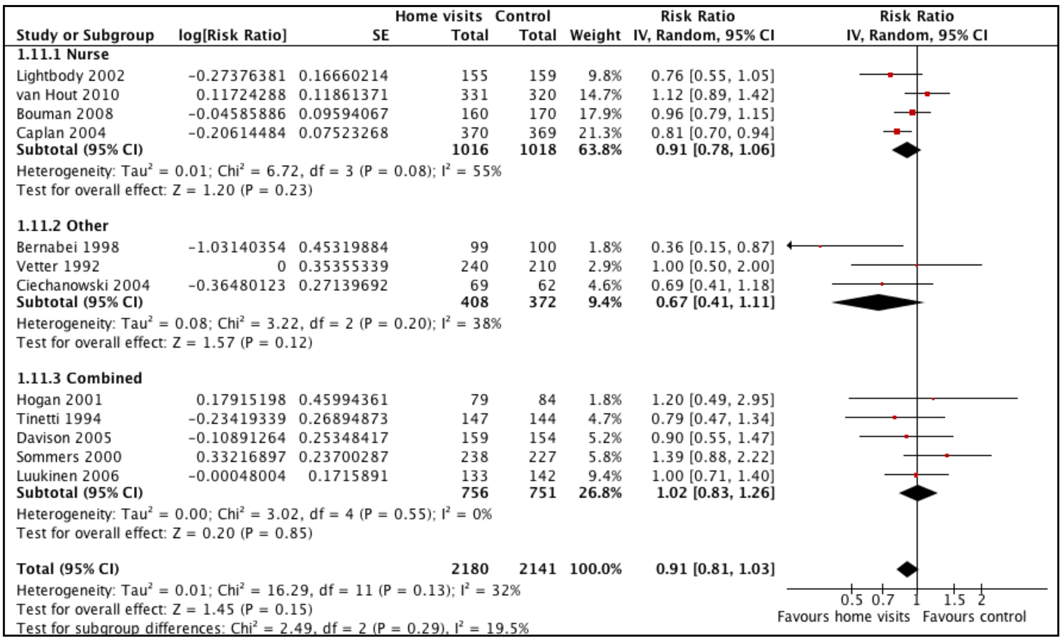

Analysis 12: ER (people); number of visits subgroups

Study or Subgroup	log[Risk Ratio]	SE	Home visits Total	Control Total	Weight	Risk Ratio IV, Random, 95% CI
**1.12.1 1 Visit**						
Lightbody 2002	−0.27376381	0.16660214	155	159	10.6%	0.76 [0.55, 1.05]
**Subtotal (95% CI)**			**155**	**159**	**10.6%**	**0.76 [0.55, 1.05]**
Heterogeneity: Not applicable						
Test for overall effect: Z = 1.64 (P = 0.10)						
**1.12.2 2-4 Visits**						
Hogan 2001	0.17915198	0.45994361	79	84	2.0%	1.20 [0.49, 2.95]
Vetter 1992	0	0.35355339	240	210	3.3%	1.00 [0.50, 2.00]
Luukinen 2006	−0.00048004	0.1715891	133	142	10.2%	1.00 [0.71, 1.40]
van Hout 2010	0.11724288	0.11861371	331	320	15.4%	1.12 [0.89, 1.42]
Caplan 2004	−0.20614484	0.07523268	370	369	21.2%	0.81 [0.70, 0.94]
**Subtotal (95% CI)**			**1153**	**1125**	**52.1%**	**0.95 [0.80, 1.13]**
Heterogeneity: $Tau^2 = 0.01$; $Chi^2 = 6.11$, df = 4 (P = 0.19); $I^2 = 35\%$						
Test for overall effect: Z = 0.54 (P = 0.59)						
**1.12.3 5 or More Visits**						
Bernabei 1998	−1.03140354	0.45319884	99	100	2.1%	0.36 [0.15, 0.87]
Ciechanowski 2004	−0.36480123	0.27139692	69	62	5.2%	0.69 [0.41, 1.18]
Tinetti 1994	−0.23419339	0.26894873	147	144	5.3%	0.79 [0.47, 1.34]
Sommers 2000	0.33216897	0.23700287	238	227	6.4%	1.39 [0.88, 2.22]
Bouman 2008	−0.04585886	0.09594067	160	170	18.3%	0.96 [0.79, 1.15]
**Subtotal (95% CI)**			**713**	**703**	**37.3%**	**0.86 [0.63, 1.17]**
Heterogeneity: $Tau^2 = 0.06$; $Chi^2 = 8.99$, df = 4 (P = 0.06); $I^2 = 55\%$						
Test for overall effect: Z = 0.96 (P = 0.34)						
**Total (95% CI)**			**2021**	**1987**	**100.0%**	**0.91 [0.80, 1.04]**
Heterogeneity: $Tau^2 = 0.02$; $Chi^2 = 16.29$, df = 10 (P = 0.09); $I^2 = 39\%$						
Test for overall effect: Z = 1.33 (P = 0.18)						
Test for subgroup differences: $Chi^2 = 1.52$, df = 2 (P = 0.47), $I^2 = 0\%$						

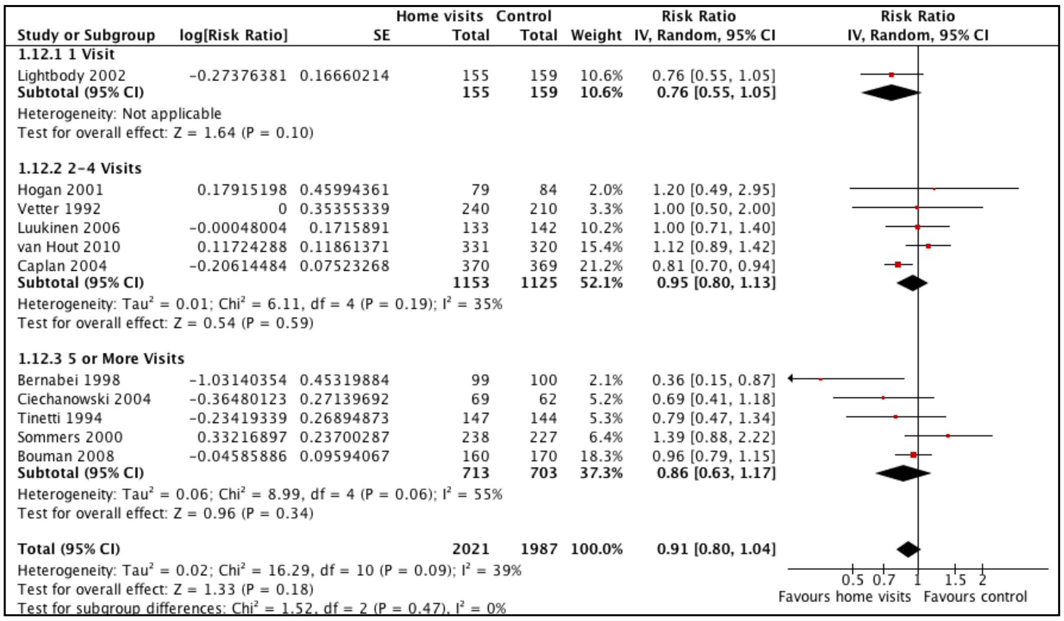

Analysis 13: Falls (number); results at longest follow-up

Study or Subgroup	log[Rate Ratio]	SE	Home visits Total	Control Total	Weight	Rate Ratio IV, Random, 95% CI
Gallagher 1996	−0.21130909	0.20634202	50	50	5.0%	0.81 [0.54, 1.21]
Markle-Reid 2010	0.22910735	0.17784366	54	55	5.6%	1.26 [0.89, 1.78]
Robertson 2001a	−0.34759773	0.14720418	121	119	6.1%	0.71 [0.53, 0.94]
Wyman 2007	−0.36301284	0.14250657	137	135	6.2%	0.70 [0.53, 0.92]
Tinetti 1994	−0.58369195	0.12936715	153	148	6.5%	0.56 [0.43, 0.72]
Campbell 2005	−0.34377154	0.12431871	98	96	6.6%	0.71 [0.56, 0.90]
Stevens 2001	0.05501813	0.12115499	635	1244	6.6%	1.06 [0.83, 1.34]
Lightbody 2002	−0.18016464	0.11375479	155	159	6.7%	0.84 [0.67, 1.04]
Pighills 2011	−0.6428122	0.09572127	87	78	7.1%	0.53 [0.44, 0.63]
Campbell 1999	−0.36028289	0.09479566	116	117	7.1%	0.70 [0.58, 0.84]
Close 1999	−0.88566251	0.08616998	184	213	7.2%	0.41 [0.35, 0.49]
Hogan 2001	−0.21110179	0.08581848	75	77	7.2%	0.81 [0.68, 0.96]
Elley 2008	−0.0251149	0.08278438	155	157	7.3%	0.98 [0.83, 1.15]
Luukinen 2006	−0.06725223	0.07599346	180	178	7.4%	0.93 [0.81, 1.09]
Davison 2005	−0.50528416	0.06484385	144	149	7.5%	0.60 [0.53, 0.69]
**Total (95% CI)**			**2344**	**2975**	**100.0%**	**0.74 [0.63, 0.86]**
Heterogeneity: $Tau^2 = 0.07$; $Chi^2 = 113.04$, df = 14 (P < 0.00001); $I^2 = 88\%$						
Test for overall effect: Z = 3.98 (P < 0.0001)						

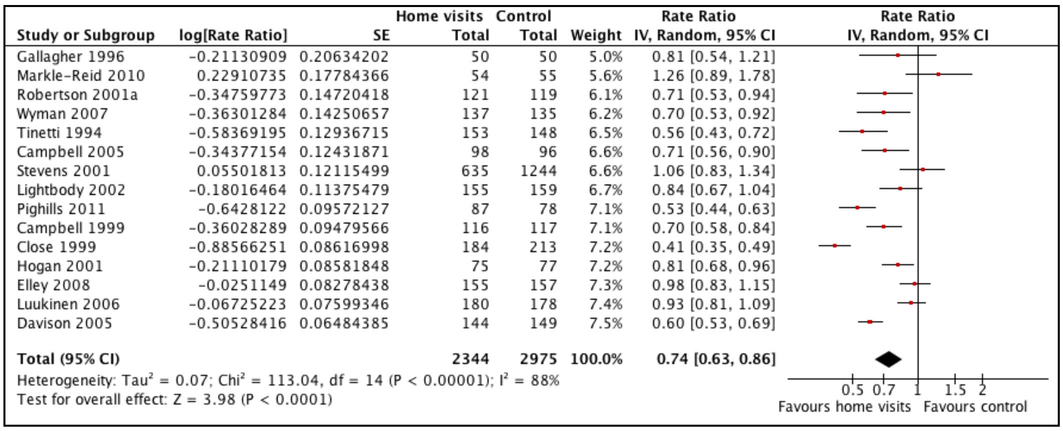

Analysis 14: Falls (number); at each follow-up interval

Study or Subgroup	log[Rate Ratio]	SE	Home visits Total	Control Total	Weight	Rate Ratio IV, Random, 95% CI
**1.14.1 01-11 months**						
Gallagher 1996	−0.21130909	0.20634202	50	50	25.7%	0.81 [0.54, 1.21]
Markle-Reid 2010	0.22910735	0.17784366	54	55	30.4%	1.26 [0.89, 1.78]
Lightbody 2002	−0.18016464	0.11375479	155	159	43.9%	0.84 [0.67, 1.04]
**Subtotal (95% CI)**			**259**	**264**	**100.0%**	**0.94 [0.72, 1.22]**
Heterogeneity: Tau² = 0.03; Chi² = 4.19, df = 2 (P = 0.12); I² = 52%						
Test for overall effect: Z = 0.47 (P = 0.64)						
**1.14.2 12-23 months**						
Robertson 2001a	−0.34759773	0.14720418	121	119	7.5%	0.71 [0.53, 0.94]
Wyman 2007	−0.36301284	0.14250657	137	135	7.6%	0.70 [0.53, 0.92]
Campbell 1999	−0.50339065	0.13124277	116	117	7.9%	0.60 [0.47, 0.78]
Tinetti 1994	−0.58369195	0.12936715	153	148	7.9%	0.56 [0.43, 0.72]
Campbell 2005	−0.34377154	0.12431871	98	96	8.0%	0.71 [0.56, 0.90]
Stevens 2001	0.05501813	0.12115499	635	1244	8.1%	1.06 [0.83, 1.34]
Pighills 2011	−0.6428122	0.09572127	87	78	8.6%	0.53 [0.44, 0.63]
Close 1999	−0.88566251	0.08616998	184	213	8.8%	0.41 [0.35, 0.49]
Hogan 2001	−0.21110179	0.08581848	75	77	8.8%	0.81 [0.68, 0.96]
Elley 2008	−0.0251149	0.08278438	155	157	8.8%	0.98 [0.83, 1.15]
Luukinen 2006	−0.06725223	0.07599346	180	178	8.9%	0.93 [0.81, 1.09]
Davison 2005	−0.50528416	0.06484385	144	149	9.1%	0.60 [0.53, 0.69]
**Subtotal (95% CI)**			**2085**	**2711**	**100.0%**	**0.69 [0.58, 0.82]**
Heterogeneity: Tau² = 0.08; Chi² = 101.06, df = 11 (P < 0.00001); I² = 89%						
Test for overall effect: Z = 4.24 (P < 0.0001)						
**1.14.3 24-35 months**						
Campbell 1999	−0.36028289	0.09479566	116	117	100.0%	0.70 [0.58, 0.84]
**Subtotal (95% CI)**			**116**	**117**	**100.0%**	**0.70 [0.58, 0.84]**
Heterogeneity: Not applicable						
Test for overall effect: Z = 3.80 (P = 0.0001)						

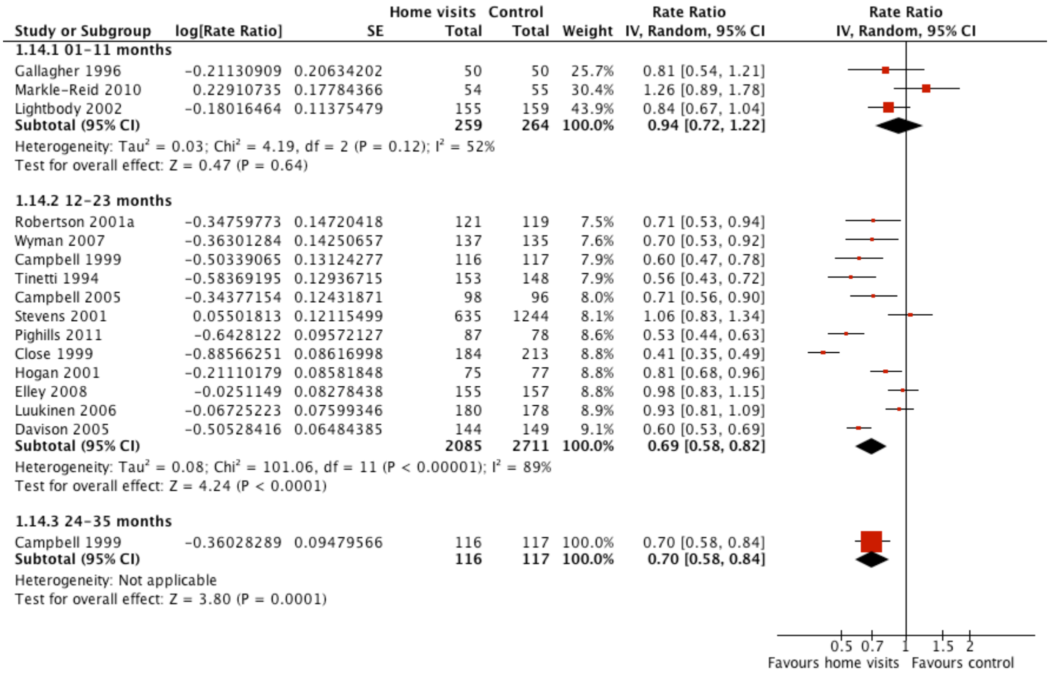

Analysis 15: Falls (number); focus of visit subgroups

Study or Subgroup	log[Rate Ratio]	SE	Home visits Total	Control Total	Weight	Rate Ratio IV, Random, 95% CI
**1.15.1 Falls Only**						
Gallagher 1996	−0.21130909	0.20634202	50	50	5.0%	0.81 [0.54, 1.21]
Robertson 2001a	−0.34759773	0.14720418	121	119	6.1%	0.71 [0.53, 0.94]
Wyman 2007	−0.36301284	0.14250657	137	135	6.2%	0.70 [0.53, 0.92]
Campbell 2005	−0.34377154	0.12431871	98	96	6.6%	0.71 [0.56, 0.90]
Stevens 2001	0.05501813	0.12115499	635	1244	6.6%	1.06 [0.83, 1.34]
Pighills 2011	−0.6428122	0.09572127	87	78	7.1%	0.53 [0.44, 0.63]
Campbell 1999	−0.36028289	0.09479566	116	117	7.1%	0.70 [0.58, 0.84]
Hogan 2001	−0.21110179	0.08581848	75	77	7.2%	0.81 [0.68, 0.96]
Elley 2008	−0.0251149	0.08278438	155	157	7.3%	0.98 [0.83, 1.15]
Luukinen 2006	−0.06725223	0.07599346	180	178	7.4%	0.93 [0.81, 1.09]
**Subtotal (95% CI)**			**1654**	**2251**	**66.5%**	**0.78 [0.68, 0.90]**
Heterogeneity: Tau² = 0.04; Chi² = 39.13, df = 9 (P < 0.0001); I² = 77%						
Test for overall effect: Z = 3.48 (P = 0.0005)						
**1.15.3 Both**						
Markle-Reid 2010	0.22910735	0.17784366	54	55	5.6%	1.26 [0.89, 1.78]
Tinetti 1994	−0.58369195	0.12936715	153	148	6.5%	0.56 [0.43, 0.72]
Lightbody 2002	−0.18016464	0.11375479	155	159	6.7%	0.84 [0.67, 1.04]
Close 1999	−0.88566251	0.08616998	184	213	7.2%	0.41 [0.35, 0.49]
Davison 2005	−0.50528416	0.06484385	144	149	7.5%	0.60 [0.53, 0.69]
**Subtotal (95% CI)**			**690**	**724**	**33.5%**	**0.67 [0.49, 0.90]**
Heterogeneity: Tau² = 0.11; Chi² = 44.97, df = 4 (P < 0.00001); I² = 91%						
Test for overall effect: Z = 2.62 (P = 0.009)						
**Total (95% CI)**			**2344**	**2975**	**100.0%**	**0.74 [0.63, 0.86]**
Heterogeneity: Tau² = 0.07; Chi² = 113.04, df = 14 (P < 0.00001); I² = 88%						
Test for overall effect: Z = 3.98 (P < 0.0001)						
Test for subgroup differences: Chi² = 0.87, df = 1 (P = 0.35), I² = 0%						

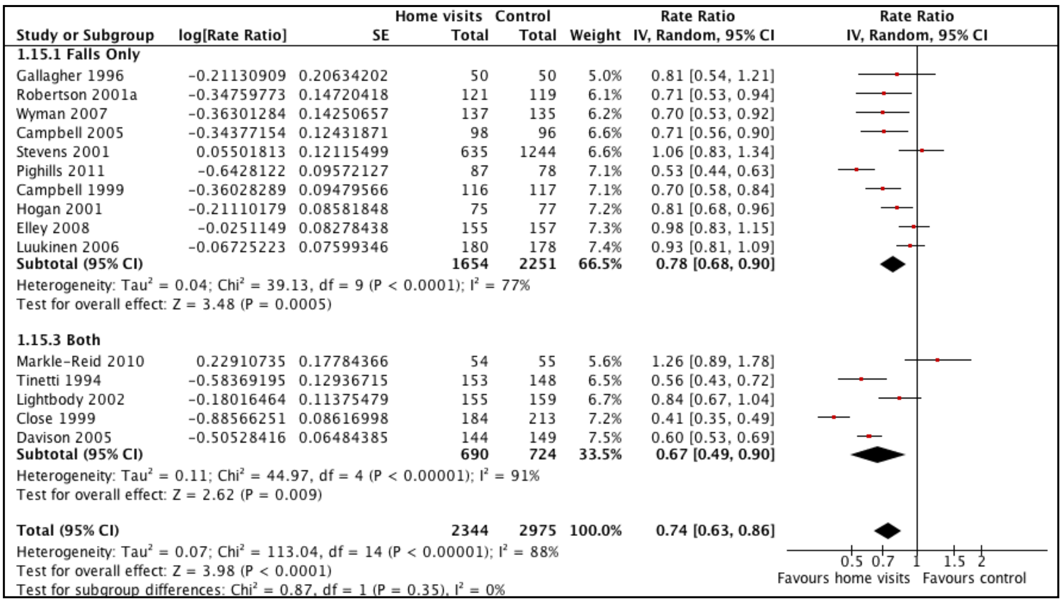

Analysis 16: Falls (number); age of participants subgroups

Study or Subgroup	log[Rate Ratio]	SE	Home visits Total	Control Total	Weight	Rate Ratio IV, Random, 95% CI
**1.16.2 71-75**						
Gallagher 1996	−0.21130909	0.20634202	50	50	5.2%	0.81 [0.54, 1.21]
Lightbody 2002	−0.18016464	0.11375479	155	159	7.1%	0.84 [0.67, 1.04]
**Subtotal (95% CI)**			**205**	**209**	**12.4%**	**0.83 [0.68, 1.01]**
Heterogeneity: Tau² = 0.00; Chi² = 0.02, df = 1 (P = 0.89); I² = 0%						
Test for overall effect: Z = 1.88 (P = 0.06)						
**1.16.3 76-80**						
Wyman 2007	−0.36301284	0.14250657	137	135	6.6%	0.70 [0.53, 0.92]
Tinetti 1994	−0.58369195	0.12936715	153	148	6.8%	0.56 [0.43, 0.72]
Stevens 2001	0.05501813	0.12115499	635	1244	7.0%	1.06 [0.83, 1.34]
Pighills 2011	−0.6428122	0.09572127	87	78	7.5%	0.53 [0.44, 0.63]
Close 1999	−0.88566251	0.08616998	184	213	7.7%	0.41 [0.35, 0.49]
Hogan 2001	−0.21110179	0.08581848	75	77	7.7%	0.81 [0.68, 0.96]
Davison 2005	−0.50528416	0.06484385	144	149	8.0%	0.60 [0.53, 0.69]
**Subtotal (95% CI)**			**1415**	**2044**	**51.2%**	**0.64 [0.51, 0.79]**
Heterogeneity: Tau² = 0.08; Chi² = 55.80, df = 6 (P < 0.00001); I² = 89%						
Test for overall effect: Z = 4.05 (P < 0.0001)						
**1.16.4 81-85**						
Robertson 2001a	−0.34759773	0.14720418	121	119	6.5%	0.71 [0.53, 0.94]
Campbell 2005	−0.34377154	0.12431871	98	96	6.9%	0.71 [0.56, 0.90]
Campbell 1999	−0.36028289	0.09479566	116	117	7.5%	0.70 [0.58, 0.84]
Elley 2008	−0.0251149	0.08278438	155	157	7.7%	0.98 [0.83, 1.15]
**Subtotal (95% CI)**			**490**	**489**	**28.6%**	**0.77 [0.64, 0.94]**
Heterogeneity: Tau² = 0.02; Chi² = 9.47, df = 3 (P = 0.02); I² = 68%						
Test for overall effect: Z = 2.64 (P = 0.008)						
**1.16.5 86+**						
Luukinen 2006	−0.06725223	0.07599346	180	178	7.8%	0.93 [0.81, 1.09]
**Subtotal (95% CI)**			**180**	**178**	**7.8%**	**0.93 [0.81, 1.09]**
Heterogeneity: Not applicable						
Test for overall effect: Z = 0.88 (P = 0.38)						
**Total (95% CI)**			**2290**	**2920**	**100.0%**	**0.71 [0.61, 0.83]**
Heterogeneity: Tau² = 0.07; Chi² = 102.74, df = 13 (P < 0.00001); I² = 87%						
Test for overall effect: Z = 4.41 (P < 0.0001)						
Test for subgroup differences: Chi² = 8.51, df = 3 (P = 0.04), I² = 64.7%						

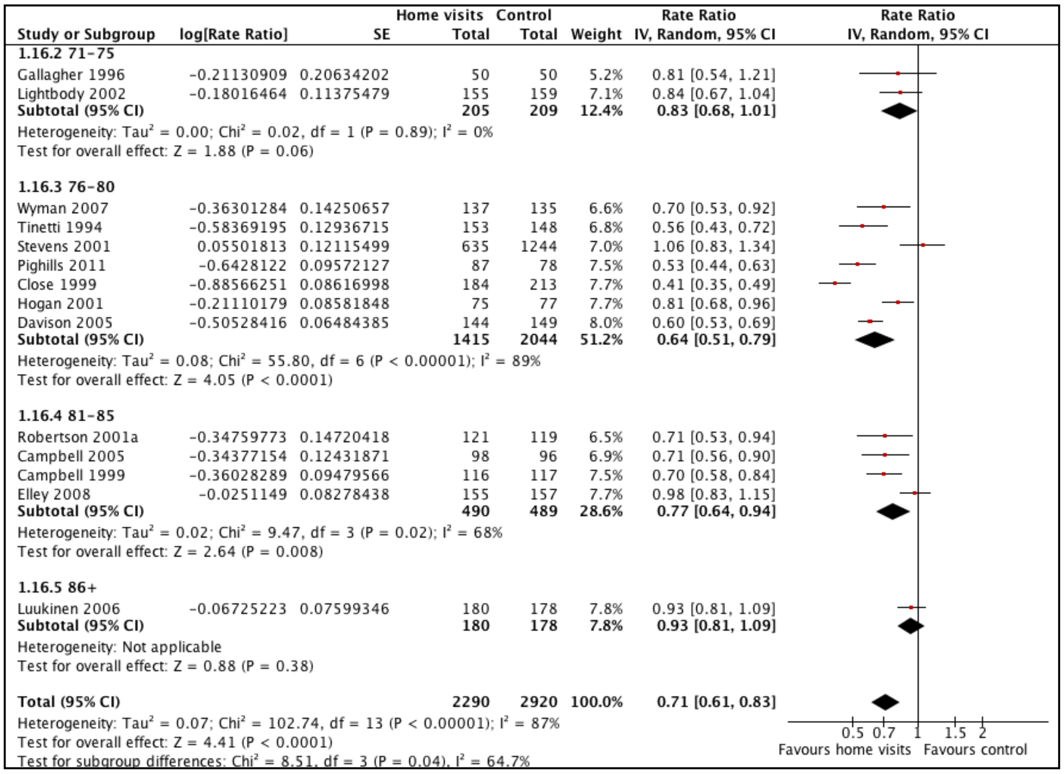

Analysis 17: Falls (number); type of visitor subgroups

Study or Subgroup	log[Rate Ratio]	SE	Home visits Total	Control Total	Weight	Rate Ratio IV, Random, 95% CI
**1.17.1 Nurse**						
Gallagher 1996	−0.21130909	0.20634202	50	50	5.0%	0.81 [0.54, 1.21]
Robertson 2001a	−0.34759773	0.14720418	121	119	6.1%	0.71 [0.53, 0.94]
Wyman 2007	−0.36301284	0.14250657	137	135	6.2%	0.70 [0.53, 0.92]
Stevens 2001	0.05501813	0.12115499	635	1244	6.6%	1.06 [0.83, 1.34]
Lightbody 2002	−0.18016464	0.11375479	155	159	6.7%	0.84 [0.67, 1.04]
Elley 2008	−0.0251149	0.08278438	155	157	7.3%	0.98 [0.83, 1.15]
**Subtotal (95% CI)**			**1253**	**1864**	**38.0%**	**0.86 [0.75, 0.98]**
Heterogeneity: Tau² = 0.01; Chi² = 9.14, df = 5 (P = 0.10); I² = 45%						
Test for overall effect: Z = 2.18 (P = 0.03)						
**1.17.2 Other**						
Pighills 2011	−0.6428122	0.09572127	87	78	7.1%	0.53 [0.44, 0.63]
Campbell 1999	−0.36028289	0.09479566	116	117	7.1%	0.70 [0.58, 0.84]
Close 1999	−0.88566251	0.08616998	184	213	7.2%	0.41 [0.35, 0.49]
**Subtotal (95% CI)**			**387**	**408**	**21.3%**	**0.53 [0.39, 0.72]**
Heterogeneity: Tau² = 0.06; Chi² = 16.82, df = 2 (P = 0.0002); I² = 88%						
Test for overall effect: Z = 4.09 (P < 0.0001)						
**1.17.3 Combined**						
Markle-Reid 2010	0.22910735	0.17784366	54	55	5.6%	1.26 [0.89, 1.78]
Tinetti 1994	−0.58369195	0.12936715	153	148	6.5%	0.56 [0.43, 0.72]
Campbell 2005	−0.34377154	0.12431871	98	96	6.6%	0.71 [0.56, 0.90]
Hogan 2001	−0.21110179	0.08581848	75	77	7.2%	0.81 [0.68, 0.96]
Luukinen 2006	−0.06725223	0.07599346	180	178	7.4%	0.93 [0.81, 1.09]
Davison 2005	−0.50528416	0.06484385	144	149	7.5%	0.60 [0.53, 0.69]
**Subtotal (95% CI)**			**704**	**703**	**40.7%**	**0.77 [0.63, 0.94]**
Heterogeneity: Tau² = 0.05; Chi² = 34.26, df = 5 (P < 0.00001); I² = 85%						
Test for overall effect: Z = 2.50 (P = 0.01)						
**Total (95% CI)**			**2344**	**2975**	**100.0%**	**0.74 [0.63, 0.86]**
Heterogeneity: Tau² = 0.07; Chi² = 113.04, df = 14 (P < 0.00001); I² = 88%						
Test for overall effect: Z = 3.98 (P < 0.0001)						
Test for subgroup differences: Chi² = 8.00, df = 2 (P = 0.02), I² = 75.0%						

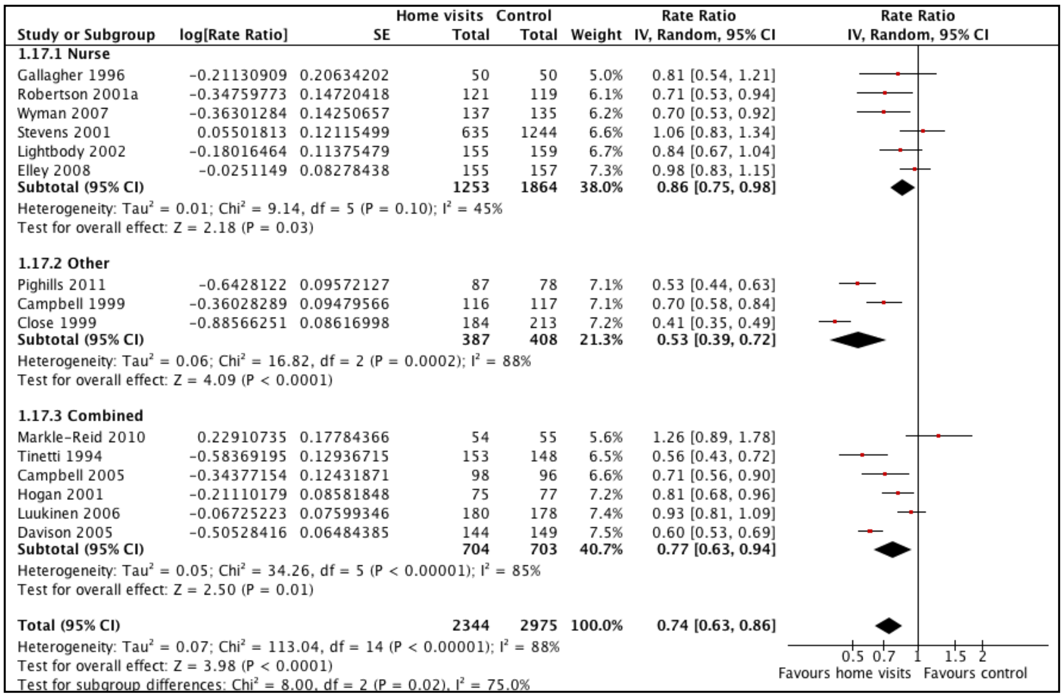

Analysis 18: Falls (number); number of visits subgroups

Study or Subgroup	log[Rate Ratio]	SE	Home visits Total	Control Total	Weight	Rate Ratio IV, Random, 95% CI
**1.18.1 1 Visit**						
Stevens 2001	0.05501813	0.12115499	635	1244	7.8%	1.06 [0.83, 1.34]
Lightbody 2002	−0.18016464	0.11375479	155	159	7.9%	0.84 [0.67, 1.04]
Pighills 2011	−0.6428122	0.09572127	87	78	8.2%	0.53 [0.44, 0.63]
Close 1999	−0.88566251	0.08616998	184	213	8.4%	0.41 [0.35, 0.49]
**Subtotal (95% CI)**			**1061**	**1694**	**32.3%**	**0.66 [0.43, 1.00]**
Heterogeneity: Tau^2 = 0.17; Chi^2 = 51.08, df = 3 (P < 0.00001); I^2 = 94%						
Test for overall effect: Z = 1.98 (P = 0.05)						
**1.18.2 2-4 Visits**						
Gallagher 1996	−0.21130909	0.20634202	50	50	6.0%	0.81 [0.54, 1.21]
Campbell 1999	−0.36028289	0.09479566	116	117	8.2%	0.70 [0.58, 0.84]
Hogan 2001	−0.21110179	0.08581848	75	77	8.4%	0.81 [0.68, 0.96]
Luukinen 2006	−0.06725223	0.07599346	180	178	8.6%	0.93 [0.81, 1.09]
**Subtotal (95% CI)**			**421**	**422**	**31.2%**	**0.82 [0.71, 0.94]**
Heterogeneity: Tau^2 = 0.01; Chi^2 = 5.90, df = 3 (P = 0.12); I^2 = 49%						
Test for overall effect: Z = 2.87 (P = 0.004)						
**1.18.3 5 or More Visits**						
Markle-Reid 2010	0.22910735	0.17784366	54	55	6.6%	1.26 [0.89, 1.78]
Robertson 2001a	−0.34759773	0.14720418	121	119	7.2%	0.71 [0.53, 0.94]
Wyman 2007	−0.36301284	0.14250657	137	135	7.3%	0.70 [0.53, 0.92]
Tinetti 1994	−0.58369195	0.12936715	153	148	7.6%	0.56 [0.43, 0.72]
Campbell 2005	−0.34377154	0.12431871	98	96	7.7%	0.71 [0.56, 0.90]
**Subtotal (95% CI)**			**563**	**553**	**36.5%**	**0.74 [0.59, 0.93]**
Heterogeneity: Tau^2 = 0.05; Chi^2 = 13.81, df = 4 (P = 0.008); I^2 = 71%						
Test for overall effect: Z = 2.53 (P = 0.01)						
**Total (95% CI)**			**2045**	**2669**	**100.0%**	**0.73 [0.62, 0.87]**
Heterogeneity: Tau^2 = 0.08; Chi^2 = 92.10, df = 12 (P < 0.00001); I^2 = 87%						
Test for overall effect: Z = 3.59 (P = 0.0003)						
Test for subgroup differences: Chi^2 = 1.22, df = 2 (P = 0.54), I^2 = 0%						

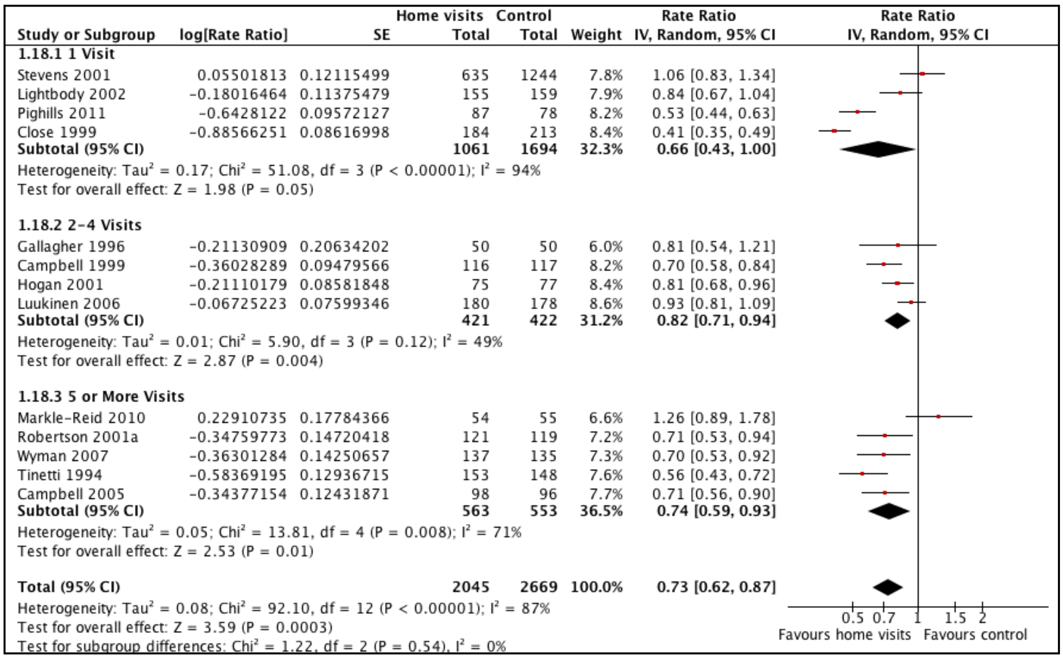

Analysis 19: Falls (people) at each follow-up interval

Study or Subgroup	log[Odds Ratio]	SE	Home visits Total	Control Total	Weight	Odds Ratio IV, Random, 95% CI
**1.20.1 01-11 months**						
Huang 2004	−2.2154069	1.50286023	55	58	1.5%	0.11 [0.01, 2.08]
Lightbody 2002	−0.0329	0.5833857	155	159	8.4%	0.97 [0.31, 3.04]
Lin 2007	−0.46798547	0.5833857	39	40	8.4%	0.63 [0.20, 1.96]
Ciaschini 2009	0.52623569	0.35023791	101	100	17.1%	1.69 [0.85, 3.36]
Green 2002	0.3769288	0.3375845	81	80	17.8%	1.46 [0.75, 2.83]
Hendriks 2008	0.30155443	0.26740693	131	143	22.7%	1.35 [0.80, 2.28]
Tinetti 1994	−0.36923617	0.24870421	147	144	24.1%	0.69 [0.42, 1.13]
**Subtotal (95% CI)**			**709**	**724**	**100.0%**	**1.06 [0.73, 1.54]**
Heterogeneity: Tau^2 = 0.09; Chi^2 = 9.58, df = 6 (P = 0.14); I^2 = 37%						
Test for overall effect: Z = 0.32 (P = 0.75)						
**1.20.2 12-23 months**						
Liu-Ambrose 2008	−0.98082925	0.57735027	28	24	1.9%	0.38 [0.12, 1.16]
Newbury 2001	−0.54897739	0.45770835	45	44	2.8%	0.58 [0.24, 1.42]
Hogan 2001	−0.39382353	0.38082937	75	77	3.8%	0.67 [0.32, 1.42]
Fabacher 1994	−0.61587298	0.37710695	100	95	3.9%	0.54 [0.26, 1.13]
Pighills 2011	−0.0873	0.3233	87	78	4.9%	0.92 [0.49, 1.73]
Campbell 2005	−0.54829756	0.29130846	98	96	5.6%	0.58 [0.33, 1.02]
Campbell 1999	−0.29264401	0.26277707	116	117	6.5%	0.75 [0.45, 1.25]
van Haastregt 2000	0.25087224	0.2621486	120	115	6.5%	1.29 [0.77, 2.15]
Hendriks 2008	−0.04718774	0.25053272	124	134	6.8%	0.95 [0.58, 1.56]
Wyman 2007	−0.17249982	0.24912063	131	132	6.9%	0.84 [0.52, 1.37]
Davison 2005	−0.14355343	0.24843272	144	149	6.9%	0.87 [0.53, 1.41]
Tinetti 1994	−0.49140754	0.24004346	147	144	7.2%	0.61 [0.38, 0.98]
Luukinen 2006	−0.32768741	0.24003644	180	178	7.2%	0.72 [0.45, 1.15]
Elley 2008	0.26418876	0.23873701	155	157	7.2%	1.30 [0.82, 2.08]
Close 1999	−0.83533368	0.20919389	184	213	8.4%	0.43 [0.29, 0.65]
Stevens 2001	−0.07257069	0.10904384	635	1244	13.4%	0.93 [0.75, 1.15]
**Subtotal (95% CI)**			**2369**	**2997**	**100.0%**	**0.77 [0.66, 0.91]**
Heterogeneity: Tau^2 = 0.04; Chi^2 = 24.98, df = 15 (P = 0.05); I^2 = 40%						
Test for overall effect: Z = 3.03 (P = 0.002)						
**1.20.3 24-35 months**						
Sorensen 1988	0.10790975	0.17417759	367	404	100.0%	1.11 [0.79, 1.57]
**Subtotal (95% CI)**			**367**	**404**	**100.0%**	**1.11 [0.79, 1.57]**
Heterogeneity: Not applicable						
Test for overall effect: Z = 0.62 (P = 0.54)						
**1.20.4 36+ months**						
Vetter 1992	0.37948962	0.19925871	240	210	100.0%	1.46 [0.99, 2.16]
**Subtotal (95% CI)**			**240**	**210**	**100.0%**	**1.46 [0.99, 2.16]**
Heterogeneity: Not applicable						
Test for overall effect: Z = 1.90 (P = 0.06)						

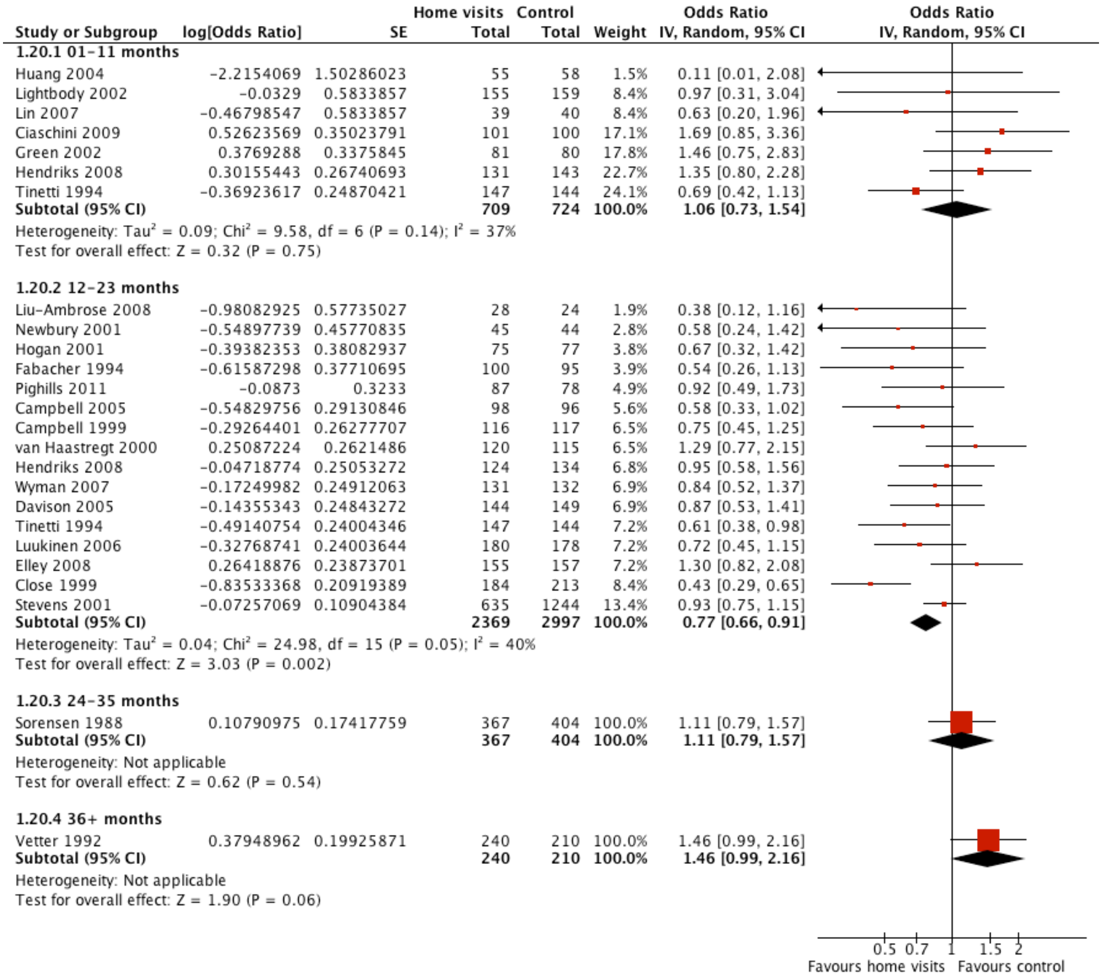

Analysis 20: Falls (people); focus of visit subgroups

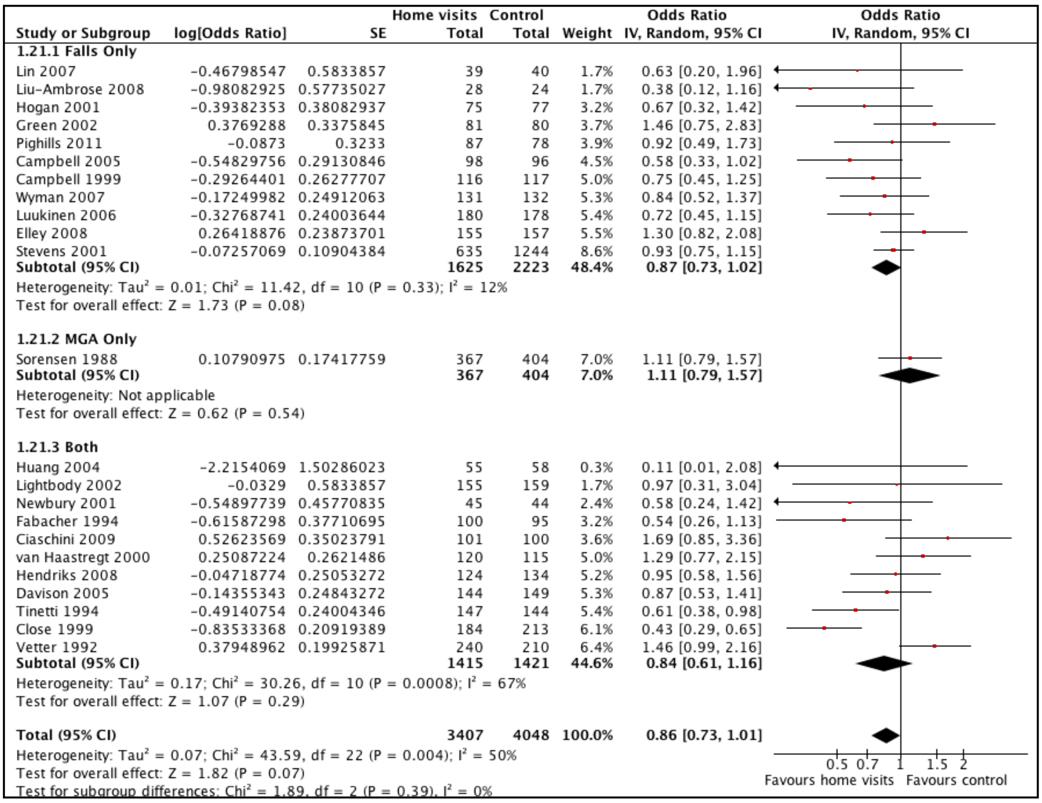

Study or Subgroup	log[Odds Ratio]	SE	Home visits Total	Control Total	Weight	Odds Ratio IV, Random, 95% CI
**1.21.1 Falls Only**						
Lin 2007	−0.46798547	0.5833857	39	40	1.7%	0.63 [0.20, 1.96]
Liu-Ambrose 2008	−0.98082925	0.57735027	28	24	1.7%	0.38 [0.12, 1.16]
Hogan 2001	−0.39382353	0.38082937	75	77	3.2%	0.67 [0.32, 1.42]
Green 2002	0.3769288	0.3375845	81	80	3.7%	1.46 [0.75, 2.83]
Pighills 2011	−0.0873	0.3233	87	78	3.9%	0.92 [0.49, 1.73]
Campbell 2005	−0.54829756	0.29130846	98	96	4.5%	0.58 [0.33, 1.02]
Campbell 1999	−0.29264401	0.26277707	116	117	5.0%	0.75 [0.45, 1.25]
Wyman 2007	−0.17249982	0.24912063	131	132	5.3%	0.84 [0.52, 1.37]
Luukinen 2006	−0.32768741	0.24003644	180	178	5.4%	0.72 [0.45, 1.15]
Elley 2008	0.26418876	0.23873701	155	157	5.5%	1.30 [0.82, 2.08]
Stevens 2001	−0.07257069	0.10904384	635	1244	8.6%	0.93 [0.75, 1.15]
**Subtotal (95% CI)**			**1625**	**2223**	**48.4%**	**0.87 [0.73, 1.02]**
Heterogeneity: Tau² = 0.01; Chi² = 11.42, df = 10 (P = 0.33); I² = 12%						
Test for overall effect: Z = 1.73 (P = 0.08)						
**1.21.2 MGA Only**						
Sorensen 1988	0.10790975	0.17417759	367	404	7.0%	1.11 [0.79, 1.57]
**Subtotal (95% CI)**			**367**	**404**	**7.0%**	**1.11 [0.79, 1.57]**
Heterogeneity: Not applicable						
Test for overall effect: Z = 0.62 (P = 0.54)						
**1.21.3 Both**						
Huang 2004	−2.2154069	1.50286023	55	58	0.3%	0.11 [0.01, 2.08]
Lightbody 2002	−0.0329	0.5833857	155	159	1.7%	0.97 [0.31, 3.04]
Newbury 2001	−0.54897739	0.45770835	45	44	2.4%	0.58 [0.24, 1.42]
Fabacher 1994	−0.61587298	0.37710695	100	95	3.2%	0.54 [0.26, 1.13]
Ciaschini 2009	0.52623569	0.35023791	101	100	3.6%	1.69 [0.85, 3.36]
van Haastregt 2000	0.25087224	0.2621486	120	115	5.0%	1.29 [0.77, 2.15]
Hendriks 2008	−0.04718774	0.25053272	124	134	5.2%	0.95 [0.58, 1.56]
Davison 2005	−0.14355343	0.24843272	144	149	5.3%	0.87 [0.53, 1.41]
Tinetti 1994	−0.49140754	0.24004346	147	144	5.4%	0.61 [0.38, 0.98]
Close 1999	−0.83533368	0.20919389	184	213	6.1%	0.43 [0.29, 0.65]
Vetter 1992	0.37948962	0.19925871	240	210	6.4%	1.46 [0.99, 2.16]
**Subtotal (95% CI)**			**1415**	**1421**	**44.6%**	**0.84 [0.61, 1.16]**
Heterogeneity: Tau² = 0.17; Chi² = 30.26, df = 10 (P = 0.0008); I² = 67%						
Test for overall effect: Z = 1.07 (P = 0.29)						
**Total (95% CI)**			**3407**	**4048**	**100.0%**	**0.86 [0.73, 1.01]**
Heterogeneity: Tau² = 0.07; Chi² = 43.59, df = 22 (P = 0.004); I² = 50%						
Test for overall effect: Z = 1.82 (P = 0.07)						
Test for subgroup differences: Chi² = 1.89, df = 2 (P = 0.39), I² = 0%						

Analysis 21: Falls (people); age of participants subgroups

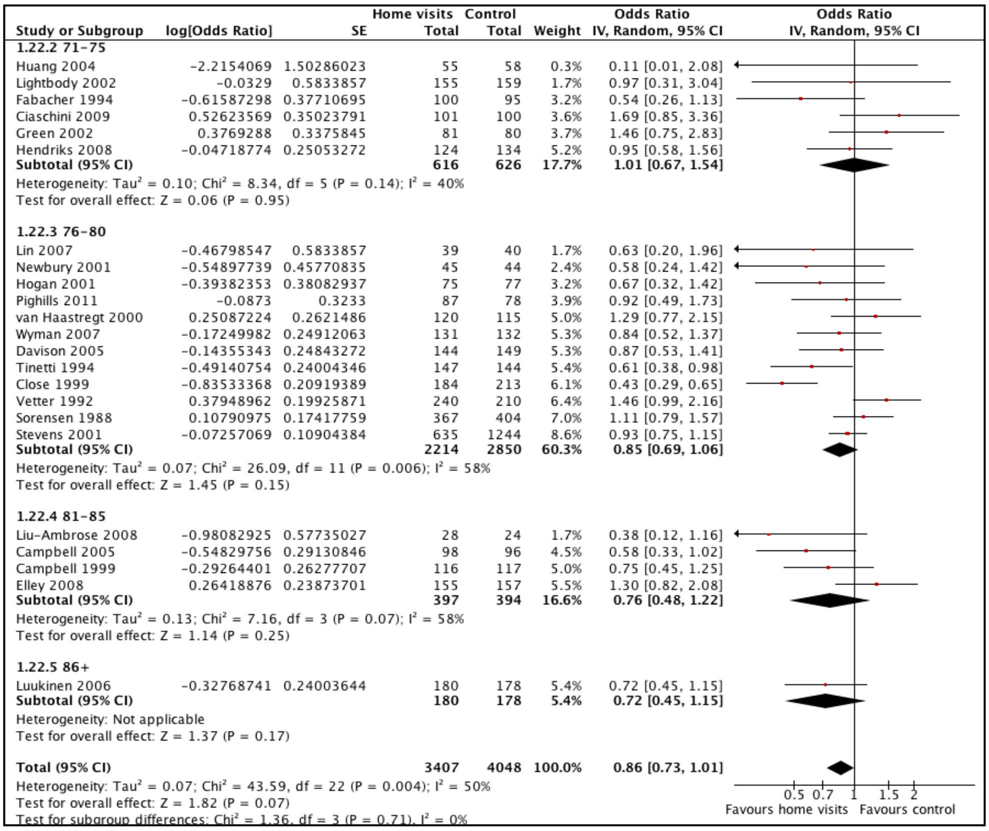

Study or Subgroup	log[Odds Ratio]	SE	Home visits Total	Control Total	Weight	Odds Ratio IV, Random, 95% CI
**1.22.2 71–75**						
Huang 2004	−2.2154069	1.50286023	55	58	0.3%	0.11 [0.01, 2.08]
Lightbody 2002	−0.0329	0.5833857	155	159	1.7%	0.97 [0.31, 3.04]
Fabacher 1994	−0.61587298	0.37710695	100	95	3.2%	0.54 [0.26, 1.13]
Ciaschini 2009	0.52623569	0.35023791	101	100	3.6%	1.69 [0.85, 3.36]
Green 2002	0.3769288	0.3375845	81	80	3.7%	1.46 [0.75, 2.83]
Hendriks 2008	−0.04718774	0.25053272	124	134	5.2%	0.95 [0.58, 1.56]
**Subtotal (95% CI)**			**616**	**626**	**17.7%**	**1.01 [0.67, 1.54]**
Heterogeneity: Tau² = 0.10; Chi² = 8.34, df = 5 (P = 0.14); I² = 40%						
Test for overall effect: Z = 0.06 (P = 0.95)						
**1.22.3 76–80**						
Lin 2007	−0.46798547	0.5833857	39	40	1.7%	0.63 [0.20, 1.96]
Newbury 2001	−0.54897739	0.45770835	45	44	2.4%	0.58 [0.24, 1.42]
Hogan 2001	−0.39382353	0.38082937	75	77	3.2%	0.67 [0.32, 1.42]
Pighills 2011	−0.0873	0.3233	87	78	3.9%	0.92 [0.49, 1.73]
van Haastregt 2000	0.25087224	0.2621486	120	115	5.0%	1.29 [0.77, 2.15]
Wyman 2007	−0.17249982	0.24912063	131	132	5.3%	0.84 [0.52, 1.37]
Davison 2005	−0.14355343	0.24843272	144	149	5.3%	0.87 [0.53, 1.41]
Tinetti 1994	−0.49140754	0.24004346	147	144	5.4%	0.61 [0.38, 0.98]
Close 1999	−0.83533368	0.20919389	184	213	6.1%	0.43 [0.29, 0.65]
Vetter 1992	0.37948962	0.19925871	240	210	6.4%	1.46 [0.99, 2.16]
Sorensen 1988	0.10790975	0.17417759	367	404	7.0%	1.11 [0.79, 1.57]
Stevens 2001	−0.07257069	0.10904384	635	1244	8.6%	0.93 [0.75, 1.15]
**Subtotal (95% CI)**			**2214**	**2850**	**60.3%**	**0.85 [0.69, 1.06]**
Heterogeneity: Tau² = 0.07; Chi² = 26.09, df = 11 (P = 0.006); I² = 58%						
Test for overall effect: Z = 1.45 (P = 0.15)						
**1.22.4 81–85**						
Liu-Ambrose 2008	−0.98082925	0.57735027	28	24	1.7%	0.38 [0.12, 1.16]
Campbell 2005	−0.54829756	0.29130846	98	96	4.5%	0.58 [0.33, 1.02]
Campbell 1999	−0.29264401	0.26277707	116	117	5.0%	0.75 [0.45, 1.25]
Elley 2008	0.26418876	0.23873701	155	157	5.5%	1.30 [0.82, 2.08]
**Subtotal (95% CI)**			**397**	**394**	**16.6%**	**0.76 [0.48, 1.22]**
Heterogeneity: Tau² = 0.13; Chi² = 7.16, df = 3 (P = 0.07); I² = 58%						
Test for overall effect: Z = 1.14 (P = 0.25)						
**1.22.5 86+**						
Luukinen 2006	−0.32768741	0.24003644	180	178	5.4%	0.72 [0.45, 1.15]
**Subtotal (95% CI)**			**180**	**178**	**5.4%**	**0.72 [0.45, 1.15]**
Heterogeneity: Not applicable						
Test for overall effect: Z = 1.37 (P = 0.17)						
**Total (95% CI)**			**3407**	**4048**	**100.0%**	**0.86 [0.73, 1.01]**
Heterogeneity: Tau² = 0.07; Chi² = 43.59, df = 22 (P = 0.004); I² = 50%						
Test for overall effect: Z = 1.82 (P = 0.07)						
Test for subgroup differences: Chi² = 1.36, df = 3 (P = 0.71), I² = 0%						

Analysis 22: Falls (people); type of visitor subgroups

Study or Subgroup	log[Odds Ratio]	SE	Home visits Total	Control Total	Weight	Odds Ratio IV, Random, 95% CI
**1.23.1 Nurse**						
Lightbody 2002	−0.0329	0.5833857	155	159	1.7%	0.97 [0.31, 3.04]
Newbury 2001	−0.54897739	0.45770835	45	44	2.4%	0.58 [0.24, 1.42]
Ciaschini 2009	0.52623569	0.35023791	101	100	3.6%	1.69 [0.85, 3.36]
van Haastregt 2000	0.25087224	0.2621486	120	115	5.0%	1.29 [0.77, 2.15]
Wyman 2007	−0.17249982	0.24912063	131	132	5.3%	0.84 [0.52, 1.37]
Elley 2008	0.26418876	0.23873701	155	157	5.5%	1.30 [0.82, 2.08]
Stevens 2001	−0.07257069	0.10904384	635	1244	8.6%	0.93 [0.75, 1.15]
**Subtotal (95% CI)**			**1342**	**1951**	**32.0%**	**1.02 [0.85, 1.24]**
Heterogeneity: Tau² = 0.01; Chi² = 6.76, df = 6 (P = 0.34); I² = 11%						
Test for overall effect: Z = 0.25 (P = 0.80)						
**1.23.2 Other**						
Huang 2004	−2.2154069	1.50286023	55	58	0.3%	0.11 [0.01, 2.08]
Lin 2007	−0.46798547	0.5833857	39	40	1.7%	0.63 [0.20, 1.96]
Liu-Ambrose 2008	−0.98082925	0.57735027	28	24	1.7%	0.38 [0.12, 1.16]
Green 2002	0.3769288	0.3375845	81	80	3.7%	1.46 [0.75, 2.83]
Pighills 2011	−0.0873	0.3233	87	78	3.9%	0.92 [0.49, 1.73]
Campbell 1999	−0.29264401	0.26277707	116	117	5.0%	0.75 [0.45, 1.25]
Close 1999	−0.83533368	0.20919389	184	213	6.1%	0.43 [0.29, 0.65]
Vetter 1992	0.37948962	0.19925871	240	210	6.4%	1.46 [0.99, 2.16]
**Subtotal (95% CI)**			**830**	**820**	**28.8%**	**0.77 [0.49, 1.21]**
Heterogeneity: Tau² = 0.25; Chi² = 24.68, df = 7 (P = 0.0009); I² = 72%						
Test for overall effect: Z = 1.14 (P = 0.26)						
**1.23.3 Combined**						
Hogan 2001	−0.39382353	0.38082937	75	77	3.2%	0.67 [0.32, 1.42]
Fabacher 1994	−0.61587298	0.37710695	100	95	3.2%	0.54 [0.26, 1.13]
Campbell 2005	−0.54829756	0.29130846	98	96	4.5%	0.58 [0.33, 1.02]
Hendriks 2008	−0.04718774	0.25053272	124	134	5.2%	0.95 [0.58, 1.56]
Davison 2005	−0.14355343	0.24843272	144	149	5.3%	0.87 [0.53, 1.41]
Tinetti 1994	−0.49140754	0.24004346	147	144	5.4%	0.61 [0.38, 0.98]
Luukinen 2006	−0.32768741	0.24003644	180	178	5.4%	0.72 [0.45, 1.15]
Sorensen 1988	0.10790975	0.17417759	367	404	7.0%	1.11 [0.79, 1.57]
**Subtotal (95% CI)**			**1235**	**1277**	**39.2%**	**0.79 [0.65, 0.96]**
Heterogeneity: Tau² = 0.01; Chi² = 8.17, df = 7 (P = 0.32); I² = 14%						
Test for overall effect: Z = 2.37 (P = 0.02)						
**Total (95% CI)**			**3407**	**4048**	**100.0%**	**0.86 [0.73, 1.01]**
Heterogeneity: Tau² = 0.07; Chi² = 43.59, df = 22 (P = 0.004); I² = 50%						
Test for overall effect: Z = 1.82 (P = 0.07)						
Test for subgroup differences: Chi² = 3.94, df = 2 (P = 0.14), I² = 49.3%						

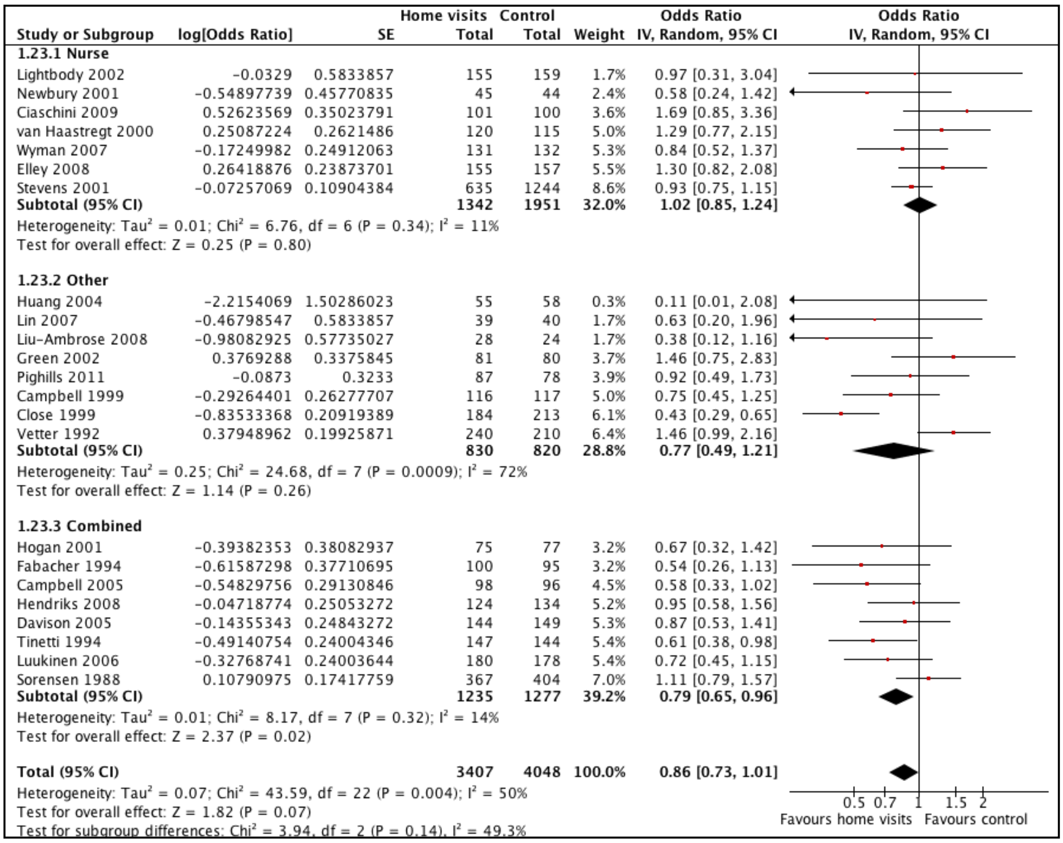

Analysis 23: Falls (people); number of visits subgroups

Study or Subgroup	log[Odds Ratio]	SE	Home visits Total	Control Total	Weight	Odds Ratio IV, Random, 95% CI
**1.24.1 1 Visit**						
Lightbody 2002	−0.0329	0.5833857	155	159	1.9%	0.97 [0.31, 3.04]
Newbury 2001	−0.54897739	0.45770835	45	44	2.8%	0.58 [0.24, 1.42]
Pighills 2011	−0.0873	0.3233	87	78	4.6%	0.92 [0.49, 1.73]
Hendriks 2008	−0.04718774	0.25053272	124	134	6.1%	0.95 [0.58, 1.56]
Close 1999	−0.83533368	0.20919389	184	213	7.2%	0.43 [0.29, 0.65]
Sorensen 1988	0.10790975	0.17417759	367	404	8.2%	1.11 [0.79, 1.57]
Stevens 2001	−0.07257069	0.10904384	635	1244	10.2%	0.93 [0.75, 1.15]
**Subtotal (95% CI)**			**1597**	**2276**	**40.9%**	**0.82 [0.62, 1.08]**
Heterogeneity: Tau² = 0.07; Chi² = 14.44, df = 6 (P = 0.03); I² = 58%						
Test for overall effect: Z = 1.42 (P = 0.16)						
**1.24.2 2-4 Visits**						
Huang 2004	−2.2154069	1.50286023	55	58	0.3%	0.11 [0.01, 2.08]
Hogan 2001	−0.39382353	0.38082937	75	77	3.7%	0.67 [0.32, 1.42]
Fabacher 1994	−0.61587298	0.37710695	100	95	3.7%	0.54 [0.26, 1.13]
Green 2002	0.3769288	0.3375845	81	80	4.3%	1.46 [0.75, 2.83]
Campbell 1999	−0.29264401	0.26277707	116	117	5.8%	0.75 [0.45, 1.25]
Luukinen 2006	−0.32768741	0.24003644	180	178	6.4%	0.72 [0.45, 1.15]
Vetter 1992	0.37948962	0.19925871	240	210	7.4%	1.46 [0.99, 2.16]
**Subtotal (95% CI)**			**847**	**815**	**31.7%**	**0.87 [0.61, 1.24]**
Heterogeneity: Tau² = 0.12; Chi² = 13.56, df = 6 (P = 0.03); I² = 56%						
Test for overall effect: Z = 0.77 (P = 0.44)						
**1.24.3 5 or More Visits**						
Lin 2007	−0.46798547	0.5833857	39	40	1.9%	0.63 [0.20, 1.96]
Liu-Ambrose 2008	−0.98082925	0.57735027	28	24	1.9%	0.38 [0.12, 1.16]
Campbell 2005	−0.54829756	0.29130846	98	96	5.2%	0.58 [0.33, 1.02]
van Haastregt 2000	0.25087224	0.2621486	120	115	5.8%	1.29 [0.77, 2.15]
Wyman 2007	−0.17249982	0.24912063	131	132	6.1%	0.84 [0.52, 1.37]
Tinetti 1994	−0.49140754	0.24004346	147	144	6.4%	0.61 [0.38, 0.98]
**Subtotal (95% CI)**			**563**	**551**	**27.3%**	**0.74 [0.54, 1.01]**
Heterogeneity: Tau² = 0.05; Chi² = 7.49, df = 5 (P = 0.19); I² = 33%						
Test for overall effect: Z = 1.90 (P = 0.06)						
**Total (95% CI)**			**3007**	**3642**	**100.0%**	**0.82 [0.69, 0.97]**
Heterogeneity: Tau² = 0.06; Chi² = 37.20, df = 19 (P = 0.007); I² = 49%						
Test for overall effect: Z = 2.31 (P = 0.02)						
Test for subgroup differences: Chi² = 0.46, df = 2 (P = 0.79), I² = 0%						

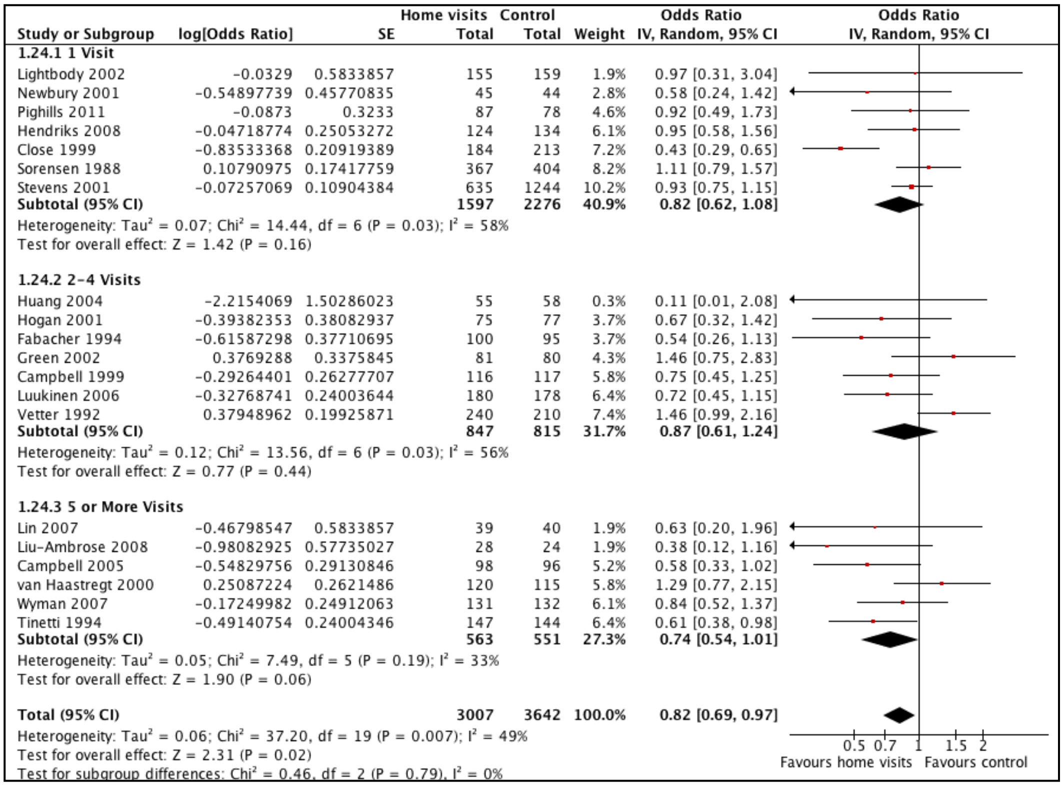

Analysis 24: Falls (subjective), results at longest follow-up

Study or Subgroup	Std. Mean Difference	SE	Home visits Total	Control Total	Weight	Std. Mean Difference IV, Random, 95% CI
Liu-Ambrose 2008	0	0.27817432	28	24	2.8%	0.00 [−0.55, 0.55]
Kono 2004	−0.02347903	0.23479855	35	36	3.7%	−0.02 [−0.48, 0.44]
Lin 2007	−0.64046659	0.22858767	39	40	3.9%	−0.64 [−1.09, −0.19]
Markle-Reid 2010	−0.03791867	0.20723195	49	43	4.6%	−0.04 [−0.44, 0.37]
Gallagher 1996	−0.09310776	0.19859121	50	50	4.9%	−0.09 [−0.48, 0.30]
Huang 2004	0.0645922	0.18698532	55	58	5.4%	0.06 [−0.30, 0.43]
Pighills 2011	−0.10720186	0.15532549	87	78	7.2%	−0.11 [−0.41, 0.20]
Hendriks 2008	−0.07620556	0.14132418	124	134	8.2%	−0.08 [−0.35, 0.20]
Campbell 1999	−0.30709109	0.13743163	103	110	8.5%	−0.31 [−0.58, −0.04]
van Haastregt 2000	−0.22889926	0.13050247	120	115	9.1%	−0.23 [−0.48, 0.03]
Thomas 2007	0.13965832	0.1207089	204	103	10.1%	0.14 [−0.10, 0.38]
Gitlin 2006	−0.23125107	0.11873019	149	136	10.3%	−0.23 [−0.46, 0.00]
Tinetti 1994	−0.31569383	0.11767364	147	144	10.4%	−0.32 [−0.55, −0.09]
Davison 2005	−0.28073774	0.11361653	159	154	10.8%	−0.28 [−0.50, −0.06]
**Total (95% CI)**			**1349**	**1225**	**100.0%**	**−0.16 [−0.26, −0.07]**

Heterogeneity: $Tau^2 = 0.01$; $Chi^2 = 18.26$, df = 13 (P = 0.15); $I^2 = 29\%$
Test for overall effect: Z = 3.32 (P = 0.0009)

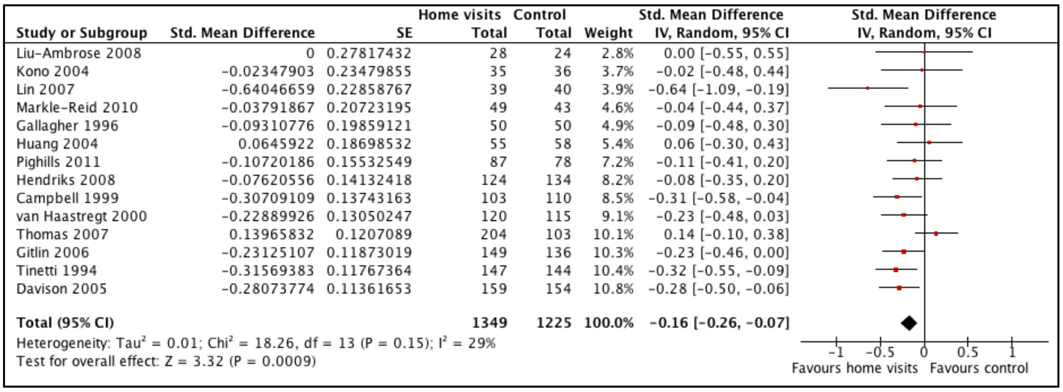

Analysis 25: Falls (subjective) at each follow-up interval

Study or Subgroup	Std. Mean Difference	SE	Home visits Total	Control Total	Weight	Std. Mean Difference IV, Random, 95% CI
**1.26.1 01-11 months**						
Liu-Ambrose 2008	0	0.27817432	28	24	8.5%	0.00 [−0.55, 0.55]
Lin 2007	−0.64046659	0.22858767	39	40	11.1%	−0.64 [−1.09, −0.19]
Markle-Reid 2010	−0.03791867	0.20723195	49	43	12.6%	−0.04 [−0.44, 0.37]
Gallagher 1996	−0.09310776	0.19859121	50	50	13.2%	−0.09 [−0.48, 0.30]
Huang 2004	0.0645922	0.18698532	55	58	14.2%	0.06 [−0.30, 0.43]
Hendriks 2008	0.21486493	0.13803665	131	143	18.9%	0.21 [−0.06, 0.49]
Gitlin 2006	−0.13807129	0.11536587	149	136	21.5%	−0.14 [−0.36, 0.09]
**Subtotal (95% CI)**			**501**	**494**	**100.0%**	**−0.07 [−0.25, 0.12]**
Heterogeneity: $Tau^2 = 0.03$; $Chi^2 = 11.39$, df = 6 (P = 0.08); $I^2 = 47\%$						
Test for overall effect: Z = 0.72 (P = 0.47)						
**1.26.2 12-23 months**						
Kono 2004	−0.02347903	0.23479855	35	36	4.0%	−0.02 [−0.48, 0.44]
Pighills 2011	−0.10720186	0.15532549	87	78	8.4%	−0.11 [−0.41, 0.20]
Hendriks 2008	−0.07620556	0.14132418	124	134	9.8%	−0.08 [−0.35, 0.20]
Campbell 1999	−0.30709109	0.13743163	103	110	10.3%	−0.31 [−0.58, −0.04]
van Haastregt 2000	−0.22889926	0.13050247	120	115	11.2%	−0.23 [−0.48, 0.03]
Gitlin 2006	−0.23125107	0.11873019	149	136	13.0%	−0.23 [−0.46, 0.00]
Tinetti 1994	−0.31569383	0.11767364	147	144	13.2%	−0.32 [−0.55, −0.09]
Davison 2005	−0.28073774	0.11361653	159	154	13.9%	−0.28 [−0.50, −0.06]
Thomas 2007	0.05677979	0.101518	299	143	16.4%	0.06 [−0.14, 0.26]
**Subtotal (95% CI)**			**1223**	**1050**	**100.0%**	**−0.18 [−0.27, −0.08]**
Heterogeneity: $Tau^2 = 0.00$; $Chi^2 = 9.92$, df = 8 (P = 0.27); $I^2 = 19\%$						
Test for overall effect: Z = 3.62 (P = 0.0003)						
**1.26.3 24-35 months**						
Thomas 2007	0.18207067	0.11020276	253	122	100.0%	0.18 [−0.03, 0.40]
**Subtotal (95% CI)**			**253**	**122**	**100.0%**	**0.18 [−0.03, 0.40]**
Heterogeneity: Not applicable						
Test for overall effect: Z = 1.65 (P = 0.10)						
**1.26.4 36+ months**						
Thomas 2007	0.13965832	0.1207089	204	103	100.0%	0.14 [−0.10, 0.38]
**Subtotal (95% CI)**			**204**	**103**	**100.0%**	**0.14 [−0.10, 0.38]**
Heterogeneity: Not applicable						
Test for overall effect: Z = 1.16 (P = 0.25)						

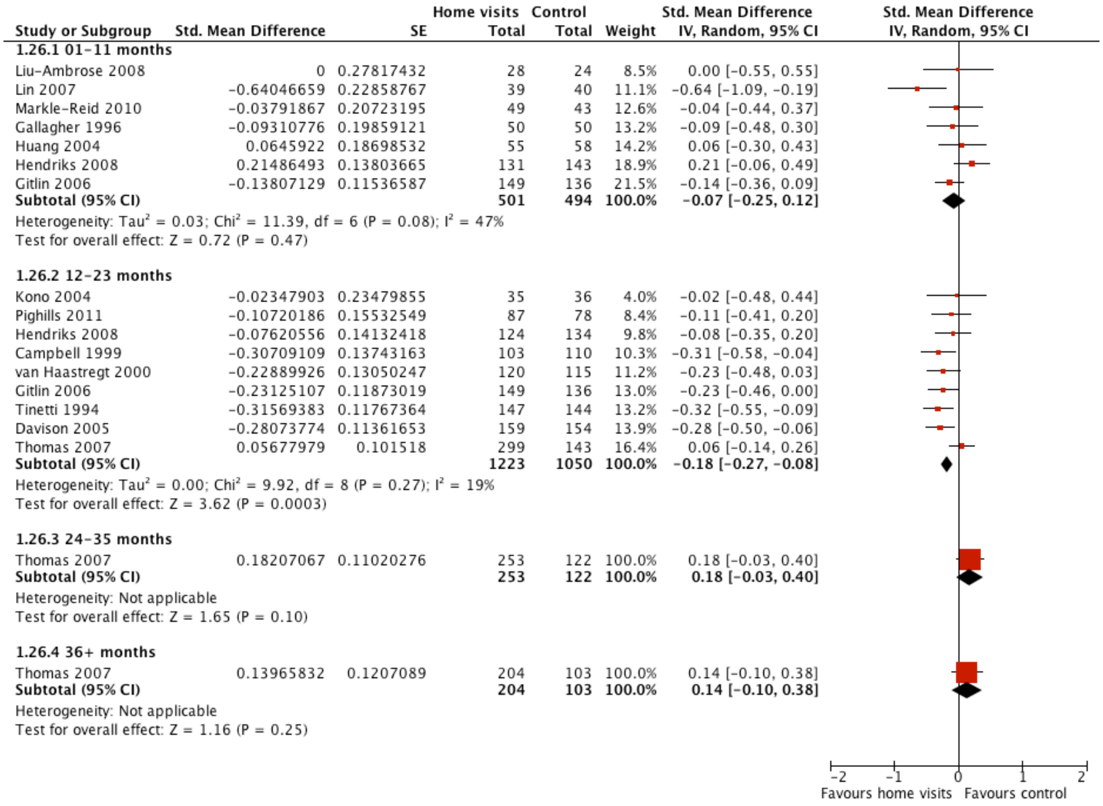

Analysis 26: Falls (subjective); focus of visit subgroups

Study or Subgroup	Std. Mean Difference	SE	Home visits Total	Control Total	Weight	Std. Mean Difference IV, Random, 95% CI
**1.27.1 Falls Only**						
Liu-Ambrose 2008	0	0.27817432	28	24	2.8%	0.00 [−0.55, 0.55]
Lin 2007	−0.64046659	0.22858767	39	40	3.9%	−0.64 [−1.09, −0.19]
Gallagher 1996	−0.09310776	0.19859121	50	50	4.9%	−0.09 [−0.48, 0.30]
Pighills 2011	−0.10720186	0.15532549	87	78	7.2%	−0.11 [−0.41, 0.20]
Campbell 1999	−0.30709109	0.13743163	103	110	8.5%	−0.31 [−0.58, −0.04]
**Subtotal (95% CI)**			**307**	**302**	**27.3%**	**−0.23 [−0.42, −0.04]**
Heterogeneity: Tau^2 = 0.01; Chi^2 = 5.32, df = 4 (P = 0.26); I^2 = 25%						
Test for overall effect: Z = 2.41 (P = 0.02)						
**1.27.2 MGA Only**						
Kono 2004	−0.02347903	0.23479855	35	36	3.7%	−0.02 [−0.48, 0.44]
Thomas 2007	0.13965832	0.1207089	204	103	10.1%	0.14 [−0.10, 0.38]
**Subtotal (95% CI)**			**239**	**139**	**13.8%**	**0.11 [−0.10, 0.32]**
Heterogeneity: Tau^2 = 0.00; Chi^2 = 0.38, df = 1 (P = 0.54); I^2 = 0%						
Test for overall effect: Z = 0.98 (P = 0.33)						
**1.27.3 Both**						
Markle-Reid 2010	−0.03791867	0.20723195	49	43	4.6%	−0.04 [−0.44, 0.37]
Huang 2004	0.0645922	0.18698532	55	58	5.4%	0.06 [−0.30, 0.43]
Hendriks 2008	−0.07620556	0.14132418	124	134	8.2%	−0.08 [−0.35, 0.20]
van Haastregt 2000	−0.22889926	0.13050247	120	115	9.1%	−0.23 [−0.48, 0.03]
Gitlin 2006	−0.23125107	0.11873019	149	136	10.3%	−0.23 [−0.46, 0.00]
Tinetti 1994	−0.31569383	0.11767364	147	144	10.4%	−0.32 [−0.55, −0.09]
Davison 2005	−0.28073774	0.11361653	159	154	10.8%	−0.28 [−0.50, −0.06]
**Subtotal (95% CI)**			**803**	**784**	**58.9%**	**−0.20 [−0.30, −0.10]**
Heterogeneity: Tau^2 = 0.00; Chi^2 = 4.97, df = 6 (P = 0.55); I^2 = 0%						
Test for overall effect: Z = 3.96 (P < 0.0001)						
**1.27.4 Neither**						
**Subtotal (95% CI)**			**0**	**0**		**Not estimable**
Heterogeneity: Not applicable						
Test for overall effect: Not applicable						
**Total (95% CI)**			**1349**	**1225**	**100.0%**	**−0.16 [−0.26, −0.07]**
Heterogeneity: Tau^2 = 0.01; Chi^2 = 18.26, df = 13 (P = 0.15); I^2 = 29%						
Test for overall effect: Z = 3.32 (P = 0.0009)						
Test for subgroup differences: Chi^2 = 7.37, df = 2 (P = 0.03), I^2 = 72.9%						

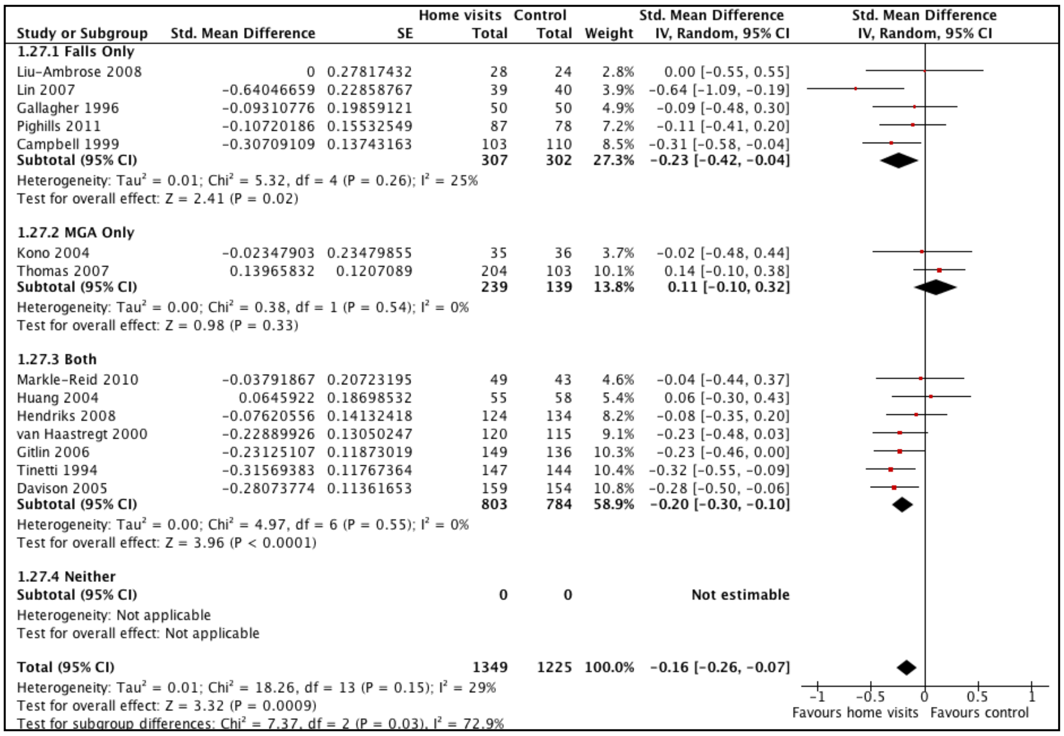

Analysis 27: Falls (subjective); age of participants subgroups

Study or Subgroup	Std. Mean Difference	SE	Home visits Total	Control Total	Weight	Std. Mean Difference IV, Random, 95% CI
**1.28.2 71-75**						
Gallagher 1996	−0.09310776	0.19859121	50	50	5.3%	−0.09 [−0.48, 0.30]
Huang 2004	0.0645922	0.18698532	55	58	5.8%	0.06 [−0.30, 0.43]
Hendriks 2008	−0.07620556	0.14132418	124	134	8.6%	−0.08 [−0.35, 0.20]
**Subtotal (95% CI)**			**229**	**242**	**19.7%**	**−0.04 [−0.23, 0.15]**
Heterogeneity: Tau^2 = 0.00; Chi^2 = 0.45, df = 2 (P = 0.80); I^2 = 0%						
Test for overall effect: Z = 0.42 (P = 0.67)						
**1.28.3 76-80**						
Lin 2007	−0.64046659	0.22858767	39	40	4.2%	−0.64 [−1.09, −0.19]
Pighills 2011	−0.10720186	0.15532549	87	78	7.6%	−0.11 [−0.41, 0.20]
van Haastregt 2000	−0.22889926	0.13050247	120	115	9.5%	−0.23 [−0.48, 0.03]
Gitlin 2006	−0.23125107	0.11873019	149	136	10.6%	−0.23 [−0.46, 0.00]
Tinetti 1994	−0.31569383	0.11767364	147	144	10.8%	−0.32 [−0.55, −0.09]
Davison 2005	−0.28073774	0.11361653	159	154	11.2%	−0.28 [−0.50, −0.06]
**Subtotal (95% CI)**			**701**	**667**	**53.9%**	**−0.27 [−0.37, −0.16]**
Heterogeneity: Tau^2 = 0.00; Chi^2 = 4.09, df = 5 (P = 0.54); I^2 = 0%						
Test for overall effect: Z = 4.94 (P < 0.00001)						
**1.28.4 81-85**						
Liu-Ambrose 2008	0	0.27817432	28	24	3.0%	0.00 [−0.55, 0.55]
Kono 2004	−0.02347903	0.23479855	35	36	4.0%	−0.02 [−0.48, 0.44]
Campbell 1999	−0.30709109	0.13743163	103	110	8.9%	−0.31 [−0.58, −0.04]
Thomas 2007	0.13965832	0.1207089	204	103	10.4%	0.14 [−0.10, 0.38]
**Subtotal (95% CI)**			**370**	**273**	**26.4%**	**−0.05 [−0.30, 0.19]**
Heterogeneity: Tau^2 = 0.03; Chi^2 = 6.01, df = 3 (P = 0.11); I^2 = 50%						
Test for overall effect: Z = 0.43 (P = 0.67)						
**Total (95% CI)**			**1300**	**1182**	**100.0%**	**−0.17 [−0.27, −0.07]**
Heterogeneity: Tau^2 = 0.01; Chi^2 = 17.85, df = 12 (P = 0.12); I^2 = 33%						
Test for overall effect: Z = 3.27 (P = 0.001)						
Test for subgroup differences: Chi^2 = 5.54, df = 2 (P = 0.06), I^2 = 63.9%						

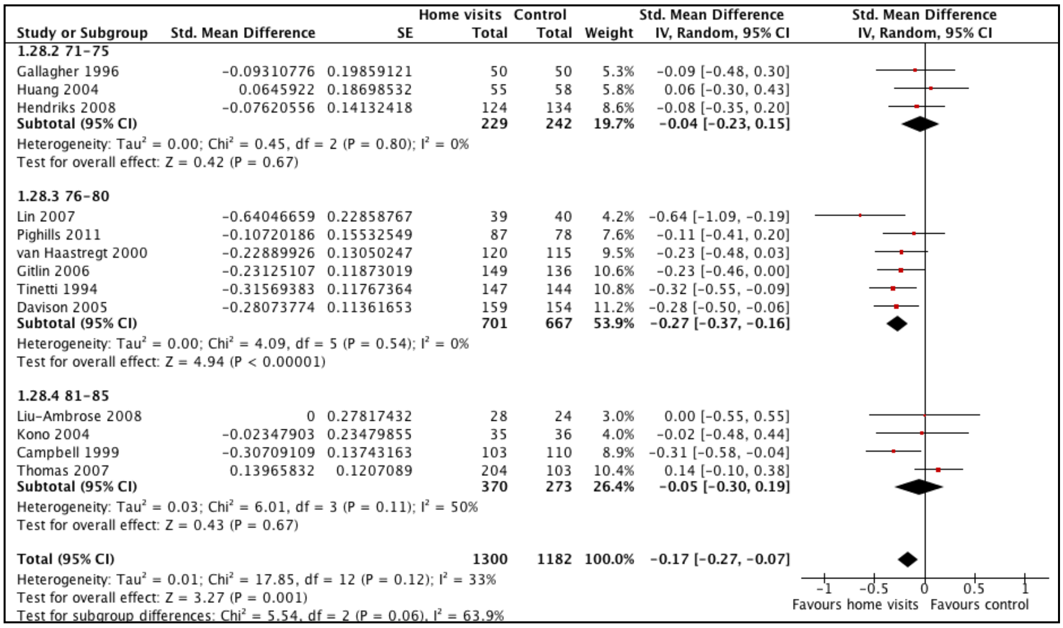

Analysis 28: Falls (subjective); type of visitor subgroups

Study or Subgroup	Std. Mean Difference	SE	Home visits Total	Control Total	Weight	Std. Mean Difference IV, Random, 95% CI
**1.29.1 Nurse**						
Kono 2004	−0.02347903	0.23479855	35	36	3.7%	−0.02 [−0.48, 0.44]
Gallagher 1996	−0.09310776	0.19859121	50	50	4.9%	−0.09 [−0.48, 0.30]
van Haastregt 2000	−0.22889926	0.13050247	120	115	9.1%	−0.23 [−0.48, 0.03]
Thomas 2007	0.13965832	0.1207089	204	103	10.1%	0.14 [−0.10, 0.38]
**Subtotal (95% CI)**			**409**	**304**	**27.9%**	**−0.04 [−0.23, 0.14]**
Heterogeneity: $Tau^2 = 0.01$; $Chi^2 = 4.39$, df = 3 (P = 0.22); $I^2 = 32\%$						
Test for overall effect: Z = 0.46 (P = 0.64)						
**1.29.2 Other**						
Liu-Ambrose 2008	0	0.27817432	28	24	2.8%	0.00 [−0.55, 0.55]
Lin 2007	−0.64046659	0.22858767	39	40	3.9%	−0.64 [−1.09, −0.19]
Huang 2004	0.0645922	0.18698532	55	58	5.4%	0.06 [−0.30, 0.43]
Pighills 2011	−0.10720186	0.15532549	87	78	7.2%	−0.11 [−0.41, 0.20]
Campbell 1999	−0.30709109	0.13743163	103	110	8.5%	−0.31 [−0.58, −0.04]
**Subtotal (95% CI)**			**312**	**310**	**27.8%**	**−0.20 [−0.42, 0.02]**
Heterogeneity: $Tau^2 = 0.03$; $Chi^2 = 7.20$, df = 4 (P = 0.13); $I^2 = 44\%$						
Test for overall effect: Z = 1.77 (P = 0.08)						
**1.29.3 Combined**						
Markle-Reid 2010	−0.03791867	0.20723195	49	43	4.6%	−0.04 [−0.44, 0.37]
Hendriks 2008	−0.07620556	0.14132418	124	134	8.2%	−0.08 [−0.35, 0.20]
Gitlin 2006	−0.23125107	0.11873019	149	136	10.3%	−0.23 [−0.46, 0.00]
Tinetti 1994	−0.31569383	0.11767364	147	144	10.4%	−0.32 [−0.55, −0.09]
Davison 2005	−0.28073774	0.11361653	159	154	10.8%	−0.28 [−0.50, −0.06]
**Subtotal (95% CI)**			**628**	**611**	**44.3%**	**−0.22 [−0.34, −0.11]**
Heterogeneity: $Tau^2 = 0.00$; $Chi^2 = 2.76$, df = 4 (P = 0.60); $I^2 = 0\%$						
Test for overall effect: Z = 3.83 (P = 0.0001)						
**Total (95% CI)**			**1349**	**1225**	**100.0%**	**−0.16 [−0.26, −0.07]**
Heterogeneity: $Tau^2 = 0.01$; $Chi^2 = 18.26$, df = 13 (P = 0.15); $I^2 = 29\%$						
Test for overall effect: Z = 3.32 (P = 0.0009)						
Test for subgroup differences: $Chi^2 = 2.53$, df = 2 (P = 0.28), $I^2 = 21.0\%$						

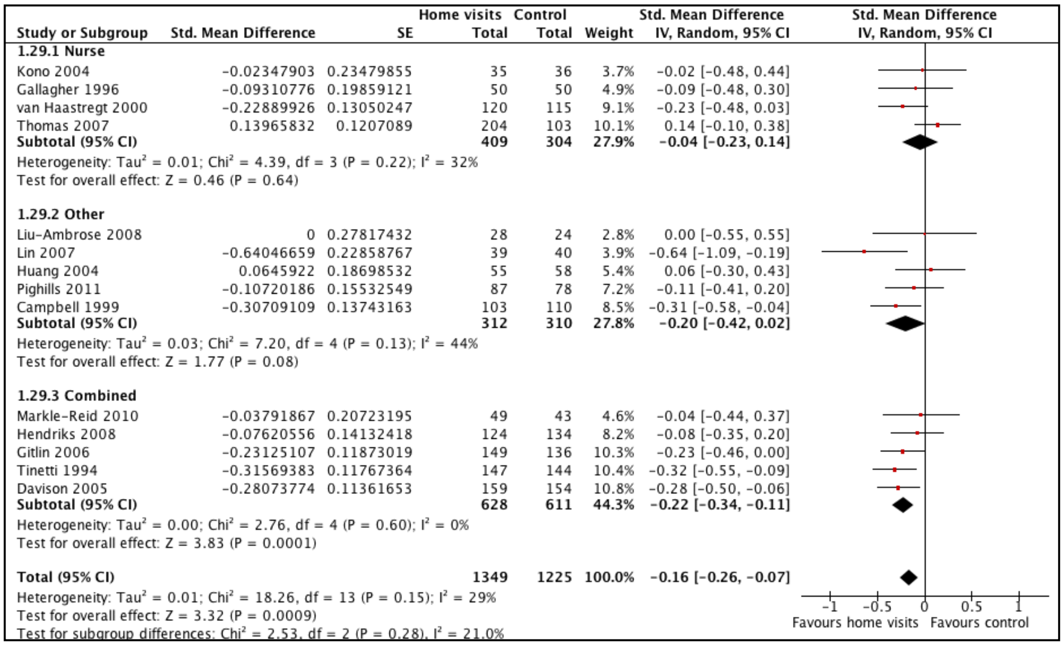

Analysis 29: Falls (subjective); number of visits subgroups

Study or Subgroup	Std. Mean Difference	SE	Home visits Total	Control Total	Weight	Std. Mean Difference IV, Random, 95% CI
**1.30.1 1 Visit**						
Pighills 2011	−0.10720186	0.15532549	87	78	8.1%	−0.11 [−0.41, 0.20]
Hendriks 2008	−0.07620556	0.14132418	124	134	9.2%	−0.08 [−0.35, 0.20]
**Subtotal (95% CI)**			**211**	**212**	**17.3%**	**−0.09 [−0.30, 0.11]**
Heterogeneity: $Tau^2 = 0.00$; $Chi^2 = 0.02$, df = 1 (P = 0.88); $I^2 = 0\%$						
Test for overall effect: Z = 0.86 (P = 0.39)						
**1.30.2 2-4 Visits**						
Kono 2004	−0.02347903	0.23479855	35	36	4.3%	−0.02 [−0.48, 0.44]
Gallagher 1996	−0.09310776	0.19859121	50	50	5.6%	−0.09 [−0.48, 0.30]
Huang 2004	0.0645922	0.18698532	55	58	6.2%	0.06 [−0.30, 0.43]
Campbell 1999	−0.30709109	0.13743163	103	110	9.5%	−0.31 [−0.58, −0.04]
Thomas 2007	0.13965832	0.1207089	204	103	11.2%	0.14 [−0.10, 0.38]
**Subtotal (95% CI)**			**447**	**357**	**36.7%**	**−0.04 [−0.23, 0.14]**
Heterogeneity: $Tau^2 = 0.02$; $Chi^2 = 6.38$, df = 4 (P = 0.17); $I^2 = 37\%$						
Test for overall effect: Z = 0.47 (P = 0.64)						
**1.30.3 5 or More Visits**						
Liu-Ambrose 2008	0	0.27817432	28	24	3.2%	0.00 [−0.55, 0.55]
Lin 2007	−0.64046659	0.22858767	39	40	4.5%	−0.64 [−1.09, −0.19]
Markle-Reid 2010	−0.03791867	0.20723195	49	43	5.2%	−0.04 [−0.44, 0.37]
van Haastregt 2000	−0.22889926	0.13050247	120	115	10.2%	−0.23 [−0.48, 0.03]
Gitlin 2006	−0.23125107	0.11873019	149	136	11.4%	−0.23 [−0.46, 0.00]
Tinetti 1994	−0.31569383	0.11767364	147	144	11.5%	−0.32 [−0.55, −0.09]
**Subtotal (95% CI)**			**532**	**502**	**46.0%**	**−0.26 [−0.38, −0.13]**
Heterogeneity: $Tau^2 = 0.00$; $Chi^2 = 5.13$, df = 5 (P = 0.40); $I^2 = 2\%$						
Test for overall effect: Z = 4.03 (P < 0.0001)						
**Total (95% CI)**			**1190**	**1071**	**100.0%**	**−0.15 [−0.25, −0.04]**
Heterogeneity: $Tau^2 = 0.01$; $Chi^2 = 17.11$, df = 12 (P = 0.15); $I^2 = 30\%$						
Test for overall effect: Z = 2.80 (P = 0.005)						
Test for subgroup differences: $Chi^2 = 4.18$, df = 2 (P = 0.12), $I^2 = 52.1\%$						

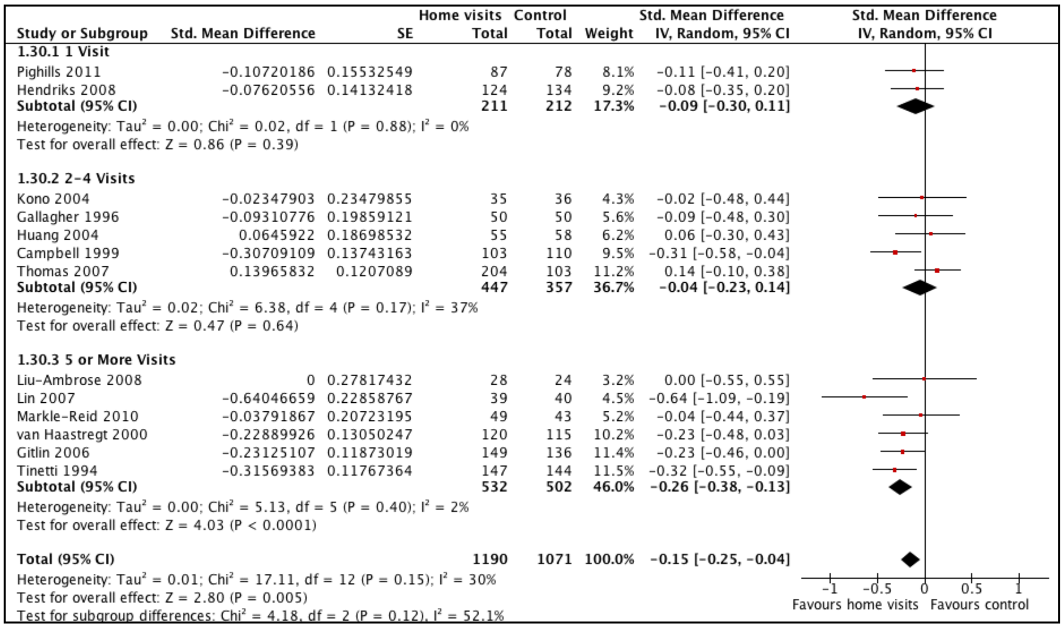

Analysis 30: Functioning ADL/IADL at each follow-up interval

Study or Subgroup	Std. Mean Difference	SE	Home visits Total	Control Total	Weight	Std. Mean Difference IV, Random, 95% CI
**1.32.1 01-11 months**						
Liu-Ambrose 2008	-0.56888253	0.27960194	28	24	8.0%	-0.57 [-1.12, -0.02]
Nelson 2004	-0.65151763	0.2435781	32	38	9.9%	-0.65 [-1.13, -0.17]
Lin 2007	-0.03271423	0.22285176	39	40	11.3%	-0.03 [-0.47, 0.40]
Hendriks 2008	0.01303871	0.12061204	131	143	22.9%	0.01 [-0.22, 0.25]
Gitlin 2006	-0.1172249	0.11532486	149	136	23.8%	-0.12 [-0.34, 0.11]
Lightbody 2002	-0.22855077	0.11297287	155	159	24.1%	-0.23 [-0.45, -0.01]
**Subtotal (95% CI)**			**534**	**540**	**100.0%**	**-0.19 [-0.37, -0.02]**
Heterogeneity: $Tau^2 = 0.02$; $Chi^2 = 9.10$, df = 5 (P = 0.11); $I^2 = 45\%$						
Test for overall effect: Z = 2.19 (P = 0.03)						
**1.32.2 12-23 months**						
Kono 2004	-0.19990984	0.22212899	43	38	2.5%	-0.20 [-0.64, 0.24]
Balaban 1988	0.1719863	0.21465724	40	46	2.7%	0.17 [-0.25, 0.59]
Newbury 2001	-0.33387362	0.21166453	45	44	2.8%	-0.33 [-0.75, 0.08]
Pighills 2011	0	0.15521335	87	78	4.4%	0.00 [-0.30, 0.30]
Bernabei 1998	-0.47892869	0.15293139	88	87	4.5%	-0.48 [-0.78, -0.18]
Luukinen 2006	0.1107394	0.14937183	144	150	4.7%	0.11 [-0.18, 0.40]
Fabacher 1994	-0.15872936	0.14316457	100	95	5.0%	-0.16 [-0.44, 0.12]
van Haastregt 2000	-0.106346	0.13025952	120	115	5.6%	-0.11 [-0.36, 0.15]
Hendriks 2008	-0.08744713	0.12431503	124	134	6.0%	-0.09 [-0.33, 0.16]
Gitlin 2006	-0.161	0.117	149	136	6.4%	-0.16 [-0.39, 0.07]
Bouman 2008	0.0184229	0.11672524	139	154	6.4%	0.02 [-0.21, 0.25]
Kono 2011	0.0271349	0.1103356	161	162	6.9%	0.03 [-0.19, 0.24]
Close 1999	-0.40541651	0.1014799	184	213	7.5%	-0.41 [-0.60, -0.21]
van Hout 2010	-0.11463482	0.09704545	215	209	7.9%	-0.11 [-0.30, 0.08]
Hebert 2001	0.0286403	0.09270249	233	231	8.2%	0.03 [-0.15, 0.21]
van Rossum 1993	0	0.08293965	292	288	9.1%	0.00 [-0.16, 0.16]
Ploeg 2010	-0.07097102	0.07914378	328	310	9.4%	-0.07 [-0.23, 0.08]
**Subtotal (95% CI)**			**2492**	**2490**	**100.0%**	**-0.10 [-0.17, -0.02]**
Heterogeneity: $Tau^2 = 0.01$; $Chi^2 = 26.87$, df = 16 (P = 0.04); $I^2 = 40\%$						
Test for overall effect: Z = 2.49 (P = 0.01)						
**1.32.3 24-35 months**						
Bouman 2008	-0.00729692	0.11669365	139	154	15.0%	-0.01 [-0.24, 0.22]
Kono 2011	0.0033795	0.11102345	161	162	16.6%	0.00 [-0.21, 0.22]
Sommers 2000	-0.15080546	0.10211874	201	183	19.6%	-0.15 [-0.35, 0.05]
Counsell 2007	0.01850388	0.06480628	474	477	48.7%	0.02 [-0.11, 0.15]
**Subtotal (95% CI)**			**975**	**976**	**100.0%**	**-0.02 [-0.11, 0.07]**
Heterogeneity: $Tau^2 = 0.00$; $Chi^2 = 2.05$, df = 3 (P = 0.56); $I^2 = 0\%$						
Test for overall effect: Z = 0.47 (P = 0.64)						
**1.32.4 36+ months**						
Stuck 1995	-0.22919786	0.11274332	170	147	18.1%	-0.23 [-0.45, -0.01]
Stuck 2000	0.1528288	0.10393887	217	459	19.1%	0.15 [-0.05, 0.36]
Vetter 1984a	-0.20375902	0.09345891	234	225	20.4%	-0.20 [-0.39, -0.02]
Vetter 1984b	0.14412128	0.09089423	254	231	20.7%	0.14 [-0.03, 0.32]
van Rossum 1993	0.04554753	0.08299354	292	288	21.7%	0.05 [-0.12, 0.21]
**Subtotal (95% CI)**			**1167**	**1350**	**100.0%**	**-0.01 [-0.17, 0.14]**
Heterogeneity: $Tau^2 = 0.02$; $Chi^2 = 13.85$, df = 4 (P = 0.008); $I^2 = 71\%$						
Test for overall effect: Z = 0.18 (P = 0.86)						

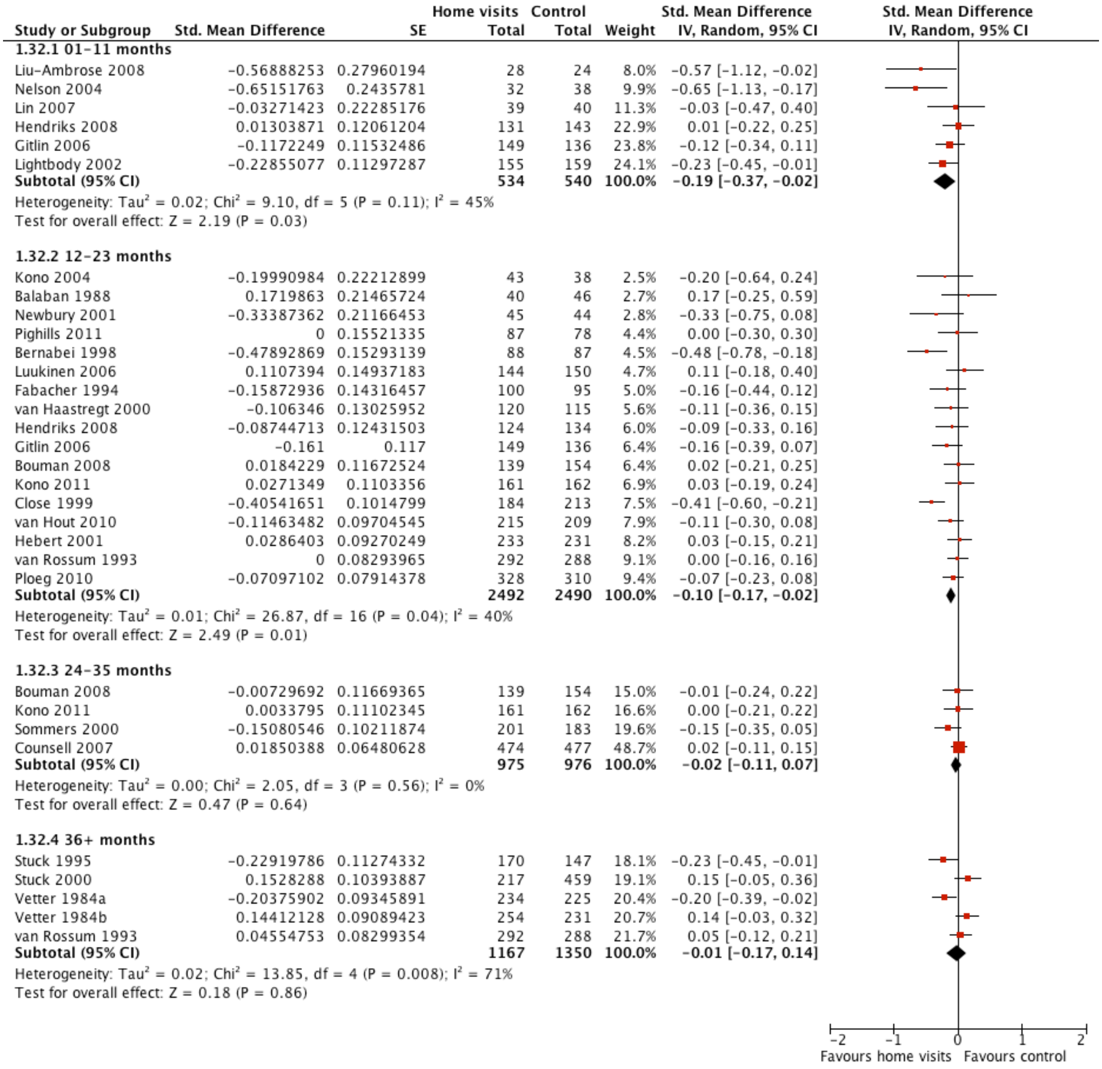

Analysis 31: Functioning ADL/IADL; focus of visit subgroups

Study or Subgroup	Std. Mean Difference	SE	Home visits Total	Control Total	Weight	Std. Mean Difference IV, Random, 95% CI
**1.33.1 Falls Only**						
Liu-Ambrose 2008	−0.56888253	0.27960194	28	24	1.2%	−0.57 [−1.12, −0.02]
Lin 2007	−0.03271423	0.22285176	39	40	1.8%	−0.03 [−0.47, 0.40]
Pighills 2011	0	0.15521335	87	78	2.9%	0.00 [−0.30, 0.30]
Luukinen 2006	0.1107394	0.14937183	144	150	3.1%	0.11 [−0.18, 0.40]
**Subtotal (95% CI)**			**298**	**292**	**9.0%**	**−0.05 [−0.28, 0.18]**
Heterogeneity: $Tau^2 = 0.02$; $Chi^2 = 4.64$, df = 3 (P = 0.20); $I^2 = 35\%$						
Test for overall effect: Z = 0.43 (P = 0.67)						
**1.33.2 MGA Only**						
Kono 2004	−0.19990984	0.22212899	43	38	1.8%	−0.20 [−0.64, 0.24]
Balaban 1988	0.1719863	0.21465724	40	46	1.9%	0.17 [−0.25, 0.59]
Bernabei 1998	−0.47892869	0.15293139	88	87	3.0%	−0.48 [−0.78, −0.18]
Bouman 2008	−0.00729692	0.11669365	139	154	4.0%	−0.01 [−0.24, 0.22]
Stuck 1995	−0.22919786	0.11274332	170	147	4.2%	−0.23 [−0.45, −0.01]
Kono 2011	0.0033795	0.11102345	161	162	4.2%	0.00 [−0.21, 0.22]
Stuck 2000	0.1528288	0.10393887	217	459	4.5%	0.15 [−0.05, 0.36]
Sommers 2000	−0.15080546	0.10211874	201	183	4.5%	−0.15 [−0.35, 0.05]
Vetter 1984a	−0.20375902	0.09345891	234	225	4.9%	−0.20 [−0.39, −0.02]
Hebert 2001	0.0286403	0.09270249	233	231	4.9%	0.03 [−0.15, 0.21]
Vetter 1984b	0.14412128	0.09089423	254	231	5.0%	0.14 [−0.03, 0.32]
van Rossum 1993	0.04554753	0.08299354	292	288	5.3%	0.05 [−0.12, 0.21]
Ploeg 2010	−0.07097102	0.07914378	328	310	5.4%	−0.07 [−0.23, 0.08]
Counsell 2007	0.01850388	0.06480628	474	477	6.0%	0.02 [−0.11, 0.15]
**Subtotal (95% CI)**			**2874**	**3038**	**59.4%**	**−0.04 [−0.12, 0.04]**
Heterogeneity: $Tau^2 = 0.01$; $Chi^2 = 27.01$, df = 13 (P = 0.01); $I^2 = 52\%$						
Test for overall effect: Z = 1.04 (P = 0.30)						
**1.33.3 Both**						
Newbury 2001	−0.33387362	0.21166453	45	44	1.9%	−0.33 [−0.75, 0.08]
Fabacher 1994	−0.15872936	0.14316457	100	95	3.2%	−0.16 [−0.44, 0.12]
van Haastregt 2000	−0.106346	0.13025952	120	115	3.6%	−0.11 [−0.36, 0.15]
Hendriks 2008	−0.08744713	0.12431503	124	134	3.8%	−0.09 [−0.33, 0.16]
Gitlin 2006	−0.161	0.117	149	136	4.0%	−0.16 [−0.39, 0.07]
Lightbody 2002	−0.22855077	0.11297287	155	159	4.1%	−0.23 [−0.45, −0.01]
Close 1999	−0.40541651	0.1014799	184	213	4.6%	−0.41 [−0.60, −0.21]
van Hout 2010	−0.11463482	0.09704545	215	209	4.7%	−0.11 [−0.30, 0.08]
**Subtotal (95% CI)**			**1092**	**1105**	**30.0%**	**−0.20 [−0.28, −0.11]**
Heterogeneity: $Tau^2 = 0.00$; $Chi^2 = 6.86$, df = 7 (P = 0.44); $I^2 = 0\%$						
Test for overall effect: Z = 4.62 (P < 0.00001)						
**1.33.4 Neither**						
Nelson 2004	−0.65151763	0.2435781	32	38	1.5%	−0.65 [−1.13, −0.17]
**Subtotal (95% CI)**			**32**	**38**	**1.5%**	**−0.65 [−1.13, −0.17]**
Heterogeneity: Not applicable						
Test for overall effect: Z = 2.67 (P = 0.007)						
**Total (95% CI)**			**4296**	**4473**	**100.0%**	**−0.10 [−0.17, −0.03]**
Heterogeneity: $Tau^2 = 0.01$; $Chi^2 = 55.40$, df = 26 (P = 0.0007); $I^2 = 53\%$						
Test for overall effect: Z = 2.92 (P = 0.004)						
Test for subgroup differences: $Chi^2 = 12.10$, df = 3 (P = 0.007), $I^2 = 75.2\%$						

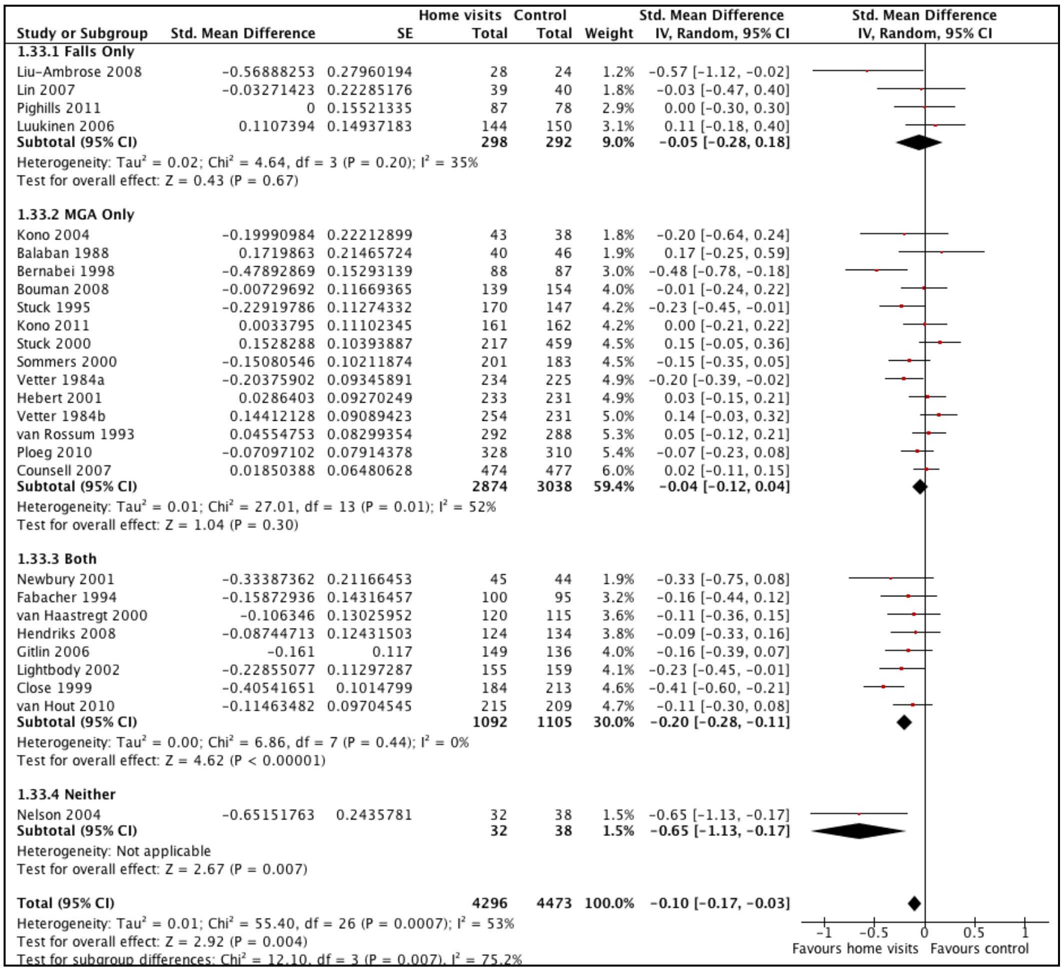

Analysis 32: Functioning ADL/IADL; age of participants subgroups

Study or Subgroup	Std. Mean Difference	SE	Home visits Total	Control Total	Weight	Std. Mean Difference IV, Random, 95% CI
**1.34.1 <=70**						
Balaban 1988	0.1719863	0.21465724	40	46	1.9%	0.17 [−0.25, 0.59]
**Subtotal (95% CI)**			**40**	**46**	**1.9%**	**0.17 [−0.25, 0.59]**
Heterogeneity: Not applicable						
Test for overall effect: Z = 0.80 (P = 0.42)						
**1.34.2 71-75**						
Fabacher 1994	−0.15872936	0.14316457	100	95	3.2%	−0.16 [−0.44, 0.12]
Hendriks 2008	−0.08744713	0.12431503	124	134	3.8%	−0.09 [−0.33, 0.16]
Lightbody 2002	−0.22855077	0.11297287	155	159	4.1%	−0.23 [−0.45, −0.01]
Counsell 2007	0.01850388	0.06480628	474	477	6.0%	0.02 [−0.11, 0.15]
**Subtotal (95% CI)**			**853**	**865**	**17.2%**	**−0.08 [−0.20, 0.04]**
Heterogeneity: Tau^2 = 0.00; Chi^2 = 4.21, df = 3 (P = 0.24); I^2 = 29%						
Test for overall effect: Z = 1.35 (P = 0.18)						
**1.34.3 76-80**						
Nelson 2004	−0.65151763	0.2435781	32	38	1.5%	−0.65 [−1.13, −0.17]
Lin 2007	−0.03271423	0.22285176	39	40	1.8%	−0.03 [−0.47, 0.40]
Newbury 2001	−0.33387362	0.21166453	45	44	1.9%	−0.33 [−0.75, 0.08]
Pighills 2011	0	0.15521335	87	78	2.9%	0.00 [−0.30, 0.30]
van Haastregt 2000	−0.106346	0.13025952	120	115	3.6%	−0.11 [−0.36, 0.15]
Gitlin 2006	−0.161	0.117	149	136	4.0%	−0.16 [−0.39, 0.07]
Bouman 2008	−0.00729692	0.11669365	139	154	4.0%	−0.01 [−0.24, 0.22]
Kono 2011	0.0033795	0.11102345	161	162	4.2%	0.00 [−0.21, 0.22]
Sommers 2000	−0.15080546	0.10211874	201	183	4.5%	−0.15 [−0.35, 0.05]
Close 1999	−0.40541651	0.1014799	184	213	4.6%	−0.41 [−0.60, −0.21]
Vetter 1984a	−0.20375902	0.09345891	234	225	4.9%	−0.20 [−0.39, −0.02]
Hebert 2001	0.0286403	0.09270249	233	231	4.9%	0.03 [−0.15, 0.21]
Vetter 1984b	0.14412128	0.09089423	254	231	5.0%	0.14 [−0.03, 0.32]
van Rossum 1993	0.04554753	0.08299354	292	288	5.3%	0.05 [−0.12, 0.21]
**Subtotal (95% CI)**			**2170**	**2138**	**53.1%**	**−0.10 [−0.20, −0.00]**
Heterogeneity: Tau^2 = 0.02; Chi^2 = 30.99, df = 13 (P = 0.003); I^2 = 58%						
Test for overall effect: Z = 2.02 (P = 0.04)						
**1.34.4 81-85**						
Liu-Ambrose 2008	−0.56888253	0.27960194	28	24	1.2%	−0.57 [−1.12, −0.02]
Kono 2004	−0.19990984	0.22212899	43	38	1.8%	−0.20 [−0.64, 0.24]
Bernabei 1998	−0.47892869	0.15293139	88	87	3.0%	−0.48 [−0.78, −0.18]
Stuck 1995	−0.22919786	0.11274332	170	147	4.2%	−0.23 [−0.45, −0.01]
Stuck 2000	0.1528288	0.10393887	217	459	4.5%	0.15 [−0.05, 0.36]
van Hout 2010	−0.11463482	0.09704545	215	209	4.7%	−0.11 [−0.30, 0.08]
Ploeg 2010	−0.07097102	0.07914378	328	310	5.4%	−0.07 [−0.23, 0.08]
**Subtotal (95% CI)**			**1089**	**1274**	**24.8%**	**−0.16 [−0.32, −0.00]**
Heterogeneity: Tau^2 = 0.03; Chi^2 = 16.42, df = 6 (P = 0.01); I^2 = 63%						
Test for overall effect: Z = 2.01 (P = 0.04)						
**1.34.5 86+**						
Luukinen 2006	0.1107394	0.14937183	144	150	3.1%	0.11 [−0.18, 0.40]
**Subtotal (95% CI)**			**144**	**150**	**3.1%**	**0.11 [−0.18, 0.40]**
Heterogeneity: Not applicable						
Test for overall effect: Z = 0.74 (P = 0.46)						
**Total (95% CI)**			**4296**	**4473**	**100.0%**	**−0.10 [−0.17, −0.03]**
Heterogeneity: Tau^2 = 0.01; Chi^2 = 55.40, df = 26 (P = 0.0007); I^2 = 53%						
Test for overall effect: Z = 2.92 (P = 0.004)						
Test for subgroup differences: Chi^2 = 4.13, df = 4 (P = 0.39), I^2 = 3.1%						

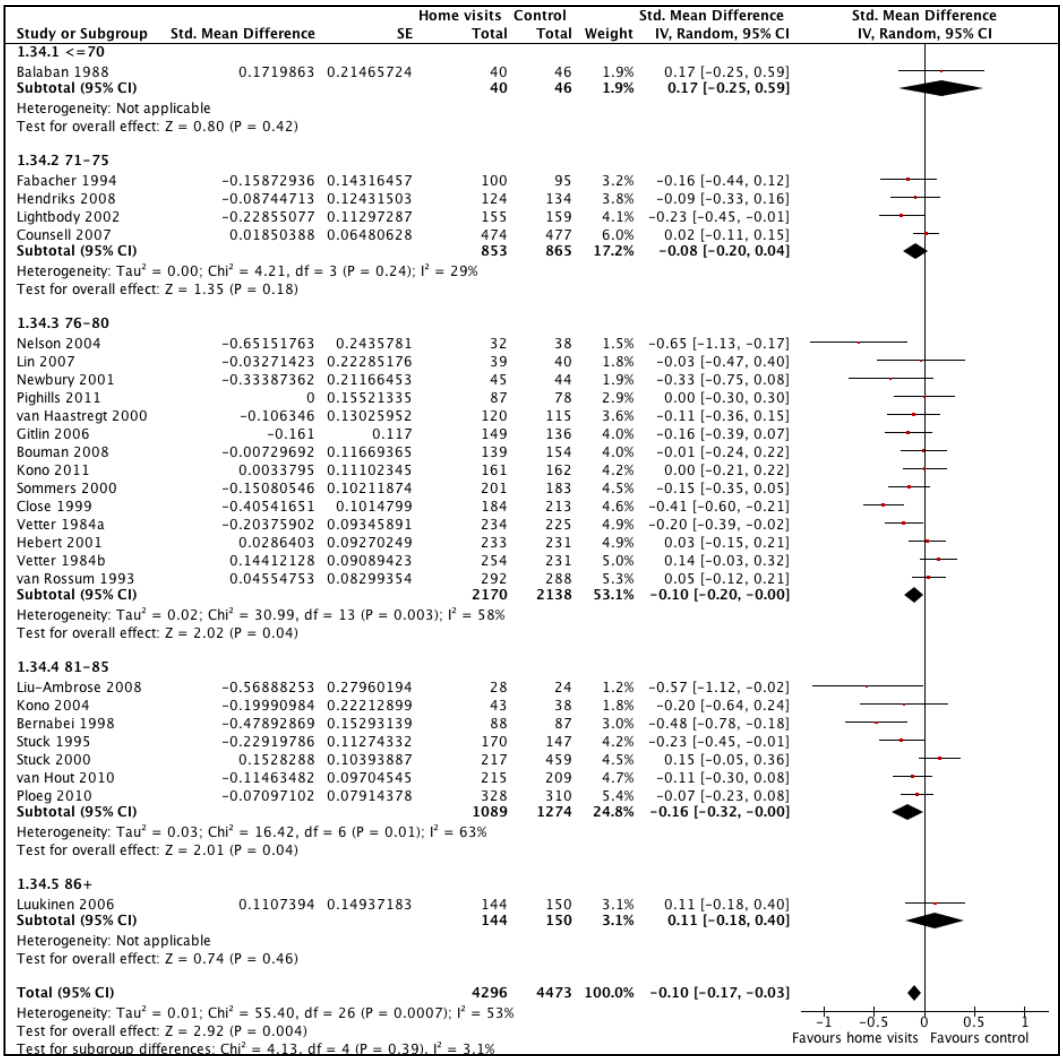

Analysis 33: Functioning ADL/IADL; type of visitor subgroups

Study or Subgroup	Std. Mean Difference	SE	Home visits Total	Control Total	Weight	Std. Mean Difference IV, Random, 95% CI
**1.35.1 Nurse**						
Kono 2004	−0.19990984	0.22212899	43	38	1.8%	−0.20 [−0.64, 0.24]
Newbury 2001	−0.33387362	0.21166453	45	44	1.9%	−0.33 [−0.75, 0.08]
van Haastregt 2000	−0.106346	0.13025952	120	115	3.6%	−0.11 [−0.36, 0.15]
Bouman 2008	−0.00729692	0.11669365	139	154	4.0%	−0.01 [−0.24, 0.22]
Lightbody 2002	−0.22855077	0.11297287	155	159	4.1%	−0.23 [−0.45, −0.01]
Stuck 1995	−0.22919786	0.11274332	170	147	4.2%	−0.23 [−0.45, −0.01]
Stuck 2000	0.1528288	0.10393887	217	459	4.5%	0.15 [−0.05, 0.36]
van Hout 2010	−0.11463482	0.09704545	215	209	4.7%	−0.11 [−0.30, 0.08]
Hebert 2001	0.0286403	0.09270249	233	231	4.9%	0.03 [−0.15, 0.21]
van Rossum 1993	0.04554753	0.08299354	292	288	5.3%	0.05 [−0.12, 0.21]
Ploeg 2010	−0.07097102	0.07914378	328	310	5.4%	−0.07 [−0.23, 0.08]
**Subtotal (95% CI)**			**1957**	**2154**	**44.4%**	**−0.06 [−0.14, 0.01]**
Heterogeneity: Tau² = 0.00; Chi² = 13.93, df = 10 (P = 0.18); I² = 28%						
Test for overall effect: Z = 1.61 (P = 0.11)						
**1.35.2 Other**						
Liu-Ambrose 2008	−0.56888253	0.27960194	28	24	1.2%	−0.57 [−1.12, −0.02]
Nelson 2004	−0.65151763	0.2435781	32	38	1.5%	−0.65 [−1.13, −0.17]
Lin 2007	−0.03271423	0.22285176	39	40	1.8%	−0.03 [−0.47, 0.40]
Pighills 2011	0	0.15521335	87	78	2.9%	0.00 [−0.30, 0.30]
Bernabei 1998	−0.47892869	0.15293139	88	87	3.0%	−0.48 [−0.78, −0.18]
Close 1999	−0.40541651	0.1014799	184	213	4.6%	−0.41 [−0.60, −0.21]
Vetter 1984a	−0.20375902	0.09345891	234	225	4.9%	−0.20 [−0.39, −0.02]
Vetter 1984b	0.14412128	0.09089423	254	231	5.0%	0.14 [−0.03, 0.32]
**Subtotal (95% CI)**			**946**	**936**	**24.9%**	**−0.24 [−0.44, −0.04]**
Heterogeneity: Tau² = 0.06; Chi² = 29.00, df = 7 (P = 0.0001); I² = 76%						
Test for overall effect: Z = 2.34 (P = 0.02)						
**1.35.3 Combined**						
Balaban 1988	0.1719863	0.21465724	40	46	1.9%	0.17 [−0.25, 0.59]
Luukinen 2006	0.1107394	0.14937183	144	150	3.1%	0.11 [−0.18, 0.40]
Fabacher 1994	−0.15872936	0.14316457	100	95	3.2%	−0.16 [−0.44, 0.12]
Hendriks 2008	−0.08744713	0.12431503	124	134	3.8%	−0.09 [−0.33, 0.16]
Gitlin 2006	−0.161	0.117	149	136	4.0%	−0.16 [−0.39, 0.07]
Kono 2011	0.0033795	0.11102345	161	162	4.2%	0.00 [−0.21, 0.22]
Sommers 2000	−0.15080546	0.10211874	201	183	4.5%	−0.15 [−0.35, 0.05]
Counsell 2007	0.01850388	0.06480628	474	477	6.0%	0.02 [−0.11, 0.15]
**Subtotal (95% CI)**			**1393**	**1383**	**30.8%**	**−0.04 [−0.12, 0.04]**
Heterogeneity: Tau² = 0.00; Chi² = 6.04, df = 7 (P = 0.53); I² = 0%						
Test for overall effect: Z = 1.02 (P = 0.31)						
**Total (95% CI)**			**4296**	**4473**	**100.0%**	**−0.10 [−0.17, −0.03]**
Heterogeneity: Tau² = 0.01; Chi² = 55.40, df = 26 (P = 0.0007); I² = 53%						
Test for overall effect: Z = 2.92 (P = 0.004)						
Test for subgroup differences: Chi² = 3.36, df = 2 (P = 0.19), I² = 40.4%						

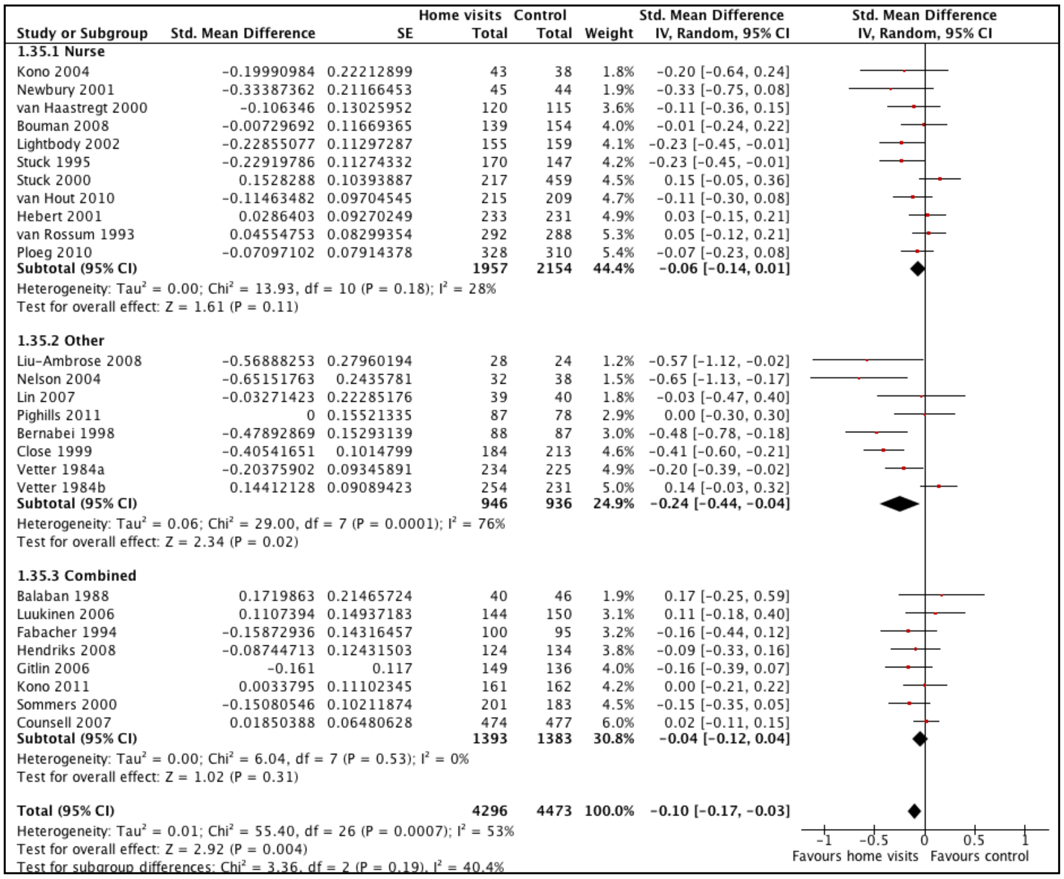

Analysis 34: Functioning ADL/IADL; number of visits subgroups

Study or Subgroup	Std. Mean Difference	SE	Home visits Total	Control Total	Weight	Std. Mean Difference IV, Random, 95% CI
**1.36.1 1 Visit**						
Newbury 2001	−0.33387362	0.21166453	45	44	2.0%	−0.33 [−0.75, 0.08]
Pighills 2011	0	0.15521335	87	78	3.0%	0.00 [−0.30, 0.30]
Hendriks 2008	−0.08744713	0.12431503	124	134	3.9%	−0.09 [−0.33, 0.16]
Lightbody 2002	−0.22855077	0.11297287	155	159	4.2%	−0.23 [−0.45, −0.01]
Close 1999	−0.40541651	0.1014799	184	213	4.6%	−0.41 [−0.60, −0.21]
Hebert 2001	0.0286403	0.09270249	233	231	5.0%	0.03 [−0.15, 0.21]
**Subtotal (95% CI)**			**828**	**859**	**22.7%**	**−0.16 [−0.32, −0.01]**
Heterogeneity: Tau² = 0.02; Chi² = 12.44, df = 5 (P = 0.03); I² = 60%						
Test for overall effect: Z = 2.04 (P = 0.04)						
**1.36.2 2-4 Visits**						
Kono 2004	−0.19990984	0.22212899	43	38	1.8%	−0.20 [−0.64, 0.24]
Luukinen 2006	0.1107394	0.14937183	144	150	3.1%	0.11 [−0.18, 0.40]
Fabacher 1994	−0.15872936	0.14316457	100	95	3.3%	−0.16 [−0.44, 0.12]
Kono 2011	0.0033795	0.11102345	161	162	4.3%	0.00 [−0.21, 0.22]
van Hout 2010	−0.11463482	0.09704545	215	209	4.8%	−0.11 [−0.30, 0.08]
Vetter 1984a	−0.20375902	0.09345891	234	225	4.9%	−0.20 [−0.39, −0.02]
Vetter 1984b	0.14412128	0.09089423	254	231	5.1%	0.14 [−0.03, 0.32]
Ploeg 2010	−0.07097102	0.07914378	328	310	5.5%	−0.07 [−0.23, 0.08]
**Subtotal (95% CI)**			**1479**	**1420**	**32.9%**	**−0.05 [−0.15, 0.04]**
Heterogeneity: Tau² = 0.01; Chi² = 10.20, df = 7 (P = 0.18); I² = 31%						
Test for overall effect: Z = 1.10 (P = 0.27)						
**1.36.3 5 or More Visits**						
Liu-Ambrose 2008	−0.56888253	0.27960194	28	24	1.3%	−0.57 [−1.12, −0.02]
Nelson 2004	−0.65151763	0.2435781	32	38	1.6%	−0.65 [−1.13, −0.17]
Lin 2007	−0.03271423	0.22285176	39	40	1.8%	−0.03 [−0.47, 0.40]
Bernabei 1998	−0.47892869	0.15293139	88	87	3.1%	−0.48 [−0.78, −0.18]
van Haastregt 2000	−0.106346	0.13025952	120	115	3.7%	−0.11 [−0.36, 0.15]
Gitlin 2006	−0.161	0.117	149	136	4.1%	−0.16 [−0.39, 0.07]
Bouman 2008	−0.00729692	0.11669365	139	154	4.1%	−0.01 [−0.24, 0.22]
Stuck 1995	−0.22919786	0.11274332	170	147	4.2%	−0.23 [−0.45, −0.01]
Stuck 2000	0.1528288	0.10393887	217	459	4.6%	0.15 [−0.05, 0.36]
Sommers 2000	−0.15080546	0.10211874	201	183	4.6%	−0.15 [−0.35, 0.05]
van Rossum 1993	0.04554753	0.08299354	292	288	5.4%	0.05 [−0.12, 0.21]
Counsell 2007	0.01850388	0.06480628	474	477	6.1%	0.02 [−0.11, 0.15]
**Subtotal (95% CI)**			**1949**	**2148**	**44.5%**	**−0.12 [−0.23, −0.01]**
Heterogeneity: Tau² = 0.02; Chi² = 28.05, df = 11 (P = 0.003); I² = 61%						
Test for overall effect: Z = 2.11 (P = 0.03)						
**Total (95% CI)**			**4256**	**4427**	**100.0%**	**−0.10 [−0.17, −0.04]**
Heterogeneity: Tau² = 0.01; Chi² = 54.02, df = 25 (P = 0.0007); I² = 54%						
Test for overall effect: Z = 3.04 (P = 0.002)						
Test for subgroup differences: Chi² = 1.73, df = 2 (P = 0.42), I² = 0%						

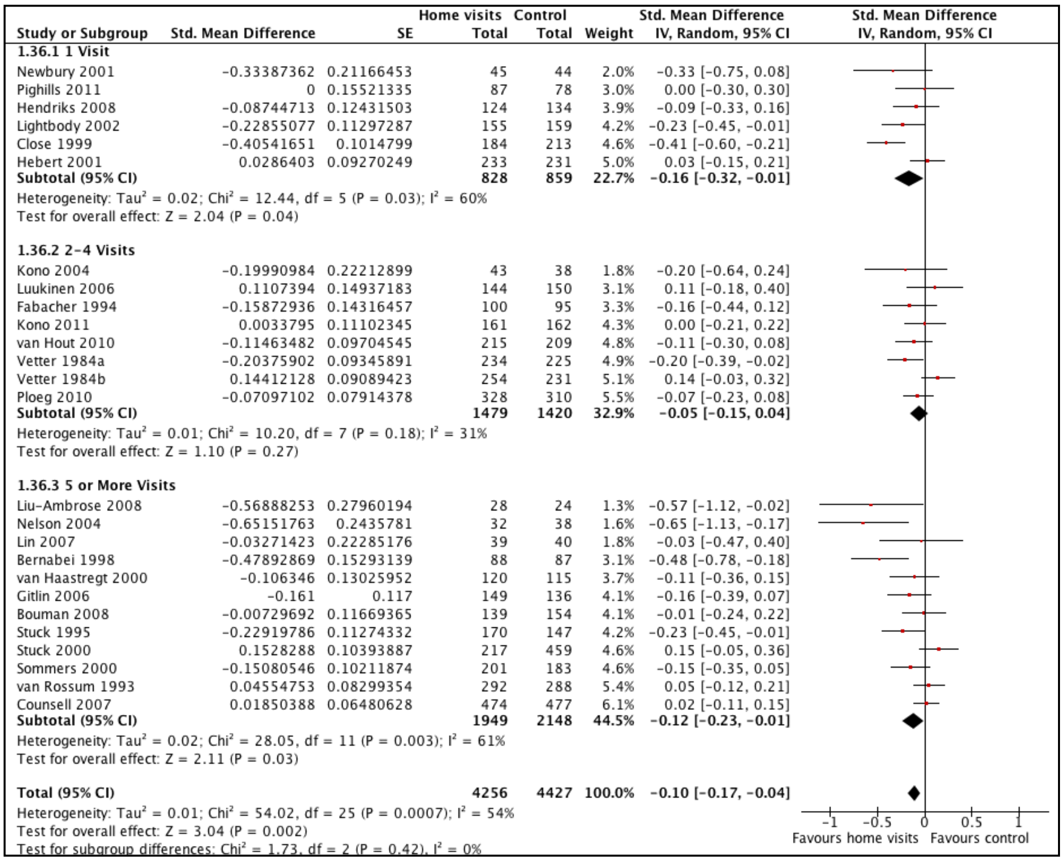

Analysis 35: Cognitive functioning, results at longest follow-up

Study or Subgroup	Std. Mean Difference	SE	Home visits Total	Control Total	Weight	Std. Mean Difference IV, Random, 95% CI
Newbury 2001	−0.46767489	0.3253822	45	44	4.5%	−0.47 [−1.11, 0.17]
Liu-Ambrose 2008	−0.34338806	0.27646923	28	24	5.9%	−0.34 [−0.89, 0.20]
Markle-Reid 2010	−0.1247856	0.21554978	45	40	8.7%	−0.12 [−0.55, 0.30]
Bernabei 1998	−0.31937041	0.15149675	88	87	13.6%	−0.32 [−0.62, −0.02]
Luukinen 2006	−0.12372351	0.14274132	144	150	14.5%	−0.12 [−0.40, 0.16]
Thomas 2007	0	0.12057725	204	103	17.1%	0.00 [−0.24, 0.24]
Bouman 2008	0.15832071	0.116876	139	154	17.6%	0.16 [−0.07, 0.39]
Davison 2005	0.14794968	0.11294314	159	154	18.1%	0.15 [−0.07, 0.37]
**Total (95% CI)**			**852**	**756**	**100.0%**	**−0.06 [−0.21, 0.09]**

Heterogeneity: Tau² = 0.02; Chi² = 12.49, df = 7 (P = 0.09); I² = 44%
Test for overall effect: Z = 0.79 (P = 0.43)

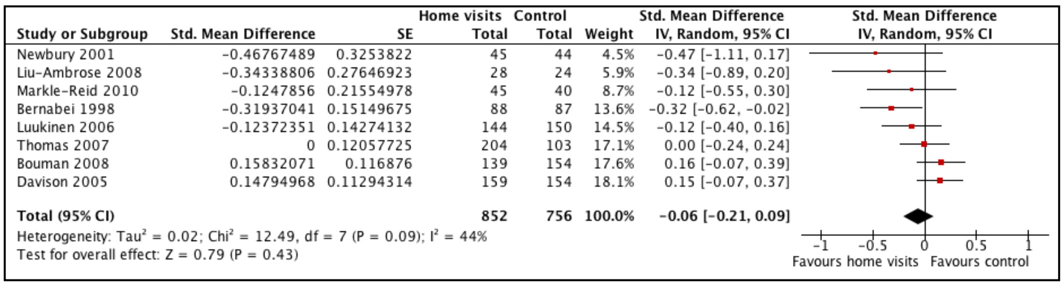

Analysis 36: Quality of life at each follow-up interval

Study or Subgroup	Std. Mean Difference	SE	Home visits Total	Control Total	Weight	Std. Mean Difference IV, Random, 95% CI
**1.44.1 01-11 months**						
Crawford Shearer 2010	−0.15973573	0.30370371	20	22	3.5%	−0.16 [−0.75, 0.44]
Lin 2007	−0.68159697	0.22962498	39	40	5.6%	−0.68 [−1.13, −0.23]
Kingston 2001	0.11056703	0.20878628	51	41	6.5%	0.11 [−0.30, 0.52]
Markle-Reid 2010	0.05052332	0.2072482	49	43	6.6%	0.05 [−0.36, 0.46]
Lenaghan 2007	0.29405032	0.20177338	50	49	6.9%	0.29 [−0.10, 0.69]
Gallagher 1996	−0.11156514	0.1986222	50	50	7.1%	−0.11 [−0.50, 0.28]
Hendriks 2008	0.0032602	0.13291559	131	143	12.5%	0.00 [−0.26, 0.26]
Markle-Reid 2006	−0.04768252	0.12860815	120	121	13.0%	−0.05 [−0.30, 0.20]
van Hout 2010	−0.00521267	0.09189674	244	229	18.4%	−0.01 [−0.19, 0.17]
Holland 2005	−0.16162187	0.08257294	307	282	20.0%	−0.16 [−0.32, 0.00]
**Subtotal (95% CI)**			**1061**	**1020**	**100.0%**	**−0.06 [−0.18, 0.06]**
Heterogeneity: Tau² = 0.01; Chi² = 13.62, df = 9 (P = 0.14); I² = 34%						
Test for overall effect: Z = 1.00 (P = 0.32)						
**1.44.2 12-23 months**						
Shapiro 2002	−0.4934019	0.28080567	32	21	1.2%	−0.49 [−1.04, 0.06]
Newbury 2001	−0.07936562	0.21500223	45	43	2.1%	−0.08 [−0.50, 0.34]
Balaban 1988	0.16224858	0.2146303	40	46	2.1%	0.16 [−0.26, 0.58]
Luukinen 2006	0.13024238	0.16854423	144	150	3.4%	0.13 [−0.20, 0.46]
Pighills 2011	−0.00959988	0.15525892	87	78	4.0%	−0.01 [−0.31, 0.29]
Hendriks 2008	−0.05194913	0.13624672	124	134	5.2%	−0.05 [−0.32, 0.22]
van Haastregt 2000	−0.01953552	0.13007778	120	115	5.7%	−0.02 [−0.27, 0.24]
Bouman 2008	0.01359048	0.11680754	139	154	7.0%	0.01 [−0.22, 0.24]
Yamada 2003	−0.10274656	0.11364501	160	149	7.4%	−0.10 [−0.33, 0.12]
Ploeg 2010	−0.10764642	0.10764642	328	310	8.3%	−0.11 [−0.32, 0.10]
Sommers 2000	−0.17900004	0.10217801	201	183	9.2%	−0.18 [−0.38, 0.02]
Thomas 2007	−0.10686591	0.10156366	299	143	9.3%	−0.11 [−0.31, 0.09]
van Hout 2010	0.13887444	0.09708599	215	209	10.2%	0.14 [−0.05, 0.33]
Hebert 2001	0.03384274	0.09270438	233	231	11.2%	0.03 [−0.15, 0.22]
van Rossum 1993	−0.04554753	0.08295043	292	288	13.9%	−0.05 [−0.21, 0.12]
**Subtotal (95% CI)**			**2459**	**2254**	**100.0%**	**−0.03 [−0.10, 0.03]**
Heterogeneity: Tau² = 0.00; Chi² = 11.82, df = 14 (P = 0.62); I² = 0%						
Test for overall effect: Z = 1.11 (P = 0.27)						
**1.44.3 24-35 months**						
Bouman 2008	−0.05872679	0.11674329	139	154	8.4%	−0.06 [−0.29, 0.17]
Thomas 2007	−0.09979879	0.11006239	253	122	9.3%	−0.10 [−0.32, 0.12]
Vetter 1984a	−0.10632843	0.09988346	201	199	10.8%	−0.11 [−0.30, 0.09]
Vetter 1984b	−0.16945091	0.09477957	237	210	11.8%	−0.17 [−0.36, 0.02]
Sorensen 1988	−0.06084525	0.07678877	315	366	16.1%	−0.06 [−0.21, 0.09]
Counsell 2007	0.09213862	0.06485754	474	477	20.1%	0.09 [−0.03, 0.22]
Byles 2004	−0.11914883	0.05635457	779	529	23.7%	−0.12 [−0.23, −0.01]
**Subtotal (95% CI)**			**2398**	**2057**	**100.0%**	**−0.07 [−0.14, 0.01]**
Heterogeneity: Tau² = 0.00; Chi² = 8.28, df = 6 (P = 0.22); I² = 28%						
Test for overall effect: Z = 1.77 (P = 0.08)						
**1.44.4 36+ months**						
Thomas 2007	−0.05336521	0.12059648	204	103	11.9%	−0.05 [−0.29, 0.18]
Pathy 1992	−0.17854933	0.09792811	223	196	18.0%	−0.18 [−0.37, 0.01]
van Rossum 1993	−0.04554753	0.08295043	292	288	25.1%	−0.05 [−0.21, 0.12]
Byles 2004	−0.14951835	0.06180403	636	446	45.1%	−0.15 [−0.27, −0.03]
**Subtotal (95% CI)**			**1355**	**1033**	**100.0%**	**−0.12 [−0.20, −0.04]**
Heterogeneity: Tau² = 0.00; Chi² = 1.69, df = 3 (P = 0.64); I² = 0%						
Test for overall effect: Z = 2.83 (P = 0.005)						

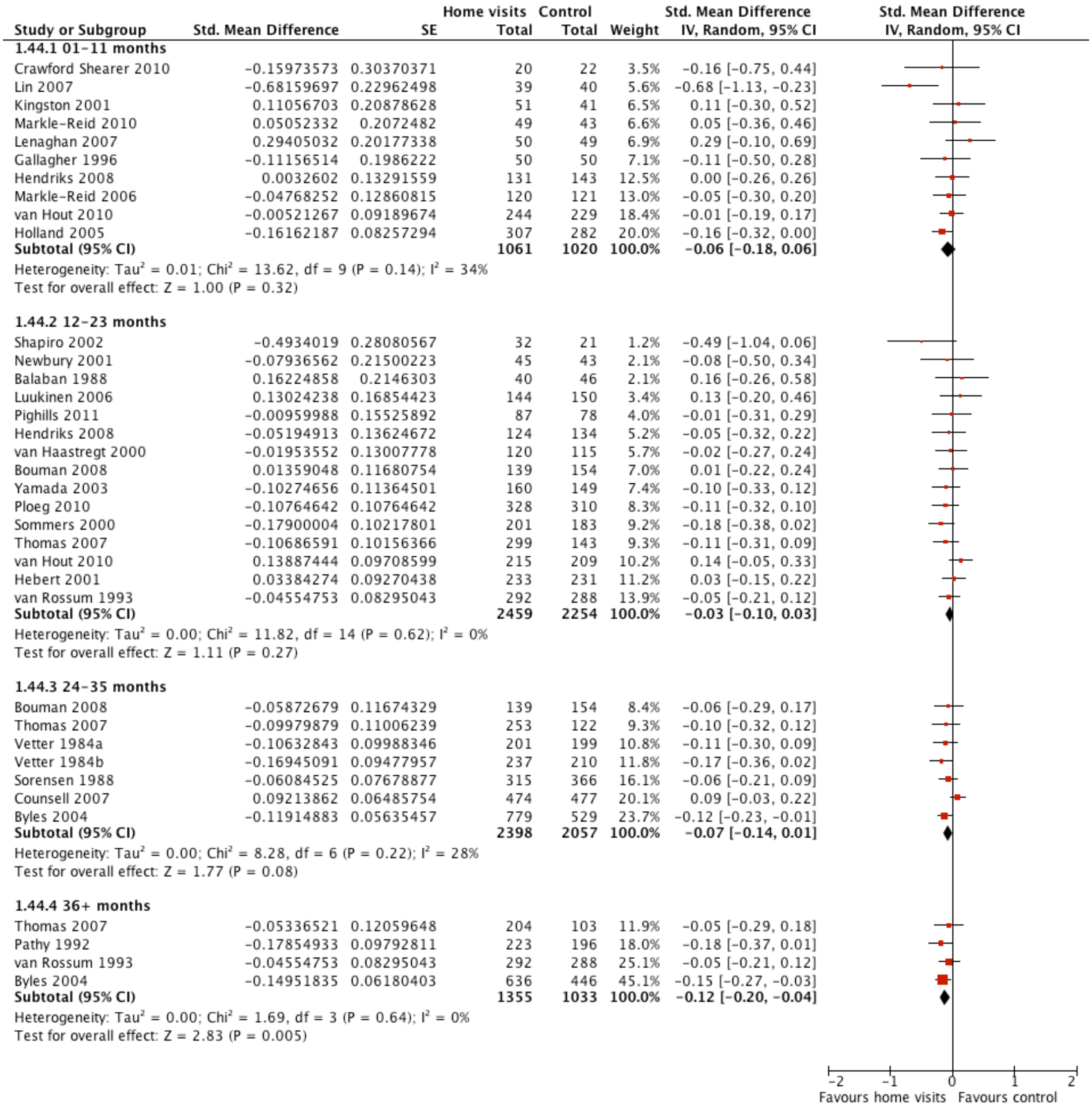

Analysis 37: Quality of life; focus of visit subgroups

Study or Subgroup	Std. Mean Difference	SE	Home visits Total	Control Total	Weight	Std. Mean Difference IV, Random, 95% CI
**1.45.1 Falls Only**						
Lin 2007	−0.68159697	0.22962498	39	40	1.1%	−0.68 [−1.13, −0.23]
Kingston 2001	0.11056703	0.20878628	51	41	1.3%	0.11 [−0.30, 0.52]
Gallagher 1996	−0.11156514	0.1986222	50	50	1.4%	−0.11 [−0.50, 0.28]
Luukinen 2006	0.13024238	0.16854423	144	150	1.9%	0.13 [−0.20, 0.46]
Pighills 2011	−0.00959988	0.15525892	87	78	2.1%	−0.01 [−0.31, 0.29]
**Subtotal (95% CI)**			**371**	**359**	**7.7%**	**−0.09 [−0.34, 0.17]**
Heterogeneity: Tau² = 0.05; Chi² = 9.44, df = 4 (P = 0.05); I² = 58%						
Test for overall effect: Z = 0.68 (P = 0.49)						
**1.45.2 MGA Only**						
Shapiro 2002	−0.4934019	0.28080567	32	21	0.7%	−0.49 [−1.04, 0.06]
Balaban 1988	0.16224858	0.2146303	40	46	1.2%	0.16 [−0.26, 0.58]
Markle-Reid 2006	−0.04768252	0.12860815	120	121	3.0%	−0.05 [−0.30, 0.20]
Thomas 2007	−0.05336521	0.12059648	204	103	3.3%	−0.05 [−0.29, 0.18]
Bouman 2008	−0.05872679	0.11674329	139	154	3.5%	−0.06 [−0.29, 0.17]
Sommers 2000	−0.17900004	0.10217801	201	183	4.3%	−0.18 [−0.38, 0.02]
Vetter 1984a	−0.10632843	0.09988346	201	199	4.4%	−0.11 [−0.30, 0.09]
Pathy 1992	−0.17854933	0.09792811	223	196	4.5%	−0.18 [−0.37, 0.01]
Vetter 1984b	−0.16945091	0.09477957	237	210	4.8%	−0.17 [−0.36, 0.02]
Hebert 2001	0.03384274	0.09270438	233	231	4.9%	0.03 [−0.15, 0.22]
van Rossum 1993	−0.04554753	0.08295043	292	288	5.7%	−0.05 [−0.21, 0.12]
Ploeg 2010	−0.10764642	0.08215403	328	310	5.8%	−0.11 [−0.27, 0.05]
Sorensen 1988	−0.06084525	0.07678877	315	366	6.3%	−0.06 [−0.21, 0.09]
Counsell 2007	0.09213862	0.06485754	474	477	7.8%	0.09 [−0.03, 0.22]
Byles 2004	−0.14951835	0.06180403	636	446	8.2%	−0.15 [−0.27, −0.03]
**Subtotal (95% CI)**			**3675**	**3351**	**68.3%**	**−0.07 [−0.13, −0.02]**
Heterogeneity: Tau² = 0.00; Chi² = 16.56, df = 14 (P = 0.28); I² = 15%						
Test for overall effect: Z = 2.76 (P = 0.006)						
**1.45.3 Both**						
Newbury 2001	−0.07936562	0.21500223	45	43	1.2%	−0.08 [−0.50, 0.34]
Markle-Reid 2010	0.05052332	0.2072482	49	43	1.3%	0.05 [−0.36, 0.46]
Hendriks 2008	−0.05194913	0.13624672	124	134	2.7%	−0.05 [−0.32, 0.22]
van Haastregt 2000	−0.01953552	0.13007778	120	115	2.9%	−0.02 [−0.27, 0.24]
Yamada 2003	−0.10274656	0.11364501	160	149	3.6%	−0.10 [−0.33, 0.12]
van Hout 2010	0.13887444	0.09708599	215	209	4.6%	0.14 [−0.05, 0.33]
**Subtotal (95% CI)**			**713**	**693**	**16.3%**	**0.01 [−0.10, 0.11]**
Heterogeneity: Tau² = 0.00; Chi² = 3.21, df = 5 (P = 0.67); I² = 0%						
Test for overall effect: Z = 0.12 (P = 0.90)						
**1.45.4 Neither**						
Crawford Shearer 2010	−0.15973573	0.30370371	20	22	0.6%	−0.16 [−0.75, 0.44]
Lenaghan 2007	0.29405032	0.20177338	50	49	1.3%	0.29 [−0.10, 0.69]
Holland 2005	−0.16162187	0.08257294	307	282	5.8%	−0.16 [−0.32, 0.00]
**Subtotal (95% CI)**			**377**	**353**	**7.7%**	**−0.02 [−0.33, 0.28]**
Heterogeneity: Tau² = 0.04; Chi² = 4.41, df = 2 (P = 0.11); I² = 55%						
Test for overall effect: Z = 0.16 (P = 0.87)						
**Total (95% CI)**			**5136**	**4756**	**100.0%**	**−0.06 [−0.11, −0.01]**
Heterogeneity: Tau² = 0.00; Chi² = 35.69, df = 28 (P = 0.15); I² = 22%						
Test for overall effect: Z = 2.57 (P = 0.01)						
Test for subgroup differences: Chi² = 1.89, df = 3 (P = 0.60), I² = 0%						

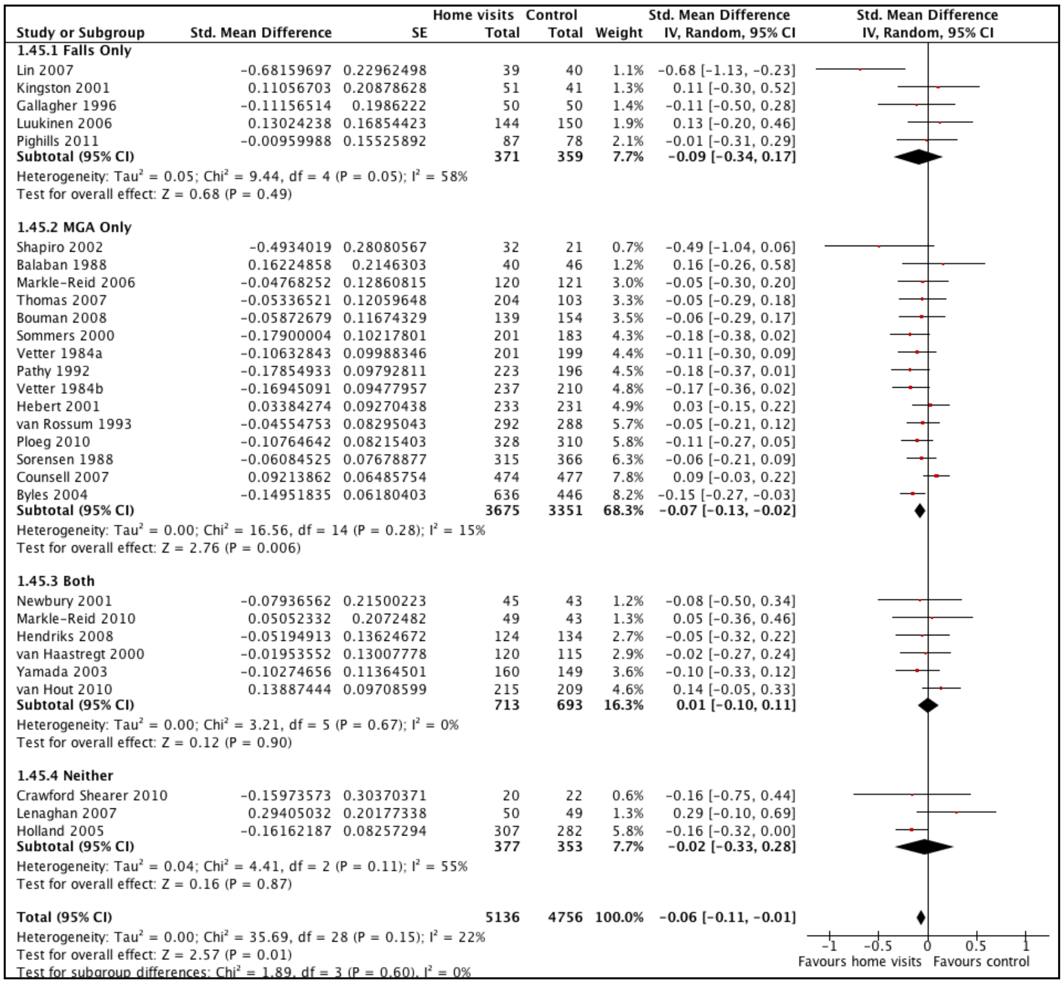

Analysis 38: Quality of life; age of participants subgroups

Study or Subgroup	Std. Mean Difference	SE	Home visits Total	Control Total	Weight	Std. Mean Difference IV, Random, 95% CI
**1.46.1 <=70**						
Balaban 1988	0.16224858	0.2146303	40	46	1.2%	0.16 [−0.26, 0.58]
**Subtotal (95% CI)**			**40**	**46**	**1.2%**	**0.16 [−0.26, 0.58]**
Heterogeneity: Not applicable						
Test for overall effect: Z = 0.76 (P = 0.45)						
**1.46.2 71-75**						
Kingston 2001	0.11056703	0.20878628	51	41	1.3%	0.11 [−0.30, 0.52]
Gallagher 1996	−0.11156514	0.1986222	50	50	1.4%	−0.11 [−0.50, 0.28]
Hendriks 2008	0.03748253	0.12425373	124	134	3.2%	0.04 [−0.21, 0.28]
Pathy 1992	−0.17854933	0.09792811	223	196	4.6%	−0.18 [−0.37, 0.01]
Counsell 2007	0.09213862	0.06485754	474	477	7.6%	0.09 [−0.03, 0.22]
**Subtotal (95% CI)**			**922**	**898**	**18.1%**	**−0.00 [−0.13, 0.12]**
Heterogeneity: Tau² = 0.01; Chi² = 5.96, df = 4 (P = 0.20); I² = 33%						
Test for overall effect: Z = 0.07 (P = 0.95)						
**1.46.3 76-80**						
Crawford Shearer 2010	−0.15973573	0.30370371	20	22	0.6%	−0.16 [−0.75, 0.44]
Shapiro 2002	−0.4934019	0.28080567	32	21	0.8%	−0.49 [−1.04, 0.06]
Lin 2007	−0.68159697	0.22962498	39	40	1.1%	−0.68 [−1.13, −0.23]
Newbury 2001	−0.07936562	0.21500223	45	43	1.2%	−0.08 [−0.50, 0.34]
Pighills 2011	−0.00959988	0.15525892	87	78	2.2%	−0.01 [−0.31, 0.29]
van Haastregt 2000	−0.01953552	0.13007778	120	115	3.0%	−0.02 [−0.27, 0.24]
Bouman 2008	−0.05872679	0.11674329	139	154	3.5%	−0.06 [−0.29, 0.17]
Yamada 2003	−0.10274656	0.11364501	160	149	3.7%	−0.10 [−0.33, 0.12]
Sommers 2000	−0.17900004	0.10217801	201	183	4.3%	−0.18 [−0.38, 0.02]
Vetter 1984a	−0.10632843	0.09988346	201	199	4.5%	−0.11 [−0.30, 0.09]
Vetter 1984b	−0.16945091	0.09477957	237	210	4.8%	−0.17 [−0.36, 0.02]
Hebert 2001	0.03384274	0.09270438	233	231	5.0%	0.03 [−0.15, 0.22]
van Rossum 1993	−0.04554753	0.08295043	292	288	5.7%	−0.05 [−0.21, 0.12]
Sorensen 1988	−0.06084525	0.07678877	315	366	6.3%	−0.06 [−0.21, 0.09]
Byles 2004	−0.14951835	0.06180403	636	446	8.0%	−0.15 [−0.27, −0.03]
**Subtotal (95% CI)**			**2757**	**2545**	**54.7%**	**−0.10 [−0.16, −0.05]**
Heterogeneity: Tau² = 0.00; Chi² = 13.82, df = 14 (P = 0.46); I² = 0%						
Test for overall effect: Z = 3.73 (P = 0.0002)						
**1.46.4 81-85**						
Lenaghan 2007	0.29405032	0.20177338	50	49	1.4%	0.29 [−0.10, 0.69]
Markle-Reid 2006	−0.04768252	0.12860815	120	121	3.0%	−0.05 [−0.30, 0.20]
Thomas 2007	−0.05336521	0.12059648	204	103	3.4%	−0.05 [−0.29, 0.18]
van Hout 2010	0.13887444	0.09708599	215	209	4.6%	0.14 [−0.05, 0.33]
Holland 2005	−0.16162187	0.08257294	307	282	5.8%	−0.16 [−0.32, 0.00]
Ploeg 2010	−0.10764642	0.08215403	328	310	5.8%	−0.11 [−0.27, 0.05]
**Subtotal (95% CI)**			**1224**	**1074**	**24.0%**	**−0.03 [−0.14, 0.09]**
Heterogeneity: Tau² = 0.01; Chi² = 8.99, df = 5 (P = 0.11); I² = 44%						
Test for overall effect: Z = 0.46 (P = 0.65)						
**1.46.5 86+**						
Luukinen 2006	0.13024238	0.16854423	144	150	1.9%	0.13 [−0.20, 0.46]
**Subtotal (95% CI)**			**144**	**150**	**1.9%**	**0.13 [−0.20, 0.46]**
Heterogeneity: Not applicable						
Test for overall effect: Z = 0.77 (P = 0.44)						
**Total (95% CI)**			**5087**	**4713**	**100.0%**	**−0.06 [−0.11, −0.01]**
Heterogeneity: Tau² = 0.00; Chi² = 36.03, df = 27 (P = 0.11); I² = 25%						
Test for overall effect: Z = 2.44 (P = 0.01)						
Test for subgroup differences: Chi² = 5.54, df = 4 (P = 0.24), I² = 27.9%						

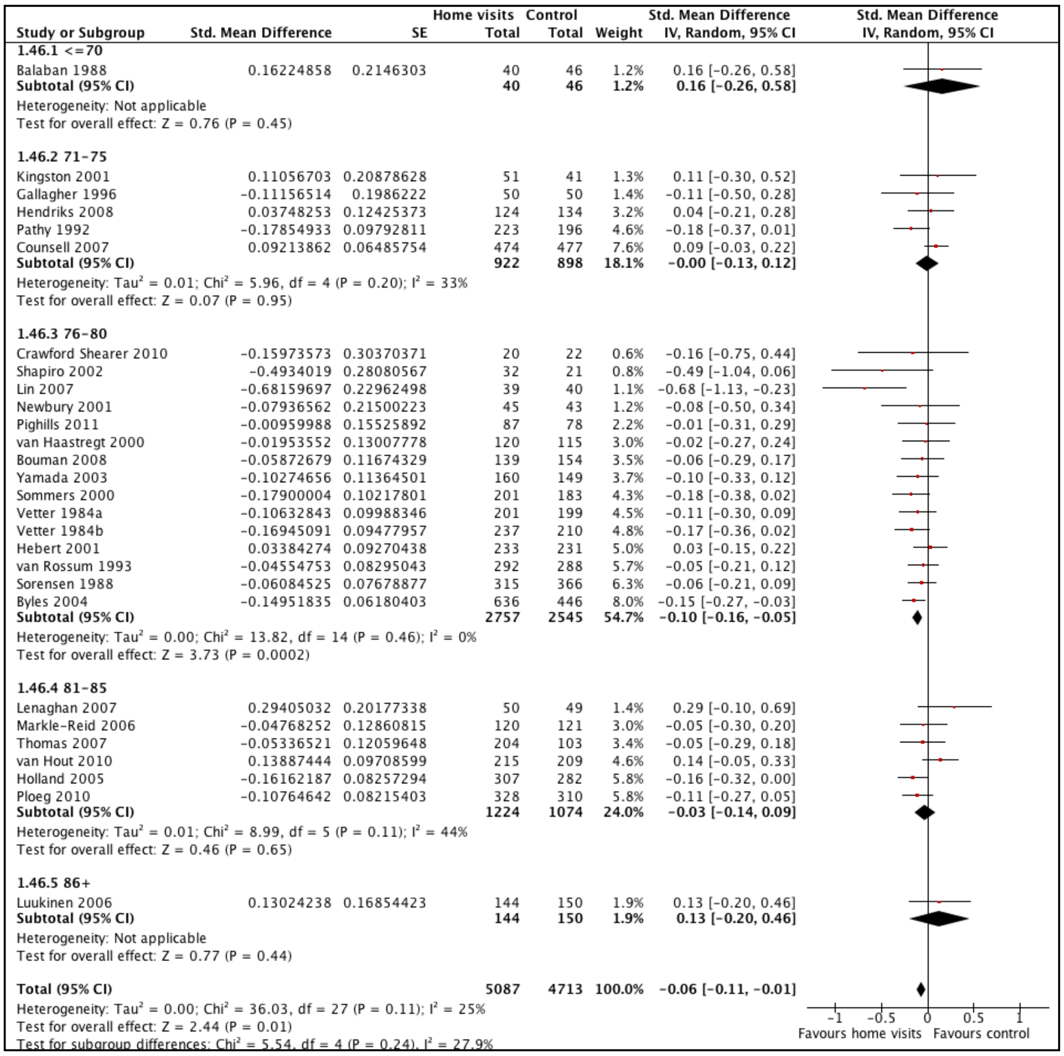

Analysis 39: Quality of life; type of visitor subgroups

Study or Subgroup	Std. Mean Difference	SE	Home visits Total	Control Total	Weight	Std. Mean Difference IV, Random, 95% CI
**1.47.1 Nurse**						
Crawford Shearer 2010	−0.15973573	0.30370371	20	22	0.6%	−0.16 [−0.75, 0.44]
Shapiro 2002	−0.4934019	0.28080567	32	21	0.7%	−0.49 [−1.04, 0.06]
Newbury 2001	−0.07936562	0.21500223	45	43	1.2%	−0.08 [−0.50, 0.34]
Gallagher 1996	−0.11156514	0.1986222	50	50	1.4%	−0.11 [−0.50, 0.28]
van Haastregt 2000	−0.01953552	0.13007778	120	115	2.9%	−0.02 [−0.27, 0.24]
Markle-Reid 2006	−0.04768252	0.12860815	120	121	3.0%	−0.05 [−0.30, 0.20]
Thomas 2007	−0.05336521	0.12059648	204	103	3.3%	−0.05 [−0.29, 0.18]
Bouman 2008	−0.05872679	0.11674329	139	154	3.5%	−0.06 [−0.29, 0.17]
Yamada 2003	−0.10274656	0.11364501	160	149	3.6%	−0.10 [−0.33, 0.12]
van Hout 2010	0.13887444	0.09708599	215	209	4.6%	0.14 [−0.05, 0.33]
Hebert 2001	0.03384274	0.09270438	233	231	4.9%	0.03 [−0.15, 0.22]
van Rossum 1993	−0.04554753	0.08295043	292	288	5.7%	−0.05 [−0.21, 0.12]
Ploeg 2010	−0.10764642	0.08215403	328	310	5.8%	−0.11 [−0.27, 0.05]
**Subtotal (95% CI)**			**1958**	**1816**	**41.2%**	**−0.04 [−0.10, 0.03]**
Heterogeneity: Tau² = 0.00; Chi² = 8.01, df = 12 (P = 0.78); I² = 0%						
Test for overall effect: Z = 1.20 (P = 0.23)						
**1.47.2 Other**						
Lin 2007	−0.68159697	0.22962498	39	40	1.1%	−0.68 [−1.13, −0.23]
Kingston 2001	0.11056703	0.20878628	51	41	1.3%	0.11 [−0.30, 0.52]
Lenaghan 2007	0.29405032	0.20177338	50	49	1.3%	0.29 [−0.10, 0.69]
Pighills 2011	−0.00959988	0.15525892	87	78	2.1%	−0.01 [−0.31, 0.29]
Vetter 1984a	−0.10632843	0.09988346	201	199	4.4%	−0.11 [−0.30, 0.09]
Pathy 1992	−0.17854933	0.09792811	223	196	4.5%	−0.18 [−0.37, 0.01]
Vetter 1984b	−0.16945091	0.09477957	237	210	4.8%	−0.17 [−0.36, 0.02]
Holland 2005	−0.16162187	0.08257294	307	282	5.8%	−0.16 [−0.32, 0.00]
**Subtotal (95% CI)**			**1195**	**1095**	**25.3%**	**−0.12 [−0.24, −0.00]**
Heterogeneity: Tau² = 0.01; Chi² = 12.74, df = 7 (P = 0.08); I² = 45%						
Test for overall effect: Z = 1.99 (P = 0.05)						
**1.47.3 Combined**						
Balaban 1988	0.16224858	0.2146303	40	46	1.2%	0.16 [−0.26, 0.58]
Markle-Reid 2010	0.05052332	0.2072482	49	43	1.3%	0.05 [−0.36, 0.46]
Luukinen 2006	0.13024238	0.16854423	144	150	1.9%	0.13 [−0.20, 0.46]
Hendriks 2008	−0.05194913	0.13624672	124	134	2.7%	−0.05 [−0.32, 0.22]
Sommers 2000	−0.17900004	0.10217801	201	183	4.3%	−0.18 [−0.38, 0.02]
Sorensen 1988	−0.06084525	0.07678877	315	366	6.3%	−0.06 [−0.21, 0.09]
Counsell 2007	0.09213862	0.06485754	474	477	7.8%	0.09 [−0.03, 0.22]
Byles 2004	−0.14951835	0.06180403	636	446	8.2%	−0.15 [−0.27, −0.03]
**Subtotal (95% CI)**			**1983**	**1845**	**33.5%**	**−0.04 [−0.13, 0.06]**
Heterogeneity: Tau² = 0.01; Chi² = 11.32, df = 7 (P = 0.13); I² = 38%						
Test for overall effect: Z = 0.77 (P = 0.44)						
**Total (95% CI)**			**5136**	**4756**	**100.0%**	**−0.06 [−0.11, −0.01]**
Heterogeneity: Tau² = 0.00; Chi² = 35.69, df = 28 (P = 0.15); I² = 22%						
Test for overall effect: Z = 2.57 (P = 0.01)						
Test for subgroup differences: Chi² = 1.55, df = 2 (P = 0.46), I² = 0%						

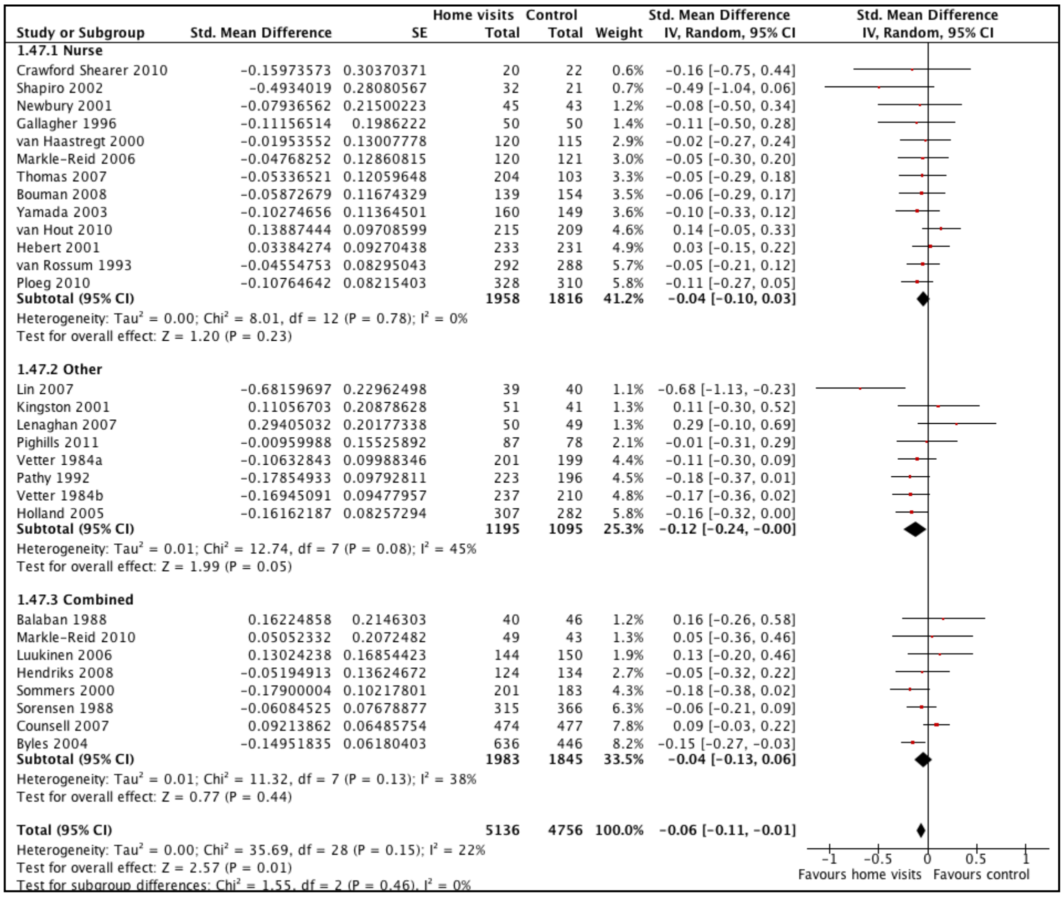

Analysis 40: Quality of life; number of visits subgroups

Study or Subgroup	Std. Mean Difference	SE	Home visits Total	Control Total	Weight	Std. Mean Difference IV, Random, 95% CI
**1.48.1 1 Visit**						
Newbury 2001	−0.07936562	0.21500223	45	43	1.2%	−0.08 [−0.50, 0.34]
Kingston 2001	0.11056703	0.20878628	51	41	1.2%	0.11 [−0.30, 0.52]
Pighills 2011	−0.00959988	0.15525892	87	78	2.1%	−0.01 [−0.31, 0.29]
Hendriks 2008	−0.05194913	0.13624672	124	134	2.7%	−0.05 [−0.32, 0.22]
Hebert 2001	0.03384274	0.09270438	233	231	5.0%	0.03 [−0.15, 0.22]
Sorensen 1988	−0.06084525	0.07678877	315	366	6.6%	−0.06 [−0.21, 0.09]
**Subtotal (95% CI)**			**855**	**893**	**18.8%**	**−0.02 [−0.12, 0.07]**
Heterogeneity: Tau² = 0.00; Chi² = 1.15, df = 5 (P = 0.95); I² = 0%						
Test for overall effect: Z = 0.42 (P = 0.67)						
**1.48.2 2-4 Visits**						
Lenaghan 2007	0.29405032	0.20177338	50	49	1.3%	0.29 [−0.10, 0.69]
Gallagher 1996	−0.11156514	0.1986222	50	50	1.4%	−0.11 [−0.50, 0.28]
Luukinen 2006	0.13024238	0.16854423	144	150	1.8%	0.13 [−0.20, 0.46]
Thomas 2007	−0.05336521	0.12059648	204	103	3.3%	−0.05 [−0.29, 0.18]
Vetter 1984a	−0.10632843	0.09988346	201	199	4.5%	−0.11 [−0.30, 0.09]
van Hout 2010	0.13887444	0.09708599	215	209	4.7%	0.14 [−0.05, 0.33]
Vetter 1984b	−0.16945091	0.09477957	237	210	4.9%	−0.17 [−0.36, 0.02]
Holland 2005	−0.16162187	0.08257294	307	282	5.9%	−0.16 [−0.32, 0.00]
Ploeg 2010	−0.10764642	0.08215403	328	310	6.0%	−0.11 [−0.27, 0.05]
**Subtotal (95% CI)**			**1736**	**1562**	**33.7%**	**−0.05 [−0.14, 0.04]**
Heterogeneity: Tau² = 0.01; Chi² = 12.01, df = 8 (P = 0.15); I² = 33%						
Test for overall effect: Z = 1.14 (P = 0.25)						
**1.48.3 5 or More Visits**						
Crawford Shearer 2010	−0.15973573	0.30370371	20	22	0.6%	−0.16 [−0.75, 0.44]
Lin 2007	−0.68159697	0.22962498	39	40	1.0%	−0.68 [−1.13, −0.23]
Markle-Reid 2010	0.05052332	0.2072482	49	43	1.2%	0.05 [−0.36, 0.46]
van Haastregt 2000	−0.01953552	0.13007778	120	115	2.9%	−0.02 [−0.27, 0.24]
Markle-Reid 2006	−0.04768252	0.12860815	120	121	3.0%	−0.05 [−0.30, 0.20]
Bouman 2008	−0.05872679	0.11674329	139	154	3.5%	−0.06 [−0.29, 0.17]
Yamada 2003	−0.10274656	0.11364501	160	149	3.6%	−0.10 [−0.33, 0.12]
Sommers 2000	−0.17900004	0.10217801	201	183	4.3%	−0.18 [−0.38, 0.02]
Pathy 1992	−0.17854933	0.09792811	223	196	4.6%	−0.18 [−0.37, 0.01]
van Rossum 1993	−0.04554753	0.08295043	292	288	5.9%	−0.05 [−0.21, 0.12]
Counsell 2007	0.09213862	0.06485754	474	477	8.2%	0.09 [−0.03, 0.22]
Byles 2004	−0.14951835	0.06180403	636	446	8.6%	−0.15 [−0.27, −0.03]
**Subtotal (95% CI)**			**2473**	**2234**	**47.5%**	**−0.09 [−0.17, −0.01]**
Heterogeneity: Tau² = 0.01; Chi² = 18.10, df = 11 (P = 0.08); I² = 39%						
Test for overall effect: Z = 2.15 (P = 0.03)						
**Total (95% CI)**			**5064**	**4689**	**100.0%**	**−0.06 [−0.11, −0.02]**
Heterogeneity: Tau² = 0.00; Chi² = 32.24, df = 26 (P = 0.19); I² = 19%						
Test for overall effect: Z = 2.60 (P = 0.009)						
Test for subgroup differences: Chi² = 1.14, df = 2 (P = 0.56), I² = 0%						

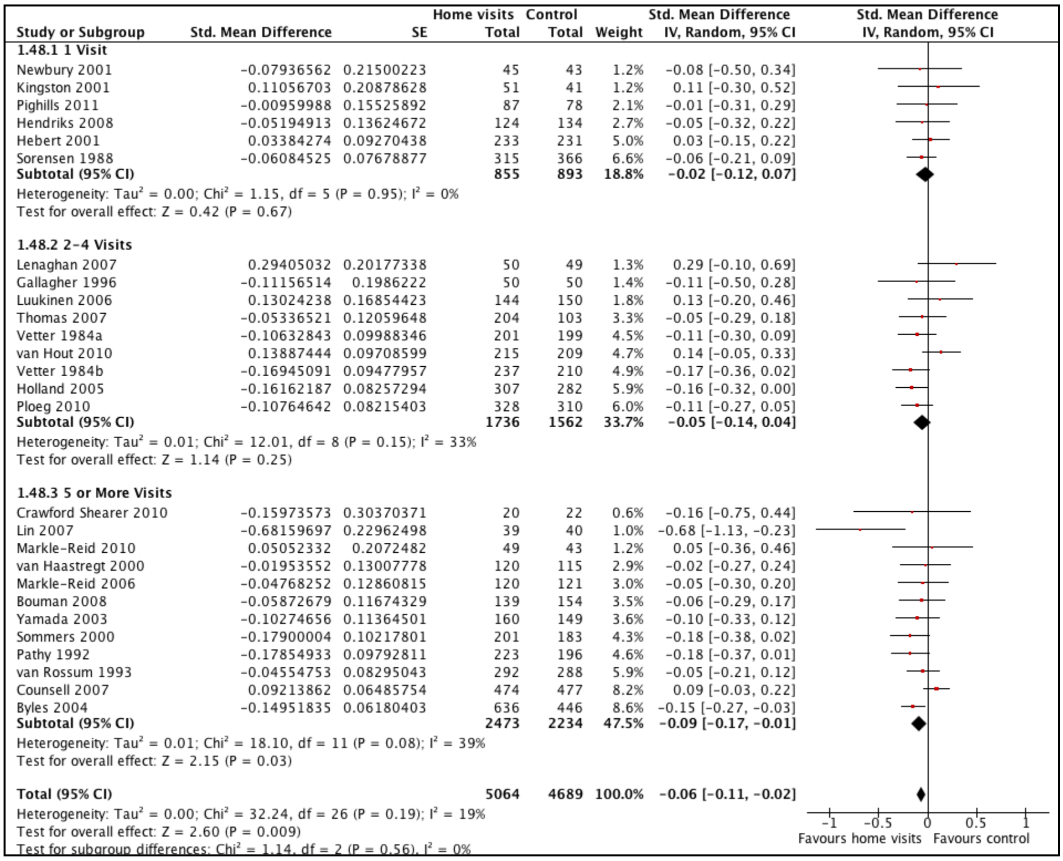

Analysis 41: Hospitalisation admissions, results at longest follow-up

Study or Subgroup	log[Rate Ratio]	SE	Home visits Total	Control Total	Weight	Rate Ratio IV, Random, 95% CI
Lightbody 2002	−0.210405	0.474342	155	159	1.9%	0.81 [0.32, 2.05]
Lenaghan 2007	−0.07195722	0.31244047	68	66	3.9%	0.93 [0.50, 1.72]
Caplan 2004	−0.48954823	0.29135827	370	369	4.3%	0.61 [0.35, 1.08]
Dalby 2000	0.27287577	0.28986801	70	69	4.4%	1.31 [0.74, 2.32]
Tinetti 1994	−0.31480238	0.19720266	153	148	7.3%	0.73 [0.50, 1.07]
Balaban 1988	0.59420569	0.18601026	103	95	7.8%	1.81 [1.26, 2.61]
Close 1999	−0.20134241	0.15748661	184	213	9.2%	0.82 [0.60, 1.11]
Sommers 2000	0.13693273	0.13824932	238	227	10.3%	1.15 [0.87, 1.50]
Pathy 1992	−0.12503838	0.13131131	219	200	10.7%	0.88 [0.68, 1.14]
Pathy 1992	−0.13916677	0.11309618	150	156	11.8%	0.87 [0.70, 1.09]
van Rossum 1993	−0.32160726	0.09614351	292	288	12.9%	0.72 [0.60, 0.88]
Counsell 2007	−0.05338644	0.05272497	474	477	15.6%	0.95 [0.85, 1.05]
**Total (95% CI)**			**2476**	**2467**	**100.0%**	**0.93 [0.81, 1.06]**

Heterogeneity: Tau² = 0.03; Chi² = 28.07, df = 11 (P = 0.003); I² = 61%
Test for overall effect: Z = 1.10 (P = 0.27)

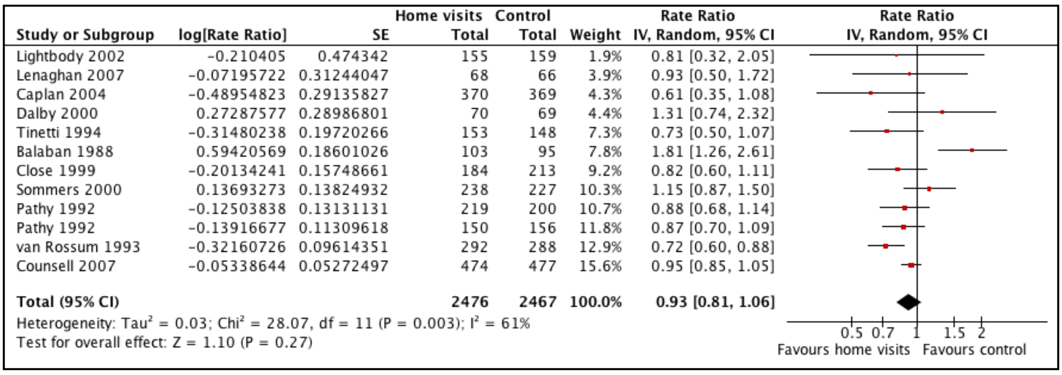

Analysis 42: Hospitalisation admissions at each follow-up interval

Study or Subgroup	log[Rate Ratio]	SE	Home visits Total	Control Total	Weight	Rate Ratio IV, Random, 95% CI
**1.50.1 01-11 months**						
Lightbody 2002	−0.210405	0.474342	155	159	16.8%	0.81 [0.32, 2.05]
Lenaghan 2007	−0.07195722	0.31244047	68	66	38.7%	0.93 [0.50, 1.72]
Caplan 2004	−0.48954823	0.29135827	370	369	44.5%	0.61 [0.35, 1.08]
**Subtotal (95% CI)**			**593**	**594**	**100.0%**	**0.75 [0.52, 1.11]**
Heterogeneity: Tau² = 0.00; Chi² = 0.98, df = 2 (P = 0.61); I² = 0%						
Test for overall effect: Z = 1.45 (P = 0.15)						
**1.50.2 12-23 months**						
Dalby 2000	0.27287577	0.28986801	70	69	9.0%	1.31 [0.74, 2.32]
Tinetti 1994	−0.31480238	0.19720266	153	148	13.0%	0.73 [0.50, 1.07]
Balaban 1988	0.59420569	0.18601026	103	95	13.6%	1.81 [1.26, 2.61]
van Rossum 1993	−0.415743	0.16824	292	288	14.5%	0.66 [0.47, 0.92]
Close 1999	−0.20134241	0.15748661	184	213	15.1%	0.82 [0.60, 1.11]
Sommers 2000	0.10222723	0.15102741	238	227	15.4%	1.11 [0.82, 1.49]
Counsell 2007	0.074388	0.073467	474	477	19.3%	1.08 [0.93, 1.24]
**Subtotal (95% CI)**			**1514**	**1517**	**100.0%**	**1.00 [0.80, 1.26]**
Heterogeneity: Tau² = 0.06; Chi² = 22.70, df = 6 (P = 0.0009); I² = 74%						
Test for overall effect: Z = 0.03 (P = 0.97)						
**1.50.3 24-35 months**						
Sommers 2000	0.13693273	0.13824932	238	227	28.2%	1.15 [0.87, 1.50]
van Rossum 1993	−0.3740302	0.11381009	292	288	31.7%	0.69 [0.55, 0.86]
Counsell 2007	−0.05338644	0.05272497	474	477	40.1%	0.95 [0.85, 1.05]
**Subtotal (95% CI)**			**1004**	**992**	**100.0%**	**0.90 [0.71, 1.15]**
Heterogeneity: Tau² = 0.04; Chi² = 9.39, df = 2 (P = 0.009); I² = 79%						
Test for overall effect: Z = 0.82 (P = 0.41)						
**1.50.4 36+ months**						
Pathy 1992	−0.12503838	0.13131131	219	200	24.3%	0.88 [0.68, 1.14]
Pathy 1992	−0.13916677	0.11309618	150	156	32.2%	0.87 [0.70, 1.09]
van Rossum 1993	−0.32160726	0.09614351	292	288	43.4%	0.72 [0.60, 0.88]
**Subtotal (95% CI)**			**661**	**644**	**100.0%**	**0.81 [0.71, 0.92]**
Heterogeneity: Tau² = 0.00; Chi² = 2.15, df = 2 (P = 0.34); I² = 7%						
Test for overall effect: Z = 3.23 (P = 0.001)						

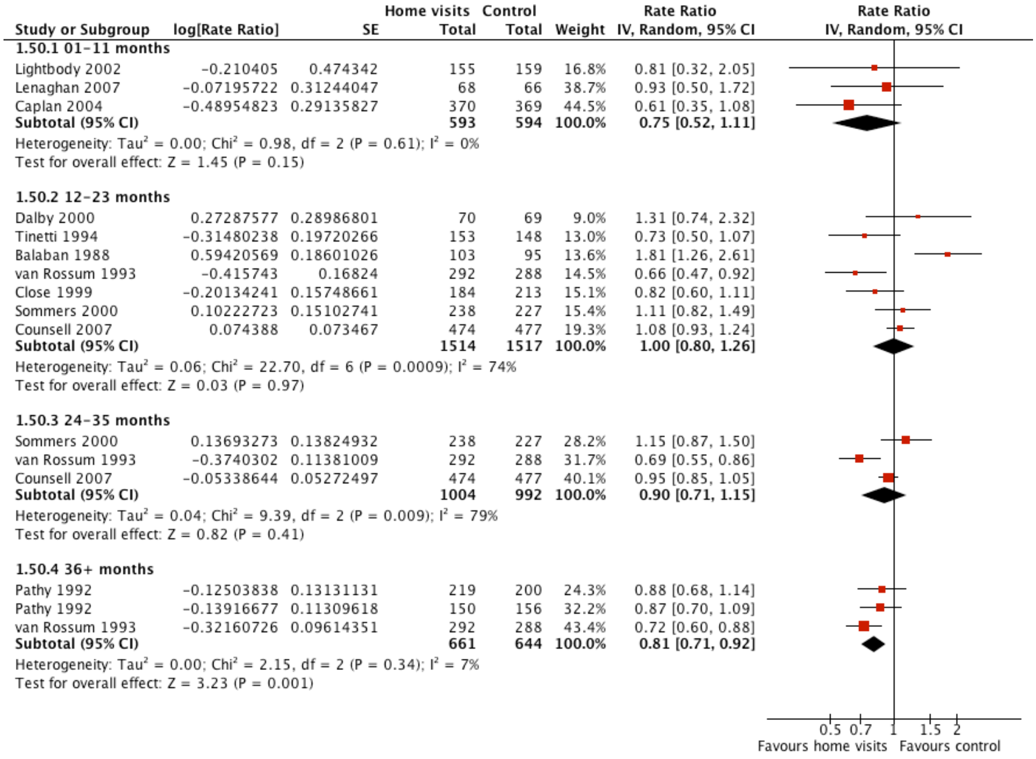

Analysis 43: Hospitalisation admissions; focus of visit subgroups

Study or Subgroup	log[Rate Ratio]	SE	Home visits Total	Control Total	Weight	Rate Ratio IV, Random, 95% CI
**1.51.2 MGA Only**						
Caplan 2004	−0.48954823	0.29135827	370	369	4.3%	0.61 [0.35, 1.08]
Dalby 2000	0.27287577	0.28986801	70	69	4.4%	1.31 [0.74, 2.32]
Balaban 1988	0.59420569	0.18601026	103	95	7.8%	1.81 [1.26, 2.61]
Sommers 2000	0.13693273	0.13824932	238	227	10.3%	1.15 [0.87, 1.50]
Pathy 1992	−0.12503838	0.13131131	219	200	10.7%	0.88 [0.68, 1.14]
Pathy 1992	−0.13916677	0.11309618	150	156	11.8%	0.87 [0.70, 1.09]
van Rossum 1993	−0.32160726	0.09614351	292	288	12.9%	0.72 [0.60, 0.88]
Counsell 2007	−0.05338644	0.05272497	474	477	15.6%	0.95 [0.85, 1.05]
**Subtotal (95% CI)**			**1916**	**1881**	**77.7%**	**0.97 [0.81, 1.15]**
Heterogeneity: Tau² = 0.04; Chi² = 25.96, df = 7 (P = 0.0005); I² = 73%						
Test for overall effect: Z = 0.37 (P = 0.71)						
**1.51.3 Both**						
Lightbody 2002	−0.210405	0.474342	155	159	1.9%	0.81 [0.32, 2.05]
Tinetti 1994	−0.31480238	0.19720266	153	148	7.3%	0.73 [0.50, 1.07]
Close 1999	−0.20134241	0.15748661	184	213	9.2%	0.82 [0.60, 1.11]
**Subtotal (95% CI)**			**492**	**520**	**18.4%**	**0.78 [0.62, 0.99]**
Heterogeneity: Tau² = 0.00; Chi² = 0.21, df = 2 (P = 0.90); I² = 0%						
Test for overall effect: Z = 2.04 (P = 0.04)						
**1.51.4 Neither**						
Lenaghan 2007	−0.07195722	0.31244047	68	66	3.9%	0.93 [0.50, 1.72]
**Subtotal (95% CI)**			**68**	**66**	**3.9%**	**0.93 [0.50, 1.72]**
Heterogeneity: Not applicable						
Test for overall effect: Z = 0.23 (P = 0.82)						
**Total (95% CI)**			**2476**	**2467**	**100.0%**	**0.93 [0.81, 1.06]**
Heterogeneity: Tau² = 0.03; Chi² = 28.07, df = 11 (P = 0.003); I² = 61%						
Test for overall effect: Z = 1.10 (P = 0.27)						
Test for subgroup differences: Chi² = 2.04, df = 2 (P = 0.36), I² = 2.1%						

Rate Ratio IV, Random, 95% CI

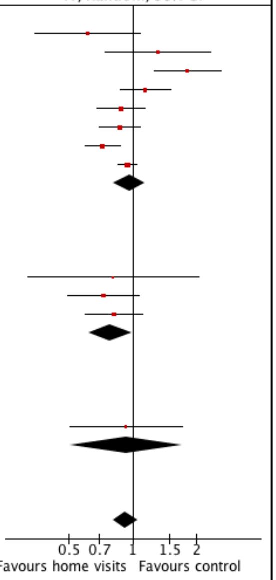

Analysis 44: Hospitalisation admissions; age of participants subgroups

Study or Subgroup	log[Rate Ratio]	SE	Home visits Total	Control Total	Weight	Rate Ratio IV, Random, 95% CI
**1.52.1 <=70**						
Balaban 1988	0.59420569	0.18601026	103	95	7.8%	1.81 [1.26, 2.61]
Pathy 1992	−0.12503838	0.13131131	219	200	10.7%	0.88 [0.68, 1.14]
**Subtotal (95% CI)**			**322**	**295**	**18.4%**	**1.25 [0.62, 2.53]**
Heterogeneity: Tau² = 0.23; Chi² = 9.98, df = 1 (P = 0.002); I² = 90%						
Test for overall effect: Z = 0.62 (P = 0.54)						
**1.52.2 71-75**						
Lightbody 2002	−0.210405	0.474342	155	159	1.9%	0.81 [0.32, 2.05]
Counsell 2007	−0.05338644	0.05272497	474	477	15.6%	0.95 [0.85, 1.05]
**Subtotal (95% CI)**			**629**	**636**	**17.5%**	**0.95 [0.85, 1.05]**
Heterogeneity: Tau² = 0.00; Chi² = 0.11, df = 1 (P = 0.74); I² = 0%						
Test for overall effect: Z = 1.06 (P = 0.29)						
**1.52.3 76-80**						
Dalby 2000	0.27287577	0.28986801	70	69	4.4%	1.31 [0.74, 2.32]
Tinetti 1994	−0.31480238	0.19720266	153	148	7.3%	0.73 [0.50, 1.07]
Close 1999	−0.20134241	0.15748661	184	213	9.2%	0.82 [0.60, 1.11]
Sommers 2000	0.13693273	0.13824932	238	227	10.3%	1.15 [0.87, 1.50]
Pathy 1992	−0.13916677	0.11309618	150	156	11.8%	0.87 [0.70, 1.09]
van Rossum 1993	−0.32160726	0.09614351	292	288	12.9%	0.72 [0.60, 0.88]
**Subtotal (95% CI)**			**1087**	**1101**	**55.8%**	**0.87 [0.74, 1.04]**
Heterogeneity: Tau² = 0.02; Chi² = 10.39, df = 5 (P = 0.07); I² = 52%						
Test for overall effect: Z = 1.55 (P = 0.12)						
**1.52.4 81-85**						
Lenaghan 2007	−0.07195722	0.31244047	68	66	3.9%	0.93 [0.50, 1.72]
Caplan 2004	−0.48954823	0.29135827	370	369	4.3%	0.61 [0.35, 1.08]
**Subtotal (95% CI)**			**438**	**435**	**8.2%**	**0.74 [0.49, 1.13]**
Heterogeneity: Tau² = 0.00; Chi² = 0.96, df = 1 (P = 0.33); I² = 0%						
Test for overall effect: Z = 1.39 (P = 0.17)						
**Total (95% CI)**			**2476**	**2467**	**100.0%**	**0.93 [0.81, 1.06]**
Heterogeneity: Tau² = 0.03; Chi² = 28.07, df = 11 (P = 0.003); I² = 61%						
Test for overall effect: Z = 1.10 (P = 0.27)						
Test for subgroup differences: Chi² = 2.35, df = 3 (P = 0.50), I² = 0%						

Rate Ratio IV, Random, 95% CI

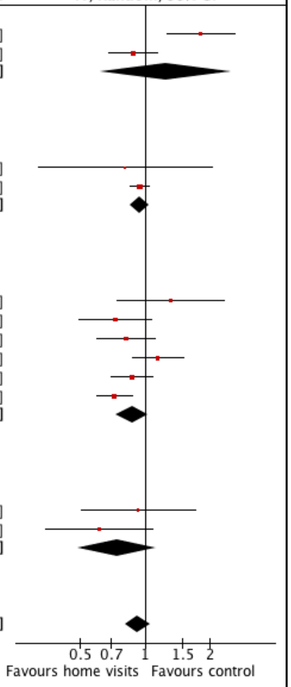

Analysis 45: Hospitalisation admissions; type of visitor subgroups

Study or Subgroup	log[Hazard Ratio]	SE	Home visits Total	Control Total	Weight	Hazard Ratio IV, Fixed, 95% CI
**1.53.1 Nurse**						
Lightbody 2002	−0.210405	0.474342	155	159	0.6%	0.81 [0.32, 2.05]
Caplan 2004	−0.48954823	0.29135827	370	369	1.5%	0.61 [0.35, 1.08]
Dalby 2000	0.27287577	0.28986801	70	69	1.5%	1.31 [0.74, 2.32]
van Rossum 1993	−0.32160726	0.09614351	292	288	13.7%	0.72 [0.60, 0.88]
**Subtotal (95% CI)**			**887**	**885**	**17.3%**	**0.76 [0.64, 0.89]**
Heterogeneity: Chi² = 4.36, df = 3 (P = 0.22); I² = 31%						
Test for overall effect: Z = 3.28 (P = 0.001)						
**1.53.2 Other**						
Lenaghan 2007	−0.07195722	0.31244047	68	66	1.3%	0.93 [0.50, 1.72]
Close 1999	−0.20134241	0.15748661	184	213	5.1%	0.82 [0.60, 1.11]
Pathy 1992	−0.12503838	0.13131131	219	200	7.3%	0.88 [0.68, 1.14]
Pathy 1992	−0.13916677	0.11309618	150	156	9.9%	0.87 [0.70, 1.09]
**Subtotal (95% CI)**			**621**	**635**	**23.6%**	**0.87 [0.75, 1.00]**
Heterogeneity: Chi² = 0.21, df = 3 (P = 0.98); I² = 0%						
Test for overall effect: Z = 1.97 (P = 0.05)						
**1.53.3 Combined**						
Tinetti 1994	−0.31480238	0.19720266	153	148	3.3%	0.73 [0.50, 1.07]
Balaban 1988	0.59420569	0.18601026	103	95	3.7%	1.81 [1.26, 2.61]
Sommers 2000	0.13693273	0.13824932	238	227	6.6%	1.15 [0.87, 1.50]
Counsell 2007	−0.05338644	0.05272497	474	477	45.6%	0.95 [0.85, 1.05]
**Subtotal (95% CI)**			**968**	**947**	**59.1%**	**0.99 [0.91, 1.09]**
Heterogeneity: Chi² = 14.74, df = 3 (P = 0.002); I² = 80%						
Test for overall effect: Z = 0.14 (P = 0.89)						
**Total (95% CI)**			**2476**	**2467**	**100.0%**	**0.92 [0.86, 0.98]**
Heterogeneity: Chi² = 28.07, df = 11 (P = 0.003); I² = 61%						
Test for overall effect: Z = 2.43 (P = 0.02)						
Test for subgroup differences: Chi² = 8.76, df = 2 (P = 0.01), I² = 77.2%						

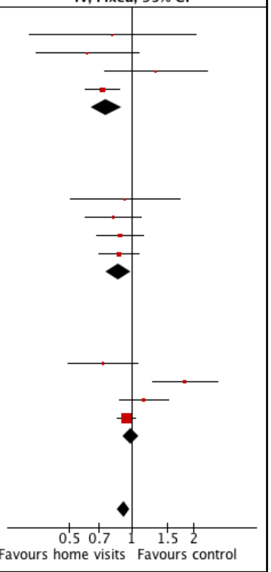

Analysis 46: Hospitalisation admissions; number of visits subgroups

Study or Subgroup	log[Rate Ratio]	SE	Home visits Total	Control Total	Weight	Rate Ratio IV, Random, 95% CI
**1.54.1 1 Visit**						
Lightbody 2002	−0.210405	0.474342	155	159	1.2%	0.81 [0.32, 2.05]
Close 1999	−0.20134241	0.15748661	184	213	8.3%	0.82 [0.60, 1.11]
**Subtotal (95% CI)**			**339**	**372**	**9.5%**	**0.82 [0.61, 1.09]**
Heterogeneity: Tau² = 0.00; Chi² = 0.00, df = 1 (P = 0.99); I² = 0%						
Test for overall effect: Z = 1.35 (P = 0.18)						
**1.54.2 2-4 Visits**						
Lenaghan 2007	−0.07195722	0.31244047	68	66	2.6%	0.93 [0.50, 1.72]
Caplan 2004	−0.48954823	0.29135827	370	369	2.9%	0.61 [0.35, 1.08]
Dalby 2000	0.27287577	0.28986801	70	69	2.9%	1.31 [0.74, 2.32]
**Subtotal (95% CI)**			**508**	**504**	**8.4%**	**0.91 [0.58, 1.41]**
Heterogeneity: Tau² = 0.06; Chi² = 3.45, df = 2 (P = 0.18); I² = 42%						
Test for overall effect: Z = 0.43 (P = 0.67)						
**1.54.3 5 or More Visits**						
Tinetti 1994	−0.31480238	0.19720266	153	148	5.8%	0.73 [0.50, 1.07]
Sommers 2000	0.13693273	0.13824932	238	227	10.1%	1.15 [0.87, 1.50]
Pathy 1992	−0.12503838	0.13131131	219	200	10.9%	0.88 [0.68, 1.14]
Pathy 1992	−0.13916677	0.11309618	150	156	13.2%	0.87 [0.70, 1.09]
van Rossum 1993	−0.32160726	0.09614351	292	288	16.0%	0.72 [0.60, 0.88]
Counsell 2007	−0.05338644	0.05272497	474	477	26.1%	0.95 [0.85, 1.05]
**Subtotal (95% CI)**			**1526**	**1496**	**82.1%**	**0.88 [0.78, 1.00]**
Heterogeneity: Tau² = 0.01; Chi² = 10.34, df = 5 (P = 0.07); I² = 52%						
Test for overall effect: Z = 1.97 (P = 0.05)						
**Total (95% CI)**			**2373**	**2372**	**100.0%**	**0.88 [0.79, 0.97]**
Heterogeneity: Tau² = 0.01; Chi² = 14.18, df = 10 (P = 0.16); I² = 29%						
Test for overall effect: Z = 2.49 (P = 0.01)						
Test for subgroup differences: Chi² = 0.25, df = 2 (P = 0.88), I² = 0%						

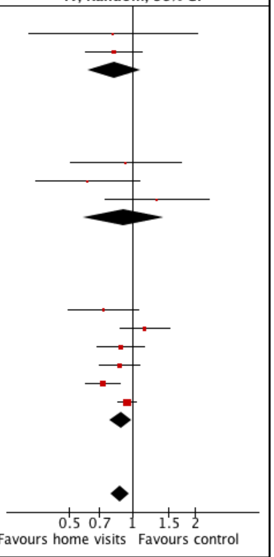

Analysis 47: Hospitalisation (days), results at longest follow-up

Study or Subgroup	log[Rate Ratio]	SE	Home visits Total	Control Total	Weight	Rate Ratio IV, Random, 95% CI
Markle-Reid 2010	2.41082692	0.30107939	54	55	4.5%	11.14 [6.18, 20.10]
Davison 2005	−1.69743135	0.09532616	159	154	7.5%	0.18 [0.15, 0.22]
Tinetti 1994	−0.83210697	0.06945248	153	148	7.8%	0.44 [0.38, 0.50]
Balaban 1988	−0.31561464	0.06397298	103	95	7.9%	0.73 [0.64, 0.83]
Lightbody 2002	−0.21465082	0.06182697	155	159	7.9%	0.81 [0.71, 0.91]
Dalby 2000	0.56767531	0.04626165	70	69	8.0%	1.76 [1.61, 1.93]
Stuck 1995	0.20802991	0.04419946	215	199	8.0%	1.23 [1.13, 1.34]
Bernabei 1998	−0.43656359	0.0429571	99	100	8.0%	0.65 [0.59, 0.70]
Bouman 2008	−0.02296921	0.03816434	160	170	8.0%	0.98 [0.91, 1.05]
Pathy 1992 (1)	−0.05789343	0.03040131	150	156	8.1%	0.94 [0.89, 1.00]
Pathy 1992 (2)	−0.38036169	0.02767676	219	200	8.1%	0.68 [0.65, 0.72]
Counsell 2007	−0.0770609	0.02262275	474	477	8.1%	0.93 [0.89, 0.97]
van Rossum 1993	−0.25133501	0.02166482	292	288	8.1%	0.78 [0.75, 0.81]
**Total (95% CI)**			**2303**	**2270**	**100.0%**	**0.85 [0.71, 1.02]**
Heterogeneity: Tau² = 0.11; Chi² = 909.84, df = 12 (P < 0.00001); I² = 99%						
Test for overall effect: Z = 1.73 (P = 0.08)						

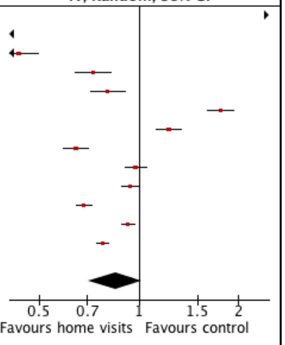

(1) Pathy 1992b
(2) Pathy 1992a

Analysis 48: Hospitalisation (days) at each follow-up interval

Study or Subgroup	Home visits Events	Home visits Total	Control Events	Control Total	Weight	Risk Ratio M-H, Random, 95% CI
**1.62.1 01-11 months**						
Ciaschini 2009	2	101	3	100	9.3%	0.66 [0.11, 3.87]
Ciechanowski 2004	15	69	21	62	90.7%	0.64 [0.36, 1.13]
**Subtotal (95% CI)**		**170**		**162**	**100.0%**	**0.64 [0.38, 1.10]**
Total events	17		24			
Heterogeneity: Tau² = 0.00; Chi² = 0.00, df = 1 (P = 0.98); I² = 0%						
Test for overall effect: Z = 1.60 (P = 0.11)						
**1.62.2 12-23 months**						
Kono 2004	1	59	1	60	0.3%	1.02 [0.07, 15.88]
McEwan 1990	3	135	4	122	0.9%	0.68 [0.15, 2.97]
Hogan 2001	5	79	6	84	1.5%	0.89 [0.28, 2.79]
Davison 2005	14	159	17	154	4.1%	0.80 [0.41, 1.56]
Fabacher 1994	22	100	23	95	6.5%	0.91 [0.54, 1.52]
Tinetti 1994	32	147	36	144	9.1%	0.87 [0.57, 1.32]
Stuck 1995	39	215	42	199	10.0%	0.86 [0.58, 1.27]
van Rossum 1993	45	292	64	288	12.0%	0.69 [0.49, 0.98]
Bernabei 1998	36	99	51	100	13.1%	0.71 [0.52, 0.99]
Bouman 2008	68	160	61	170	16.5%	1.18 [0.90, 1.55]
van Hout 2010	163	331	141	320	25.9%	1.12 [0.95, 1.32]
**Subtotal (95% CI)**		**1776**		**1736**	**100.0%**	**0.92 [0.80, 1.07]**
Total events	428		446			
Heterogeneity: Tau² = 0.01; Chi² = 13.53, df = 10 (P = 0.20); I² = 26%						
Test for overall effect: Z = 1.10 (P = 0.27)						
**1.62.3 24-35 months**						
Bouman 2008	80	160	71	170	26.8%	1.20 [0.95, 1.52]
van Rossum 1993	88	292	108	288	27.5%	0.80 [0.64, 1.01]
Sorensen 1988	345	585	482	777	45.7%	0.95 [0.87, 1.04]
**Subtotal (95% CI)**		**1037**		**1235**	**100.0%**	**0.97 [0.81, 1.15]**
Total events	513		661			
Heterogeneity: Tau² = 0.02; Chi² = 5.72, df = 2 (P = 0.06); I² = 65%						
Test for overall effect: Z = 0.39 (P = 0.70)						
**1.62.4 36+ months**						
Stuck 1995	99	215	93	199	22.4%	0.99 [0.80, 1.21]
van Rossum 1993	121	292	133	288	28.2%	0.90 [0.75, 1.08]
Byles 2004	261	636	198	446	49.4%	0.92 [0.80, 1.06]
**Subtotal (95% CI)**		**1143**		**933**	**100.0%**	**0.93 [0.84, 1.03]**
Total events	481		424			
Heterogeneity: Tau² = 0.00; Chi² = 0.45, df = 2 (P = 0.80); I² = 0%						
Test for overall effect: Z = 1.45 (P = 0.15)						

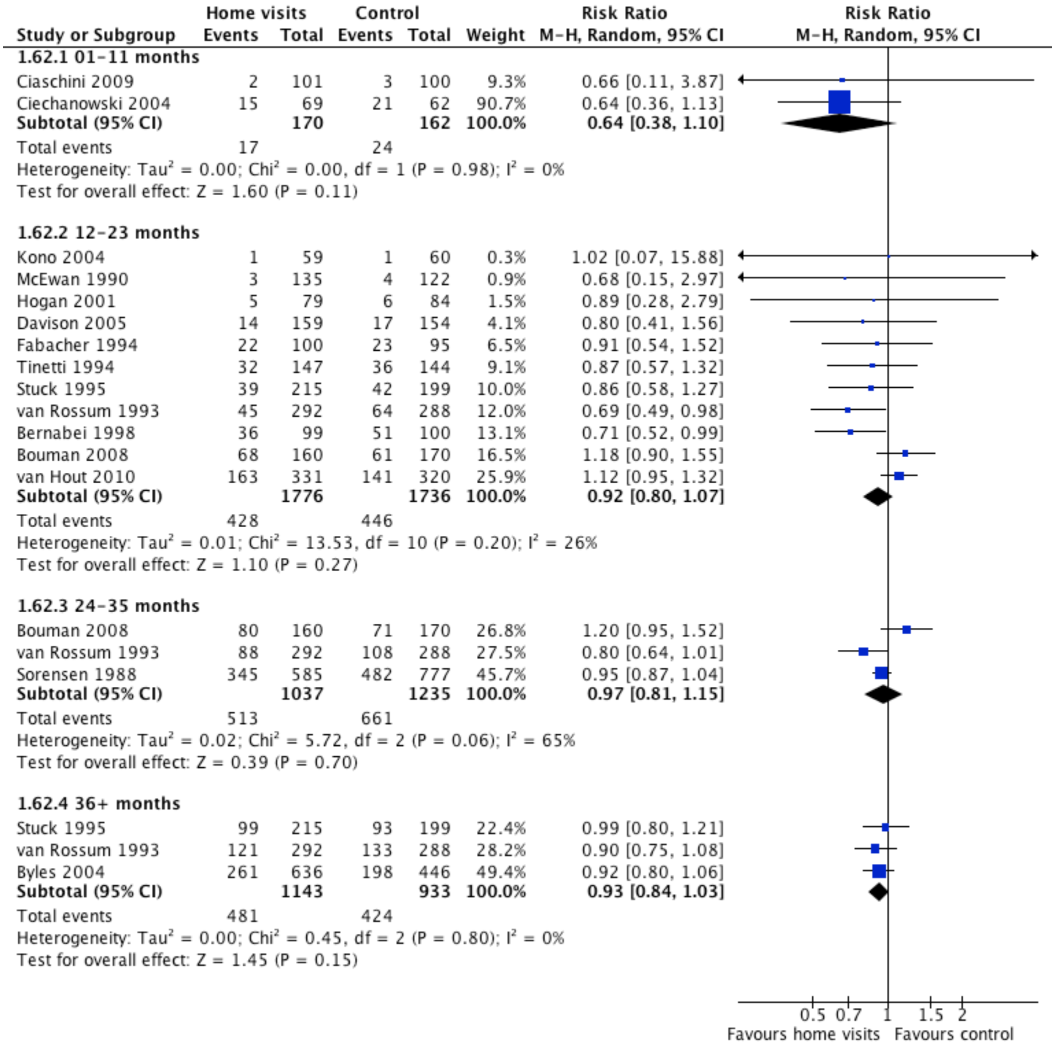

Analysis 49: Hospitalisation (days); focus of visit subgroups

Study or Subgroup	log[Rate Ratio]	SE	Home visits Total	Control Total	Weight	Rate Ratio IV, Random, 95% CI
**1.57.2 MGA Only**						
Balaban 1988	−0.31561464	0.06397298	103	95	7.9%	0.73 [0.64, 0.83]
Dalby 2000	0.56767531	0.04626165	70	69	8.0%	1.76 [1.61, 1.93]
Stuck 1995	0.20802991	0.04419946	215	199	8.0%	1.23 [1.13, 1.34]
Bernabei 1998	−0.43656359	0.0429571	99	100	8.0%	0.65 [0.59, 0.70]
Bouman 2008	−0.02296921	0.03816434	160	170	8.0%	0.98 [0.91, 1.05]
Pathy 1992 (1)	−0.05789343	0.03040131	219	200	8.1%	0.94 [0.89, 1.00]
Pathy 1992 (2)	−0.38036169	0.02767676	150	156	8.1%	0.68 [0.65, 0.72]
Counsell 2007	−0.0770609	0.02262275	474	477	8.1%	0.93 [0.89, 0.97]
van Rossum 1993	−0.25133501	0.02166482	292	288	8.1%	0.78 [0.75, 0.81]
**Subtotal (95% CI)**			**1782**	**1754**	**72.3%**	**0.92 [0.78, 1.08]**
Heterogeneity: Tau² = 0.06; Chi² = 477.23, df = 8 (P < 0.00001); I² = 98%						
Test for overall effect: Z = 1.01 (P = 0.31)						
**1.57.3 Both**						
Markle-Reid 2010	2.41082692	0.30107939	54	55	4.5%	11.14 [6.18, 20.10]
Davison 2005	−1.69743135	0.09532616	159	154	7.5%	0.18 [0.15, 0.22]
Tinetti 1994	−0.83210697	0.06945248	153	148	7.8%	0.44 [0.38, 0.50]
Lightbody 2002	−0.21465082	0.06182697	155	159	7.9%	0.81 [0.71, 0.91]
**Subtotal (95% CI)**			**521**	**516**	**27.7%**	**0.86 [0.37, 2.02]**
Heterogeneity: Tau² = 0.73; Chi² = 280.49, df = 3 (P < 0.00001); I² = 99%						
Test for overall effect: Z = 0.34 (P = 0.73)						
**Total (95% CI)**			**2303**	**2270**	**100.0%**	**0.85 [0.71, 1.02]**
Heterogeneity: Tau² = 0.11; Chi² = 909.84, df = 12 (P < 0.00001); I² = 99%						
Test for overall effect: Z = 1.73 (P = 0.08)						
Test for subgroup differences: Chi² = 0.02, df = 1 (P = 0.89), I² = 0%						

(1) Pathy 1992a
(2) Pathy 1992b

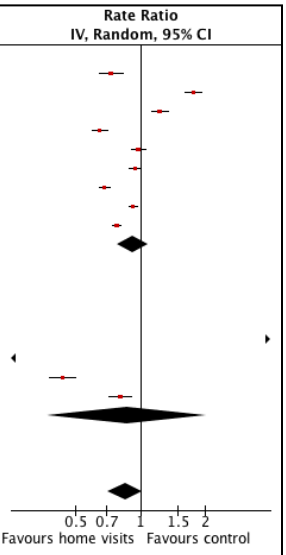

Analysis 50: Hospitalisation (days); age of participants subgroups

Study or Subgroup	log[Rate Ratio]	SE	Home visits Total	Control Total	Weight	Rate Ratio IV, Random, 95% CI
**1.58.1 <=70**						
Balaban 1988	−0.31561464	0.06397298	103	95	8.2%	0.73 [0.64, 0.83]
Pathy 1992 (1)	−0.38036169	0.02767676	219	200	8.5%	0.68 [0.65, 0.72]
**Subtotal (95% CI)**			**322**	**295**	**16.7%**	**0.69 [0.66, 0.73]**
Heterogeneity: Tau² = 0.00; Chi² = 0.86, df = 1 (P = 0.35); I² = 0%						
Test for overall effect: Z = 14.57 (P < 0.00001)						
**1.58.2 71-75**						
Lightbody 2002	−0.21465082	0.06182697	155	159	8.2%	0.81 [0.71, 0.91]
Counsell 2007	−0.0770609	0.02262275	474	477	8.5%	0.93 [0.89, 0.97]
**Subtotal (95% CI)**			**629**	**636**	**16.7%**	**0.87 [0.77, 1.00]**
Heterogeneity: Tau² = 0.01; Chi² = 4.37, df = 1 (P = 0.04); I² = 77%						
Test for overall effect: Z = 1.98 (P = 0.05)						
**1.58.3 76-80**						
Davison 2005	−1.69743135	0.09532616	159	154	7.8%	0.18 [0.15, 0.22]
Tinetti 1994	−0.83210697	0.06945248	153	148	8.2%	0.44 [0.38, 0.50]
Dalby 2000	0.56767531	0.04626165	70	69	8.4%	1.76 [1.61, 1.93]
Bouman 2008	−0.02296921	0.03816434	160	170	8.4%	0.98 [0.91, 1.05]
Pathy 1992	−0.05789343	0.03040131	150	156	8.5%	0.94 [0.89, 1.00]
van Rossum 1993	−0.25133501	0.02166482	292	288	8.5%	0.78 [0.75, 0.81]
**Subtotal (95% CI)**			**984**	**985**	**49.8%**	**0.69 [0.48, 0.98]**
Heterogeneity: Tau² = 0.19; Chi² = 642.76, df = 5 (P < 0.00001); I² = 99%						
Test for overall effect: Z = 2.06 (P = 0.04)						
**1.58.4 81-85**						
Stuck 1995	0.20802991	0.04419946	215	199	8.4%	1.23 [1.13, 1.34]
Bernabei 1998	−0.43656359	0.0429571	99	100	8.4%	0.65 [0.59, 0.70]
**Subtotal (95% CI)**			**314**	**299**	**16.8%**	**0.89 [0.47, 1.68]**
Heterogeneity: Tau² = 0.21; Chi² = 109.37, df = 1 (P < 0.00001); I² = 99%						
Test for overall effect: Z = 0.35 (P = 0.72)						
**Total (95% CI)**			**2249**	**2215**	**100.0%**	**0.75 [0.63, 0.90]**
Heterogeneity: Tau² = 0.10; Chi² = 836.43, df = 11 (P < 0.00001); I² = 99%						
Test for overall effect: Z = 3.05 (P = 0.002)						
Test for subgroup differences: Chi² = 11.20, df = 3 (P = 0.01), I² = 73.2%						

(1) Pathy 1992a

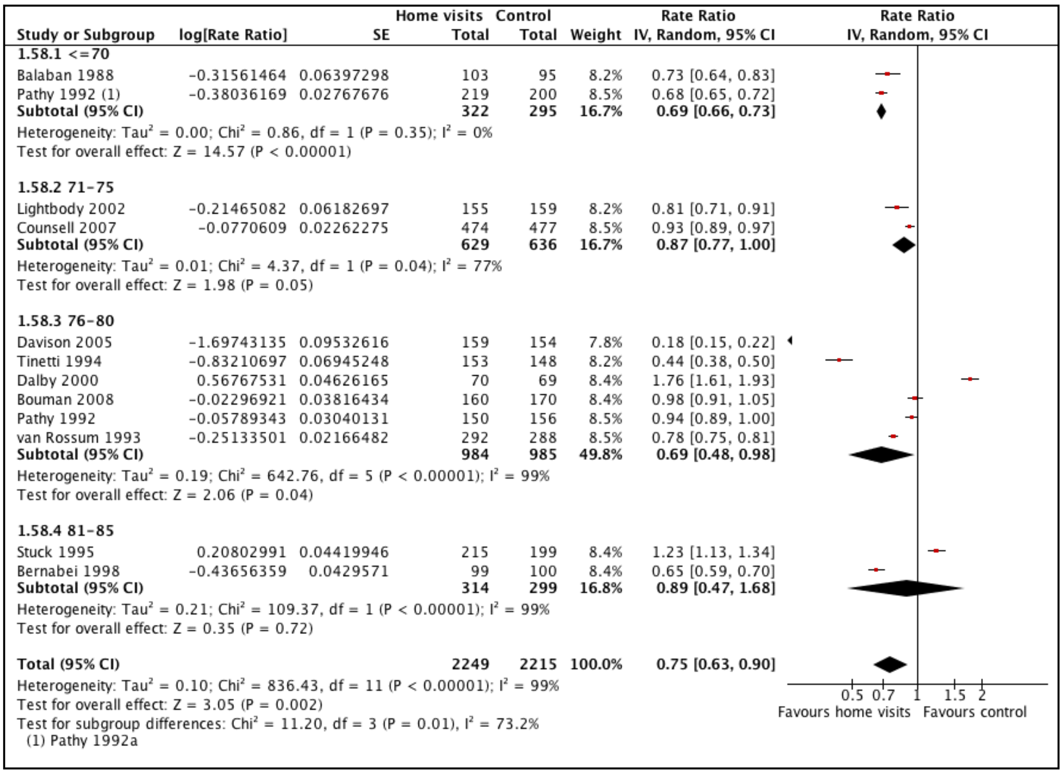

Analysis 51: Hospitalisation (days); type of visitor subgroups

Study or Subgroup	log[Rate Ratio]	SE	Home visits Total	Control Total	Weight	Rate Ratio IV, Random, 95% CI
**1.59.1 Nurse**						
Lightbody 2002	−0.21465082	0.06182697	155	159	7.9%	0.81 [0.71, 0.91]
Dalby 2000	0.56767531	0.04626165	70	69	8.0%	1.76 [1.61, 1.93]
Stuck 1995	0.20802991	0.04419946	215	199	8.0%	1.23 [1.13, 1.34]
Bouman 2008	−0.02296921	0.03816434	160	170	8.0%	0.98 [0.91, 1.05]
van Rossum 1993	−0.25133501	0.02166482	292	288	8.1%	0.78 [0.75, 0.81]
**Subtotal (95% CI)**			**892**	**885**	**40.0%**	**1.06 [0.79, 1.42]**
Heterogeneity: Tau² = 0.11; Chi² = 306.04, df = 4 (P < 0.00001); I² = 99%						
Test for overall effect: Z = 0.38 (P = 0.71)						
**1.59.2 Other**						
Bernabei 1998	−0.43656359	0.0429571	99	100	8.0%	0.65 [0.59, 0.70]
Pathy 1992	−0.05789343	0.03040131	150	156	8.1%	0.94 [0.89, 1.00]
Pathy 1992 (1)	−0.38036169	0.02767676	219	200	8.1%	0.68 [0.65, 0.72]
**Subtotal (95% CI)**			**468**	**456**	**24.2%**	**0.75 [0.59, 0.95]**
Heterogeneity: Tau² = 0.04; Chi² = 79.60, df = 2 (P < 0.00001); I² = 97%						
Test for overall effect: Z = 2.42 (P = 0.02)						
**1.59.3 Combined**						
Markle-Reid 2010	2.41082692	0.30107939	54	55	4.5%	11.14 [6.18, 20.10]
Davison 2005	−1.69743135	0.09532616	159	154	7.5%	0.18 [0.15, 0.22]
Tinetti 1994	−0.83210697	0.06945248	153	148	7.8%	0.44 [0.38, 0.50]
Balaban 1988	−0.31561464	0.06397298	103	95	7.9%	0.73 [0.64, 0.83]
Counsell 2007	−0.0770609	0.02262275	474	477	8.1%	0.93 [0.89, 0.97]
**Subtotal (95% CI)**			**943**	**929**	**35.8%**	**0.83 [0.46, 1.52]**
Heterogeneity: Tau² = 0.45; Chi² = 436.38, df = 4 (P < 0.00001); I² = 99%						
Test for overall effect: Z = 0.60 (P = 0.55)						
**Total (95% CI)**			**2303**	**2270**	**100.0%**	**0.85 [0.71, 1.02]**
Heterogeneity: Tau² = 0.11; Chi² = 909.84, df = 12 (P < 0.00001); I² = 99%						
Test for overall effect: Z = 1.73 (P = 0.08)						
Test for subgroup differences: Chi² = 3.25, df = 2 (P = 0.20), I² = 38.5%						

(1) Pathy 1992a

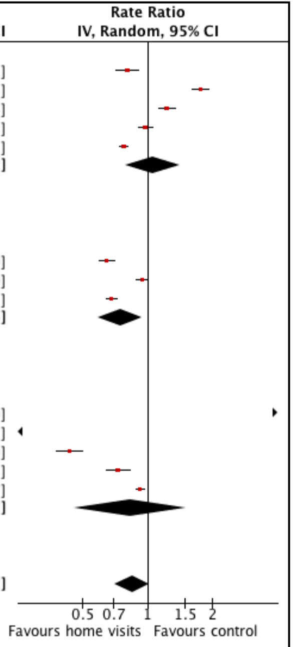

Analysis 52: Hospitalisation (days); number of visits subgroups

Study or Subgroup	log[Rate Ratio]	SE	Home visits Total	Control Total	Weight	Rate Ratio IV, Random, 95% CI
**1.60.1 1 Visit**						
Lightbody 2002	−0.21465082	0.06182697	155	159	9.3%	0.81 [0.71, 0.91]
**Subtotal (95% CI)**			**155**	**159**	**9.3%**	**0.81 [0.71, 0.91]**
Heterogeneity: Not applicable						
Test for overall effect: Z = 3.47 (P = 0.0005)						
**1.60.2 2–4 Visits**						
Dalby 2000	0.56767531	0.04626165	70	69	9.5%	1.76 [1.61, 1.93]
**Subtotal (95% CI)**			**70**	**69**	**9.5%**	**1.76 [1.61, 1.93]**
Heterogeneity: Not applicable						
Test for overall effect: Z = 12.27 (P < 0.00001)						
**1.60.3 5 or More Visits**						
Markle-Reid 2010	2.41082692	0.30107939	54	55	4.6%	11.14 [6.18, 20.10]
Tinetti 1994	−0.83210697	0.06945248	153	148	9.2%	0.44 [0.38, 0.50]
Stuck 1995	0.20802991	0.04419946	215	199	9.5%	1.23 [1.13, 1.34]
Bernabei 1998	−0.43656359	0.0429571	99	100	9.5%	0.65 [0.59, 0.70]
Bouman 2008	−0.02296921	0.03816434	160	170	9.6%	0.98 [0.91, 1.05]
Pathy 1992	−0.05789343	0.03040131	150	156	9.6%	0.94 [0.89, 1.00]
Pathy 1992	−0.38036169	0.02767676	219	200	9.7%	0.68 [0.65, 0.72]
Counsell 2007	−0.0770609	0.02262275	474	477	9.7%	0.93 [0.89, 0.97]
van Rossum 1993	−0.25133501	0.02166482	292	288	9.7%	0.78 [0.75, 0.81]
**Subtotal (95% CI)**			**1816**	**1793**	**81.2%**	**0.90 [0.77, 1.06]**
Heterogeneity: Tau² = 0.06; Chi² = 391.92, df = 8 (P < 0.00001); I² = 98%						
Test for overall effect: Z = 1.28 (P = 0.20)						
**Total (95% CI)**			**2041**	**2021**	**100.0%**	**0.97 [0.82, 1.16]**
Heterogeneity: Tau² = 0.08; Chi² = 642.25, df = 10 (P < 0.00001); I² = 98%						
Test for overall effect: Z = 0.33 (P = 0.74)						
Test for subgroup differences: Chi² = 121.54, df = 2 (P < 0.00001), I² = 98.4%						

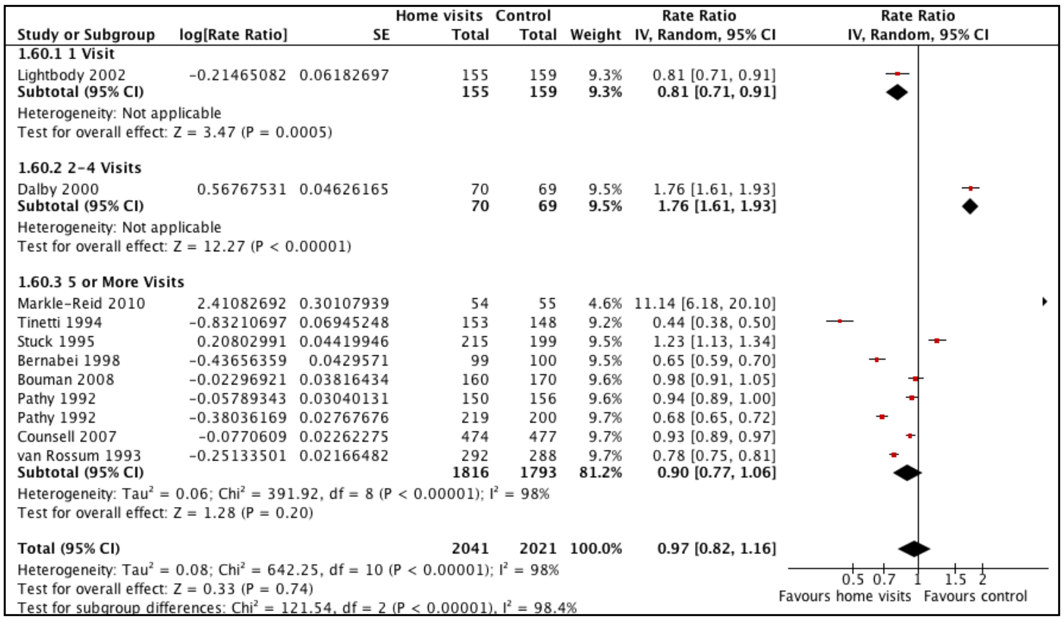

Analysis 53: Hospitalisation (people) at each follow-up interval

Study or Subgroup	Home visits Events	Home visits Total	Control Events	Control Total	Weight	Risk Ratio M-H, Random, 95% CI
**1.62.1 01-11 months**						
Ciaschini 2009	2	101	3	100	9.3%	0.66 [0.11, 3.87]
Ciechanowski 2004	15	69	21	62	90.7%	0.64 [0.36, 1.13]
**Subtotal (95% CI)**		**170**		**162**	**100.0%**	**0.64 [0.38, 1.10]**
Total events	17		24			
Heterogeneity: Tau^2 = 0.00; Chi^2 = 0.00, df = 1 (P = 0.98); I^2 = 0%						
Test for overall effect: Z = 1.60 (P = 0.11)						
**1.62.2 12-23 months**						
Kono 2004	1	59	1	60	0.3%	1.02 [0.07, 15.88]
McEwan 1990	3	135	4	122	0.9%	0.68 [0.15, 2.97]
Hogan 2001	5	79	6	84	1.5%	0.89 [0.28, 2.79]
Davison 2005	14	159	17	154	4.1%	0.80 [0.41, 1.56]
Fabacher 1994	22	100	23	95	6.5%	0.91 [0.54, 1.52]
Tinetti 1994	32	147	36	144	9.1%	0.87 [0.57, 1.32]
Stuck 1995	39	215	42	199	10.0%	0.86 [0.58, 1.27]
van Rossum 1993	45	292	64	288	12.0%	0.69 [0.49, 0.98]
Bernabei 1998	36	99	51	100	13.1%	0.71 [0.52, 0.99]
Bouman 2008	68	160	61	170	16.5%	1.18 [0.90, 1.55]
van Hout 2010	163	331	141	320	25.9%	1.12 [0.95, 1.32]
**Subtotal (95% CI)**		**1776**		**1736**	**100.0%**	**0.92 [0.80, 1.07]**
Total events	428		446			
Heterogeneity: Tau^2 = 0.01; Chi^2 = 13.53, df = 10 (P = 0.20); I^2 = 26%						
Test for overall effect: Z = 1.10 (P = 0.27)						
**1.62.3 24-35 months**						
Bouman 2008	80	160	71	170	26.8%	1.20 [0.95, 1.52]
van Rossum 1993	88	292	108	288	27.5%	0.80 [0.64, 1.01]
Sorensen 1988	345	585	482	777	45.7%	0.95 [0.87, 1.04]
**Subtotal (95% CI)**		**1037**		**1235**	**100.0%**	**0.97 [0.81, 1.15]**
Total events	513		661			
Heterogeneity: Tau^2 = 0.02; Chi^2 = 5.72, df = 2 (P = 0.06); I^2 = 65%						
Test for overall effect: Z = 0.39 (P = 0.70)						
**1.62.4 36+ months**						
Stuck 1995	99	215	93	199	22.4%	0.99 [0.80, 1.21]
van Rossum 1993	121	292	133	288	28.2%	0.90 [0.75, 1.08]
Byles 2004	261	636	198	446	49.4%	0.92 [0.80, 1.06]
**Subtotal (95% CI)**		**1143**		**933**	**100.0%**	**0.93 [0.84, 1.03]**
Total events	481		424			
Heterogeneity: Tau^2 = 0.00; Chi^2 = 0.45, df = 2 (P = 0.80); I^2 = 0%						
Test for overall effect: Z = 1.45 (P = 0.15)						

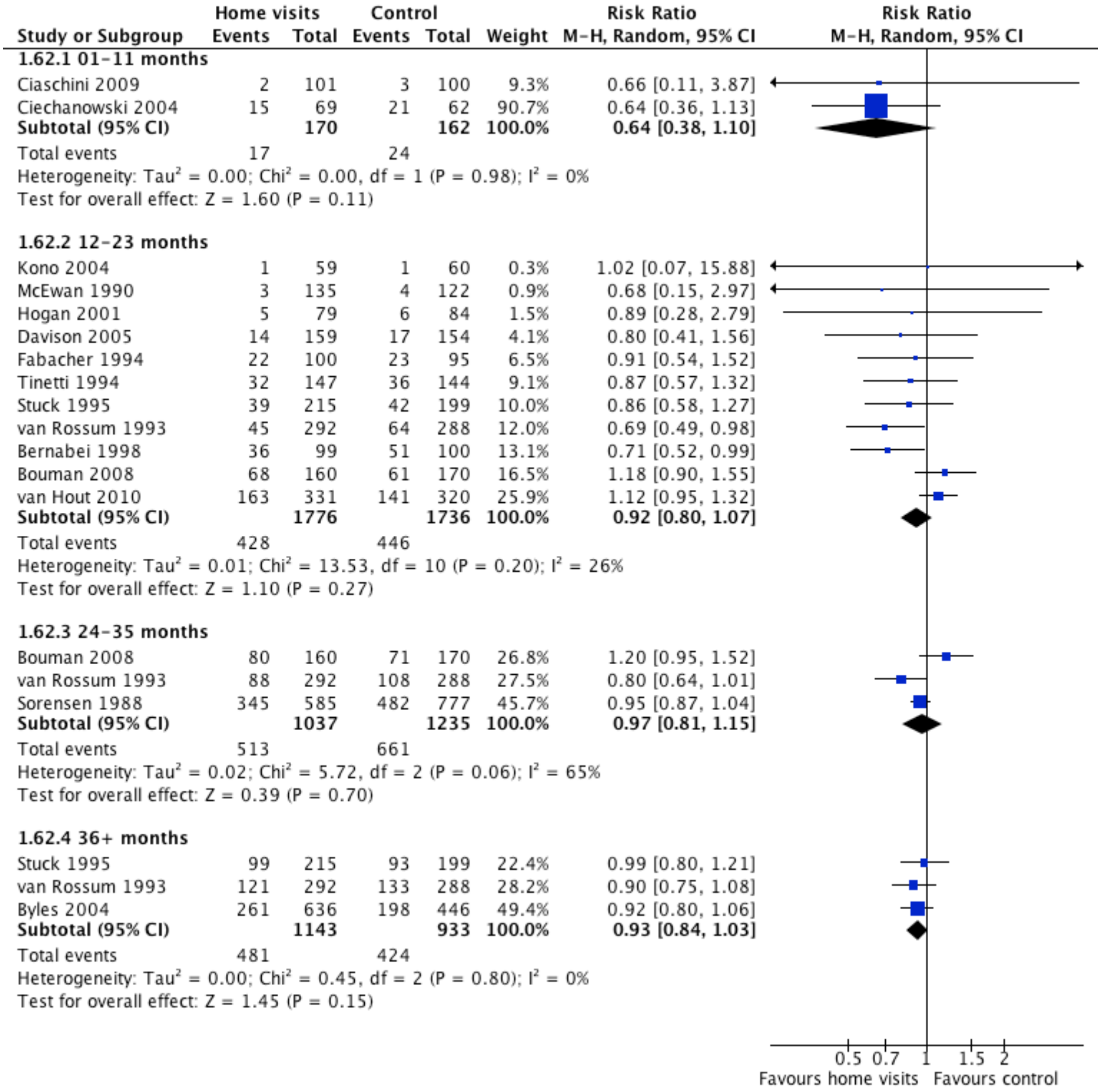

Analysis 54: Hospitalisation (people); focus of visit subgroups

Study or Subgroup	Home visits Events	Home visits Total	Control Events	Control Total	Weight	Risk Ratio M-H, Random, 95% CI
**1.63.1 Falls Only**						
Hogan 2001	5	79	6	84	0.2%	0.89 [0.28, 2.79]
**Subtotal (95% CI)**		**79**		**84**	**0.2%**	**0.89 [0.28, 2.79]**
Total events	5		6			
Heterogeneity: Not applicable						
Test for overall effect: Z = 0.21 (P = 0.84)						
**1.63.2 MGA Only**						
Kono 2004	1	59	1	60	0.0%	1.02 [0.07, 15.88]
McEwan 1990	3	135	4	122	0.1%	0.68 [0.15, 2.97]
Bernabei 1998	36	99	51	100	3.0%	0.71 [0.52, 0.99]
Bouman 2008	80	160	71	170	5.7%	1.20 [0.95, 1.52]
Stuck 1995	99	215	93	199	7.4%	0.99 [0.80, 1.21]
van Rossum 1993	121	292	133	288	9.3%	0.90 [0.75, 1.08]
Byles 2004	261	636	198	446	16.2%	0.92 [0.80, 1.06]
Sorensen 1988	345	585	482	777	41.6%	0.95 [0.87, 1.04]
**Subtotal (95% CI)**		**2181**		**2162**	**83.3%**	**0.95 [0.88, 1.01]**
Total events	946		1033			
Heterogeneity: $Tau^2 = 0.00$; $Chi^2 = 7.55$, df = 7 (P = 0.37); $I^2 = 7\%$						
Test for overall effect: Z = 1.57 (P = 0.12)						
**1.63.3 Both**						
Ciaschini 2009	2	101	3	100	0.1%	0.66 [0.11, 3.87]
Davison 2005	14	159	17	154	0.7%	0.80 [0.41, 1.56]
Fabacher 1994	22	100	23	95	1.2%	0.91 [0.54, 1.52]
Tinetti 1994	32	147	36	144	1.8%	0.87 [0.57, 1.32]
van Hout 2010	163	331	141	320	11.6%	1.12 [0.95, 1.32]
**Subtotal (95% CI)**		**838**		**813**	**15.4%**	**1.05 [0.91, 1.21]**
Total events	233		220			
Heterogeneity: $Tau^2 = 0.00$; $Chi^2 = 2.63$, df = 4 (P = 0.62); $I^2 = 0\%$						
Test for overall effect: Z = 0.64 (P = 0.52)						
**1.63.4 Neither**						
Ciechanowski 2004	15	69	21	62	1.0%	0.64 [0.36, 1.13]
**Subtotal (95% CI)**		**69**		**62**	**1.0%**	**0.64 [0.36, 1.13]**
Total events	15		21			
Heterogeneity: Not applicable						
Test for overall effect: Z = 1.53 (P = 0.13)						
**Total (95% CI)**		**3167**		**3121**	**100.0%**	**0.96 [0.91, 1.01]**
Total events	1199		1280			
Heterogeneity: $Tau^2 = 0.00$; $Chi^2 = 13.70$, df = 14 (P = 0.47); $I^2 = 0\%$						
Test for overall effect: Z = 1.49 (P = 0.14)						
Test for subgroup differences: $Chi^2 = 3.56$, df = 3 (P = 0.31), $I^2 = 15.8\%$						

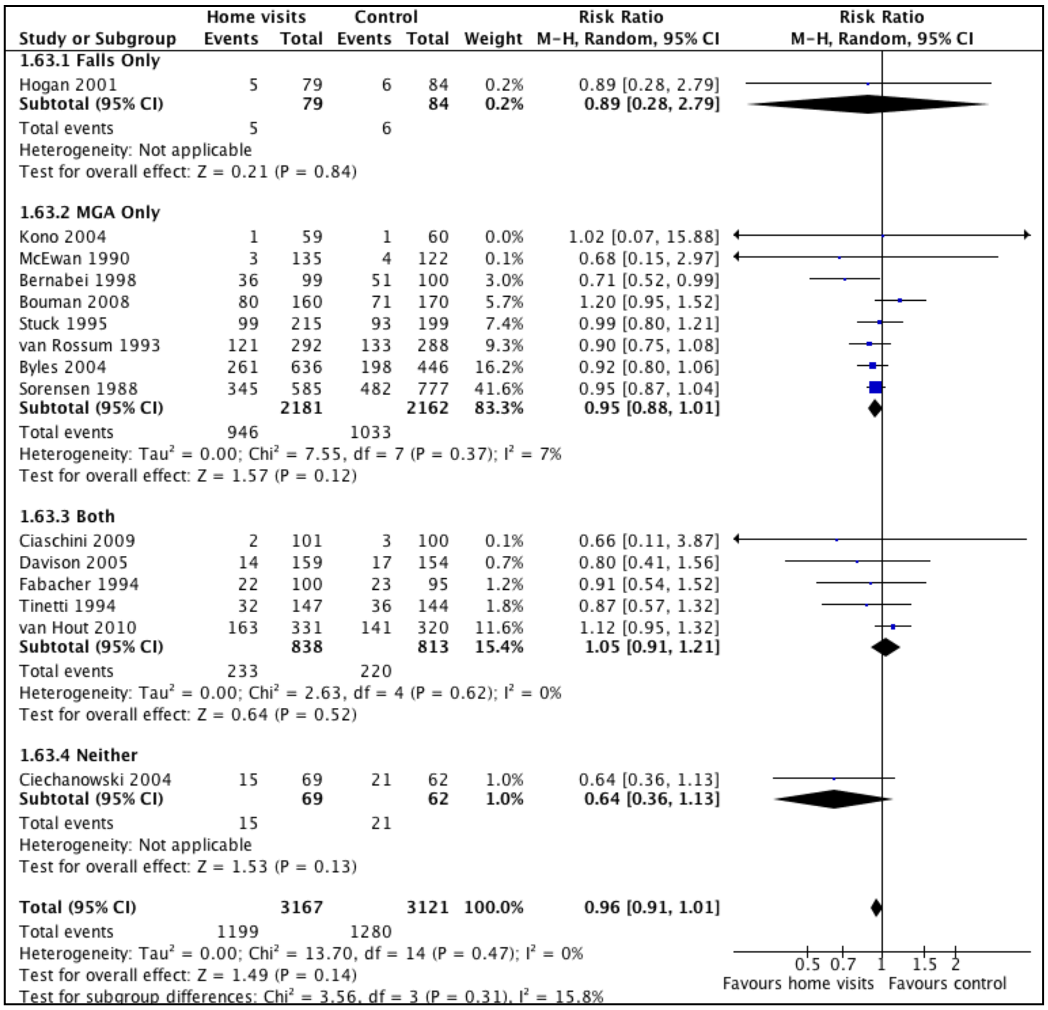

Analysis 55: Hospitalisation (people); age of participants subgroups

Study or Subgroup	Home visits Events	Home visits Total	Control Events	Control Total	Weight	Risk Ratio M-H, Random, 95% CI
**1.64.2 71-75**						
Ciaschini 2009	2	101	3	100	0.1%	0.66 [0.11, 3.87]
Ciechanowski 2004	15	69	21	62	1.1%	0.64 [0.36, 1.13]
Fabacher 1994	22	100	23	95	1.4%	0.91 [0.54, 1.52]
**Subtotal (95% CI)**		**270**		**257**	**2.6%**	**0.77 [0.53, 1.12]**
Total events	39		47			
Heterogeneity: Tau² = 0.00; Chi² = 0.83, df = 2 (P = 0.66); I² = 0%						
Test for overall effect: Z = 1.37 (P = 0.17)						
**1.64.3 76-80**						
Hogan 2001	5	79	6	84	0.3%	0.89 [0.28, 2.79]
Davison 2005	14	159	17	154	0.8%	0.80 [0.41, 1.56]
Tinetti 1994	32	147	36	144	2.0%	0.87 [0.57, 1.32]
Bouman 2008	80	160	71	170	6.3%	1.20 [0.95, 1.52]
van Rossum 1993	121	292	133	288	10.0%	0.90 [0.75, 1.08]
Byles 2004	261	636	198	446	16.8%	0.92 [0.80, 1.06]
Sorensen 1988	345	585	482	777	37.6%	0.95 [0.87, 1.04]
**Subtotal (95% CI)**		**2058**		**2063**	**73.7%**	**0.95 [0.89, 1.01]**
Total events	858		943			
Heterogeneity: Tau² = 0.00; Chi² = 4.66, df = 6 (P = 0.59); I² = 0%						
Test for overall effect: Z = 1.52 (P = 0.13)						
**1.64.4 81-85**						
Kono 2004	1	59	1	60	0.0%	1.02 [0.07, 15.88]
Bernabei 1998	36	99	51	100	3.4%	0.71 [0.52, 0.99]
Stuck 1995	99	215	93	199	8.0%	0.99 [0.80, 1.21]
van Hout 2010	163	331	141	320	12.3%	1.12 [0.95, 1.32]
**Subtotal (95% CI)**		**704**		**679**	**23.8%**	**0.96 [0.79, 1.18]**
Total events	299		286			
Heterogeneity: Tau² = 0.02; Chi² = 5.95, df = 3 (P = 0.11); I² = 50%						
Test for overall effect: Z = 0.36 (P = 0.72)						
**Total (95% CI)**		**3032**		**2999**	**100.0%**	**0.96 [0.90, 1.02]**
Total events	1196		1276			
Heterogeneity: Tau² = 0.00; Chi² = 13.48, df = 13 (P = 0.41); I² = 4%						
Test for overall effect: Z = 1.38 (P = 0.17)						
Test for subgroup differences: Chi² = 1.21, df = 2 (P = 0.55), I² = 0%						

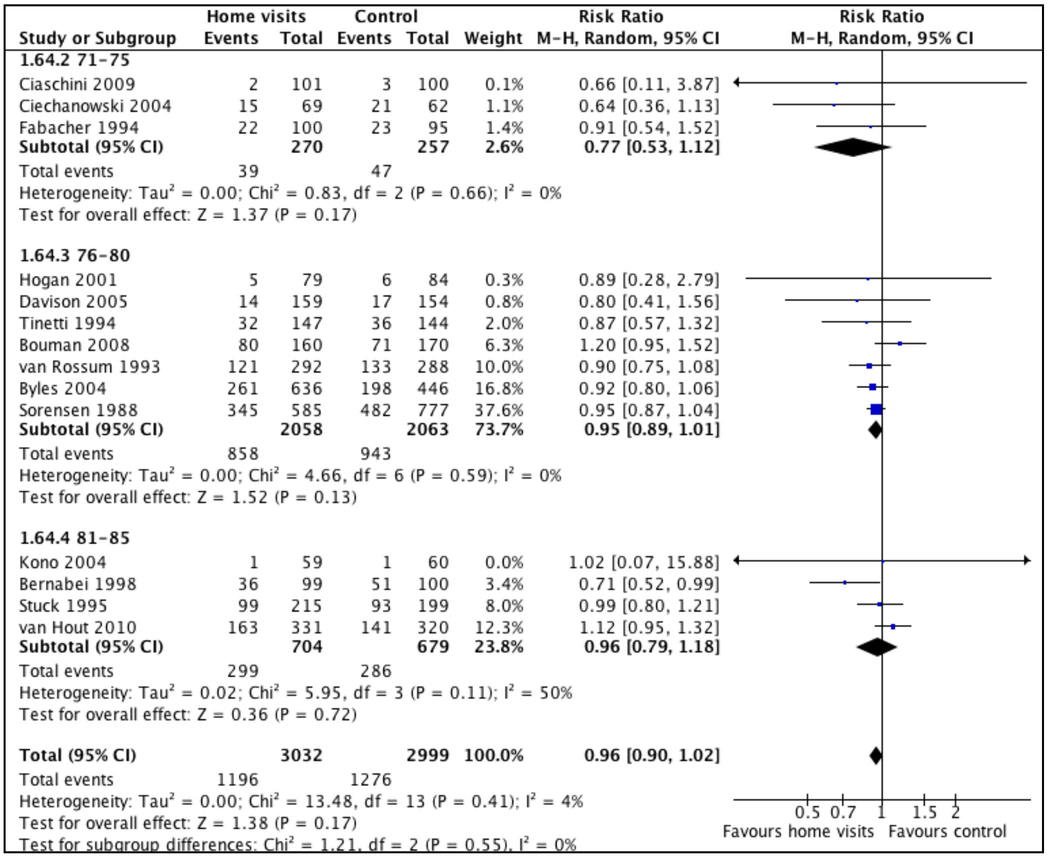

Analysis 56: Hospitalisation (people); type of visitor subgroups

Study or Subgroup	Home visits Events	Home visits Total	Control Events	Control Total	Weight	Risk Ratio M-H, Random, 95% CI
**1.65.1 Nurse**						
Kono 2004	1	59	1	60	0.0%	1.02 [0.07, 15.88]
Ciaschini 2009	2	101	3	100	0.1%	0.66 [0.11, 3.87]
McEwan 1990	3	135	4	122	0.1%	0.68 [0.15, 2.97]
Bouman 2008	80	160	71	170	5.7%	1.20 [0.95, 1.52]
Stuck 1995	99	215	93	199	7.4%	0.99 [0.80, 1.21]
van Rossum 1993	121	292	133	288	9.3%	0.90 [0.75, 1.08]
van Hout 2010	163	331	141	320	11.6%	1.12 [0.95, 1.32]
**Subtotal (95% CI)**		**1293**		**1259**	**34.2%**	**1.03 [0.94, 1.14]**
Total events	469		446			
Heterogeneity: Tau² = 0.00; Chi² = 5.38, df = 6 (P = 0.50); I² = 0%						
Test for overall effect: Z = 0.66 (P = 0.51)						
**1.65.2 Other**						
Ciechanowski 2004	15	69	21	62	1.0%	0.64 [0.36, 1.13]
Bernabei 1998	36	99	51	100	3.0%	0.71 [0.52, 0.99]
**Subtotal (95% CI)**		**168**		**162**	**4.0%**	**0.69 [0.52, 0.92]**
Total events	51		72			
Heterogeneity: Tau² = 0.00; Chi² = 0.10, df = 1 (P = 0.75); I² = 0%						
Test for overall effect: Z = 2.54 (P = 0.01)						
**1.65.3 Combined**						
Hogan 2001	5	79	6	84	0.2%	0.89 [0.28, 2.79]
Davison 2005	14	159	17	154	0.7%	0.80 [0.41, 1.56]
Fabacher 1994	22	100	23	95	1.2%	0.91 [0.54, 1.52]
Tinetti 1994	32	147	36	144	1.8%	0.87 [0.57, 1.32]
Byles 2004	261	636	198	446	16.2%	0.92 [0.80, 1.06]
Sorensen 1988	345	585	482	777	41.6%	0.95 [0.87, 1.04]
**Subtotal (95% CI)**		**1706**		**1700**	**61.8%**	**0.94 [0.87, 1.01]**
Total events	679		762			
Heterogeneity: Tau² = 0.00; Chi² = 0.53, df = 5 (P = 0.99); I² = 0%						
Test for overall effect: Z = 1.75 (P = 0.08)						
**Total (95% CI)**		**3167**		**3121**	**100.0%**	**0.96 [0.91, 1.01]**
Total events	1199		1280			
Heterogeneity: Tau² = 0.00; Chi² = 13.70, df = 14 (P = 0.47); I² = 0%						
Test for overall effect: Z = 1.49 (P = 0.14)						
Test for subgroup differences: Chi² = 7.69, df = 2 (P = 0.02), I² = 74.0%						

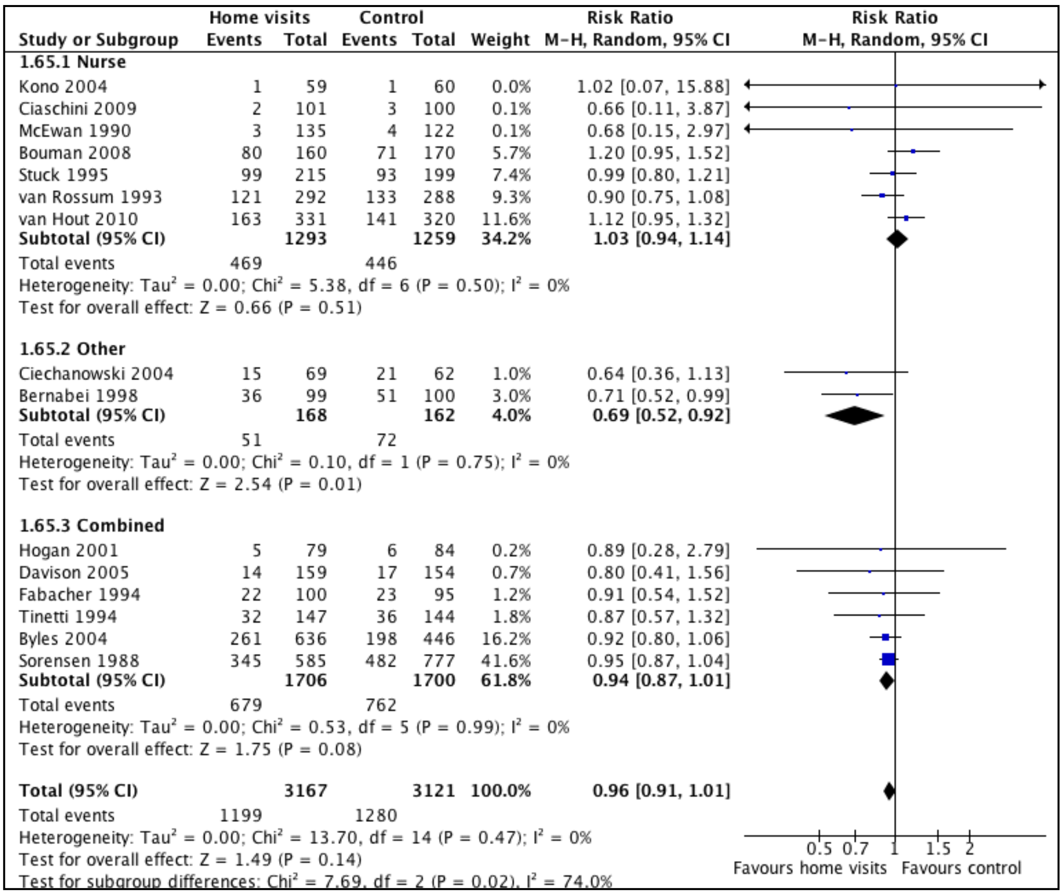

Analysis 57: Hospitalisation (people); number of visits subgroups

Study or Subgroup	Home visits Events	Home visits Total	Control Events	Control Total	Weight	Risk Ratio M-H, Random, 95% CI
**1.66.1 1 Visit**						
McEwan 1990	3	135	4	122	0.2%	0.68 [0.15, 2.97]
Sorensen 1988	345	585	482	777	33.4%	0.95 [0.87, 1.04]
**Subtotal (95% CI)**		**720**		**899**	**33.6%**	**0.95 [0.87, 1.04]**
Total events	348		486			
Heterogeneity: Tau² = 0.00; Chi² = 0.20, df = 1 (P = 0.65); I² = 0%						
Test for overall effect: Z = 1.17 (P = 0.24)						
**1.66.2 2-4 Visits**						
Kono 2004	1	59	1	60	0.1%	1.02 [0.07, 15.88]
Hogan 2001	5	79	6	84	0.3%	0.89 [0.28, 2.79]
Fabacher 1994	22	100	23	95	1.6%	0.91 [0.54, 1.52]
van Hout 2010	163	331	141	320	13.1%	1.12 [0.95, 1.32]
Byles 2004	261	636	198	446	17.2%	0.92 [0.80, 1.06]
**Subtotal (95% CI)**		**1205**		**1005**	**32.3%**	**1.00 [0.90, 1.10]**
Total events	452		369			
Heterogeneity: Tau² = 0.00; Chi² = 3.14, df = 4 (P = 0.53); I² = 0%						
Test for overall effect: Z = 0.08 (P = 0.93)						
**1.66.3 5 or More Visits**						
Ciechanowski 2004	15	69	21	62	1.3%	0.64 [0.36, 1.13]
Tinetti 1994	32	147	36	144	2.4%	0.87 [0.57, 1.32]
Bernabei 1998	36	99	51	100	3.9%	0.71 [0.52, 0.99]
Bouman 2008	80	160	71	170	7.0%	1.20 [0.95, 1.52]
Stuck 1995	99	215	93	199	8.8%	0.99 [0.80, 1.21]
van Rossum 1993	121	292	133	288	10.8%	0.90 [0.75, 1.08]
**Subtotal (95% CI)**		**982**		**963**	**34.1%**	**0.92 [0.79, 1.07]**
Total events	383		405			
Heterogeneity: Tau² = 0.02; Chi² = 9.19, df = 5 (P = 0.10); I² = 46%						
Test for overall effect: Z = 1.07 (P = 0.28)						
**Total (95% CI)**		**2907**		**2867**	**100.0%**	**0.96 [0.90, 1.02]**
Total events	1183		1260			
Heterogeneity: Tau² = 0.00; Chi² = 13.22, df = 12 (P = 0.35); I² = 9%						
Test for overall effect: Z = 1.23 (P = 0.22)						
Test for subgroup differences: Chi² = 0.84, df = 2 (P = 0.66), I² = 0%						

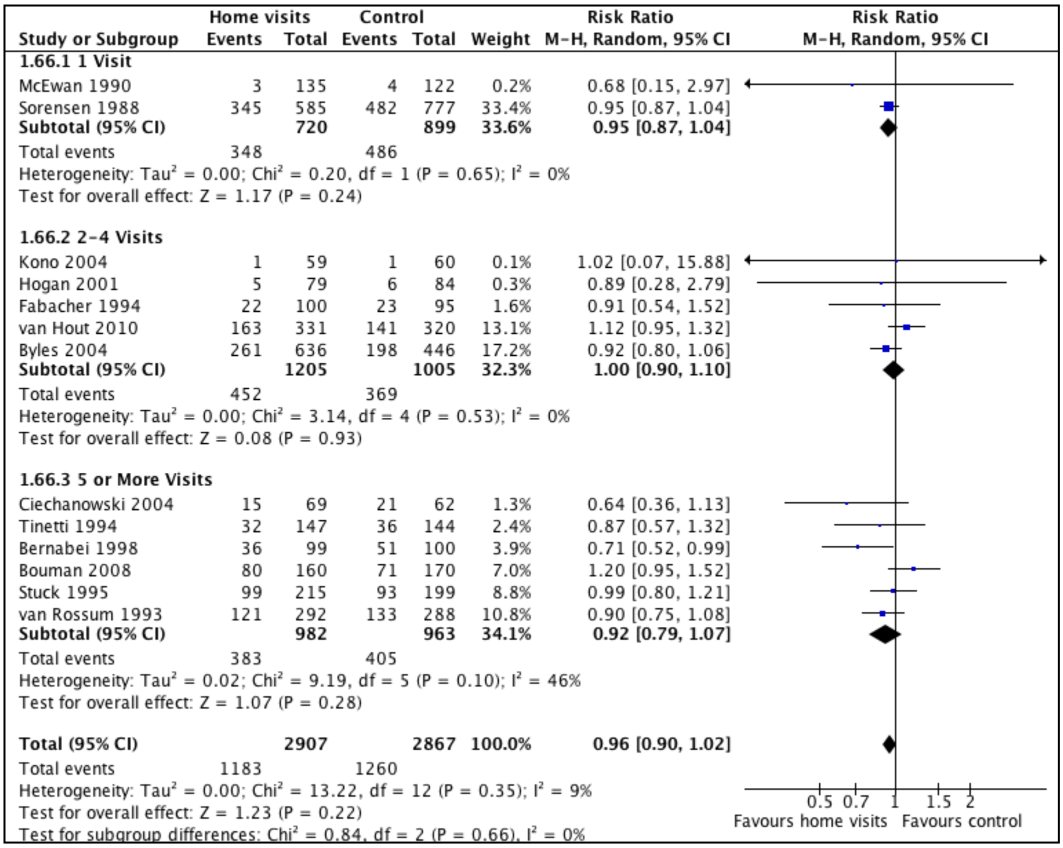

Analysis 58: Injuries (number), results at longest follow-up

Study or Subgroup	log[Rate Ratio]	SE	Home visits Total	Control Total	Weight	Rate Ratio IV, Random, 95% CI
Tinetti 1994	−0.35254271	0.36397614	147	144	2.9%	0.70 [0.34, 1.43]
van Haastregt 2000	0.25131443	0.26514719	159	157	5.4%	1.29 [0.76, 2.16]
Robertson 2001a	−0.18993339	0.21025423	121	119	8.6%	0.83 [0.55, 1.25]
Luukinen 2006	−0.05406722	0.19269937	243	243	10.3%	0.95 [0.65, 1.38]
Campbell 2005	−0.07302514	0.17609464	98	96	12.3%	0.93 [0.66, 1.31]
Stevens 2001	−0.08338161	0.11371102	635	1244	29.5%	0.92 [0.74, 1.15]
Elley 2008	0.10878192	0.11087204	155	157	31.0%	1.11 [0.90, 1.39]
**Total (95% CI)**			**1558**	**2160**	**100.0%**	**0.98 [0.87, 1.11]**

Heterogeneity: Tau² = 0.00; Chi² = 4.32, df = 6 (P = 0.63); I² = 0%
Test for overall effect: Z = 0.30 (P = 0.77)

Rate Ratio IV, Random, 95% CI

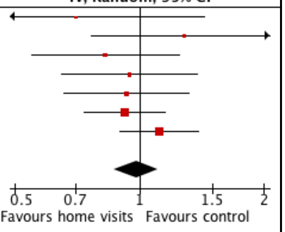

Analysis 59: Injuries (people), results at longest follow-up

Study or Subgroup	log[Risk Ratio]	SE	Home visits Total	Control Total	Weight	Risk Ratio IV, Random, 95% CI
Ciaschini 2009	−1.8017098	1.07087146	101	100	1.0%	0.17 [0.02, 1.35]
Hogan 2001	−0.44945668	0.71328139	79	84	2.2%	0.64 [0.16, 2.58]
Davison 2005	−0.65099081	0.45496967	159	154	5.4%	0.52 [0.21, 1.27]
Close 1999	−0.54679077	0.42115363	184	213	6.3%	0.58 [0.25, 1.32]
Tinetti 1994	−0.17476997	0.37551929	147	144	7.9%	0.84 [0.40, 1.75]
Vetter 1992	0	0.35355339	240	210	8.9%	1.00 [0.50, 2.00]
Wyman 2007	−0.24370983	0.33432736	131	132	9.9%	0.78 [0.41, 1.51]
Hendriks 2008	−0.27911671	0.3254249	124	134	10.5%	0.76 [0.40, 1.43]
Luukinen 2006 (1)	0.0861777	0.22333797	243	243	22.3%	1.09 [0.70, 1.69]
Campbell 1999	−0.45677951	0.20766596	116	117	25.8%	0.63 [0.42, 0.95]
**Total (95% CI)**			**1524**	**1531**	**100.0%**	**0.77 [0.63, 0.95]**

Heterogeneity: Tau² = 0.00; Chi² = 7.24, df = 9 (P = 0.61); I² = 0%
Test for overall effect: Z = 2.49 (P = 0.01)

(1) Hazard Ratio

Risk Ratio IV, Random, 95% CI

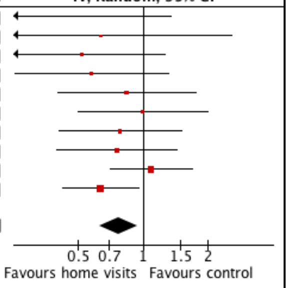

Analysis 60: Injuries (people) at each follow-up interval

Study or Subgroup	log[Risk Ratio]	SE	Home visits Total	Control Total	Weight	Risk Ratio IV, Random, 95% CI
**1.74.1 01-11 months**						
Ciaschini 2009	−1.8017098	1.07087146	101	100	29.5%	0.17 [0.02, 1.35]
Hendriks 2008	−0.24882493	0.39598229	131	143	70.5%	0.78 [0.36, 1.69]
**Subtotal (95% CI)**			**232**	**243**	**100.0%**	**0.49 [0.12, 1.98]**
Heterogeneity: Tau² = 0.55; Chi² = 1.85, df = 1 (P = 0.17); I² = 46%						
Test for overall effect: Z = 1.00 (P = 0.32)						
**1.74.2 12-23 months**						
Hogan 2001	−0.44945668	0.71328139	79	84	2.4%	0.64 [0.16, 2.58]
Davison 2005	−0.65099081	0.45496967	159	154	6.0%	0.52 [0.21, 1.27]
Close 1999	−0.54679077	0.42115363	184	213	6.9%	0.58 [0.25, 1.32]
Tinetti 1994	−0.17476997	0.37551929	147	144	8.7%	0.84 [0.40, 1.75]
Wyman 2007	−0.24370983	0.33432736	131	132	11.0%	0.78 [0.41, 1.51]
Hendriks 2008	−0.27911671	0.3254249	124	134	11.6%	0.76 [0.40, 1.43]
Luukinen 2006	0.0861777	0.22333797	243	243	24.7%	1.09 [0.70, 1.69]
Campbell 1999	−0.45677951	0.20766596	116	117	28.6%	0.63 [0.42, 0.95]
**Subtotal (95% CI)**			**1183**	**1221**	**100.0%**	**0.76 [0.61, 0.95]**
Heterogeneity: Tau² = 0.00; Chi² = 4.62, df = 7 (P = 0.71); I² = 0%						
Test for overall effect: Z = 2.45 (P = 0.01)						
**1.74.4 36+ months**						
Vetter 1992	0	0.35355339	240	210	100.0%	1.00 [0.50, 2.00]
**Subtotal (95% CI)**			**240**	**210**	**100.0%**	**1.00 [0.50, 2.00]**
Heterogeneity: Not applicable						
Test for overall effect: Z = 0.00 (P = 1.00)						

Risk Ratio IV, Random, 95% CI

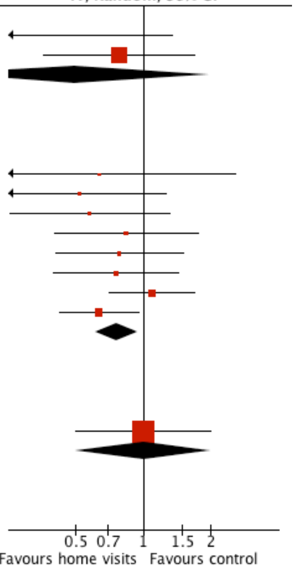

Analysis 61: Injuries (people); focus of visit subgroups

Study or Subgroup	log[Risk Ratio]	SE	Home visits Total	Control Total	Weight	Risk Ratio IV, Random, 95% CI
**1.75.1 Falls Only**						
Hogan 2001	−0.44945668	0.71328139	79	84	2.2%	0.64 [0.16, 2.58]
Wyman 2007	−0.24370983	0.33432736	131	132	9.9%	0.78 [0.41, 1.51]
Luukinen 2006	0.0861777	0.22333797	243	243	22.3%	1.09 [0.70, 1.69]
Campbell 1999	−0.45677951	0.20766596	116	117	25.8%	0.63 [0.42, 0.95]
**Subtotal (95% CI)**			**569**	**576**	**60.1%**	**0.80 [0.60, 1.07]**
Heterogeneity: Tau² = 0.01; Chi² = 3.29, df = 3 (P = 0.35); I² = 9%						
Test for overall effect: Z = 1.51 (P = 0.13)						
**1.75.3 Both**						
Ciaschini 2009	−1.8017098	1.07087146	101	100	1.0%	0.17 [0.02, 1.35]
Davison 2005	−0.65099081	0.45496967	159	154	5.4%	0.52 [0.21, 1.27]
Close 1999	−0.54679077	0.42115363	184	213	6.3%	0.58 [0.25, 1.32]
Tinetti 1994	−0.17476997	0.37551929	147	144	7.9%	0.84 [0.40, 1.75]
Vetter 1992	0	0.35355339	240	210	8.9%	1.00 [0.50, 2.00]
Hendriks 2008	−0.27911671	0.3254249	124	134	10.5%	0.76 [0.40, 1.43]
**Subtotal (95% CI)**			**955**	**955**	**39.9%**	**0.72 [0.52, 1.00]**
Heterogeneity: Tau² = 0.00; Chi² = 3.72, df = 5 (P = 0.59); I² = 0%						
Test for overall effect: Z = 1.95 (P = 0.05)						
**Total (95% CI)**			**1524**	**1531**	**100.0%**	**0.77 [0.63, 0.95]**
Heterogeneity: Tau² = 0.00; Chi² = 7.24, df = 9 (P = 0.61); I² = 0%						
Test for overall effect: Z = 2.49 (P = 0.01)						
Test for subgroup differences: Chi² = 0.23, df = 1 (P = 0.63), I² = 0%						

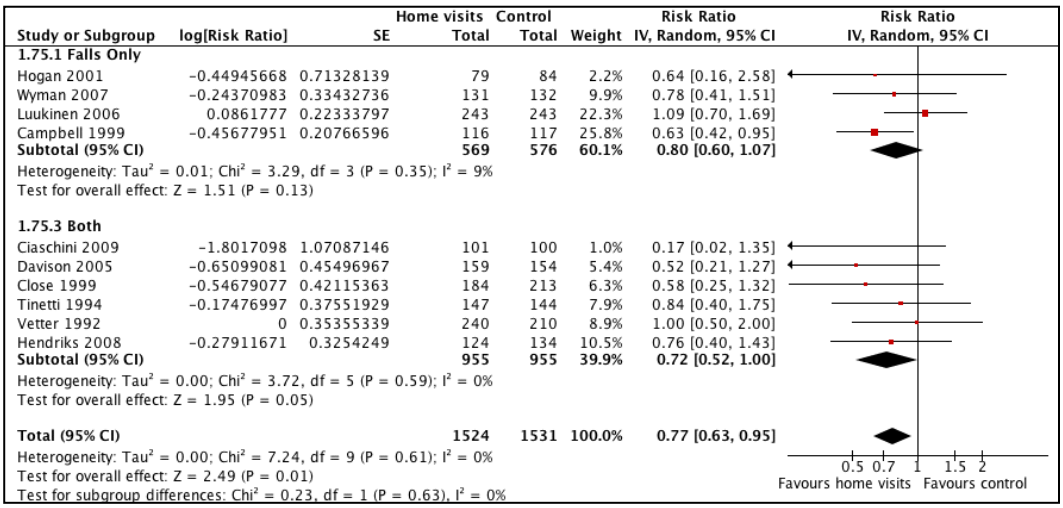

Analysis 62: Injuries (people); age of participants subgroups

Study or Subgroup	log[Risk Ratio]	SE	Home visits Total	Control Total	Weight	Risk Ratio IV, Random, 95% CI
**1.76.2 71-75**						
Ciaschini 2009	−1.8017098	1.07087146	101	100	1.0%	0.17 [0.02, 1.35]
Hendriks 2008	−0.27911671	0.3254249	124	134	10.5%	0.76 [0.40, 1.43]
**Subtotal (95% CI)**			**225**	**234**	**11.5%**	**0.50 [0.13, 1.89]**
Heterogeneity: Tau² = 0.53; Chi² = 1.85, df = 1 (P = 0.17); I² = 46%						
Test for overall effect: Z = 1.03 (P = 0.30)						
**1.76.3 76-80**						
Hogan 2001	−0.44945668	0.71328139	79	84	2.2%	0.64 [0.16, 2.58]
Davison 2005	−0.65099081	0.45496967	159	154	5.4%	0.52 [0.21, 1.27]
Close 1999	−0.54679077	0.42115363	184	213	6.3%	0.58 [0.25, 1.32]
Tinetti 1994	−0.17476997	0.37551929	147	144	7.9%	0.84 [0.40, 1.75]
Vetter 1992	0	0.35355339	240	210	8.9%	1.00 [0.50, 2.00]
Wyman 2007	−0.24370983	0.33432736	131	132	9.9%	0.78 [0.41, 1.51]
**Subtotal (95% CI)**			**940**	**937**	**40.5%**	**0.75 [0.54, 1.04]**
Heterogeneity: Tau² = 0.00; Chi² = 1.84, df = 5 (P = 0.87); I² = 0%						
Test for overall effect: Z = 1.74 (P = 0.08)						
**1.76.4 81-85**						
Campbell 1999	−0.45677951	0.20766596	116	117	25.8%	0.63 [0.42, 0.95]
**Subtotal (95% CI)**			**116**	**117**	**25.8%**	**0.63 [0.42, 0.95]**
Heterogeneity: Not applicable						
Test for overall effect: Z = 2.20 (P = 0.03)						
**1.76.5 86+**						
Luukinen 2006	0.0861777	0.22333797	243	243	22.3%	1.09 [0.70, 1.69]
**Subtotal (95% CI)**			**243**	**243**	**22.3%**	**1.09 [0.70, 1.69]**
Heterogeneity: Not applicable						
Test for overall effect: Z = 0.39 (P = 0.70)						
**Total (95% CI)**			**1524**	**1531**	**100.0%**	**0.77 [0.63, 0.95]**
Heterogeneity: Tau² = 0.00; Chi² = 7.24, df = 9 (P = 0.61); I² = 0%						
Test for overall effect: Z = 2.49 (P = 0.01)						
Test for subgroup differences: Chi² = 3.74, df = 3 (P = 0.29), I² = 19.9%						

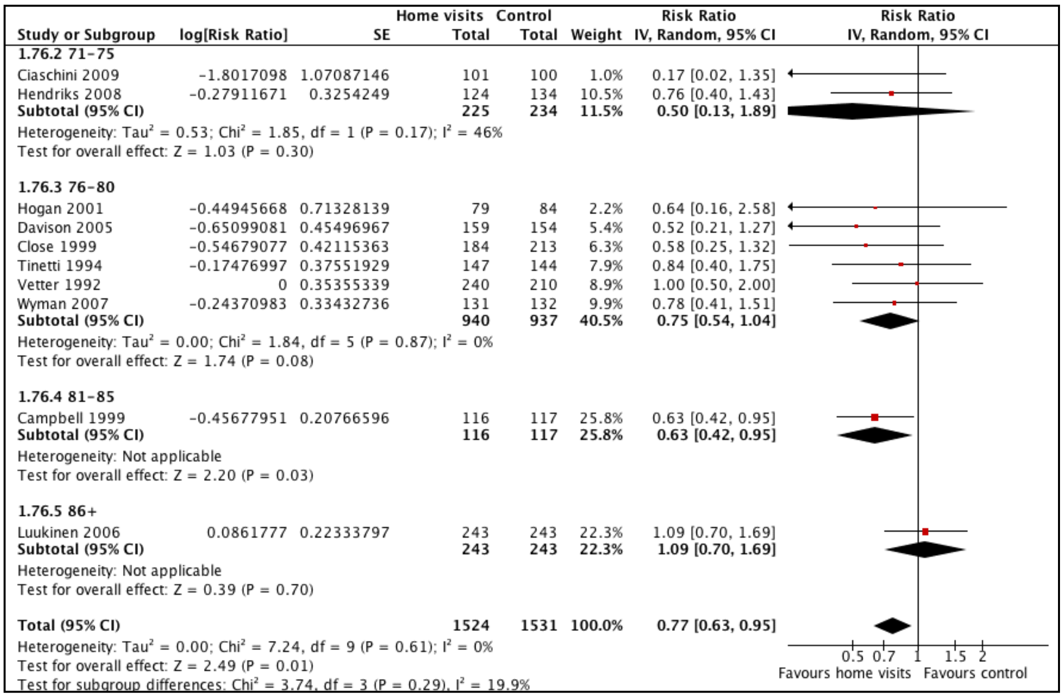

Analysis 63: Injuries (people); type of visitor subgroups

Study or Subgroup	log[Rate Ratio]	SE	Home visits Total	Control Total	Weight	Rate Ratio IV, Random, 95% CI
**1.77.1 Nurse**						
Ciaschini 2009	−1.8017098	1.07087146	101	100	1.0%	0.17 [0.02, 1.35]
Wyman 2007	−0.24370983	0.33432736	131	132	9.9%	0.78 [0.41, 1.51]
**Subtotal (95% CI)**			**232**	**232**	**10.9%**	**0.50 [0.13, 1.99]**
Heterogeneity: Tau² = 0.58; Chi² = 1.93, df = 1 (P = 0.16); I² = 48%						
Test for overall effect: Z = 0.98 (P = 0.33)						
**1.77.2 Other**						
Close 1999	−0.54679077	0.42115363	184	213	6.3%	0.58 [0.25, 1.32]
Vetter 1992	0	0.35355339	240	210	8.9%	1.00 [0.50, 2.00]
Campbell 1999	−0.45677951	0.20766596	116	117	25.8%	0.63 [0.42, 0.95]
**Subtotal (95% CI)**			**540**	**540**	**40.9%**	**0.69 [0.50, 0.95]**
Heterogeneity: Tau² = 0.00; Chi² = 1.45, df = 2 (P = 0.49); I² = 0%						
Test for overall effect: Z = 2.25 (P = 0.02)						
**1.77.3 Combined**						
Hogan 2001	−0.44945668	0.71328139	79	84	2.2%	0.64 [0.16, 2.58]
Davison 2005	−0.65099081	0.45496967	159	154	5.4%	0.52 [0.21, 1.27]
Tinetti 1994	−0.17476997	0.37551929	147	144	7.9%	0.84 [0.40, 1.75]
Hendriks 2008	−0.27911671	0.3254249	124	134	10.5%	0.76 [0.40, 1.43]
Luukinen 2006 (1)	0.0861777	0.22333797	243	243	22.3%	1.09 [0.70, 1.69]
**Subtotal (95% CI)**			**752**	**759**	**48.2%**	**0.87 [0.64, 1.17]**
Heterogeneity: Tau² = 0.00; Chi² = 2.67, df = 4 (P = 0.62); I² = 0%						
Test for overall effect: Z = 0.94 (P = 0.35)						
**Total (95% CI)**			**1524**	**1531**	**100.0%**	**0.77 [0.63, 0.95]**
Heterogeneity: Tau² = 0.00; Chi² = 7.24, df = 9 (P = 0.61); I² = 0%						
Test for overall effect: Z = 2.49 (P = 0.01)						
Test for subgroup differences: Chi² = 1.43, df = 2 (P = 0.49), I² = 0%						

(1) Hazard Ratio

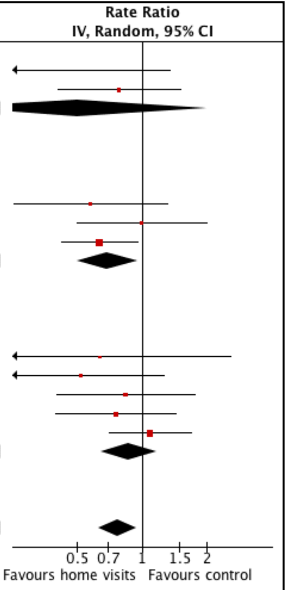

Analysis 64: Injuries (people); number of visits subgroups

Study or Subgroup	log[Risk Ratio]	SE	Home visits Total	Control Total	Weight	Risk Ratio IV, Random, 95% CI
**1.78.1 1 Visit**						
Close 1999	−0.54679077	0.42115363	184	213	6.7%	0.58 [0.25, 1.32]
Hendriks 2008	−0.27911671	0.3254249	124	134	11.2%	0.76 [0.40, 1.43]
**Subtotal (95% CI)**			**308**	**347**	**17.9%**	**0.68 [0.41, 1.13]**
Heterogeneity: Tau² = 0.00; Chi² = 0.25, df = 1 (P = 0.62); I² = 0%						
Test for overall effect: Z = 1.47 (P = 0.14)						
**1.78.2 2-4 Visits**						
Hogan 2001	−0.44945668	0.71328139	79	84	2.3%	0.64 [0.16, 2.58]
Vetter 1992	0	0.35355339	240	210	9.5%	1.00 [0.50, 2.00]
Luukinen 2006	0.0861777	0.22333797	243	243	23.8%	1.09 [0.70, 1.69]
Campbell 1999	−0.45677951	0.20766596	116	117	27.5%	0.63 [0.42, 0.95]
**Subtotal (95% CI)**			**678**	**654**	**63.1%**	**0.84 [0.62, 1.14]**
Heterogeneity: Tau² = 0.02; Chi² = 3.60, df = 3 (P = 0.31); I² = 17%						
Test for overall effect: Z = 1.12 (P = 0.26)						
**1.78.3 5 or More Visits**						
Tinetti 1994	−0.17476997	0.37551929	147	144	8.4%	0.84 [0.40, 1.75]
Wyman 2007	−0.24370983	0.33432736	131	132	10.6%	0.78 [0.41, 1.51]
**Subtotal (95% CI)**			**278**	**276**	**19.0%**	**0.81 [0.50, 1.32]**
Heterogeneity: Tau² = 0.00; Chi² = 0.02, df = 1 (P = 0.89); I² = 0%						
Test for overall effect: Z = 0.85 (P = 0.39)						
**Total (95% CI)**			**1264**	**1277**	**100.0%**	**0.80 [0.65, 0.99]**
Heterogeneity: Tau² = 0.00; Chi² = 4.32, df = 7 (P = 0.74); I² = 0%						
Test for overall effect: Z = 2.06 (P = 0.04)						
Test for subgroup differences: Chi² = 0.45, df = 2 (P = 0.80), I² = 0%						

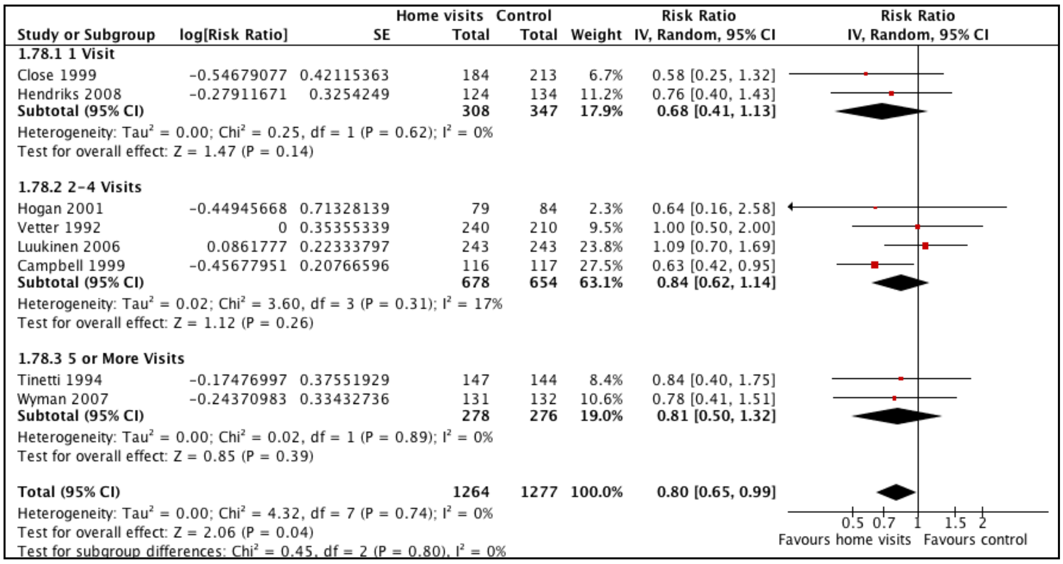

Analysis 65: Institutionalisation (days), results at longest follow-up

Study or Subgroup	log[Rate Ratio]	SE	Home visits Total	Control Total	Weight	Rate Ratio IV, Fixed, 95% CI
Fabacher 1994	0	0	100	95		Not estimable
Bouman 2008	−0.16929775	0.10792658	160	170	1.3%	0.84 [0.68, 1.04]
Bernabei 1998	−0.67379941	0.037302	99	100	11.0%	0.51 [0.47, 0.55]
Stuck 1995	−1.45636192	0.03080896	215	199	16.1%	0.23 [0.22, 0.25]
van Rossum 1993	0.08819482	0.01460808	292	288	71.6%	1.09 [1.06, 1.12]
**Total (95% CI)**			**866**	**852**	**100.0%**	**0.78 [0.76, 0.80]**
Heterogeneity: Chi² = 2198.79, df = 3 (P < 0.00001); I² = 100%						
Test for overall effect: Z = 20.02 (P < 0.00001)						

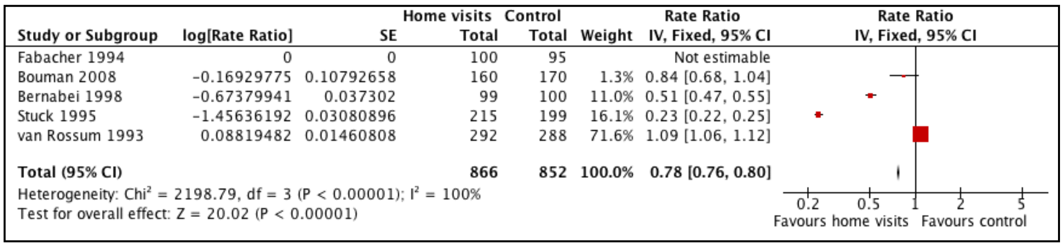

Analysis 66: Institutionalisation (people) at each follow-up interval

Study or Subgroup	Home visits Events	Home visits Total	Control Events	Control Total	Weight	Risk Ratio M-H, Random, 95% CI
**1.86.1 01-11 months**						
Lenaghan 2007	1	68	3	66	11.2%	0.32 [0.03, 3.03]
Holland 2005	37	429	32	426	88.8%	1.15 [0.73, 1.81]
**Subtotal (95% CI)**		**497**		**492**	**100.0%**	**1.00 [0.46, 2.18]**
Total events	38		35			
Heterogeneity: Tau² = 0.13; Chi² = 1.19, df = 1 (P = 0.28); I² = 16%						
Test for overall effect: Z = 0.01 (P = 0.99)						
**1.86.2 12-23 months**						
Fabacher 1994	0	100	0	95		Not estimable
Dalby 2000	0	70	1	69	0.4%	0.33 [0.01, 7.93]
Hogan 2001	2	79	1	84	0.7%	2.13 [0.20, 22.99]
Shapiro 2002	1	40	11	65	1.0%	0.15 [0.02, 1.10]
Newbury 2001	2	45	2	44	1.1%	0.98 [0.14, 6.64]
Sommers 2000	7	238	3	227	2.3%	2.23 [0.58, 8.50]
Hall 1992	3	81	8	86	2.4%	0.40 [0.11, 1.45]
Hebert 2001	5	250	5	253	2.7%	1.01 [0.30, 3.45]
Kono 2004	5	59	8	60	3.6%	0.64 [0.22, 1.83]
Yamada 2003	12	184	9	169	5.7%	1.22 [0.53, 2.83]
Kono 2011	12	161	9	162	5.8%	1.34 [0.58, 3.10]
Bernabei 1998	10	99	15	100	7.2%	0.67 [0.32, 1.43]
Close 1999	18	184	18	213	10.4%	1.16 [0.62, 2.16]
Gunner-Svensson 1984	23	1844	36	1899	15.0%	0.66 [0.39, 1.11]
Caplan 2004	32	370	28	369	17.1%	1.14 [0.70, 1.85]
van Hout 2010	43	331	39	320	24.6%	1.07 [0.71, 1.60]
**Subtotal (95% CI)**		**4135**		**4215**	**100.0%**	**0.95 [0.78, 1.17]**
Total events	175		193			
Heterogeneity: Tau² = 0.00; Chi² = 13.06, df = 14 (P = 0.52); I² = 0%						
Test for overall effect: Z = 0.47 (P = 0.64)						
**1.86.3 24-35 months**						
Hall 1992	2	81	12	86	2.6%	0.18 [0.04, 0.77]
Bouman 2008	10	160	11	170	7.5%	0.97 [0.42, 2.21]
Sommers 2000	18	238	14	227	10.5%	1.23 [0.62, 2.41]
Counsell 2007	26	436	18	440	13.0%	1.46 [0.81, 2.62]
Gunner-Svensson 1984	96	1844	107	1899	31.9%	0.92 [0.71, 1.21]
Sorensen 1988	99	585	124	777	34.4%	1.06 [0.83, 1.35]
**Subtotal (95% CI)**		**3344**		**3599**	**100.0%**	**1.02 [0.80, 1.30]**
Total events	251		286			
Heterogeneity: Tau² = 0.03; Chi² = 7.87, df = 5 (P = 0.16); I² = 36%						
Test for overall effect: Z = 0.14 (P = 0.89)						
**1.86.4 36+ months**						
Pathy 1992 (1)	8	201	6	195	6.4%	1.29 [0.46, 3.66]
Hall 1992	6	81	17	86	7.9%	0.37 [0.16, 0.90]
Byles 2004	30	942	7	627	8.6%	2.85 [1.26, 6.45]
Stuck 1995	9	215	20	199	9.2%	0.42 [0.19, 0.89]
Pathy 1992 (2)	12	139	22	143	10.5%	0.56 [0.29, 1.09]
van Rossum 1993	27	292	23	288	12.4%	1.16 [0.68, 1.97]
Thomas 2007	54	345	23	175	13.6%	1.19 [0.76, 1.87]
Stuck 2000	34	264	45	527	14.2%	1.51 [0.99, 2.30]
Gunner-Svensson 1984	154	1844	189	1899	17.3%	0.84 [0.69, 1.03]
**Subtotal (95% CI)**		**4323**		**4139**	**100.0%**	**0.96 [0.69, 1.33]**
Total events	334		352			
Heterogeneity: Tau² = 0.15; Chi² = 26.07, df = 8 (P = 0.001); I² = 69%						
Test for overall effect: Z = 0.25 (P = 0.80)						

Risk Ratio
M-H, Random, 95% CI

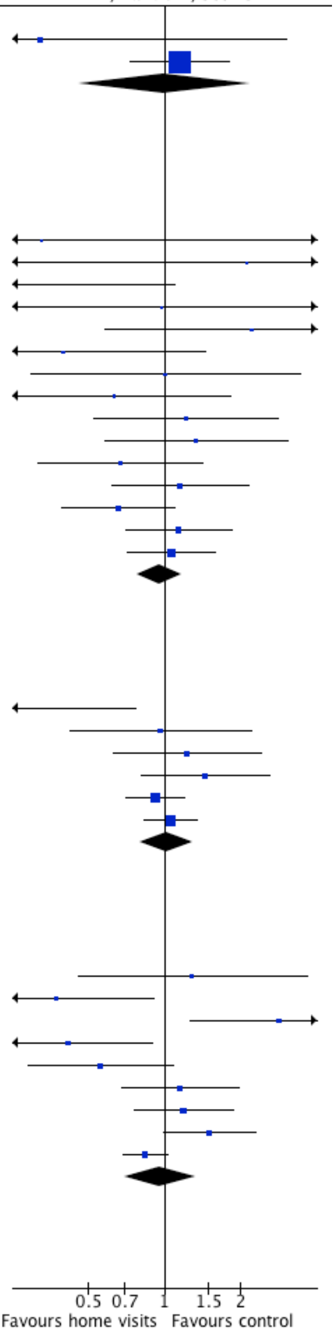

Analysis 67: Institutionalisation (people); focus of visit subgroups

Study or Subgroup	Home visits Events	Home visits Total	Control Events	Control Total	Weight	Risk Ratio M-H, Random, 95% CI
**1.87.1 Falls Only**						
Hogan 2001	2	79	1	84	0.4%	2.13 [0.20, 22.99]
**Subtotal (95% CI)**		**79**		**84**	**0.4%**	**2.13 [0.20, 22.99]**
Total events	2		1			
Heterogeneity: Not applicable						
Test for overall effect: Z = 0.62 (P = 0.53)						
**1.87.2 MGA Only**						
Dalby 2000	0	70	1	69	0.2%	0.33 [0.01, 7.93]
Shapiro 2002	1	40	11	65	0.5%	0.15 [0.02, 1.10]
Hebert 2001	5	250	5	253	1.3%	1.01 [0.30, 3.45]
Kono 2004	5	59	8	60	1.7%	0.64 [0.22, 1.83]
Pathy 1992 (1)	8	201	6	195	1.7%	1.29 [0.46, 3.66]
Hall 1992	6	81	17	86	2.3%	0.37 [0.16, 0.90]
Kono 2011	12	161	9	162	2.5%	1.34 [0.58, 3.10]
Bouman 2008	10	160	11	170	2.6%	0.97 [0.42, 2.21]
Byles 2004	30	942	7	627	2.6%	2.85 [1.26, 6.45]
Stuck 1995	9	215	20	199	2.9%	0.42 [0.19, 0.89]
Bernabei 1998	10	99	15	100	3.0%	0.67 [0.32, 1.43]
Sommers 2000	18	238	14	227	3.6%	1.23 [0.62, 2.41]
Pathy 1992 (2)	12	139	22	143	3.7%	0.56 [0.29, 1.09]
Counsell 2007	26	436	18	440	4.4%	1.46 [0.81, 2.62]
van Rossum 1993	27	292	23	288	5.0%	1.16 [0.68, 1.97]
Caplan 2004	32	370	28	369	5.6%	1.14 [0.70, 1.85]
Thomas 2007	54	345	23	175	6.2%	1.19 [0.76, 1.87]
Stuck 2000	34	264	45	527	6.7%	1.51 [0.99, 2.30]
Sorensen 1988	99	585	124	777	10.7%	1.06 [0.83, 1.35]
Gunner-Svensson 1984	154	1844	189	1899	11.7%	0.84 [0.69, 1.03]
**Subtotal (95% CI)**		**6791**		**6831**	**79.0%**	**0.99 [0.82, 1.20]**
Total events	552		596			
Heterogeneity: Tau² = 0.06; Chi² = 35.34, df = 19 (P = 0.01); I² = 46%						
Test for overall effect: Z = 0.07 (P = 0.94)						
**1.87.3 Both**						
Fabacher 1994	0	100	0	95		Not estimable
Newbury 2001	2	45	2	44	0.6%	0.98 [0.14, 6.64]
Yamada 2003	12	184	9	169	2.5%	1.22 [0.53, 2.83]
Close 1999	18	184	18	213	4.0%	1.16 [0.62, 2.16]
van Hout 2010	43	331	39	320	7.0%	1.07 [0.71, 1.60]
**Subtotal (95% CI)**		**844**		**841**	**14.1%**	**1.11 [0.81, 1.51]**
Total events	75		68			
Heterogeneity: Tau² = 0.00; Chi² = 0.13, df = 3 (P = 0.99); I² = 0%						
Test for overall effect: Z = 0.64 (P = 0.52)						
**1.87.4 Neither**						
Lenaghan 2007	1	68	3	66	0.4%	0.32 [0.03, 3.03]
Holland 2005	37	429	32	426	6.1%	1.15 [0.73, 1.81]
**Subtotal (95% CI)**		**497**		**492**	**6.6%**	**1.00 [0.46, 2.18]**
Total events	38		35			
Heterogeneity: Tau² = 0.13; Chi² = 1.19, df = 1 (P = 0.28); I² = 16%						
Test for overall effect: Z = 0.01 (P = 0.99)						
**Total (95% CI)**		**8211**		**8248**	**100.0%**	**1.02 [0.88, 1.18]**
Total events	667		700			
Heterogeneity: Tau² = 0.04; Chi² = 37.64, df = 26 (P = 0.07); I² = 31%						
Test for overall effect: Z = 0.30 (P = 0.77)						
Test for subgroup differences: Chi² = 0.71, df = 3 (P = 0.87), I² = 0%						

(1) Pathy 1992a
(2) Pathy 1992b

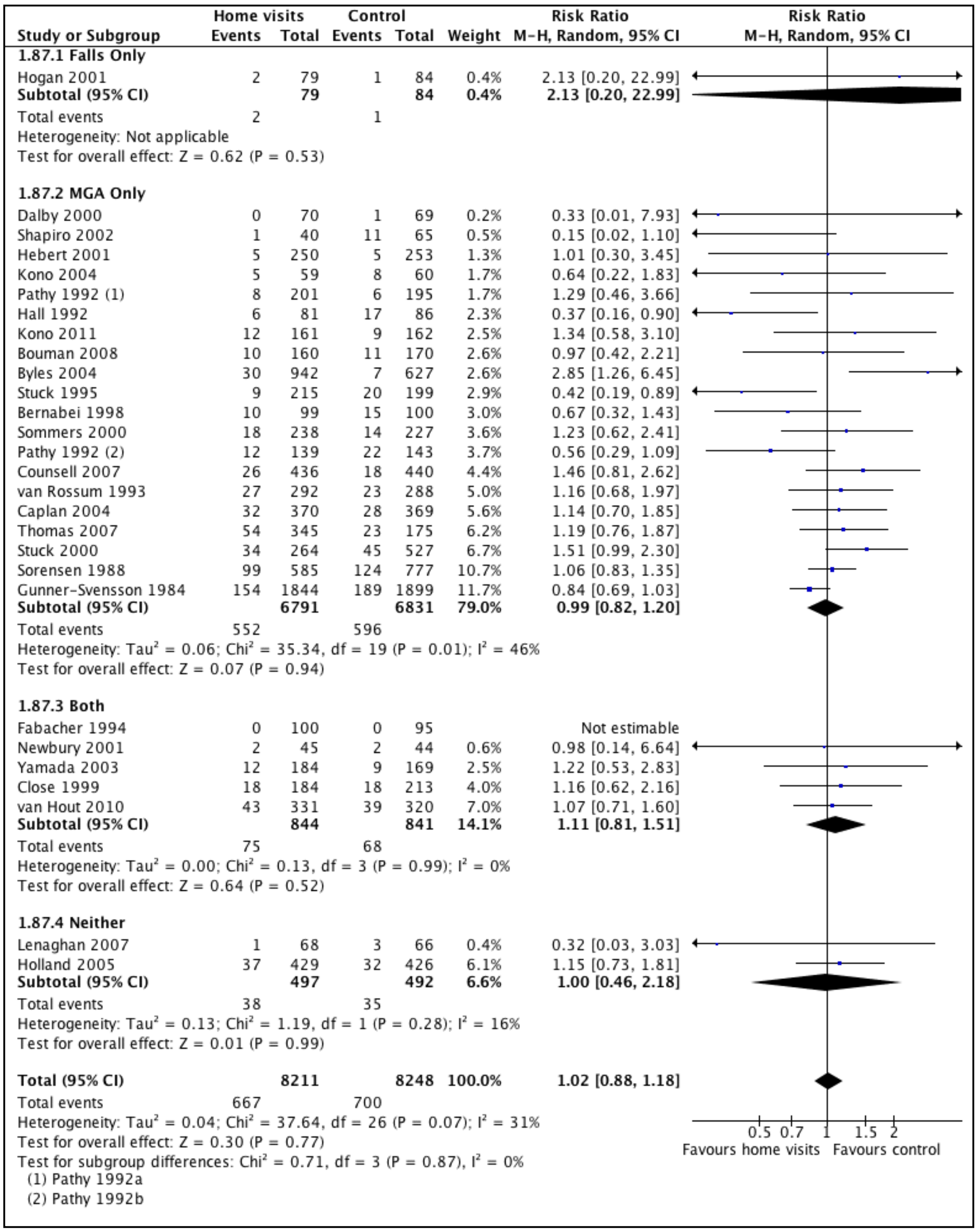

Analysis 68: Institutionalisation (people); age of participants subgroups

Study or Subgroup	Home visits Events	Home visits Total	Control Events	Control Total	Weight	Risk Ratio M-H, Random, 95% CI
**1.88.1 <=70**						
Pathy 1992 (1)	8	201	6	195	1.9%	1.29 [0.46, 3.66]
**Subtotal (95% CI)**		**201**		**195**	**1.9%**	**1.29 [0.46, 3.66]**
Total events	8		6			
Heterogeneity: Not applicable						
Test for overall effect: Z = 0.49 (P = 0.63)						
**1.88.2 71-75**						
Fabacher 1994	0	100	0	95		Not estimable
Counsell 2007	26	436	18	440	5.0%	1.46 [0.81, 2.62]
**Subtotal (95% CI)**		**536**		**535**	**5.0%**	**1.46 [0.81, 2.62]**
Total events	26		18			
Heterogeneity: Not applicable						
Test for overall effect: Z = 1.26 (P = 0.21)						
**1.88.3 76-80**						
Dalby 2000	0	70	1	69	0.2%	0.33 [0.01, 7.93]
Hogan 2001	2	79	1	84	0.4%	2.13 [0.20, 22.99]
Shapiro 2002	1	40	11	65	0.6%	0.15 [0.02, 1.10]
Newbury 2001	2	45	2	44	0.6%	0.98 [0.14, 6.64]
Hebert 2001	5	250	5	253	1.4%	1.01 [0.30, 3.45]
Hall 1992	6	81	17	86	2.6%	0.37 [0.16, 0.90]
Yamada 2003	12	184	9	169	2.8%	1.22 [0.53, 2.83]
Kono 2011	12	161	9	162	2.8%	1.34 [0.58, 3.10]
Bouman 2008	10	160	11	170	2.9%	0.97 [0.42, 2.21]
Byles 2004	30	942	7	627	3.0%	2.85 [1.26, 6.45]
Sommers 2000	18	238	14	227	4.0%	1.23 [0.62, 2.41]
Pathy 1992 (2)	12	139	22	143	4.1%	0.56 [0.29, 1.09]
Close 1999	18	184	18	213	4.5%	1.16 [0.62, 2.16]
van Rossum 1993	27	292	23	288	5.7%	1.16 [0.68, 1.97]
Sorensen 1988	99	585	124	777	12.3%	1.06 [0.83, 1.35]
**Subtotal (95% CI)**		**3450**		**3377**	**48.1%**	**1.03 [0.81, 1.31]**
Total events	254		274			
Heterogeneity: $Tau^2 = 0.06$; $Chi^2 = 19.90$, df = 14 (P = 0.13); $I^2 = 30\%$						
Test for overall effect: Z = 0.21 (P = 0.83)						
**1.88.4 81-85**						
Lenaghan 2007	1	68	3	66	0.5%	0.32 [0.03, 3.03]
Kono 2004	5	59	8	60	1.9%	0.64 [0.22, 1.83]
Stuck 1995	9	215	20	199	3.3%	0.42 [0.19, 0.89]
Bernabei 1998	10	99	15	100	3.4%	0.67 [0.32, 1.43]
Caplan 2004	32	370	28	369	6.4%	1.14 [0.70, 1.85]
Holland 2005	37	429	32	426	7.0%	1.15 [0.73, 1.81]
Thomas 2007	54	345	23	175	7.0%	1.19 [0.76, 1.87]
Stuck 2000	34	264	45	527	7.6%	1.51 [0.99, 2.30]
van Hout 2010	43	331	39	320	7.9%	1.07 [0.71, 1.60]
**Subtotal (95% CI)**		**2180**		**2242**	**45.0%**	**1.02 [0.80, 1.29]**
Total events	225		213			
Heterogeneity: $Tau^2 = 0.04$; $Chi^2 = 12.33$, df = 8 (P = 0.14); $I^2 = 35\%$						
Test for overall effect: Z = 0.12 (P = 0.90)						
**Total (95% CI)**		**6367**		**6349**	**100.0%**	**1.05 [0.90, 1.22]**
Total events	513		511			
Heterogeneity: $Tau^2 = 0.03$; $Chi^2 = 33.51$, df = 25 (P = 0.12); $I^2 = 25\%$						
Test for overall effect: Z = 0.62 (P = 0.53)						
Test for subgroup differences: $Chi^2 = 1.46$, df = 3 (P = 0.69), $I^2 = 0\%$						

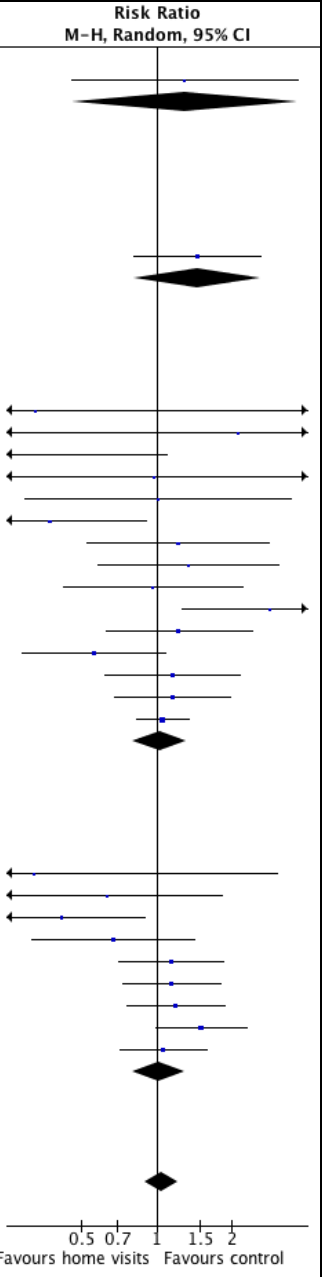

(1) Pathy 1992a
(2) Pathy 1992b

Analysis 69: Institutionalisation (people); type of visitor subgroups

Study or Subgroup	Home visits Events	Home visits Total	Control Events	Control Total	Weight	Risk Ratio M-H, Random, 95% CI
**1.89.1 Nurse**						
Dalby 2000	0	70	1	69	0.2%	0.33 [0.01, 7.93]
Shapiro 2002	1	40	11	65	0.5%	0.15 [0.02, 1.10]
Newbury 2001	2	45	2	44	0.6%	0.98 [0.14, 6.64]
Hebert 2001	5	250	5	253	1.3%	1.01 [0.30, 3.45]
Kono 2004	5	59	8	60	1.7%	0.64 [0.22, 1.83]
Hall 1992	6	81	17	86	2.3%	0.37 [0.16, 0.90]
Yamada 2003	12	184	9	169	2.5%	1.22 [0.53, 2.83]
Bouman 2008	10	160	11	170	2.6%	0.97 [0.42, 2.21]
Stuck 1995	9	215	20	199	2.9%	0.42 [0.19, 0.89]
van Rossum 1993	27	292	23	288	5.0%	1.16 [0.68, 1.97]
Caplan 2004	32	370	28	369	5.6%	1.14 [0.70, 1.85]
Thomas 2007	54	345	23	175	6.2%	1.19 [0.76, 1.87]
Stuck 2000	34	264	45	527	6.7%	1.51 [0.99, 2.30]
van Hout 2010	43	331	39	320	7.0%	1.07 [0.71, 1.60]
Gunner-Svensson 1984	154	1844	189	1899	11.7%	0.84 [0.69, 1.03]
**Subtotal (95% CI)**		**4550**		**4693**	**56.8%**	**0.95 [0.77, 1.16]**
Total events	394		431			
Heterogeneity: Tau² = 0.05; Chi² = 21.93, df = 14 (P = 0.08); I² = 36%						
Test for overall effect: Z = 0.51 (P = 0.61)						
**1.89.2 Other**						
Lenaghan 2007	1	68	3	66	0.4%	0.32 [0.03, 3.03]
Pathy 1992 (1)	8	201	6	195	1.7%	1.29 [0.46, 3.66]
Bernabei 1998	10	99	15	100	3.0%	0.67 [0.32, 1.43]
Pathy 1992 (2)	12	139	22	143	3.7%	0.56 [0.29, 1.09]
Close 1999	18	184	18	213	4.0%	1.16 [0.62, 2.16]
Holland 2005	37	429	32	426	6.1%	1.15 [0.73, 1.81]
**Subtotal (95% CI)**		**1120**		**1143**	**19.0%**	**0.92 [0.68, 1.24]**
Total events	86		96			
Heterogeneity: Tau² = 0.01; Chi² = 5.48, df = 5 (P = 0.36); I² = 9%						
Test for overall effect: Z = 0.56 (P = 0.58)						
**1.89.3 Combined**						
Fabacher 1994	0	100	0	95		Not estimable
Hogan 2001	2	79	1	84	0.4%	2.13 [0.20, 22.99]
Kono 2011	12	161	9	162	2.5%	1.34 [0.58, 3.10]
Byles 2004	30	942	7	627	2.6%	2.85 [1.26, 6.45]
Sommers 2000	18	238	14	227	3.6%	1.23 [0.62, 2.41]
Counsell 2007	26	436	18	440	4.4%	1.46 [0.81, 2.62]
Sorensen 1988	99	585	124	777	10.7%	1.06 [0.83, 1.35]
**Subtotal (95% CI)**		**2541**		**2412**	**24.2%**	**1.29 [0.99, 1.69]**
Total events	187		173			
Heterogeneity: Tau² = 0.02; Chi² = 6.13, df = 5 (P = 0.29); I² = 18%						
Test for overall effect: Z = 1.87 (P = 0.06)						
**Total (95% CI)**		**8211**		**8248**	**100.0%**	**1.02 [0.88, 1.18]**
Total events	667		700			
Heterogeneity: Tau² = 0.04; Chi² = 37.64, df = 26 (P = 0.07); I² = 31%						
Test for overall effect: Z = 0.30 (P = 0.77)						
Test for subgroup differences: Chi² = 3.94, df = 2 (P = 0.14), I² = 49.2%						

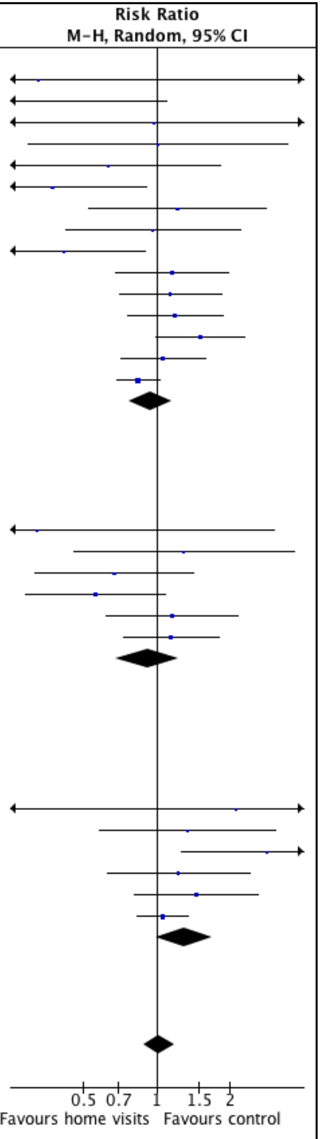

(1) Pathy 1992a
(2) Pathy 1992b

Analysis 70: Institutionalisation (people); number of visits subgroups

Study or Subgroup	Home visits Events	Home visits Total	Control Events	Control Total	Weight	Risk Ratio M-H, Random, 95% CI
**1.90.1 1 Visit**						
Newbury 2001	2	45	2	44	0.4%	0.98 [0.14, 6.64]
Hebert 2001	5	250	5	253	1.1%	1.01 [0.30, 3.45]
Close 1999	18	184	18	213	4.0%	1.16 [0.62, 2.16]
Sorensen 1988	99	585	124	777	21.3%	1.06 [0.83, 1.35]
**Subtotal (95% CI)**		**1064**		**1287**	**26.8%**	**1.07 [0.86, 1.33]**
Total events	124		149			
Heterogeneity: $Tau^2 = 0.00$; $Chi^2 = 0.08$, df = 3 (P = 0.99); $I^2 = 0\%$						
Test for overall effect: Z = 0.60 (P = 0.55)						
**1.90.2 2-4 Visits**						
Fabacher 1994	0	100	0	95		Not estimable
Dalby 2000	0	70	1	69	0.2%	0.33 [0.01, 7.93]
Hogan 2001	2	79	1	84	0.3%	2.13 [0.20, 22.99]
Lenaghan 2007	1	68	3	66	0.3%	0.32 [0.03, 3.03]
Kono 2004	5	59	8	60	1.4%	0.64 [0.22, 1.83]
Kono 2011	12	161	9	162	2.3%	1.34 [0.58, 3.10]
Caplan 2004	32	370	28	369	6.4%	1.14 [0.70, 1.85]
Holland 2005	37	429	32	426	7.2%	1.15 [0.73, 1.81]
Thomas 2007	54	345	23	175	7.3%	1.19 [0.76, 1.87]
van Hout 2010	43	331	39	320	8.9%	1.07 [0.71, 1.60]
**Subtotal (95% CI)**		**2012**		**1826**	**34.2%**	**1.10 [0.90, 1.36]**
Total events	186		144			
Heterogeneity: $Tau^2 = 0.00$; $Chi^2 = 3.44$, df = 8 (P = 0.90); $I^2 = 0\%$						
Test for overall effect: Z = 0.93 (P = 0.35)						
**1.90.3 5 or More Visits**						
Pathy 1992	8	201	6	195	1.5%	1.29 [0.46, 3.66]
Yamada 2003	12	184	9	169	2.2%	1.22 [0.53, 2.83]
Bouman 2008	10	160	11	170	2.3%	0.97 [0.42, 2.21]
Byles 2004	30	942	7	627	2.4%	2.85 [1.26, 6.45]
Stuck 1995	9	215	20	199	2.7%	0.42 [0.19, 0.89]
Bernabei 1998	10	99	15	100	2.8%	0.67 [0.32, 1.43]
Sommers 2000	18	238	14	227	3.4%	1.23 [0.62, 2.41]
Pathy 1992	12	139	22	143	3.5%	0.56 [0.29, 1.09]
Counsell 2007	26	436	18	440	4.5%	1.46 [0.81, 2.62]
van Rossum 1993	27	292	23	288	5.4%	1.16 [0.68, 1.97]
Stuck 2000	34	264	45	527	8.3%	1.51 [0.99, 2.30]
**Subtotal (95% CI)**		**3170**		**3085**	**39.0%**	**1.08 [0.80, 1.45]**
Total events	196		190			
Heterogeneity: $Tau^2 = 0.12$; $Chi^2 = 20.46$, df = 10 (P = 0.03); $I^2 = 51\%$						
Test for overall effect: Z = 0.52 (P = 0.60)						
**Total (95% CI)**		**6246**		**6198**	**100.0%**	**1.10 [0.97, 1.25]**
Total events	506		483			
Heterogeneity: $Tau^2 = 0.00$; $Chi^2 = 24.09$, df = 23 (P = 0.40); $I^2 = 5\%$						
Test for overall effect: Z = 1.47 (P = 0.14)						
Test for subgroup differences: $Chi^2 = 0.05$, df = 2 (P = 0.98), $I^2 = 0\%$						

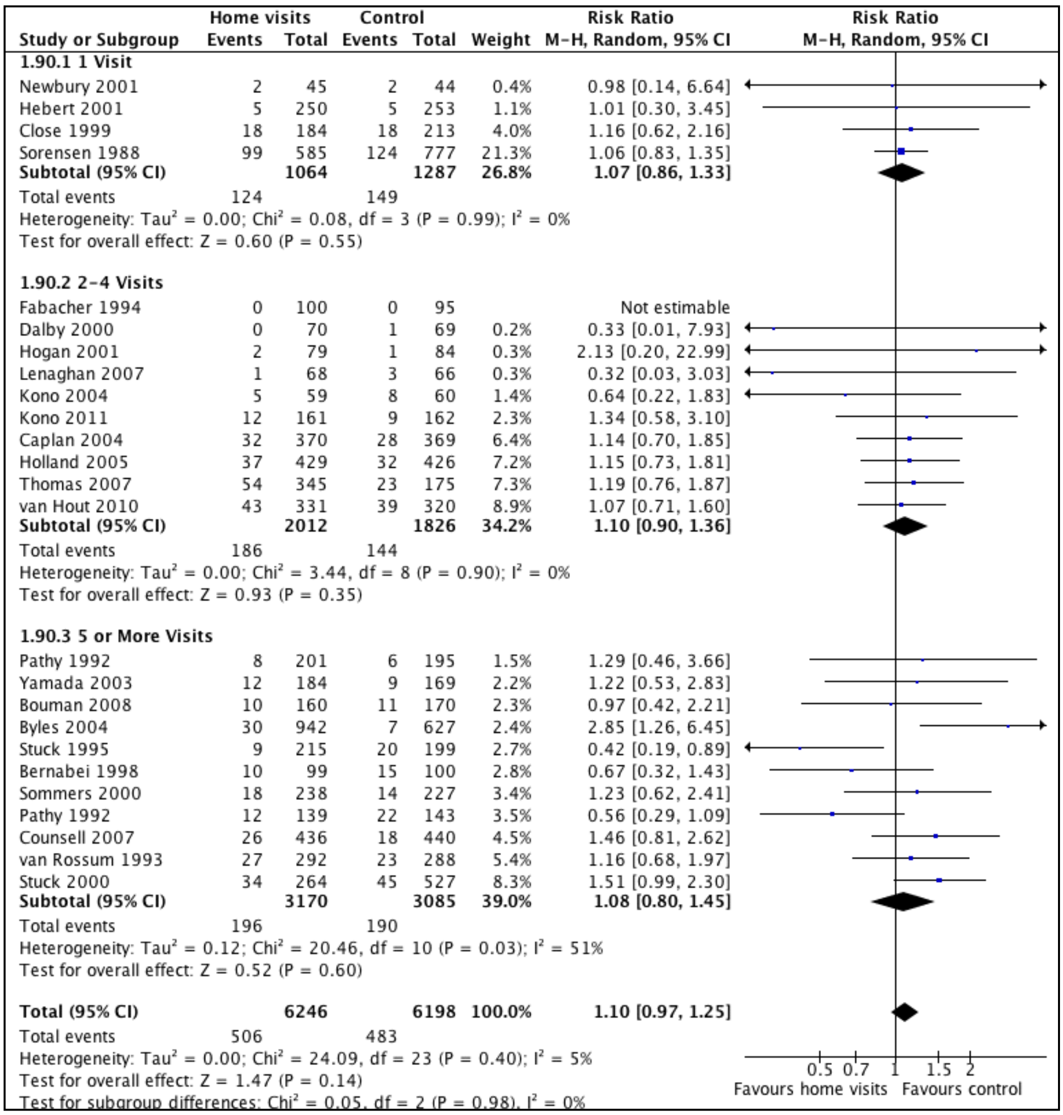

Analysis 71: Mortality (Focus of visits subgroups)

Study or Subgroup	Home visits Events	Home visits Total	Control Events	Control Total	Weight	Risk Ratio M-H, Random, 95% CI
**2.90.1 Falls Only**						
Wyman 2007	0	137	0	135		Not estimable
Gallagher 1996	0	50	0	50		Not estimable
Lin 2007	1	50	0	50	0.0%	3.00 [0.13, 71.92]
Gustafsson 2012	2	174	0	114	0.1%	3.29 [0.16, 67.82]
Liu-Ambrose 2008	1	36	2	38	0.1%	0.53 [0.05, 5.57]
Robertson 2001a	1	121	6	119	0.1%	0.16 [0.02, 1.34]
Hogan 2001	2	79	5	84	0.2%	0.43 [0.08, 2.13]
Green 2002	4	85	5	85	0.3%	0.80 [0.22, 2.88]
Elley 2008	7	155	4	157	0.3%	1.77 [0.53, 5.93]
Campbell 1999	4	116	7	117	0.3%	0.58 [0.17, 1.92]
Campbell 2005	4	98	7	96	0.3%	0.56 [0.17, 1.85]
Luukinen 2006	48	243	50	243	3.5%	0.96 [0.67, 1.37]
**Subtotal (95% CI)**		**1344**		**1288**	**5.2%**	**0.88 [0.66, 1.18]**
Total events	74		86			
Heterogeneity: $Tau^2 = 0.00$; $Chi^2 = 7.32$, df = 9 (P = 0.60); $I^2 = 0\%$						
Test for overall effect: Z = 0.84 (P = 0.40)						
**2.90.2 MGA Only**						
Shapiro 2002	1	40	4	65	0.1%	0.41 [0.05, 3.51]
Dalby 2000	7	70	3	69	0.3%	2.30 [0.62, 8.53]
Kono 2004	4	59	6	60	0.3%	0.68 [0.20, 2.28]
Ploeg 2010	10	361	10	358	0.6%	0.99 [0.42, 2.35]
Bernabei 1998	12	100	13	100	0.8%	0.92 [0.44, 1.92]
Hebert 2001	12	250	18	253	0.9%	0.67 [0.33, 1.37]
Kono 2011	11	161	20	162	0.9%	0.55 [0.27, 1.12]
Hall 1992	14	81	18	86	1.2%	0.83 [0.44, 1.55]
McEwan 1990	16	151	23	145	1.3%	0.67 [0.37, 1.21]
Sommers 2000	26	383	26	351	1.6%	0.92 [0.54, 1.55]
Stuck 1995	24	215	26	199	1.7%	0.85 [0.51, 1.44]
Bouman 2008	29	160	23	170	1.8%	1.34 [0.81, 2.22]
Balaban 1988	31	103	20	95	1.9%	1.43 [0.88, 2.33]
Pathy 1992	27	219	31	200	2.0%	0.80 [0.49, 1.28]
Counsell 2007	33	474	37	477	2.2%	0.90 [0.57, 1.41]
Vetter 1984b	35	296	60	296	3.0%	0.58 [0.40, 0.86]
Vetter 1984a	45	281	45	273	3.1%	0.97 [0.67, 1.42]
van Rossum 1993	42	292	50	288	3.1%	0.83 [0.57, 1.21]
Caplan 2004	55	370	53	369	3.6%	1.03 [0.73, 1.47]
Stuck 2000	47	264	67	527	3.7%	1.40 [0.99, 1.97]
Pathy 1992	40	150	55	156	3.8%	0.76 [0.54, 1.06]
Thomas 2007	89	345	49	175	4.8%	0.92 [0.68, 1.24]
Byles 2004	115	942	61	627	4.9%	1.25 [0.94, 1.68]
Gunner-Svensson 1984	449	1844	443	1899	21.7%	1.04 [0.93, 1.17]
**Subtotal (95% CI)**		**7611**		**7400**	**69.1%**	**0.95 [0.86, 1.05]**
Total events	1174		1161			
Heterogeneity: $Tau^2 = 0.01$; $Chi^2 = 31.42$, df = 23 (P = 0.11); $I^2 = 27\%$						
Test for overall effect: Z = 0.95 (P = 0.34)						
**2.90.3 Both**						
Hendriks 2008	5	166	1	167	0.1%	5.03 [0.59, 42.59]
Newbury 2001	1	50	5	50	0.1%	0.20 [0.02, 1.65]
Markle-Reid 2010	3	54	4	55	0.2%	0.76 [0.18, 3.25]
Davison 2005	3	159	5	154	0.2%	0.58 [0.14, 2.39]
Fabacher 1994	4	131	4	123	0.2%	0.94 [0.24, 3.67]
Ciaschini 2009	6	101	4	100	0.3%	1.49 [0.43, 5.10]
Tinetti 1994	7	153	5	148	0.4%	1.35 [0.44, 4.17]
Lightbody 2002	11	171	7	177	0.5%	1.63 [0.65, 4.10]
van Haastregt 2000	10	159	14	157	0.8%	0.71 [0.32, 1.54]
Yamada 2003	11	184	15	184	0.8%	0.73 [0.35, 1.55]
Close 1999	19	184	27	213	1.5%	0.81 [0.47, 1.42]
van Hout 2010	27	331	31	320	1.9%	0.84 [0.51, 1.38]
Gitlin 2006	34	160	42	159	2.8%	0.80 [0.54, 1.19]
Sahlen 2006	50	248	82	346	4.4%	0.85 [0.62, 1.16]
Vetter 1992	88	350	106	324	7.1%	0.77 [0.61, 0.98]
**Subtotal (95% CI)**		**2601**		**2677**	**21.4%**	**0.82 [0.72, 0.95]**
Total events	279		352			
Heterogeneity: $Tau^2 = 0.00$; $Chi^2 = 9.12$, df = 14 (P = 0.82); $I^2 = 0\%$						
Test for overall effect: Z = 2.66 (P = 0.008)						
**2.90.4 Neither**						
Nelson 2004	1	34	0	38	0.0%	3.34 [0.14, 79.42]
Crawford Shearer 2010	2	27	2	32	0.1%	1.19 [0.18, 7.86]
Ciechanowski 2004	3	72	2	66	0.2%	1.38 [0.24, 7.97]
Lenaghan 2007	7	69	6	67	0.4%	1.13 [0.40, 3.20]
Holland 2005	49	437	63	435	3.6%	0.77 [0.55, 1.10]
**Subtotal (95% CI)**		**639**		**638**	**4.3%**	**0.84 [0.61, 1.16]**
Total events	62		73			
Heterogeneity: $Tau^2 = 0.00$; $Chi^2 = 1.69$, df = 4 (P = 0.79); $I^2 = 0\%$						
Test for overall effect: Z = 1.07 (P = 0.28)						
**Total (95% CI)**		**12195**		**12003**	**100.0%**	**0.93 [0.87, 0.99]**
Total events	1589		1672			
Heterogeneity: $Tau^2 = 0.00$; $Chi^2 = 54.89$, df = 53 (P = 0.40); $I^2 = 3\%$						
Test for overall effect: Z = 2.11 (P = 0.03)						
Test for subgroup differences: $Chi^2 = 2.72$, df = 3 (P = 0.44), $I^2 = 0\%$						

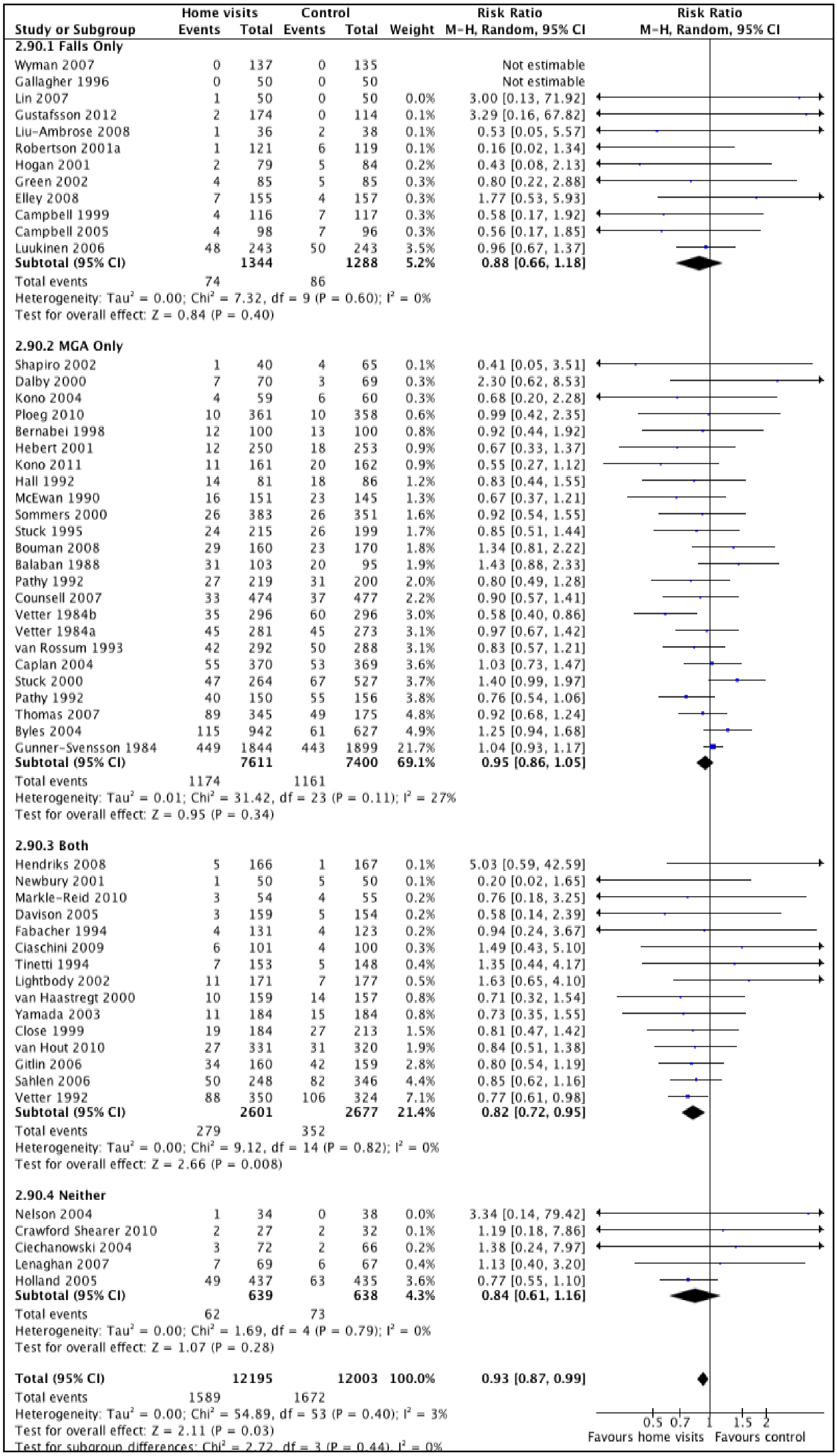

Analysis 72: Mortality; age of participants subgroups

Study or Subgroup	Home visits Events	Home visits Total	Control Events	Control Total	Weight	Risk Ratio M-H, Random, 95% CI
**2.91.1 <=70**						
Balaban 1988	31	103	20	95	2.4%	1.43 [0.88, 2.33]
**Subtotal (95% CI)**		**103**		**95**	**2.4%**	**1.43 [0.88, 2.33]**
Total events	31		20			
Heterogeneity: Not applicable						
Test for overall effect: Z = 1.44 (P = 0.15)						
**2.91.2 71-75**						
Gallagher 1996	0	50	0	50		Not estimable
Hendriks 2008	5	166	1	167	0.1%	5.03 [0.59, 42.59]
Ciechanowski 2004	3	72	2	66	0.2%	1.38 [0.24, 7.97]
Fabacher 1994	4	131	4	123	0.3%	0.94 [0.24, 3.67]
Green 2002	4	85	5	85	0.3%	0.80 [0.22, 2.88]
Ciaschini 2009	6	101	4	100	0.4%	1.49 [0.43, 5.10]
Lightbody 2002	11	171	7	177	0.7%	1.63 [0.65, 4.10]
Pathy 1992	27	219	31	200	2.5%	0.80 [0.49, 1.28]
Counsell 2007	33	474	37	477	2.8%	0.90 [0.57, 1.41]
**Subtotal (95% CI)**		**1469**		**1445**	**7.3%**	**0.97 [0.73, 1.28]**
Total events	93		91			
Heterogeneity: Tau² = 0.00; Chi² = 4.99, df = 7 (P = 0.66); I² = 0%						
Test for overall effect: Z = 0.23 (P = 0.82)						
**2.91.3 76-80**						
Wyman 2007	0	137	0	135		Not estimable
Lin 2007	1	50	0	50	0.1%	3.00 [0.13, 71.92]
Nelson 2004	1	34	0	38	0.1%	3.34 [0.14, 79.42]
Shapiro 2002	1	40	4	65	0.1%	0.41 [0.05, 3.51]
Newbury 2001	1	50	5	50	0.1%	0.20 [0.02, 1.65]
Crawford Shearer 2010	2	27	2	32	0.2%	1.19 [0.18, 7.86]
Hogan 2001	2	79	5	84	0.2%	0.43 [0.08, 2.13]
Davison 2005	3	159	5	154	0.3%	0.58 [0.14, 2.39]
Dalby 2000	7	70	3	69	0.3%	2.30 [0.62, 8.53]
Tinetti 1994	7	153	5	148	0.4%	1.35 [0.44, 4.17]
van Haastregt 2000	10	159	14	157	0.9%	0.71 [0.32, 1.54]
Yamada 2003	11	184	15	184	1.0%	0.73 [0.35, 1.55]
Hebert 2001	12	250	18	253	1.1%	0.67 [0.33, 1.37]
Kono 2011	11	161	20	162	1.2%	0.55 [0.27, 1.12]
Hall 1992	14	81	18	86	1.4%	0.83 [0.44, 1.55]
Close 1999	19	184	27	213	1.9%	0.81 [0.47, 1.42]
Sommers 2000	26	383	26	351	2.1%	0.92 [0.54, 1.55]
Bouman 2008	29	160	23	170	2.3%	1.34 [0.81, 2.22]
Gitlin 2006	34	160	42	159	3.6%	0.80 [0.54, 1.19]
Vetter 1984b	35	296	60	296	3.8%	0.58 [0.40, 0.86]
Vetter 1984a	45	281	45	273	4.0%	0.97 [0.67, 1.42]
van Rossum 1993	42	292	50	288	4.0%	0.83 [0.57, 1.21]
Pathy 1992	40	150	55	156	4.9%	0.76 [0.54, 1.06]
Sahlen 2006	50	248	82	346	5.9%	0.85 [0.62, 1.16]
Byles 2004	115	942	61	627	6.6%	1.25 [0.94, 1.68]
Vetter 1992	88	350	106	324	10.0%	0.77 [0.61, 0.98]
**Subtotal (95% CI)**		**5080**		**4870**	**56.5%**	**0.85 [0.76, 0.94]**
Total events	606		691			
Heterogeneity: Tau² = 0.00; Chi² = 25.18, df = 24 (P = 0.40); I² = 5%						
Test for overall effect: Z = 3.03 (P = 0.002)						
**2.91.4 81-85**						
Liu-Ambrose 2008	1	36	2	38	0.1%	0.53 [0.05, 5.57]
Robertson 2001a	1	121	6	119	0.1%	0.16 [0.02, 1.34]
Kono 2004	4	59	6	60	0.4%	0.68 [0.20, 2.28]
Elley 2008	7	155	4	157	0.4%	1.77 [0.53, 5.93]
Campbell 1999	4	116	7	117	0.4%	0.58 [0.17, 1.92]
Campbell 2005	4	98	7	96	0.4%	0.56 [0.17, 1.85]
Lenaghan 2007	7	69	6	67	0.5%	1.13 [0.40, 3.20]
Ploeg 2010	10	361	10	358	0.8%	0.99 [0.42, 2.35]
Bernabei 1998	12	100	13	100	1.1%	0.92 [0.44, 1.92]
Stuck 1995	24	215	26	199	2.1%	0.85 [0.51, 1.44]
van Hout 2010	27	331	31	320	2.3%	0.84 [0.51, 1.38]
Holland 2005	49	437	63	435	4.7%	0.77 [0.55, 1.10]
Caplan 2004	55	370	53	369	4.7%	1.03 [0.73, 1.47]
Stuck 2000	47	264	67	527	4.9%	1.40 [0.99, 1.97]
Thomas 2007	89	345	49	175	6.4%	0.92 [0.68, 1.24]
**Subtotal (95% CI)**		**3077**		**3137**	**29.2%**	**0.95 [0.83, 1.10]**
Total events	341		350			
Heterogeneity: Tau² = 0.00; Chi² = 12.73, df = 14 (P = 0.55); I² = 0%						
Test for overall effect: Z = 0.65 (P = 0.51)						
**2.91.5 86+**						
Gustafsson 2012	2	174	0	114	0.1%	3.29 [0.16, 67.82]
Luukinen 2006	48	243	50	243	4.5%	0.96 [0.67, 1.37]
**Subtotal (95% CI)**		**417**		**357**	**4.6%**	**0.98 [0.69, 1.39]**
Total events	50		50			
Heterogeneity: Tau² = 0.00; Chi² = 0.63, df = 1 (P = 0.43); I² = 0%						
Test for overall effect: Z = 0.14 (P = 0.89)						
**Total (95% CI)**		**10146**		**9904**	**100.0%**	**0.90 [0.84, 0.98]**
Total events	1121		1202			
Heterogeneity: Tau² = 0.00; Chi² = 49.32, df = 50 (P = 0.50); I² = 0%						
Test for overall effect: Z = 2.62 (P = 0.009)						
Test for subgroup differences: Chi² = 5.73, df = 4 (P = 0.22), I² = 30.2%						

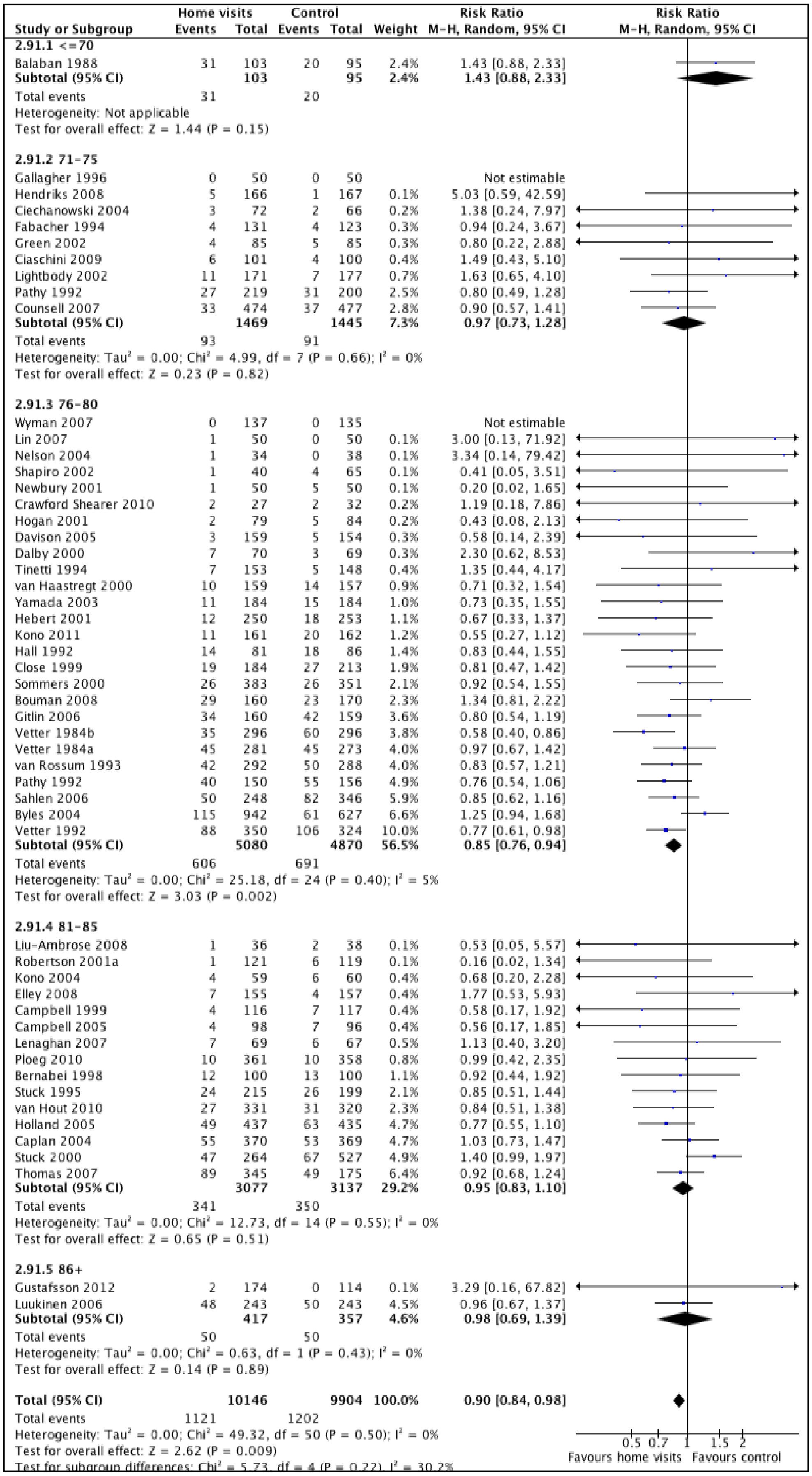

Analysis 73: Mortality; type of visitor subgroups

Study or Subgroup	Home visits Events	Home visits Total	Control Events	Control Total	Weight	Risk Ratio M-H, Random, 95% CI
**2.92.1 Nurse**						
Gallagher 1996	0	50	0	50		Not estimable
Wyman 2007	0	137	0	135		Not estimable
Shapiro 2002	1	40	4	65	0.1%	0.41 [0.05, 3.51]
Newbury 2001	1	50	5	50	0.1%	0.20 [0.02, 1.65]
Robertson 2001a	1	121	6	119	0.1%	0.16 [0.02, 1.34]
Crawford Shearer 2010	2	27	2	32	0.1%	1.19 [0.18, 7.86]
Dalby 2000	7	70	3	69	0.3%	2.30 [0.62, 8.53]
Ciaschini 2009	6	101	4	100	0.3%	1.49 [0.43, 5.10]
Kono 2004	4	59	6	60	0.3%	0.68 [0.20, 2.28]
Elley 2008	7	155	4	157	0.3%	1.77 [0.53, 5.93]
Lightbody 2002	11	171	7	177	0.5%	1.63 [0.65, 4.10]
Ploeg 2010	10	361	10	358	0.6%	0.99 [0.42, 2.35]
van Haastregt 2000	10	159	14	157	0.8%	0.71 [0.32, 1.54]
Yamada 2003	11	184	15	184	0.8%	0.73 [0.35, 1.55]
Hebert 2001	12	250	18	253	0.9%	0.67 [0.33, 1.37]
Hall 1992	14	81	18	86	1.2%	0.83 [0.44, 1.55]
McEwan 1990	16	151	23	145	1.3%	0.67 [0.37, 1.21]
Stuck 1995	24	215	26	199	1.7%	0.85 [0.51, 1.44]
Bouman 2008	29	160	23	170	1.8%	1.34 [0.81, 2.22]
van Hout 2010	27	331	31	320	1.9%	0.84 [0.51, 1.38]
van Rossum 1993	42	292	50	288	3.1%	0.83 [0.57, 1.21]
Caplan 2004	55	370	53	369	3.6%	1.03 [0.73, 1.47]
Stuck 2000	47	264	67	527	3.7%	1.40 [0.99, 1.97]
Thomas 2007	89	345	49	175	4.8%	0.92 [0.68, 1.24]
Gunner-Svensson 1984	449	1844	443	1899	21.7%	1.04 [0.93, 1.17]
**Subtotal (95% CI)**		**5988**		**6144**	**49.9%**	**1.00 [0.91, 1.10]**
Total events	875		881			
Heterogeneity: Tau² = 0.00; Chi² = 22.43, df = 22 (P = 0.43); I² = 2%						
Test for overall effect: Z = 0.03 (P = 0.97)						
**2.92.2 Other**						
Lin 2007	1	50	0	50	0.0%	3.00 [0.13, 71.92]
Nelson 2004	1	34	0	38	0.0%	3.34 [0.14, 79.42]
Liu-Ambrose 2008	1	36	2	38	0.1%	0.53 [0.05, 5.57]
Ciechanowski 2004	3	72	2	66	0.2%	1.38 [0.24, 7.97]
Green 2002	4	85	5	85	0.3%	0.80 [0.22, 2.88]
Campbell 1999	4	116	7	117	0.3%	0.58 [0.17, 1.92]
Lenaghan 2007	7	69	6	67	0.4%	1.13 [0.40, 3.20]
Bernabei 1998	12	100	13	100	0.8%	0.92 [0.44, 1.92]
Close 1999	19	184	27	213	1.5%	0.81 [0.47, 1.42]
Pathy 1992	27	219	31	200	2.0%	0.80 [0.49, 1.28]
Vetter 1984b	35	296	60	296	3.0%	0.58 [0.40, 0.86]
Vetter 1984a	45	281	45	273	3.1%	0.97 [0.67, 1.42]
Holland 2005	49	437	63	435	3.6%	0.77 [0.55, 1.10]
Pathy 1992	40	150	55	156	3.8%	0.76 [0.54, 1.06]
Vetter 1992	88	350	106	324	7.1%	0.77 [0.61, 0.98]
**Subtotal (95% CI)**		**2479**		**2458**	**26.1%**	**0.78 [0.69, 0.89]**
Total events	336		422			
Heterogeneity: Tau² = 0.00; Chi² = 6.52, df = 14 (P = 0.95); I² = 0%						
Test for overall effect: Z = 3.77 (P = 0.0002)						
**2.92.3 Combined**						
Gustafsson 2012	2	174	0	114	0.1%	3.29 [0.16, 67.82]
Hendriks 2008	5	166	1	167	0.1%	5.03 [0.59, 42.59]
Hogan 2001	2	79	5	84	0.2%	0.43 [0.08, 2.13]
Markle-Reid 2010	3	54	4	55	0.2%	0.76 [0.18, 3.25]
Davison 2005	3	159	5	154	0.2%	0.58 [0.14, 2.39]
Fabacher 1994	4	131	4	123	0.2%	0.94 [0.24, 3.67]
Campbell 2005	4	98	7	96	0.3%	0.56 [0.17, 1.85]
Tinetti 1994	7	153	5	148	0.4%	1.35 [0.44, 4.17]
Kono 2011	11	161	20	162	0.9%	0.55 [0.27, 1.12]
Sommers 2000	26	383	26	351	1.6%	0.92 [0.54, 1.55]
Balaban 1988	31	103	20	95	1.9%	1.43 [0.88, 2.33]
Counsell 2007	33	474	37	477	2.2%	0.90 [0.57, 1.41]
Gitlin 2006	34	160	42	159	2.8%	0.80 [0.54, 1.19]
Luukinen 2006	48	243	50	243	3.5%	0.96 [0.67, 1.37]
Sahlen 2006	50	248	82	346	4.4%	0.85 [0.62, 1.16]
Byles 2004	115	942	61	627	4.9%	1.25 [0.94, 1.68]
**Subtotal (95% CI)**		**3728**		**3401**	**24.0%**	**0.97 [0.84, 1.11]**
Total events	378		369			
Heterogeneity: Tau² = 0.00; Chi² = 15.21, df = 15 (P = 0.44); I² = 1%						
Test for overall effect: Z = 0.47 (P = 0.64)						
**Total (95% CI)**		**12195**		**12003**	**100.0%**	**0.93 [0.87, 0.99]**
Total events	1589		1672			
Heterogeneity: Tau² = 0.00; Chi² = 54.89, df = 53 (P = 0.40); I² = 3%						
Test for overall effect: Z = 2.11 (P = 0.03)						
Test for subgroup differences: Chi² = 9.94, df = 2 (P = 0.007), I² = 79.9%						

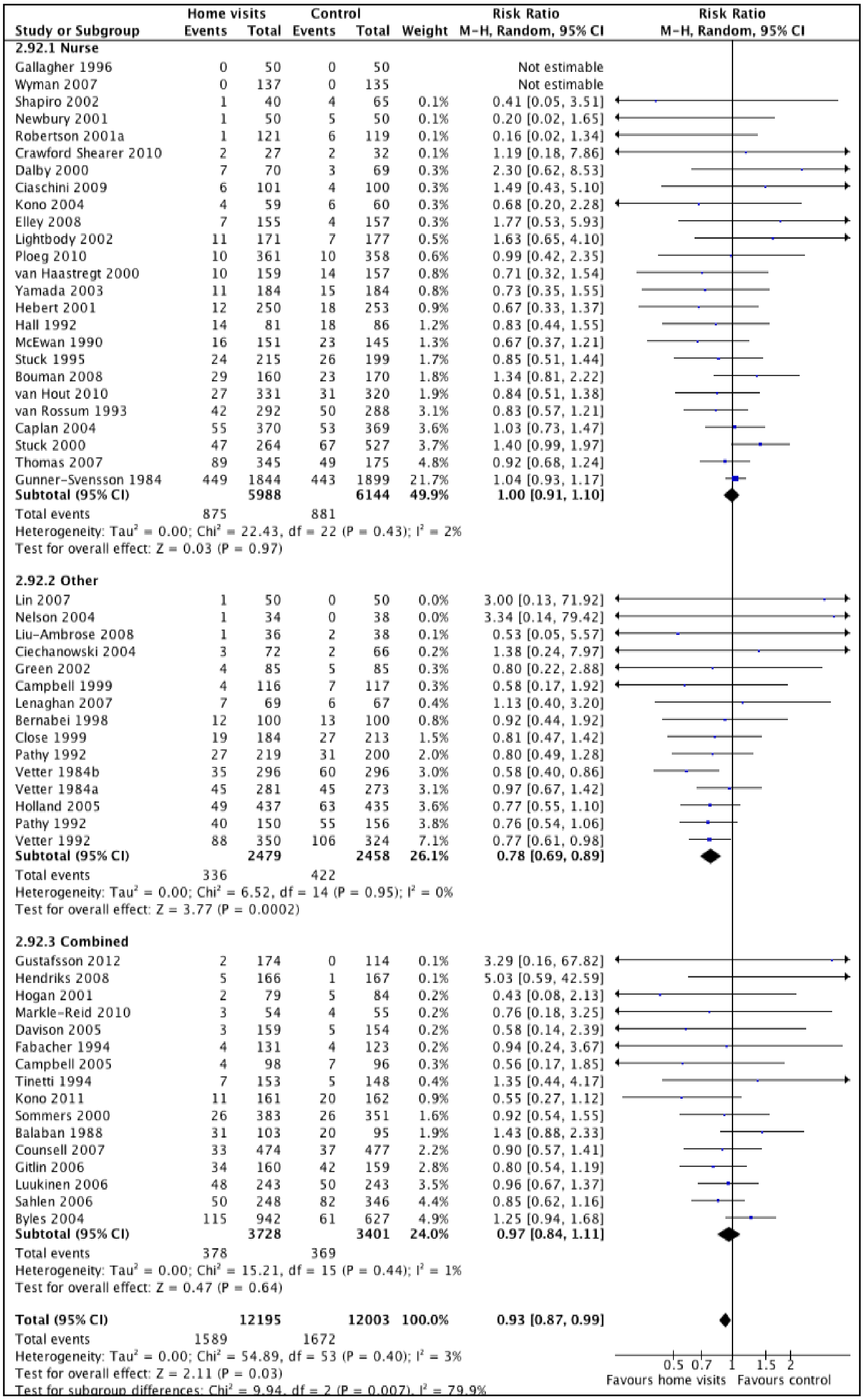

Analysis 74: Mortality; number of visits subgroups

Study or Subgroup	Home visits Events	Home visits Total	Control Events	Control Total	Weight	Risk Ratio M-H, Random, 95% CI
**2.93.1 1 Visit**						
Gustafsson 2012	2	174	0	114	0.1%	3.29 [0.16, 67.82]
Hendriks 2008	5	166	1	167	0.1%	5.03 [0.59, 42.59]
Newbury 2001	1	50	5	50	0.1%	0.20 [0.02, 1.65]
Lightbody 2002	11	171	7	177	0.7%	1.63 [0.65, 4.10]
Hebert 2001	12	250	18	253	1.2%	0.67 [0.33, 1.37]
McEwan 1990	16	151	23	145	1.7%	0.67 [0.37, 1.21]
Close 1999	19	184	27	213	1.9%	0.81 [0.47, 1.42]
**Subtotal (95% CI)**		**1146**		**1119**	**5.8%**	**0.86 [0.57, 1.29]**
Total events	66		81			
Heterogeneity: Tau² = 0.08; Chi² = 8.17, df = 6 (P = 0.23); I² = 27%						
Test for overall effect: Z = 0.74 (P = 0.46)						
**2.93.2 2-4 Visits**						
Gallagher 1996	0	50	0	50		Not estimable
Hogan 2001	2	79	5	84	0.2%	0.43 [0.08, 2.13]
Fabacher 1994	4	131	4	123	0.3%	0.94 [0.24, 3.67]
Dalby 2000	7	70	3	69	0.3%	2.30 [0.62, 8.53]
Green 2002	4	85	5	85	0.4%	0.80 [0.22, 2.88]
Kono 2004	4	59	6	60	0.4%	0.68 [0.20, 2.28]
Campbell 1999	4	116	7	117	0.4%	0.58 [0.17, 1.92]
Lenaghan 2007	7	69	6	67	0.5%	1.13 [0.40, 3.20]
Ploeg 2010	10	361	10	358	0.8%	0.99 [0.42, 2.35]
Kono 2011	11	161	20	162	1.2%	0.55 [0.27, 1.12]
van Hout 2010	27	331	31	320	2.4%	0.84 [0.51, 1.38]
Vetter 1984b	35	296	60	296	4.0%	0.58 [0.40, 0.86]
Vetter 1984a	45	281	45	273	4.1%	0.97 [0.67, 1.42]
Luukinen 2006	48	243	50	243	4.7%	0.96 [0.67, 1.37]
Holland 2005	49	437	63	435	4.8%	0.77 [0.55, 1.10]
Caplan 2004	55	370	53	369	4.8%	1.03 [0.73, 1.47]
Sahlen 2006	50	248	82	346	6.1%	0.85 [0.62, 1.16]
Thomas 2007	89	345	49	175	6.6%	0.92 [0.68, 1.24]
Vetter 1992	88	350	106	324	10.3%	0.77 [0.61, 0.98]
**Subtotal (95% CI)**		**4082**		**3956**	**52.4%**	**0.84 [0.75, 0.93]**
Total events	539		605			
Heterogeneity: Tau² = 0.00; Chi² = 12.37, df = 17 (P = 0.78); I² = 0%						
Test for overall effect: Z = 3.25 (P = 0.001)						
**2.93.3 5 or More Visits**						
Wyman 2007	0	137	0	135		Not estimable
Lin 2007	1	50	0	50	0.1%	3.00 [0.13, 71.92]
Nelson 2004	1	34	0	38	0.1%	3.34 [0.14, 79.42]
Liu-Ambrose 2008	1	36	2	38	0.1%	0.53 [0.05, 5.57]
Robertson 2001a	1	121	6	119	0.1%	0.16 [0.02, 1.34]
Crawford Shearer 2010	2	27	2	32	0.2%	1.19 [0.18, 7.86]
Ciechanowski 2004	3	72	2	66	0.2%	1.38 [0.24, 7.97]
Markle-Reid 2010	3	54	4	55	0.3%	0.76 [0.18, 3.25]
Campbell 2005	4	98	7	96	0.4%	0.56 [0.17, 1.85]
Tinetti 1994	7	153	5	148	0.5%	1.35 [0.44, 4.17]
van Haastregt 2000	10	159	14	157	1.0%	0.71 [0.32, 1.54]
Yamada 2003	11	184	15	184	1.0%	0.73 [0.35, 1.55]
Bernabei 1998	12	100	13	100	1.1%	0.92 [0.44, 1.92]
Sommers 2000	26	383	26	351	2.1%	0.92 [0.54, 1.55]
Stuck 1995	24	215	26	199	2.2%	0.85 [0.51, 1.44]
Bouman 2008	29	160	23	170	2.3%	1.34 [0.81, 2.22]
Pathy 1992	27	219	31	200	2.6%	0.80 [0.49, 1.28]
Counsell 2007	33	474	37	477	2.9%	0.90 [0.57, 1.41]
Gitlin 2006	34	160	42	159	3.8%	0.80 [0.54, 1.19]
van Rossum 1993	42	292	50	288	4.1%	0.83 [0.57, 1.21]
Stuck 2000	47	264	67	527	5.0%	1.40 [0.99, 1.97]
Pathy 1992	40	150	55	156	5.1%	0.76 [0.54, 1.06]
Byles 2004	115	942	61	627	6.8%	1.25 [0.94, 1.68]
**Subtotal (95% CI)**		**4484**		**4372**	**41.8%**	**0.96 [0.86, 1.09]**
Total events	473		488			
Heterogeneity: Tau² = 0.00; Chi² = 20.30, df = 21 (P = 0.50); I² = 0%						
Test for overall effect: Z = 0.61 (P = 0.54)						
**Total (95% CI)**		**9712**		**9447**	**100.0%**	**0.89 [0.82, 0.96]**
Total events	1078		1174			
Heterogeneity: Tau² = 0.00; Chi² = 43.98, df = 46 (P = 0.56); I² = 0%						
Test for overall effect: Z = 3.03 (P = 0.002)						
Test for subgroup differences: Chi² = 2.98, df = 2 (P = 0.23), I² = 32.8%						

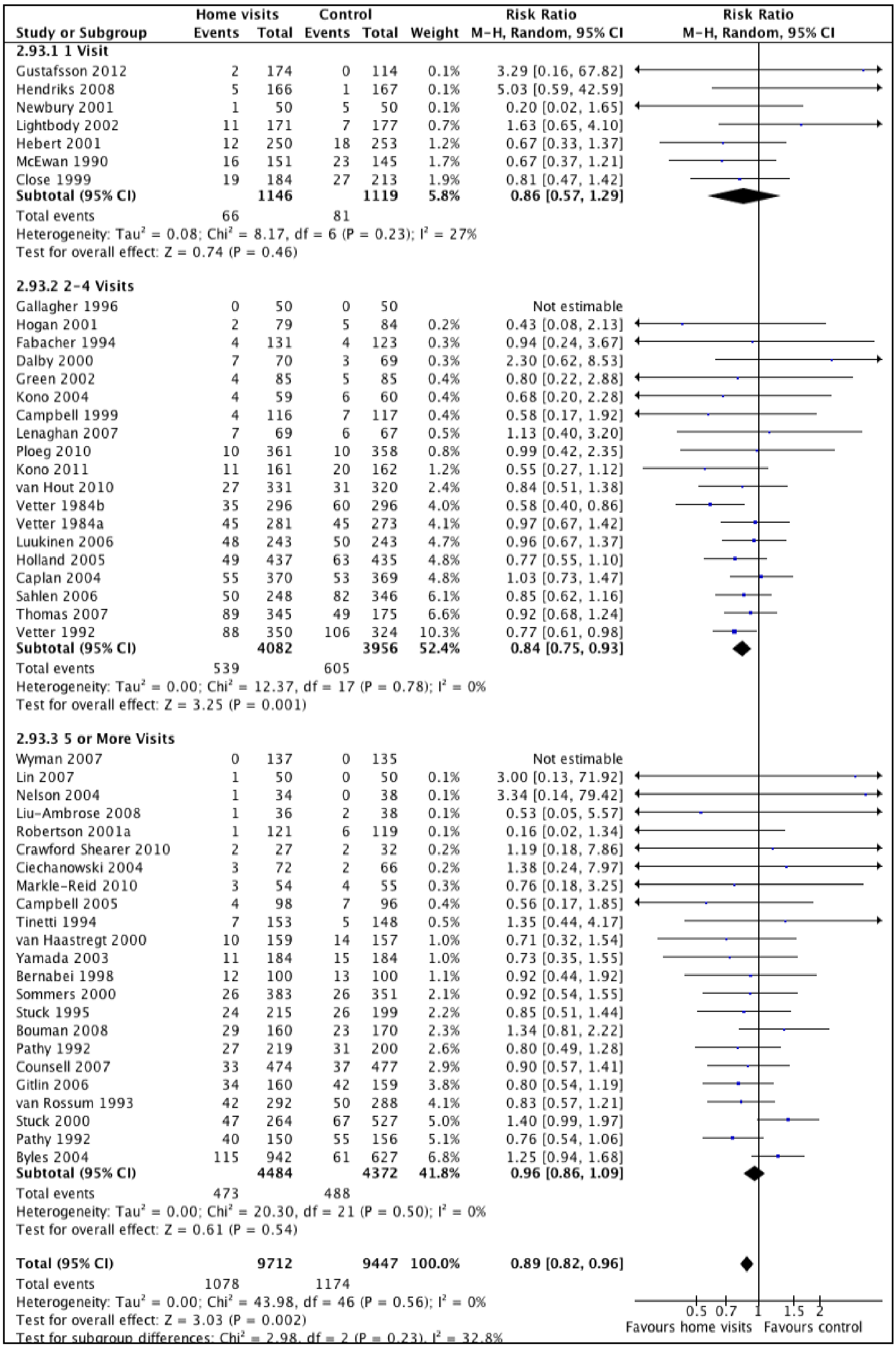

Analysis 75: Psychiatric (anxiety and depression), results at longest follow-up

Study or Subgroup	Std. Mean Difference	SE	Home visits Total	Control Total	Weight	Std. Mean Difference IV, Random, 95% CI
Shapiro 2002	−0.38438454	0.27919357	32	21	2.1%	−0.38 [−0.93, 0.16]
Kono 2004	−0.1249206	0.26073557	32	26	2.3%	−0.12 [−0.64, 0.39]
Lin 2007	−0.21979435	0.22352157	39	40	3.1%	−0.22 [−0.66, 0.22]
Balaban 1988	−0.04008557	0.21429989	40	46	3.3%	−0.04 [−0.46, 0.38]
Markle-Reid 2010	0.14926786	0.20750508	49	43	3.5%	0.15 [−0.26, 0.56]
Ciechanowski 2004	−0.34318576	0.17798035	67	60	4.6%	−0.34 [−0.69, 0.01]
Bernabei 1998	−0.40447154	0.15207651	88	87	6.0%	−0.40 [−0.70, −0.11]
Luukinen 2006	−0.10410841	0.1411547	144	150	6.7%	−0.10 [−0.38, 0.17]
Markle-Reid 2006	0.01619677	0.13202045	113	115	7.5%	0.02 [−0.24, 0.27]
Hendriks 2008	0.05020461	0.12426243	124	134	8.2%	0.05 [−0.19, 0.29]
Bouman 2008	−0.08667185	0.11676946	139	154	9.0%	−0.09 [−0.32, 0.14]
Kono 2011	−0.02557471	0.11102786	161	162	9.6%	−0.03 [−0.24, 0.19]
Sommers 2000	−0.06566503	0.10200118	201	183	10.8%	−0.07 [−0.27, 0.13]
Vetter 1984a	−0.28999705	0.09935649	206	202	11.2%	−0.29 [−0.48, −0.10]
Vetter 1984b	0.05857493	0.09322435	241	219	12.1%	0.06 [−0.12, 0.24]
**Total (95% CI)**			**1676**	**1642**	**100.0%**	**−0.10 [−0.18, −0.02]**

Heterogeneity: $Tau^2 = 0.01$; $Chi^2 = 18.06$, df = 14 (P = 0.20); $I^2 = 22\%$
Test for overall effect: Z = 2.37 (P = 0.02)

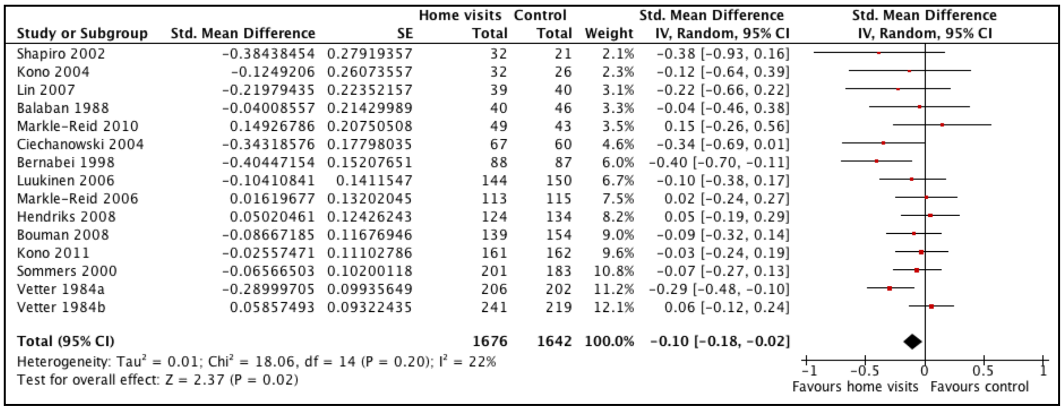

Analysis 76: Psychiatric (anxiety and depression) at each follow-up interval

Study or Subgroup	Std. Mean Difference	SE	Home visits Total	Control Total	Weight	Std. Mean Difference IV, Random, 95% CI
**2.95.1 01-11 months**						
Lin 2007	−0.21979435	0.22352157	39	40	17.3%	−0.22 [−0.66, 0.22]
Markle-Reid 2010	0.14926786	0.20750508	49	43	18.2%	0.15 [−0.26, 0.56]
Ciechanowski 2004	−0.80517631	0.18094344	69	62	19.6%	−0.81 [−1.16, −0.45]
Markle-Reid 2006	0.01619677	0.13202045	113	115	22.2%	0.02 [−0.24, 0.27]
Hendriks 2008	0.04129397	0.12061958	131	143	22.7%	0.04 [−0.20, 0.28]
**Subtotal (95% CI)**			**401**	**403**	**100.0%**	**−0.16 [−0.47, 0.16]**
Heterogeneity: $Tau^2 = 0.10$; $Chi^2 = 19.05$, df = 4 (P = 0.0008); $I^2 = 79\%$						
Test for overall effect: Z = 0.97 (P = 0.33)						
**2.95.2 12-23 months**						
Shapiro 2002	−0.38438454	0.27919357	32	21	3.4%	−0.38 [−0.93, 0.16]
Kono 2004	−0.1249206	0.26073557	32	26	3.9%	−0.12 [−0.64, 0.39]
Balaban 1988	−0.04008557	0.21429989	40	46	5.8%	−0.04 [−0.46, 0.38]
Ciechanowski 2004	−0.34318576	0.17798035	67	60	8.3%	−0.34 [−0.69, 0.01]
Bernabei 1998	−0.40447154	0.15207651	88	87	11.2%	−0.40 [−0.70, −0.11]
Luukinen 2006	−0.10410841	0.1411547	144	150	12.9%	−0.10 [−0.38, 0.17]
Hendriks 2008	0.05020461	0.12426243	124	134	16.3%	0.05 [−0.19, 0.29]
Bouman 2008	−0.0923089	0.11675516	139	154	18.3%	−0.09 [−0.32, 0.14]
Kono 2011	−0.04926539	0.11104022	161	162	20.0%	−0.05 [−0.27, 0.17]
**Subtotal (95% CI)**			**827**	**840**	**100.0%**	**−0.13 [−0.23, −0.02]**
Heterogeneity: $Tau^2 = 0.00$; $Chi^2 = 8.45$, df = 8 (P = 0.39); $I^2 = 5\%$						
Test for overall effect: Z = 2.41 (P = 0.02)						
**2.95.3 24-35 months**						
Bouman 2008	−0.08667185	0.11676946	139	154	17.4%	−0.09 [−0.32, 0.14]
Kono 2011	−0.02557471	0.11102786	161	162	18.5%	−0.03 [−0.24, 0.19]
Sommers 2000	−0.06566503	0.10200118	201	183	20.5%	−0.07 [−0.27, 0.13]
Vetter 1984a	−0.28999705	0.09935649	206	202	21.1%	−0.29 [−0.48, −0.10]
Vetter 1984b	0.05857493	0.09322435	241	219	22.6%	0.06 [−0.12, 0.24]
**Subtotal (95% CI)**			**948**	**920**	**100.0%**	**−0.08 [−0.20, 0.04]**
Heterogeneity: $Tau^2 = 0.01$; $Chi^2 = 6.94$, df = 4 (P = 0.14); $I^2 = 42\%$						
Test for overall effect: Z = 1.32 (P = 0.19)						

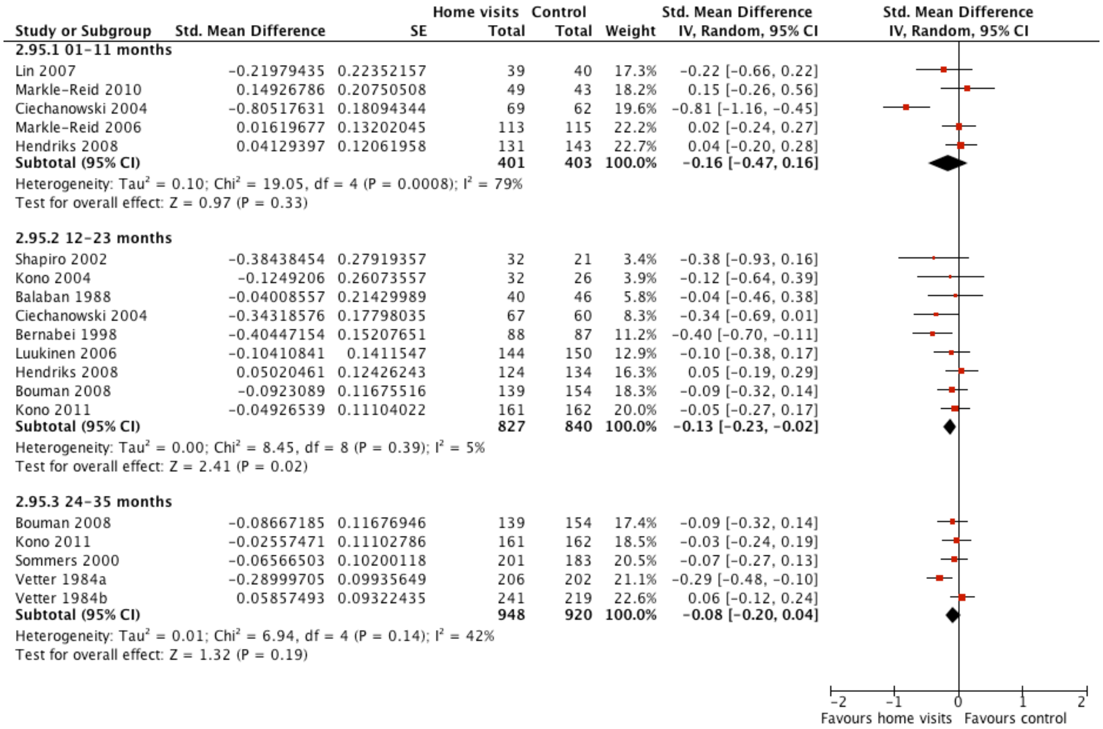

Analysis 77: Psychiatric (anxiety and depression); focus of visit subgroups

Study or Subgroup	Std. Mean Difference	SE	Home visits Total	Control Total	Weight	Std. Mean Difference IV, Random, 95% CI
**2.96.1 Falls Only**						
Lin 2007	−0.21979435	0.22352157	39	40	3.1%	−0.22 [−0.66, 0.22]
Luukinen 2006	−0.10410841	0.1411547	144	150	6.7%	−0.10 [−0.38, 0.17]
**Subtotal (95% CI)**			**183**	**190**	**9.8%**	**−0.14 [−0.37, 0.10]**
Heterogeneity: $Tau^2 = 0.00$; $Chi^2 = 0.19$, df = 1 (P = 0.66); $I^2 = 0\%$						
Test for overall effect: Z = 1.15 (P = 0.25)						
**2.96.2 MGA Only**						
Shapiro 2002	−0.38438454	0.27919357	32	21	2.1%	−0.38 [−0.93, 0.16]
Kono 2004	−0.1249206	0.26073557	32	26	2.3%	−0.12 [−0.64, 0.39]
Balaban 1988	−0.04008557	0.21429989	40	46	3.3%	−0.04 [−0.46, 0.38]
Bernabei 1998	−0.40447154	0.15207651	88	87	6.0%	−0.40 [−0.70, −0.11]
Markle-Reid 2006	0.01619677	0.13202045	113	115	7.5%	0.02 [−0.24, 0.27]
Bouman 2008	−0.08667185	0.11676946	139	154	9.0%	−0.09 [−0.32, 0.14]
Kono 2011	−0.02557471	0.11102786	161	162	9.6%	−0.03 [−0.24, 0.19]
Sommers 2000	−0.06566503	0.10200118	201	183	10.8%	−0.07 [−0.27, 0.13]
Vetter 1984a	−0.28999705	0.09935649	206	202	11.2%	−0.29 [−0.48, −0.10]
Vetter 1984b	0.05857493	0.09322435	241	219	12.1%	0.06 [−0.12, 0.24]
**Subtotal (95% CI)**			**1253**	**1215**	**73.8%**	**−0.11 [−0.21, −0.01]**
Heterogeneity: $Tau^2 = 0.01$; $Chi^2 = 13.03$, df = 9 (P = 0.16); $I^2 = 31\%$						
Test for overall effect: Z = 2.11 (P = 0.04)						
**2.96.3 Both**						
Markle-Reid 2010	0.14926786	0.20750508	49	43	3.5%	0.15 [−0.26, 0.56]
Hendriks 2008	0.05020461	0.12426243	124	134	8.2%	0.05 [−0.19, 0.29]
**Subtotal (95% CI)**			**173**	**177**	**11.7%**	**0.08 [−0.13, 0.29]**
Heterogeneity: $Tau^2 = 0.00$; $Chi^2 = 0.17$, df = 1 (P = 0.68); $I^2 = 0\%$						
Test for overall effect: Z = 0.72 (P = 0.47)						
**2.96.4 Neither**						
Ciechanowski 2004	−0.34318576	0.17798035	67	60	4.6%	−0.34 [−0.69, 0.01]
**Subtotal (95% CI)**			**67**	**60**	**4.6%**	**−0.34 [−0.69, 0.01]**
Heterogeneity: Not applicable						
Test for overall effect: Z = 1.93 (P = 0.05)						
**Total (95% CI)**			**1676**	**1642**	**100.0%**	**−0.10 [−0.18, −0.02]**
Heterogeneity: $Tau^2 = 0.01$; $Chi^2 = 18.06$, df = 14 (P = 0.20); $I^2 = 22\%$						
Test for overall effect: Z = 2.37 (P = 0.02)						
Test for subgroup differences: $Chi^2 = 4.71$, df = 3 (P = 0.19), $I^2 = 36.3\%$						

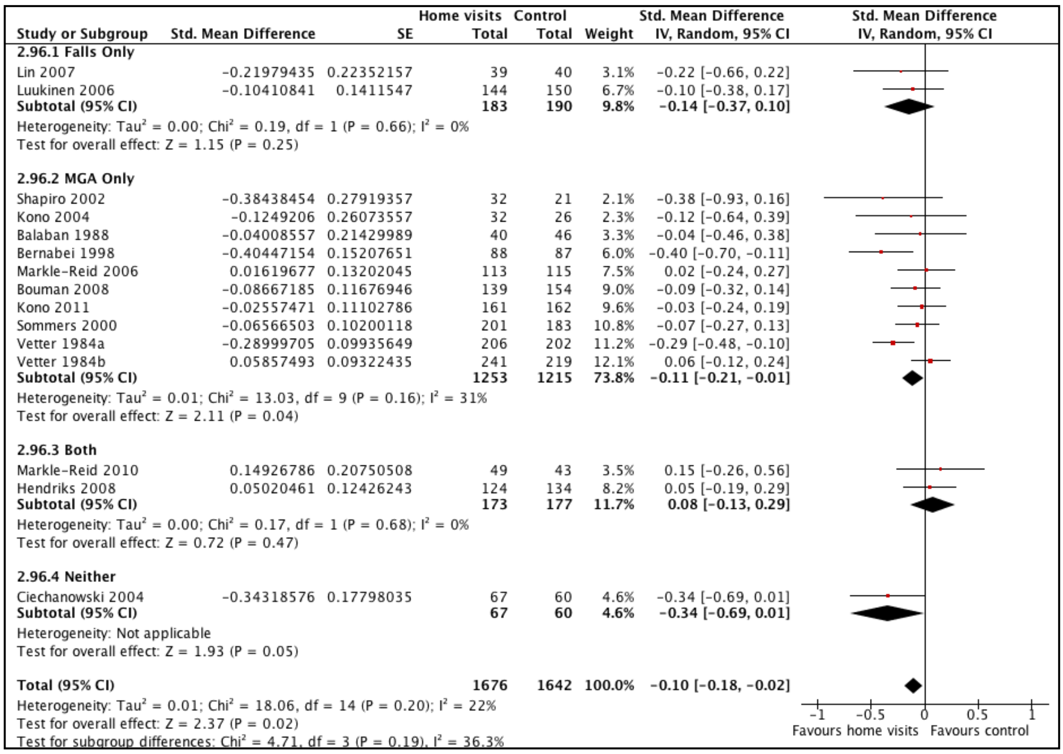

Analysis 78: Psychiatric (anxiety and depression); age of participants subgroups

Study or Subgroup	Std. Mean Difference	SE	Home visits Total	Control Total	Weight	Std. Mean Difference IV, Random, 95% CI
**2.97.1 <=70**						
Balaban 1988	−0.04008557	0.21429989	40	46	3.4%	−0.04 [−0.46, 0.38]
**Subtotal (95% CI)**			**40**	**46**	**3.4%**	**−0.04 [−0.46, 0.38]**
Heterogeneity: Not applicable						
Test for overall effect: Z = 0.19 (P = 0.85)						
**2.97.2 71–75**						
Ciechanowski 2004	−0.34318576	0.17798035	67	60	4.7%	−0.34 [−0.69, 0.01]
Hendriks 2008	0.05020461	0.12426243	124	134	8.5%	0.05 [−0.19, 0.29]
**Subtotal (95% CI)**			**191**	**194**	**13.2%**	**−0.13 [−0.51, 0.26]**
Heterogeneity: $Tau^2 = 0.05$; $Chi^2 = 3.28$, df = 1 (P = 0.07); $I^2 = 70\%$						
Test for overall effect: Z = 0.64 (P = 0.52)						
**2.97.3 76–80**						
Shapiro 2002	−0.38438454	0.27919357	32	21	2.1%	−0.38 [−0.93, 0.16]
Lin 2007	−0.21979435	0.22352157	39	40	3.2%	−0.22 [−0.66, 0.22]
Bouman 2008	−0.08667185	0.11676946	139	154	9.3%	−0.09 [−0.32, 0.14]
Kono 2011	−0.02557471	0.11102786	161	162	10.0%	−0.03 [−0.24, 0.19]
Sommers 2000	−0.06566503	0.10200118	201	183	11.2%	−0.07 [−0.27, 0.13]
Vetter 1984a	−0.28999705	0.09935649	206	202	11.6%	−0.29 [−0.48, −0.10]
Vetter 1984b	0.05857493	0.09322435	241	219	12.6%	0.06 [−0.12, 0.24]
**Subtotal (95% CI)**			**1019**	**981**	**60.1%**	**−0.10 [−0.21, 0.01]**
Heterogeneity: $Tau^2 = 0.01$; $Chi^2 = 8.43$, df = 6 (P = 0.21); $I^2 = 29\%$						
Test for overall effect: Z = 1.80 (P = 0.07)						
**2.97.4 81–85**						
Kono 2004	−0.1249206	0.26073557	32	26	2.4%	−0.12 [−0.64, 0.39]
Bernabei 1998	−0.40447154	0.15207651	88	87	6.2%	−0.40 [−0.70, −0.11]
Markle-Reid 2006	0.01619677	0.13202045	113	115	7.7%	0.02 [−0.24, 0.27]
**Subtotal (95% CI)**			**233**	**228**	**16.3%**	**−0.17 [−0.46, 0.12]**
Heterogeneity: $Tau^2 = 0.03$; $Chi^2 = 4.38$, df = 2 (P = 0.11); $I^2 = 54\%$						
Test for overall effect: Z = 1.16 (P = 0.25)						
**2.97.5 86+**						
Luukinen 2006	−0.10410841	0.1411547	144	150	7.0%	−0.10 [−0.38, 0.17]
**Subtotal (95% CI)**			**144**	**150**	**7.0%**	**−0.10 [−0.38, 0.17]**
Heterogeneity: Not applicable						
Test for overall effect: Z = 0.74 (P = 0.46)						
**Total (95% CI)**			**1627**	**1599**	**100.0%**	**−0.11 [−0.19, −0.02]**
Heterogeneity: $Tau^2 = 0.01$; $Chi^2 = 16.65$, df = 13 (P = 0.22); $I^2 = 22\%$						
Test for overall effect: Z = 2.55 (P = 0.01)						
Test for subgroup differences: $Chi^2 = 0.31$, df = 4 (P = 0.99), $I^2 = 0\%$						

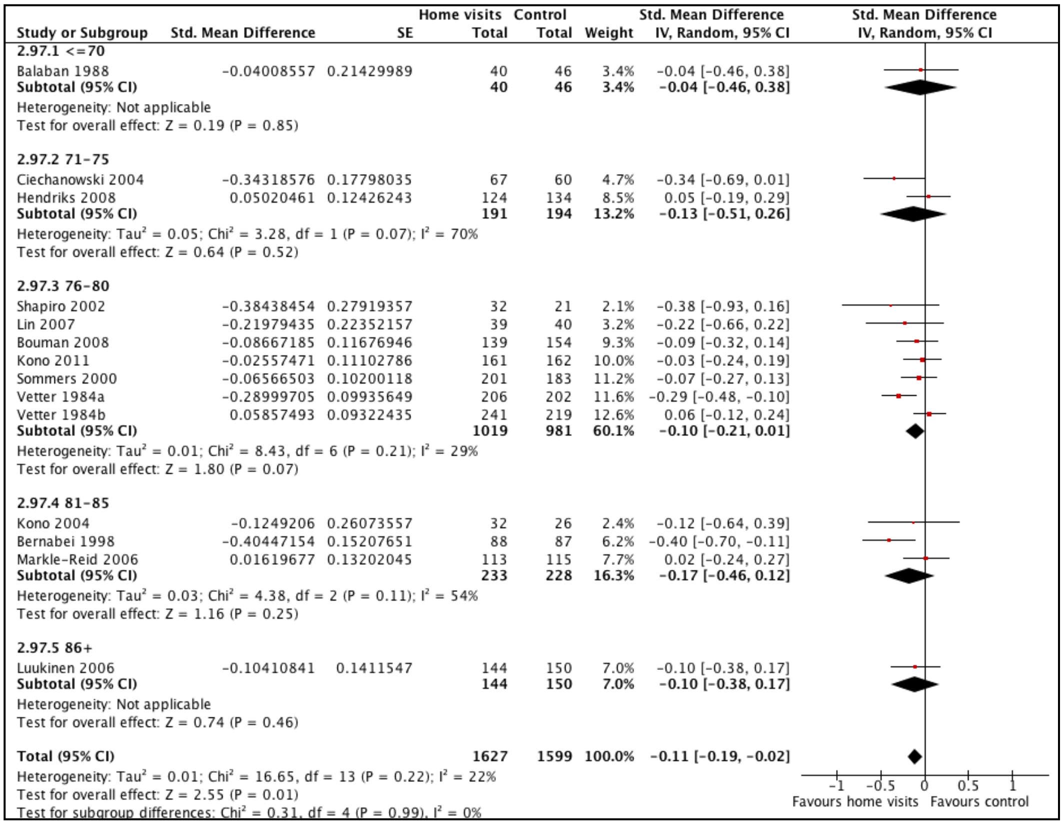

Analysis 79: Psychiatric (anxiety and depression); type of visitor subgroups

Study or Subgroup	Std. Mean Difference	SE	Home visits Total	Control Total	Weight	Std. Mean Difference IV, Random, 95% CI
**2.98.1 Nurse**						
Shapiro 2002	−0.38438454	0.27919357	32	21	2.1%	−0.38 [−0.93, 0.16]
Kono 2004	−0.1249206	0.26073557	32	26	2.3%	−0.12 [−0.64, 0.39]
Markle-Reid 2006	0.01619677	0.13202045	113	115	7.5%	0.02 [−0.24, 0.27]
Bouman 2008	−0.08667185	0.11676946	139	154	9.0%	−0.09 [−0.32, 0.14]
**Subtotal (95% CI)**			**316**	**316**	**20.8%**	**−0.08 [−0.23, 0.08]**
Heterogeneity: $Tau^2 = 0.00$; $Chi^2 = 1.75$, df = 3 (P = 0.63); $I^2 = 0\%$						
Test for overall effect: Z = 0.97 (P = 0.33)						
**2.98.2 Other**						
Lin 2007	−0.21979435	0.22352157	39	40	3.1%	−0.22 [−0.66, 0.22]
Ciechanowski 2004	−0.34318576	0.17798035	67	60	4.6%	−0.34 [−0.69, 0.01]
Bernabei 1998	−0.40447154	0.15207651	88	87	6.0%	−0.40 [−0.70, −0.11]
Vetter 1984a	−0.28999705	0.09935649	206	202	11.2%	−0.29 [−0.48, −0.10]
Vetter 1984b	0.05857493	0.09322435	241	219	12.1%	0.06 [−0.12, 0.24]
**Subtotal (95% CI)**			**641**	**608**	**36.9%**	**−0.22 [−0.42, −0.02]**
Heterogeneity: $Tau^2 = 0.03$; $Chi^2 = 10.82$, df = 4 (P = 0.03); $I^2 = 63\%$						
Test for overall effect: Z = 2.18 (P = 0.03)						
**2.98.3 Combined**						
Balaban 1988	−0.04008557	0.21429989	40	46	3.3%	−0.04 [−0.46, 0.38]
Markle-Reid 2010	0.14926786	0.20750508	49	43	3.5%	0.15 [−0.26, 0.56]
Luukinen 2006	−0.10410841	0.1411547	144	150	6.7%	−0.10 [−0.38, 0.17]
Hendriks 2008	0.05020461	0.12426243	124	134	8.2%	0.05 [−0.19, 0.29]
Kono 2011	−0.02557471	0.11102786	161	162	9.6%	−0.03 [−0.24, 0.19]
Sommers 2000	−0.06566503	0.10200118	201	183	10.8%	−0.07 [−0.27, 0.13]
**Subtotal (95% CI)**			**719**	**718**	**42.2%**	**−0.02 [−0.13, 0.08]**
Heterogeneity: $Tau^2 = 0.00$; $Chi^2 = 1.55$, df = 5 (P = 0.91); $I^2 = 0\%$						
Test for overall effect: Z = 0.42 (P = 0.67)						
**Total (95% CI)**			**1676**	**1642**	**100.0%**	**−0.10 [−0.18, −0.02]**
Heterogeneity: $Tau^2 = 0.01$; $Chi^2 = 18.06$, df = 14 (P = 0.20); $I^2 = 22\%$						
Test for overall effect: Z = 2.37 (P = 0.02)						
Test for subgroup differences: $Chi^2 = 2.96$, df = 2 (P = 0.23), $I^2 = 32.5\%$						

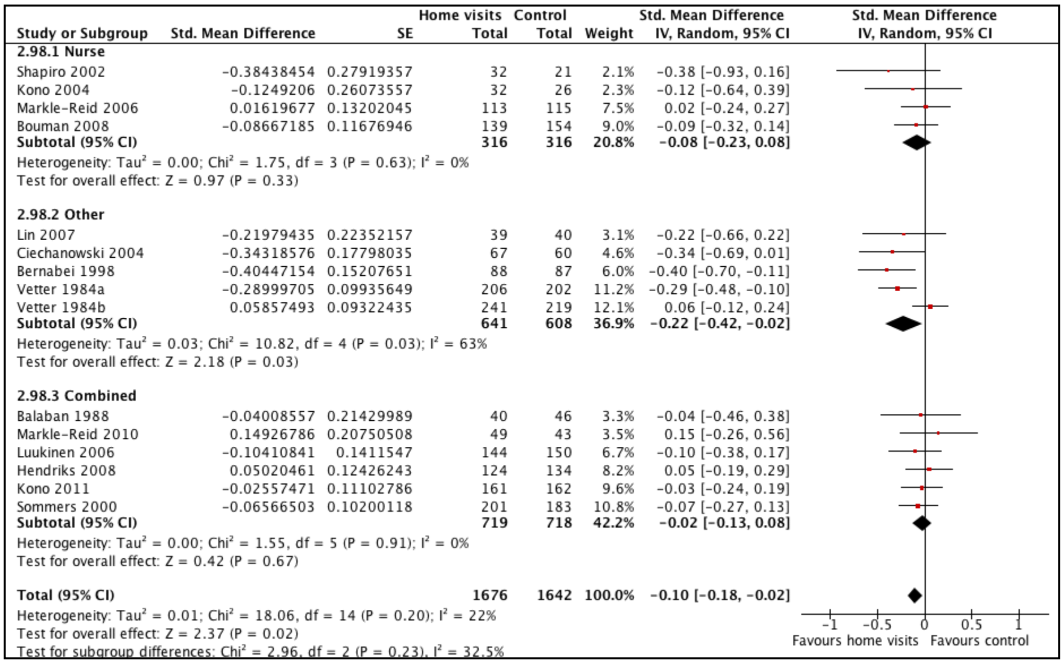

Analysis 80: Psychiatric (anxiety and depression); number of visits subgroups

Study or Subgroup	Std. Mean Difference	SE	Home visits Total	Control Total	Weight	Std. Mean Difference IV, Random, 95% CI
**2.99.1 1 Visit**						
Hendriks 2008	0.05020461	0.12426243	124	134	8.7%	0.05 [−0.19, 0.29]
**Subtotal (95% CI)**			**124**	**134**	**8.7%**	**0.05 [−0.19, 0.29]**
Heterogeneity: Not applicable						
Test for overall effect: Z = 0.40 (P = 0.69)						
**2.99.2 2–4 Visits**						
Kono 2004	−0.1249206	0.26073557	32	26	2.6%	−0.12 [−0.64, 0.39]
Luukinen 2006	−0.10410841	0.1411547	144	150	7.2%	−0.10 [−0.38, 0.17]
Kono 2011	−0.02557471	0.11102786	161	162	10.1%	−0.03 [−0.24, 0.19]
Vetter 1984a	−0.28999705	0.09935649	206	202	11.5%	−0.29 [−0.48, −0.10]
Vetter 1984b	0.05857493	0.09322435	241	219	12.4%	0.06 [−0.12, 0.24]
**Subtotal (95% CI)**			**784**	**759**	**43.9%**	**−0.09 [−0.23, 0.05]**
Heterogeneity: $Tau^2 = 0.01$; $Chi^2 = 6.95$, df = 4 (P = 0.14); $I^2 = 42\%$						
Test for overall effect: Z = 1.25 (P = 0.21)						
**2.99.3 5 or More Visits**						
Lin 2007	−0.21979435	0.22352157	39	40	3.4%	−0.22 [−0.66, 0.22]
Markle-Reid 2010	0.14926786	0.20750508	49	43	3.9%	0.15 [−0.26, 0.56]
Ciechanowski 2004	−0.34318576	0.17798035	67	60	5.0%	−0.34 [−0.69, 0.01]
Bernabei 1998	−0.40447154	0.15207651	88	87	6.5%	−0.40 [−0.70, −0.11]
Markle-Reid 2006	0.01619677	0.13202045	113	115	8.0%	0.02 [−0.24, 0.27]
Bouman 2008	−0.08667185	0.11676946	139	154	9.4%	−0.09 [−0.32, 0.14]
Sommers 2000	−0.06566503	0.10200118	201	183	11.2%	−0.07 [−0.27, 0.13]
**Subtotal (95% CI)**			**696**	**682**	**47.4%**	**−0.13 [−0.26, 0.00]**
Heterogeneity: $Tau^2 = 0.01$; $Chi^2 = 8.38$, df = 6 (P = 0.21); $I^2 = 28\%$						
Test for overall effect: Z = 1.91 (P = 0.06)						
**Total (95% CI)**			**1604**	**1575**	**100.0%**	**−0.09 [−0.18, −0.01]**
Heterogeneity: $Tau^2 = 0.01$; $Chi^2 = 16.90$, df = 12 (P = 0.15); $I^2 = 29\%$						
Test for overall effect: Z = 2.14 (P = 0.03)						
Test for subgroup differences: $Chi^2 = 1.57$, df = 2 (P = 0.46), $I^2 = 0\%$						

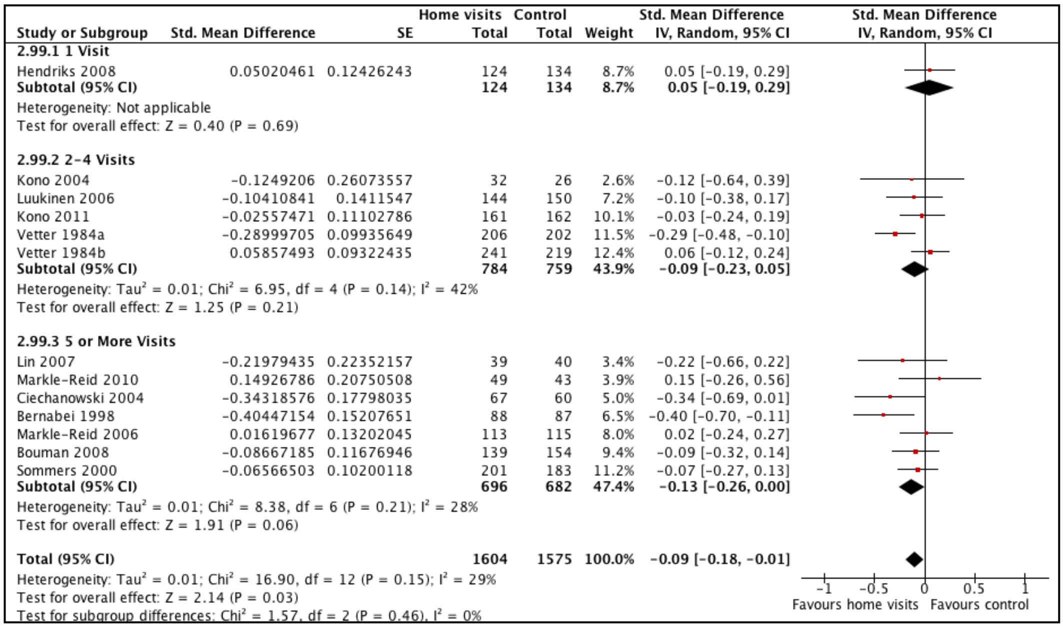

Supplement: Figure S5 — Results for each follow-up interval, longest follow-up, and subgroup analyses. (PDF) [file pone.0089257.s005.pdf]
