# Supplementary material for: Preventive Home Visits for Mortality, Morbidity, and Institutionalization in Older Adults: A Systematic Review and Meta-Analysis
Source: PLoS One. 2014 Mar 12;9(3):e89257. doi: 10.1371/journal.pone.0089257 (PMC3951196; doi:10.1371/journal.pone.0089257)
Supplement: Figure S6 — Extended table of included studies. (PDF) [file pone.0089257.s006.pdf]

Figure S6: Extended table of included studies

Balaban 1988

**Methods**	**Place of recruitment**: Family practice; method of solicitation not reported.<br>**Inclusion criteria**: aged 65+, partial or total disability, living alone, unlikely to make contact with a physician.
**Participants**	**Number randomised**: 103 (Intervention) / 95 (Comparison)<br>**Mean age**: 68.5<br>**Sex (% female)**: 76.0%<br>**Mortality per year in the comparison group**: 12.6%<br>**Living alone (%):** Not reported
**Interventions**	**Visitors' professional group**: Physician, nurse, other health professionals<br>**Frequency and duration of visits**: Not reported<br>**Fall prevention:** No<br>**Included exercise**: No<br>**Included multidimensional geriatric assessment:** Yes<br>**Description of intervention:** Visitors assessed medical and social needs (including diagnostic and therapeutic medical care), post-hospital follow up, education, counselling, and social service referrals.<br>**Description of comparison**: Usual care<br>**Implementation (fidelity and participant compliance)**: 92.2% of intervention participants received one or more home visits over the study period. 30.0% of comparison participants received one or more visits; however, the majority of these visits occurred shortly before the follow-up study was conducted.
**Outcomes**	**Functioning ADL/IADL:** Barthal Activities of Daily Living Index (19.5 months)<br>**Health Related QoL:** Global Health Status Visual Analog (19.5 months); Quality of Well-Being (19.5 months)<br>**Hospitalisation Admissions:**<br>Total number of hospital admissions (19.5 months)<br>**Hospitalisation Days:** Total number of days in hospital (19.5 months)<br>**Mortality:** Total number of deaths (19.5 months)<br>**Psychiatric Anxiety and Depression:** Beck Depression Inventory (19.5 months); Philadelphia Geriatrics Center Morale Scale (20 months)
**Notes**	**Location**: Northeast, United States<br>**Funding Source**: Robert Wood Johnson Foundation<br>**Other notes**:

Risk of bias table

**Bias**	**Authors' judgement**	**Support for judgement**
Random sequence generation (selection bias)	Low risk	Quote: "randomization tables were used"
Allocation concealment (selection bias)	High risk	Quote: "the director of the home visit program recalled that the home visit staff had allowed 'one or two' patients to be transferred from the experimental group to control group"
Blinding (performance bias and detection bias)	Low risk	Quote: "data collection began with an abstract of the Family Practice Center patient charts for all 198 study participants.... Data were collected on precoded forms"
Incomplete outcome data (attrition bias)	Low risk	Quote: "data collection began with an abstract of the Family Practice Center patient charts for all 198 study participants... Of the 198 participants, 51 died. The interviewers were able to collect information on 143 of the remaining 147 patients" "The in-person interview population included all patients who completed the full battery of questionnaires"<br>Comment: Some outcome data were collected for 97.3% of living participants, but only 86 of 143 completed the in-person interview.
Selective reporting (reporting bias)	Unclear risk	Study does not reference a trial protocol

Bernabei 1998

**Methods**	**Place of recruitment**: Local town practice; method of solicitation not reported.<br>**Inclusion criteria**: aged 65+, received home health services or home assistance programmes.
**Participants**	**Number randomised**: 100 (Intervention) / 100 (Comparison)<br>**Mean age**: 81.0<br>**Sex (% female)**: 70.5%<br>**Mortality per year in the comparison group**: 13.0%<br>**Living alone (%):** 50.0%
**Interventions**	**Visitors' professional group**: Case managers<br>**Frequency and duration of visits**: 6 visits over 12 months<br>**Fall prevention:** No<br>**Included exercise**: No<br>**Included multidimensional geriatric assessment:** Yes<br>**Description of intervention:** Visits involved case management and care planning. Visitors assessed physical and cognitive functioning, mood, drug treatments, and the number of home visits by a general practitioner.<br>**Description of comparison**: Usual care.<br>**Implementation (fidelity and participant compliance)**: All participants were already receiving home health services or home assistance programmes at baseline. Comparison participants may also have received some home visits.
**Outcomes**	**A&E People**: Total number of people admitted to A&E (12 months)<br>**Functioning ADL/IADL:** Activities of Daily Living (12 months); Instrumental Activities of Daily Living Items (12 months)<br>**Functioning Cognitive:** Short portable mental status questionnaire (12 months)<br>**Hospitalisation Days:** Total number of days in hospital (12 months)<br>**Hospitalisation People:** Total number of people hospitalised (12 months)<br>**Institutionalisation Days:** Total number of days in a nursing home (12 months)<br>**Institutionalisation People:** Total number of people admitted to nursing home (12 months)<br>**Mortality:** Total number of deaths (12 months)<br>**Psychiatric Anxiety and Depression:** Geriatric Depression Scale (12 months)
**Notes**	**Location**: Rovereto, Italy<br>**Funding Source**: Progetto Finalizzato Invecchiamento, National Research Council<br>**Other notes**: Estimated cost of intervention was £744 per participant

Risk of bias table

**Bias**	**Authors' judgement**	**Support for judgement**
Random sequence generation (selection bias)	Low risk	Quote: "subjects were randomly stratified by age and sex according to a computer generated list"
Allocation concealment (selection bias)	Unclear risk	Quote: "subjects were randomly stratified by age and sex according to a computer generated list"
Blinding (performance bias and detection bias)	Low risk	Quote: "information on outcomes ... was collected every 2 months by a research assistant unaware of patients' assignments"
Incomplete outcome data (attrition bias)	Low risk	Data missing for only 1 participant
Selective reporting (reporting bias)	Unclear risk	Study does not reference a trial protocol

Bouman 2008

**Methods**	**Place of recruitment**: Population registry of municipality; solicited by mail.<br>**Inclusion criteria**: aged 70-84, self-reported poor health.<br>Excluded if: moderate to good health status, received regular home nursing care, or were on waiting list for admission to nursing homes.
**Participants**	**Number randomised**: 160 (Intervention) / 170 (Comparison)<br>**Mean age**: 75.7<br>**Sex (% female)**: 60.0%<br>**Mortality per year in the comparison group**: 6.8%<br>**Living alone (%):** 35.0%
**Interventions**	**Visitors' professional group**: Nurses<br>**Frequency and duration of visits**: 6.9 visits over 18 months<br>**Fall prevention:** No<br>**Included exercise**: No<br>**Included multidimensional geriatric assessment:** Yes<br>**Description of intervention:** Visits involved health assessment and diagnostics. Nurses recorded health problems and provided advice to address them, and they provided follow-up support by phone.<br>**Description of comparison**: Usual care.<br>**Implementation (fidelity and participant compliance)**: 77.5% of intervention participants completed all 8 visits. Intervention participants complied with 65.0% of referrals and 58.0% of advice; overall compliance was 61.0%.
**Outcomes**	**A&E Number:** Total number of paramedic consultations (18, 24 months)<br>**A&E People**: Total number of people receiving paramedic consultations (18, 24 months)<br>**Functioning ADL/IADL:** Groningen Activity Restriction Scale A (12, 18, 24 months); Groningen Activity Restriction Scale (12, 18, 24 months)<br>**Functioning Cognitive:** Mini Mental State Examination-12 (18 months)<br>**Health Related QoL:** RAND-36 Health Change Survey (12, 18, 24 months); Self-rated health (12, 18, 24 months)<br>**Hospitalisation Days:** Number of days in hospital per person (18, 24 months)<br>**Hospitalisation People:** Total number of people hospitalised (18, 24 months)<br>**Institutionalisation Days:** Total number of days in a nursing home (24 months)<br>**Institutionalisation People:** Total number of people admitted to nursing home (24 months)<br>**Mortality:** Total number of deaths (18, 24 months)<br>**Psychiatric Anxiety and Depression:** Medical Outcomes Study 20-item Short Form Survey mental health score (12, 18, 24 months); Geriatric Depression Scale (18 months)
**Notes**	**Location**: Sittard, Netherlands<br>**Funding Source**: Netherlands Organisation for Health Research and Development (ZonMw), The Hague, Netherlands<br>**Other notes**: Estimated cost of intervention was €753 per participant

Risk of bias table

**Bias**	**Authors' judgement**	**Support for judgement**
Random sequence generation (selection bias)	Low risk	Quote: "computer-generated randomization list"
Allocation concealment (selection bias)	Unclear risk	Quote: "the random allocation sequence was conducted using a computer-generated randomization list with a block length of 4"
Blinding (performance bias and detection bias)	Low risk	Quote: "individual interviews conducted by six trained, independent interviewers, blinded to group allocation"
Incomplete outcome data (attrition bias)	High risk	10.0% of intervention participants compared to 4.7% of control participants self-withdrew from the study. Last observation carried forward (LOCF) used to impute missing data<br>Comment: Using LOCF may bias estimate in favour of the intervention, as health outcomes tend to deteriorate with time in this population
Selective reporting (reporting bias)	Unclear risk	Study does not reference a trial protocol

Byles 2004

**Methods**	**Place of recruitment**: Australian Department of Veterans' Affairs; solicited by letter.<br>**Inclusion criteria**: aged 70+, lived in 1 of 10 eligible geographical areas, received full entitlements from Department of Veterans' Affairs.
**Participants**	**Number randomised**: 942 (Intervention) / 627 (Comparison)<br>**Mean age**: 77.3<br>**Sex (% female)**: Not reported<br>**Mortality per year in the comparison group**: 3.2%<br>**Living alone (%):** Not reported
**Interventions**	**Visitors' professional group**: Nurses, social workers, psychologists, physical therapists<br>**Frequency and duration of visits**: 4.5 visits over 36 months<br>**Fall prevention:** No<br>**Included exercise**: No<br>**Included multidimensional geriatric assessment:** Yes<br>**Description of intervention:** Visits involved assessment of various physical and mental health problems, as well as environmental hazards. Visitors provided feedback and health materials. The 3 month follow-up included a report to participant's general practitioner.<br>**Description of comparison**: Usual care.<br>**Implementation (fidelity and participant compliance)**: Although the checklist was standardised for all visits, visits varied in emphasis because health professionals were asked to apply their professional judgment and skills in determining feedback.
**Outcomes**	**Health Related QoL:** Medical Outcomes Study Short Form Health Survey-36 items for Mental Health (24, 36 months) and Physical Health (36 months)<br>**Hospitalisation People:** Total number of people hospitalised (36 months)<br>**Institutionalisation People:** Total number of people in a nursing home at the completion of the study (36 months)<br>**Mortality:** Total number of deaths (36 months)
**Notes**	**Location**: New South Wales and Queensland, Australia<br>**Funding Source**: Australian Dept of Veterans' Affairs<br>**Other notes**:

Risk of bias table

**Bias**	**Authors' judgement**	**Support for judgement**
Random sequence generation (selection bias)	Low risk	Quote: "randomly allocated by computer using Statistical Applications Software (SAS) to one of four intervention groups or the control group"
Allocation concealment (selection bias)	Low risk	Quote: "participants who returned written consent to the study team were entered into the study database by the data manager and randomly allocated by computer using Statistical Applications Software (SAS) to one of four intervention groups or the control group"
Blinding (performance bias and detection bias)	Low risk	Quote: "interviewers were not part of the intervention team and were blinded to participants' trial status"
Incomplete outcome data (attrition bias)	Low risk	Quote: "an interaction between time and treatment group ... did not show a systematic difference in the occurrence of missing data over time according to treatment group"<br>Comment: Missing outcome data balanced in numbers between groups, with similar reasons for missing data between groups
Selective reporting (reporting bias)	Unclear risk	Study does not reference a trial protocol

Campbell 1999

**Methods**	**Place of recruitment**: General practices; method of solicitation not reported.<br>**Inclusion criteria**: aged 80+, able to move around home, not receiving physiotherapy.
**Participants**	**Number randomised**: 116 (Intervention) / 117 (Comparison)<br>**Mean age**: 83.9<br>**Sex (% female)**: 100%<br>**Mortality per year in the comparison group**: 3.0%<br>**Living alone (%):** 77.0%
**Interventions**	**Visitors' professional group**: Physical therapist<br>**Frequency and duration of visits**: 4 visits over 12 months<br>**Fall prevention:** Yes<br>**Included exercise**: Yes<br>**Included multidimensional geriatric assessment:** No<br>**Description of intervention:** Visitors prescribed exercises and walking plan. Conducted regular phone calls to maintain motiviation.<br>**Description of comparison**: Attention-matched control.<br>**Implementation (fidelity and participant compliance)**: At 1 year, 42.1% of intervention participants were still completing the programme 3 or more times a week. After 2 years, 43.6% of the remaining intervention participants were still exercising at least 3 times per week.
**Outcomes**	**Falls Number:** Total number of falls (12, 24 months)<br>**Falls People:** Total number of people who experienced a fall (12 months)<br>**Falls Subjective:** Falls Self-Efficacy scale (12 months)<br>**Injuries People:** Total number of people experiencing injury due to fall (12 months)<br>**Mortality:** Total number of deaths (12, 24 months)
**Notes**	**Location**: Dunedin, New Zealand<br>**Funding Source**: Accident Rehabilitation and Compensation Insurance Corporation of New Zealand and Department of Veterans' Affairs, United States<br>**Other notes**:

Risk of bias table

**Bias**	**Authors' judgement**	**Support for judgement**
Random sequence generation (selection bias)	Low risk	Quote: "the group allocation schedule was developed by a statistician using computer generated random numbers"
Allocation concealment (selection bias)	Low risk	Quote: "the [random sequence] list was held off site by an independent person"
Blinding (performance bias and detection bias)	Low risk	Quote: "the investigator classifying fall events remained blind to group allocation"
Incomplete outcome data (attrition bias)	High risk	64.7% of intervention participants and 47.0% of control participants withdrew after 2 years. The authors did not report their intention-to-treat procedure.
Selective reporting (reporting bias)	Unclear risk	Study does not reference a trial protocol

Campbell 2005

**Methods**	**Place of recruitment**: Optometry and opthamology clinics; method of solicitation not reported.<br>**Inclusion criteria**: aged 75+, poor "distance visual acuity" in the better eye.
**Participants**	**Number randomised**: 98 (Intervention) / 96 (Comparison)<br>**Mean age**: 83.6<br>**Sex (% female)**: 66.5%<br>**Mortality per year in the comparison group**: 7.3%<br>**Living alone (%):** 53.2%
**Interventions**	**Visitors' professional group**: Occupational therapist; physiotherapist<br>**Frequency and duration of visits**: 6 visits over 12 months<br>**Fall prevention:** Yes<br>**Included exercise**: Yes<br>**Included multidimensional geriatric assessment:** No<br>**Description of intervention:** Visitors identified and then recommended removal or modification of hazards, provision of new equipment, or making behavioural changes.<br>**Description of comparison**: Social visit.<br>**Implementation (fidelity and participant compliance)**: 66.7% of intervention participants were prescribed an exercise component to their treatment plan. Of these, 89.9% reported complying partially or completely with one or more of the recommendations made by the occupational therapist.
**Outcomes**	**A&E Number:** Number of fall-related visits to an A&E department per person (12 months)<br>**Falls Number:** Number of falls per person (12 months)<br>**Falls People:** Total number of people who experienced a fall (12 months)<br>**Injuries Number:** Number of injurious falls per person (12 months)<br>**Mortality:** Total number of deaths (12 months)
**Notes**	**Location**: Dunedin and Auckland, New Zealand<br>**Funding Source**: Health Research Council of New Zealand<br>**Other notes**: Only two groups from a four-arm trial were used: exercise and home safety (intervention group) and social visit (control).<br>Estimated cost of intervention was NZ$325 per participant

Risk of bias table

Bias	Authors' judgement	Support for judgement
Random sequence generation (selection bias)	Low risk	Quote: "a statistician developed the group allocation schedule using computer generated random numbers"
Allocation concealment (selection bias)	Low risk	Quote: "the schedule was held by an independent person at a separate site and was accessed by a research administrator for the study, who telephoned after each baseline assessment was completed"
Blinding (performance bias and detection bias)	Low risk	Quote: "the independent assessor ... remained blind to group allocation"
Incomplete outcome data (attrition bias)	Low risk	Rate of and reasons for missing data were similar across groups
Selective reporting (reporting bias)	High risk	Outcomes for the exercise group were not fully reported to allow for meta-analysis

Caplan 2004

**Methods**	**Place of recruitment**: A&E department; solicited by in-person contact at A&E department or telephone the day after discharge.<br>**Inclusion criteria**: aged 75+, discharged from the A&E department.<br>Excluded if: lived outside the immediate area of a local hospital or in a nursing home.
**Participants**	**Number randomised**: 370 (Intervention) / 369 (Comparison)<br>**Mean age**: 82.3<br>**Sex (% female)**: 60.5%<br>**Mortality per year in the comparison group**: 9.6%<br>**Living alone (%):** 39.0%
**Interventions**	**Visitors' professional group**: Nurse<br>**Frequency and duration of visits**: 2.29 visits on average over 4 weeks<br>**Fall prevention:** No<br>**Included exercise**: No<br>**Included multidimensional geriatric assessment:** Yes<br>**Description of intervention:** Visits involved health assessment guided by clinical findings, formulation of care plan, urgent medical interventions, and referrals.<br>**Description of comparison**: Usual care<br>**Implementation (fidelity and participant compliance)**: Not reported.
**Outcomes**	**A&E Number:** Total number of visits to an A&E department (18 months)<br>**A&E People:** Total number of people admitted to an A&E department (1, 18 months)<br>**Hospitalisation Admissions:** Total number of hospital admissions (1 month)<br>**Institutionalisation People:** Total number of people admitted to nursing home (18 months)<br>**Mortality:** Total number of deaths (18 months)
**Notes**	**Location**: Sydney, Austrailia<br>**Funding Source**: National Demonstration Hospitals Program of the Commonwealth Department of Health and Aged Care<br>**Other notes**:

Risk of bias table

**Bias**	**Authors' judgement**	**Support for judgement**
Random sequence generation (selection bias)	Low risk	Quote: "patients were randomized using computer-generated random numbers"
Allocation concealment (selection bias)	Low risk	Quote: "patients were randomized using computer-generated random numbers coded into opaque envelopes"
Blinding (performance bias and detection bias)	High risk	Quote: "almost half conducted by a research assistant, with the rest performed by different members of the multidisciplinary team"
Incomplete outcome data (attrition bias)	Unclear risk	10.0% of intervention participants and 13.0% of control participants withdrew from the study. The authors did not report reasons for withdrawal.
Selective reporting (reporting bias)	High risk	Only data at 1 and 18 months were reported, yet measurements were taken at 1, 3, 6, 12 and 18 months

Chandler 1998

**Methods**	**Place of recruitment**: Outpatient medical clinics, aging registry, elderly housing complexes, home health agencies, Meals on Wheels programs, and private clinics; solicited by phone.<br>**Inclusion criteria**: aged 64+, unable to descend stairs without holding the railing. Excluded if: terminally ill, high level of fitness, severe, unstable cardiac disease, severe neurologic disease, complete blindness, lower extremity amputation,high cognitive impairment or lived in a resting home.
**Participants**	**Number randomised**: 50 (Intervention) / 50 (Comparison)<br>**Mean age**: 77.6<br>**Sex (% female)**: 50.0%<br>**Mortality per year in the comparison group**: Not reported<br>**Living alone (%):** Not reported
**Interventions**	**Visitors' professional group**: Physical therapist<br>**Frequency and duration of visits**: 30 visits over 10 weeks<br>**Fall prevention:** Yes<br>**Included exercise**: Yes<br>**Included multidimensional geriatric assessment:** No<br>**Description of intervention:** In-home program consisting of resistive lower extremity exercises. Visitors systematically increased resistance for each participant during the 10-week program.<br>**Description of comparison**: Wait-list control.<br>**Implementation (fidelity and participant compliance)**: 86.0% of intervention participants completed the intervention.
**Outcomes**	**No relevant outcome data reported**
**Notes**	**Location**: Durham, North Carolina, U.S.A.<br>**Funding Source**: Department of Veterans Affairs, Rehabilitation Research and Development Service, and the Center for the Study of Aging and Human Development, Claude Pepper Center, Duke University
	**Other notes**: Authors did not report post-intervention data on depression, 6-minute walk distance, falls efficacy scale, level of frailty, and SF36-Physical; no response from authors after attempted contact to provide this data.

Risk of bias table

**Bias**	**Authors' judgement**	**Support for judgement**
Random sequence generation (selection bias)	Unclear risk	Quote: "subjects were then block-randomized and stratified by the two levels of functioning"
Allocation concealment (selection bias)	Unclear risk	Quote: "subjects were then block-randomized and stratified by the two levels of functioning"
Blinding (performance bias and detection bias)	Low risk	Quote: "all baseline laboratory testing was performed by persons blinded to the subject's intervention status. The examiner who performed postintervention testing was, by protocol design, not familiar with participant and had no knowledge of the participant's baseline scores"
Incomplete outcome data (attrition bias)	Unclear risk	The study did not address this outcome, because the authors did not report any relevant outcome data
Selective reporting (reporting bias)	High risk	Multiple outcome measures described in the study were either not included in the results section or were reported incompletely to provide for meta-analysis

Ciaschini 2009

**Methods**	**Place of recruitment**: A&E registration database, fracture clinic registry, and local health care providers; solicited by clinician referrals and advertisements in hospitals and health centres.<br>**Inclusion criteria**: aged 55+, at risk of falls based on walking speed, previous fracture, or bone strength.
**Participants**	**Number randomised**: 101 (Intervention) / 100 (Comparison)<br>**Mean age**: 71.9<br>**Sex (% female)**: 94.0%<br>**Mortality per year in the comparison group**: 8.0%<br>**Living alone (%):** Not reported
**Interventions**	**Visitors' professional group**: Nurse<br>**Frequency and duration of visits**: Not reported<br>**Fall prevention:** Yes<br>**Included exercise**: Yes<br>**Included multidimensional geriatric assessment:** Yes<br>**Description of intervention:** Initial visit included medication review and assessments for functional risk for falls. Follow-up visits included medication review, referral to physio- and occupational therapy, provision of educational materials, and counselling on enactment of falls prevention plan.<br>**Description of comparison**: Usual care.<br>**Implementation (fidelity and participant compliance)**: 100% of intervention participants received the intervention. 21.8% of intervention participants and 6.0% of comparison participants were referred to physiotherapy at 6 months. 14.9% of intervention participants and 0.0% of control participants were referred to occupational therapy at 6 months.
**Outcomes**	**Falls People:** Total number of individuals reporting falls (6 months)<br>**Hospitalisation People:** Total number of people hospitalised (6 months)<br>**Injuries People:** Total number of people experiencing injury due to fall (6 months)<br>**Mortality:** Total number of deaths (6 months)
**Notes**	**Location**: Algoma District, Ontario, Canada<br>**Funding Source**: Merck Frost Canada Ltd, Sanofi-Aventis Pharmca Inc., Proctor and Gamble Pharmaceuticals Canada, Inc., Eli Lilly Canada Inc., and the Greenshield Foundation<br>**Other notes**:

Risk of bias table

**Bias**	**Authors' judgement**	**Support for judgement**
Random sequence generation (selection bias)	Low risk	Quote: "eligible patients were randomised, using a computer-generated randomisation scheme under the supervision of the study biostatistician"
Allocation concealment (selection bias)	Low risk	Quote: "eligible patients were randomised, using a computer-generated randomisation scheme under the supervision of the study biostatistician"
Blinding (performance bias and detection bias)	High risk	Quote: "the patients, treating physicians and outcomes collectors could not be blinded to the intervention status"
Incomplete outcome data (attrition bias)	Low risk	All randomized participants were included in the analysis
Selective reporting (reporting bias)	High risk	Quote:"This trial has been registered with clinicaltrials.gov (ID: NCT00465387)" Comment: Secondary outcomes that appear in the study were not specified in the protocol yet appear in the study, making it probable that the authors recorded other outcomes not reported.

Ciechanowski 2004

**Methods**	**Place of recruitment**: Senior service agencies and senior public housing; solicited by in-person visits to service agencies or housing units, phone calls, and mail.<br>**Inclusion criteria**: aged 60+, Diagnostic and Statistics Manual IV criteria for minor depression, receiving services from senior service agencies or living in senior service public housing.
**Participants**	**Number randomised**: 72 (Intervention) / 66 (Comparison)<br>**Mean age**: 73.0<br>**Sex (% female)**: 79.0%<br>**Mortality per year in the comparison group**: 3.0%<br>**Living alone (%):** 71.7%
**Interventions**	**Visitors' professional group**: Psychotherapist<br>**Frequency and duration of visits**: 6.6 vists on average over 19 weeks<br>**Fall prevention:** No<br>**Included exercise**: No<br>**Included multidimensional geriatric assessment:** No<br>**Description of intervention:** Visitors administered problem-solving treatment for depressive symptoms.<br>**Description of comparison**: Usual care, as well as letters sent to participants' physicians and social workers reporting their depression diagnosis with recommendations to continue usual care.<br>**Implementation (fidelity and participant compliance)**: Not reported.
**Outcomes**	**A&E People**: Total number of people who visited an A&E department (6 months)<br>**Hospitalisation People:** Total number of people hospitalised (6 months)<br>**Mortality:** Total number of deaths (6, 12 months)<br>**Psychiatric Anxiety and Depression:** Hopkins Symptom Checklist - Depression (6, 12 months)
**Notes**	**Location**: Seattle, Washington, U.S.A.<br>**Funding Source**: Prevention Research Centers Program of the Centers of Disease Control and Prevention, and University of Washington Health Promotion Research Centre<br>**Other notes**: Estimated cost of intervention was US$630 per participant

Risk of bias table

**Bias**	**Authors' judgement**	**Support for judgement**
Random sequence generation (selection bias)	Unclear risk	Quote: "using 50:50 randomized allocation ratio, investigators created envelopes containing concealed assignment codes assigned sequentially to eligible patients in blocks of 10 by a research associate"<br>Comment: No mention of how assignment codes were generated
Allocation concealment (selection bias)	Unclear risk	Quote: "using 50:50 randomized allocation ratio, investigators created envelopes containing concealed assignment codes assigned sequentially to eligible patients in blocks of 10 by a research associate"
Blinding (performance bias and detection bias)	Low risk	Quote: "outcome assessments were conducted in person at baseline and at 6 and 12 months by trained interviewers not involved in the intervention"
Incomplete outcome data (attrition bias)	Low risk	Missing outcome data was <10% and balanced in numbers between groups, with similar reasons for missing data between groups
Selective reporting (reporting bias)	Unclear risk	Study does not reference a trial protocol.

Close 1999

**Methods**	**Place of recruitment**: Computerised registration system of the A&E; solicited by letter and telephone.<br>**Inclusion criteria**: aged 65+, lived in local community, attended A&E for a fall. Excluded if: cognitively impaired (Abbreviated Mental Test < 7), had no regular carer, and did not speak English.
**Participants**	**Number randomised**: 184 (Intervention) / 213 (Comparison)<br>**Mean age**: 78.2<br>**Sex (% female)**: 67.5%<br>**Mortality per year in the comparison group**: 12.7%<br>**Living alone (%):** 61.2%
**Interventions**	**Visitors' professional group**: Occupational therapist<br>**Frequency and duration of visits**: 1 visit after hospital examination<br>**Fall prevention:** Yes<br>**Included exercise**: No<br>**Included multidimensional geriatric assessment:** Yes<br>**Description of intervention:** Physicians led a detailed medical assessment in hospital, followed by an occupational-therapy home-based assessment and education of functionality and environmental hazards. Referral to relevant services and home modifications, if indicated, were also provided.<br>**Description of comparison**: Usual care<br>**Implementation (fidelity and participant compliance)**: 152 (83%) medical assessments were undertaken; most were completed within 3 weeks of the index fall. Of the 32 patients who did not attend for assessment, 8 had died, 5 had moved into institutional care, and 19 gave no reason but were willing to complete the follow-up questionnaire. 140 occupational-therapy home assessment were undertaken in the intervention group; 12 patients who had undergone a medical assessment declined the home assessment. These assessments resulted in 67 referrals to hospital outpatient departments, 38 referrals to the day hospital, follow-up by the general practitioner for 33 patients, (16 for drug modification), and a visit to an optician for 27 cases. In only 24 (16%) of the assessments, no further action was required.
**Outcomes**	**Falls Number:** Total number of falls (12 months)<br>**Falls People:** Number of participants reporting falls (12 months)<br>**Functional ADL/IADL:** Barthel index (12 months)<br>**Hospital Admissions:** Total number of hospital admissions (12 months)<br>**Injuries People:** Number of participants reporting serious injuries from falls (12 months)<br>**Institutionalisation People:** Number of people moved to institutional care (12 months)<br>**Mortality:** Total number of deaths (12 months)
**Notes**	**Location**: England<br>**Funding Source**: South Thames NHS Research and Development Project Grant<br>**Other notes**:

Risk of bias table

Bias	Authors' judgement	Support for judgement
Random sequence generation (selection bias)	Low risk	Quote: “Randomisation was by a random-numbers table.”
Allocation concealment (selection bias)	Low risk	Quote: “the [random numbers] list was held independently of the investigators.”
Blinding (performance bias and detection bias)	High risk	Quote: “Follow-up was done by postal questionnaire, which was sent to all participants every 4 months for 1 year after the fall. Information about subsequent falls, fall-related injury, and details of doctor and hospital visits or admissions and degree of function were requested.”
Incomplete outcome data (attrition bias)	Unclear risk	Quote: “The analyses were by intention to treat.“
Selective reporting (reporting bias)	Unclear risk	Study does not reference a trial protocol.

Counsell 2007

**Methods**	**Place of recruitment**: Community health centres; method of solicitation not reported.<br>**Inclusion criteria**: aged 65+, establishd patient at health centre, income below 200% of the federal poverty level.
**Participants**	**Number randomised**: 474 (Intervention) / 477 (Comparison)<br>**Mean age**: 71.7<br>**Sex (% female)**: 75.0%<br>**Mortality per year in the comparison group**: 3.9%<br>**Living alone (%):** 46.8%
**Interventions**	**Visitors' professional group**: Nurse, social worker<br>**Frequency and duration of visits**: 13 visits over 24 months<br>**Fall prevention:** No<br>**Included exercise**: No<br>**Included multidimensional geriatric assessment:** Yes<br>**Description of intervention:** Visitors provided comprehensive geriatric assessment with development of individualised care plan.<br>**Description of comparison**: Usual care.<br>**Implementation (fidelity and participant compliance)**: Not reported
**Outcomes**	**A&E Number:** Number of visits to an A&E department per person (12, 24 months)<br>**Functioning ADL/IADL:** Assets and Health Dynamics of the Oldest-Old Survey (24 months); Instrumental Activities of Daily Living Items (24 months)<br>**Health Related QoL:** Medical Outcomes Study Short Form Health Survey-36 items for (1) Mental Health (24 months) and (2) Physical Health (24 months)<br>**Hospitalisation Admissions:** Total number of hospital admissions (12, 24 months)<br>**Hospitalisation Days:** Number of days in hospital per person (12, 24 months)<br>**Instituionalisation People:** Total number of people admitted to nursing homes (24 months)<br>**Mortality:** Total number of deaths (6, 12, 18, 24 months)
**Notes**	**Location**: Indianapolis, Indiana, U.S.A.<br>**Funding Source**: National Institute on Ageing, National Institutes of Health<br>**Other notes**: Estimated cost of intervention was US$1000 per participant

Risk of bias table

**Bias**	**Authors' judgement**	**Support for judgement**
Random sequence generation (selection bias)	Low risk	Quote: "randomization lists were generated by the study biostatistician (T.E.S.) with the aid of [a] pseudorandom-number generator"
Allocation concealment (selection bias)	Low risk	Quote: "randomization lists were generated by the study biostatistician (T.E.S.) with the aid of [a] pseudorandom-number generator"
Blinding (performance bias and detection bias)	Low risk	Quote: "interviewers ... were blinded to the patient’s randomization status and ... were not part of the recruitment or intervention process"
Incomplete outcome data (attrition bias)	Low risk	Data was missing for only 3.2% in the intervention group and 3.7% in the control group. Authors used two different imputation methods (last observation carried forward and multiple regression) that provided similar results
Selective reporting (reporting bias)	Low risk	Registration at clinicaltrials.gov. Identifier number is NCT00182962

Crawford Shearer 2010

**Methods**	**Place of recruitment**: Participants' homes; solicited by flyer distribution and in-person contact during meal delivery service.<br>**Inclusion criteria**: aged 60+, low cognitive impairment, proficiency in English, received home-delivered meals through local community action agency.
**Participants**	**Number randomised**: 32 (Intervention) / 27 (Comparison)<br>**Mean age**: 77.8<br>**Sex (% female)**: 71.2%<br>**Mortality per year in the comparison group**: 25.0%<br>**Living alone (%):** Not reported
**Interventions**	**Visitors' professional group**: Nurse<br>**Frequency and duration of visits**: 6 visits over 12 weeks<br>**Fall prevention:** No<br>**Included exercise**: No<br>**Included multidimensional geriatric assessment:** No<br>**Description of intervention:** Visits involved establishment of health goals, as well as support and guidance in attaining health goals and accessing local resources.<br>**Description of comparison**: Attention-matched control (received health and safety newsletters).<br>**Implementation (fidelity and participant compliance)**: 56.0% of intervention participants completed all six sessions.
**Outcomes**	**Health Related QoL:** Well-Being Picture Scale (3 months); Ryff Psychological Scale (3 months)<br>**Mortality:** Total number of deaths (3 months)
**Notes**	**Location**: Southwest metropolitan area, U.S.A.<br>**Funding Source**: National Institutes of Health, National Institute of Nursing Research<br>**Other notes**:

Risk of bias table

**Bias**	**Authors' judgement**	**Support for judgement**
Random sequence generation (selection bias)	Low risk	Quote: "random assignment ... was accomplished using SAS software"
Allocation concealment (selection bias)	Low risk	Quote: "random assignment to the intervention group or attentional comparison group was accomplished using SAS software"
Blinding (performance bias and detection bias)	Low risk	Quote: "the people conducting the measures were blinded to the group assignment"
Incomplete outcome data (attrition bias)	High risk	Quote: "the reasons for attrition varied across groups"<br>37.5% of intervention participants and 18.5% of control participants were lost to follow-up
Selective reporting (reporting bias)	Unclear risk	Study does not reference a trial protocol

Dalby 2000

**Methods**	**Place of recruitment**: Primary care practice; solicited by mail.<br>**Inclusion criteria**: aged 70+, reported functional impairment, hospital admission in past 6 months.<br>Excluded if: involved in another research study, had previously been visited by nurse in home.
**Participants**	**Number randomised**: 73 (Intervention) / 69 (Comparison)<br>**Mean age**: 78.6<br>**Sex (% female)**: 66.9%<br>**Mortality per year in the comparison group**: 3.7%<br>**Living alone (%):** 39.4%
**Interventions**	**Visitors' professional group**: Nurse<br>**Frequency and duration of visits**: 3 visits over 14 months<br>**Fall prevention:** No<br>**Included exercise**: No<br>**Included multidimensional geriatric assessment:** Yes<br>**Description of intervention:** Visitors reviewed medical records, medication, and safety and suitability of participants' home environment. Visitors also completed assessment of physical, cognitive, emotional, and social functioning, and they developed care plans with primary care physician, patient, family, caregivers, and other healthcare professionals.<br>**Description of comparison**: Usual care<br>**Implementation (fidelity and participant compliance)**: Not reported
**Outcomes**	**A&E Number:** Total number of visits to an A&E department (14 months)<br>**Hospitalisations Admissions:** Total number of hospital admissions (14 months)<br>**Hospitalisation Days:** Total number of days in hospital (14 months)<br>**Institutionalisation People:** Total number of people admitted to nursing homes (14 months)<br>**Mortality:** Total number of deaths (14 months)
**Notes**	**Location**: Stoney Creek, Ontario, Canada<br>**Funding Source**: Ontario Ministry of Health, Community Health Branch<br>**Other notes**:

Risk of bias table

Bias	Authors' judgement	Support for judgement
Random sequence generation (selection bias)	Low risk	Quote: "random numbers table"
Allocation concealment (selection bias)	Low risk	Quote: "the randomization schedule was developed by another research assistant, who was not involved in the randomization [i.e., allocation] process"
Blinding (performance bias and detection bias)	Low risk	Quote: "the research nurse involved in reviewing the medical records was blinded to group allocation"
Incomplete outcome data (attrition bias)	Low risk	Outcome data were available for 95.9% of intervention participants and 100% of control participants
Selective reporting (reporting bias)	Unclear risk	Study does not reference a trial protocol

Davison 2005

**Methods**	**Place of recruitment**: A&E department in a university teaching hospital; solicited by postal questionnaire and telephone.<br>**Inclusion criteria**: aged 65+, presented to A&E with a fall or fall-related injury, sustained at least one additional fall in the preceding year.<br>Excluded if: cognitively impaired, had at least 1 previous episode of fainting, immobile, lived more than 15 miles from hospital, registered blind, aphasic, had a clear medical explanation for their fall (e.g., stroke), enrolled in another study.
**Participants**	**Number randomised**: 159 (Intervention) / 154 (Comparison)<br>**Mean age**: 77.0<br>**Sex (% female)**: 72.2%<br>**Mortality per year in the comparison group**: 3.2%<br>**Living alone (%):** Not reported
**Interventions**	**Visitors' professional group**: Occupational therapist, physiotherapist<br>**Frequency and duration of visits**: Not reported<br>**Fall prevention:** Yes<br>**Included exercise**: Yes<br>**Included multidimensional geriatric assessment:** Yes<br>**Description of intervention:** Programme began with a hospital-based medical assessment of fall history and full medical examination. Visits involved home-based physiotherapy and a occupational therapy assessment of home environmental hazards, followed by a prioritised individualised intervention for fall risk factors.<br>**Description of comparison**: Usual care<br>**Implementation (fidelity and participant compliance)**: 91.8% of intervention participants underwent a full risk factor assessment. Intervention participants attended hospital a median of 2 occasions (range 0–10) for stabilisation of risk factors, received a median of 2 physiotherapy intervention visits (range 0–16), and 1 occupational therapy visit (range 0–4) with a median follow-up time of 32 days (range 0–143). 21.4% of the comparison participants received some form of specialist falls intervention during follow-up.
**Outcomes**	**A&E People:** Total number of people admitted to an A&E department due to fall (12 months)<br>**Falls Number:** Total number of falls (12 months)<br>**Falls People:** Total number of people who experienced a fall (12 months)<br>**Falls Subjective:** Activities-specific Balance Confidence Scale (12 months)<br>**Functioning Cognitive:** Mini-Mental State Examination (12 months)<br>**Hospitalisation Days:** Total number of days in hospital (12 months)<br>**Hospitalisation People:** Total number of people admitted to hospital due to fall (12 months)<br>**Injuries People:**<br>Total number of people who experienced a fracture (12 months)<br>**Mortality:** Total number of deaths (12 months)
**Notes**	**Location**: Newcastle upon Tyne, North Tyneside, and Gateshead; U.K.<br>**Funding Source**: Wellcome Trust and Northern, and Yorkshire NHS Executive<br>**Other notes**:

Risk of bias table

Bias	Authors' judgement	Support for judgement
Random sequence generation (selection bias)	Low risk	Quote: "randomisation was by computer-generated block randomisation"
Allocation concealment (selection bias)	Low risk	Quote: "randomisation was by computer-generated block randomisation"
Blinding (performance bias and detection bias)	Low risk	Quote: "interviewer was blind to randomisation"<br>Quote: "data were processed by a researcher blinded to randomisation and otherwise unconnected with the study"
Incomplete outcome data (attrition bias)	Low risk	11.3% of intervention participants and 8.4% of control participants were lost to follow-up. Reasons for missing data were similar between groups
Selective reporting (reporting bias)	High risk	Outcome data were measured at 3, 6, 9 and 12 months but only reported for 12 months

Elley 2008

**Methods**	**Place of recruitment**: Primary care practices; solicited by mail.<br>**Inclusion criteria**: aged 75+ (or aged 55+ if Maori), had fallen in the previous 12 months.<br>Excluded if: unable to understand study information and consent processes, unstable or progressive medical condition, severe physical disability, dementia.
**Participants**	**Number randomised**: 155 (Intervention) / 157 (Comparison)<br>**Mean age**: 80.8<br>**Sex (% female)**: 68.9%<br>**Mortality per year in the comparison group**: 2.6%<br>**Living alone (%):** Not reported
**Interventions**	**Visitors' professional group**: Nurse<br>**Frequency and duration of visits**: Frequency not reported, duration of 12 months<br>**Fall prevention:** Yes<br>**Included exercise**: Yes<br>**Included multidimensional geriatric assessment:** No<br>**Description of intervention:** Visits involved health and falls risk assessment. Visitors referral of participants to physicians, optomotrists, podiatrists, and other health professionals.<br>**Description of comparison**: Usual care from general practitioner. Also offer at least two social visits and a written falls prevention pamphlet.<br>**Implementation (fidelity and participant compliance)**: 24.4% of referred intervention participants reported undertaking three exercise sessions per week 10 months after the programme concluded.
**Outcomes**	**Falls Number:** Total number of falls (12 months)<br>**Falls People:**<br>Total number of people who experienced at least 1 fall (12 months)<br>**Injuries Number:** Total number of moderate injurious falls (12 months)<br>**Mortality:** Total number of deaths (12 months)
**Notes**	**Location**: Hutt Valley, New Zealand<br>**Funding Source**: Accident Compensation Corporation, the Hutt Valley District Health Board, the Lotteries Commission, and the Wellington Medical Research Foundation<br>**Other notes**:

Risk of bias table

Bias	Authors' judgement	Support for judgement
Random sequence generation (selection bias)	Low risk	Quote: "computer-based schedule generated by a statistician"
Allocation concealment (selection bias)	Low risk	Quote: "an independent researcher at a distant site randomly allocated participants to the intervention or control group using a computer-based schedule generated by a statistician"
Blinding (performance bias and detection bias)	Low risk	Quote: "the research nurses who recorded the demographic, clinical, and outcome measures at baseline and after 1 year, remained blind to group allocation"
Incomplete outcome data (attrition bias)	Low risk	12.9% of intervention participants and 7.6% of control participants were lost to follow-up. Reasons for missing data were similar between groups
Selective reporting (reporting bias)	High risk	Quote: "The trial was registered with the Australia New Zealand Clinical Trials Register (ID number: 12605000054617)"<br>Comment: Several outcomes of interest were reported incompletely so that they cannot be entered in a meta-analysis (i.e., Falls Subjective, Functioning ADL/IADL, Health Related QoL, Institutionalisation People)

Fabacher 1994

**Methods**	**Place of recruitment**: Directories of veterans groups and voter registries; solicited by mail.<br>**Inclusion criteria**: aged 70+, veteran of US armed services, not enrolled in Veterans Affairs outpatient clinic.<br>Excluded if: suffering from dementia or known terminal disease.
**Participants**	**Number randomised**: 131 (Intervention) / 123 (Comparison)<br>**Mean age**: 72.7<br>**Sex (% female)**: 2.3%<br>**Mortality per year in the comparison group**: 3.3%<br>**Living alone (%):** 20.1%
**Interventions**	**Visitors' professional group**: Physician’s assistant or nurse, volunteer home visitors<br>**Frequency and duration of visits**: 4 visits over 12 months<br>**Fall prevention:** Yes<br>**Included exercise**: No<br>**Included multidimensional geriatric assessment:** Yes<br>**Description of intervention:** Visits involved home safety inspection, health education, physical and mental health assessments, medication review, and other geriatric screenings. Follow-ups included sharing of assessment results with physician, a letter detailing recommendations for each participant, and additional visits to assist with compliance with recommendations.<br>**Description of comparison**: Usual care, with follow-up phone interviews.<br>**Implementation (fidelity and participant compliance)**: Staff members accompanied volunteers on 2+ visits to ensure fidelity. 53.0% of intervention participants complied with all recommendations, 42.0% complied with at least one (but not all), and 5.0% complied with none.
**Outcomes**	**Falls People:** Total number of people who experienced a fall (12 months)<br>**Functioning ADL/IADL:** Activities of Daily Living (12 months); Instrumental Activities of Daily Living (12 months)<br>**Hospitalisation People:** Total number of people hospitalised (12 months)<br>**Institutionalisation Days:** Total number of days in a nursing home (12 months)<br>**Institutionalisation People:** Total number of people admitted to nursing home (12 months)<br>**Mortality:** Total number of deaths (12 months)
**Notes**	**Location**: San Fernando Valley, Los Angeles, U.S.A.<br>**Funding Source**: Disabled American Veterans Charities of Greater Los Angeles and the Disabled American Veterans California Rehabilitation Foundation, Inc.<br>**Other notes**:

Risk of bias table

**Bias**	**Authors' judgement**	**Support for judgement**
Random sequence generation (selection bias)	Low risk	Quote: "randomly generated assignment cards in sealed envelopes"
Allocation concealment (selection bias)	Low risk	Quote: "randomly generated assignment cards in sealed envelopes"
Blinding (performance bias and detection bias)	High risk	Quote: "intervention subjects were also asked whether they complied with each HAPSA recommendation"<br>Comment: The protocol for outcome assessment differed by trial arm, indicating that assessors knew each participant's allocated intervention
Incomplete outcome data (attrition bias)	High risk	23.7% of intervention participants and 22.8% of control participants were lost to follow-up, and reasons for missing data differed by group.
Selective reporting (reporting bias)	Unclear risk	Study does not reference a trial protocol

Gallagher 1996

**Methods**	**Place of recruitment**: Capital Regional District of Victoria, British Columbia; solicited by community advertising campaign.<br>**Inclusion criteria**: aged 60+, sustained a fall within previous 6 months, lived in Capital Regional District.
**Participants**	**Number randomised**: 50 (Intervention) / 50 (Comparison)<br>**Mean age**: 74.6<br>**Sex (% female)**: 80.0%<br>**Mortality per year in the comparison group**: 0.0%<br>**Living alone (%):** Not reported
**Interventions**	**Visitors' professional group**: Nurse<br>**Frequency and duration of visits**: 3 visits over 6 months<br>**Fall prevention:** Yes<br>**Included exercise**: No<br>**Included multidimensional geriatric assessment:** No<br>**Description of intervention:** Visits involved comprehensive personal and home risk profile, comprehensive falls assessment risk, counselling session, booklet, and video.<br>**Description of comparison**: Not reported.<br>**Implementation (fidelity and participant compliance)**: Not reported.
**Outcomes**	**Falls Number:** Number of falls per person (6 months)<br>**Falls Subjective:** Fear of falling (6 months); Falls self-efficacy (6 months)<br>**Health Related QoL:** Medical Outcomes Study Short Form Health Survey-36 (6 months)<br>**Mortality:** Total number of deaths (6 months)
**Notes**	**Location**: Victoria, British Columbia, Canada<br>**Funding Source**: Not reported<br>**Other notes**:

Risk of bias table

**Bias**	**Authors' judgement**	**Support for judgement**
Random sequence generation (selection bias)	Unclear risk	Quote: "randomly assigned"
Allocation concealment (selection bias)	Unclear risk	Quote: "randomly assigned"
Blinding (performance bias and detection bias)	High risk	Quote: "the interviewer was not blind as to group assignment at the time of the six-month follow-up interview"
Incomplete outcome data (attrition bias)	Low risk	Quote: "no one dropped out of the study.... all participants received baseline and six-month interviews to measure the key study variables"
Selective reporting (reporting bias)	Unclear risk	Study does not reference a trial protocol

Gitlin 2006

**Methods**	**Place of recruitment**: Local social service agencies and an area agency on aging; solicited by in-person contact at agencies and media announcements in the Philadelphia region.<br>**Inclusion criteria**: aged 70+, low cognitive impairment, receiving home or occupational therapy, functionally vulnerable, ambulatory, English speaking
**Participants**	**Number randomised**: 160 (Intervention) / 159 (Comparison)<br>**Mean age**: 79.0<br>**Sex (% female)**: 81.8%<br>**Mortality per year in the comparison group**: 6.6%<br>**Living alone (%):** 61.8%
**Interventions**	**Visitors' professional group**: Occupational therapist, physical therapist<br>**Frequency and duration of visits**: 6 visits over 12 months<br>**Fall prevention:** Yes<br>**Included exercise**: Yes<br>**Included multidimensional geriatric assessment:** Yes<br>**Description of intervention:** Visits involved environmental modification, behavioural and cognitive strategies, and exercises for fall prevention led by physical therapist. Follow-ups involved phone calls to reinforce strategy.<br>**Description of comparison**: Usual care<br>**Implementation (fidelity and participant compliance)**: Not reported
**Outcomes**	**Falls Subjective:** Falls self-efficacy (6 months); Overall functional efficacy (6 months)<br>**Functioning ADL/IADL:** Activities of Daily Living (6 months); Mobility items (6 months); Instrumental Activities of Daily Living (6 months)<br>**Mortality:** Total number of deaths (12, 24, 48 months)
**Notes**	**Location**: Philadelphia area, Pennsylvania, U.S.A.<br>**Funding Source**: National Institute on Aging<br>**Other notes**: Estimated cost of intervention was US$1222 per participant

Risk of bias table

**Bias**	**Authors' judgement**	**Support for judgement**
Random sequence generation (selection bias)	Low risk	Quote: "study participants were ... randomized within each of four strata using random permuted blocks to control for possible changes in subject mix over time. The blocking number [was] developed by the project statistician"
Allocation concealment (selection bias)	Low risk	Quote: "randomization lists and four sets of randomization were prepared using double, opaque envelope"
Blinding (performance bias and detection bias)	Low risk	Quote: "trained interviewers ... were masked to group assignment and study hypotheses and ... had no role in the intervention"
Incomplete outcome data (attrition bias)	Unclear risk	6.9% of intervention participants and 14.5% of control participants were lost at follow-up. Reasons for missing data and intention-to-treat principle were not reported by group
Selective reporting (reporting bias)	Low risk	"The only censoring was administrative; all participants' follow-up was administratively censored at December 31, 2005, corresponding to the last date for which NDI information was available"

Green 2002

**Methods**	**Place of recruitment**: Hospital and community therapy stroke registers; method of solicitation not reported<br>**Inclusion criteria**: aged 50+, had a stroke at least 1 year previously and associated mobility problems<br>Excluded if: "non-stroke caused" mobility problems, dementia, severe comorbidity, bedfast, undergone physiotherapy treatment within past 6 months
**Participants**	**Number randomised**: 85 (Intervention) / 85 (Comparison)<br>**Mean age**: 72.5<br>**Sex (% female)**: 44.0%<br>**Mortality per year in the comparison group**: 7.8%<br>**Living alone (%):** 27.5%
**Interventions**	**Visitors' professional group**: Physical therapist<br>**Frequency and duration of visits**: 3 visits on average over 3 months<br>**Fall prevention:** Yes<br>**Included exercise**: No<br>**Included multidimensional geriatric assessment:** No<br>**Description of intervention:** Visits involved physiological assessment followed by a physiotherapy problem solving approach.<br>**Description of comparison**: Usual care.<br>**Implementation (fidelity and participant compliance)**: The median number of treatments per intervention participant was 3 (inter-quartile range: 2–7, range: 0–22).<br>The mean duration of every treatment for intervention participants was 44 min (*SD*: 21, range: 10–90).
**Outcomes**	**Falls People:** Total number of people who experienced a fall (3, 9 months)<br>**Mortality:** Total number of deaths (9 months)
**Notes**	**Location**: U.K.<br>**Funding Source**: Stroke Association, Hospital Savings Association<br>**Other notes**:

Risk of bias table

Bias	Authors' judgement	Support for judgement
Random sequence generation (selection bias)	Low risk	Quote: "prepared from random number tables and used four length random permuted blocks"
Allocation concealment (selection bias)	Low risk	Quote: "numbered, sealed, opaque envelopes"<br>Quote: "patients were assigned ... by an assistant, who was otherwise unconnected with the study"
Blinding (performance bias and detection bias)	Low risk	Quote: "all assessments were done in patients' homes by one researcher who did not know treatment allocation"
Incomplete outcome data (attrition bias)	Low risk	15.3% of intervention participants and 12.9% of control participants were lost at follow-up. Reasons for follow-up were similar between groups
Selective reporting (reporting bias)	Unclear risk	Study does not reference a trial protocol

Gunner-Svensson 1984

**Methods**	**Place of recruitment**: national registry; method of solicitation not reported<br>**Inclusion criteria**: aged 70+, not currently living in nursing home
**Participants**	**Number randomised**: 1844 (Intervention) / 1899 (Comparison)<br>**Mean age**: Not reported<br>**Sex (% female)**: 58.5%<br>**Mortality per year in the comparison group**: 4.7%<br>**Living alone (%):** Not reported
**Interventions**	**Visitors' professional group**: Nurse<br>**Frequency and duration of visits**: Varied over 5 years<br>**Fall prevention:** No<br>**Included exercise**: No<br>**Included multidimensional geriatric assessment:** Yes<br>**Description of intervention:** Social medical intervention that involved introduction to services, advice to the aged and associated personnel, coordination and follow-up for assessment of results.<br>**Description of comparison**: Usual care.<br>**Implementation (fidelity and participant compliance)**: Did not report.
**Outcomes**	**Institutionalisation, People**: Total number of people admitted to nursing homes (12, 28 and 60 months)<br>**Mortality**: Total number of deaths (60 months)
**Notes**	**Location**:Odense, Denmark<br>**Funding Source**: Odense's third magistral department. Sygekassernes Helsefond (No. 9665) and Statens samfundsvidenskabeligeforskningsrgd (J.nr. 514-10161<br>**Other notes**:

Risk of bias table

**Bias**	**Authors' judgement**	**Support for judgement**
Random sequence generation (selection bias)	High risk	Quote: "The material used is a random sample taken from the national person register systematically stratified for age. . . The sample was then randomized into an experimental group and a control group by consecutively and alternately dividing it into the two groups"
Allocation concealment (selection bias)	High risk	Quote: "The material used is a random sample taken from the national person register systematically stratified for age. . . The sample was then randomized into an experimental group and a control group by consecutively and alternately dividing it into the two groups"
Blinding (performance bias and detection bias)	Unclear risk	Quote: "The work was done by four to five nurses with the assistance of one full-time and two part-time secretaries."
Incomplete outcome data (attrition bias)	Unclear risk	Comment: The authors did not reported attrition or the statistical handling of it post-intervention.
Selective reporting (reporting bias)	Unclear risk	Comment: The authors did not register a trial protcol.

Gustafsson 2012

**Methods**	**Place of recruitment**: Official registers from two urban districts; solicitation method not reported.<br>**Inclusion criteria**: Aged 80+, community dwelling, not be dependent on municipal home help service or care, be independent from another person in ADLs, frail, and be cognitively intact (MMSE ≥ 25).
**Participants**	**Number randomised**: 174 (Intervention) / 114 (Comparison)<br>**Mean age**: 86.0<br>**Sex (% female)**: 62.8%<br>**Mortality per year in the comparison group**: 0%<br>**Living alone (%):** 53.5%
**Interventions**	**Visitors' professional group**: Occupational therapist, physiotherapist, registered nurse, or social worker<br>**Frequency and duration of visits**: 1 visit lasting 1.5-2 hours<br>**Fall prevention:** Yes<br>**Included exercise**: Yes<br>**Included multidimensional geriatric assessment:** No<br>**Description of intervention:** Educational information about relevant services in the community for senior citizens, identification of and advice for environmental fall risk factors in the home, and exercise advice and instruction.<br>**Description of comparison**: Usual care<br>**Implementation (fidelity and participant compliance)**: All participants assigned to a preventive home visit received the intervention. No adverse events were reported, and no known organized co-intervention took place during the period in question.
**Outcomes**	**Functional ADL/IADL:** The ADL Staircase (3 months)<br>**Health-Related QoL:** Self-rated health (3 months)<br>**Mortality:** Total number of deaths (3 months)
**Notes**	**Location**: Gothenburg, Sweden<br>**Funding Source**: Vardalinstitutet, the Swedish Institute for Health Sciences, the Research and Development Council of the County of Sodra Alvsborg, and the SwedBank Sjuharads Foundation for Research at the Hospital of Sodra Alvsborg.<br>**Other notes**:

Risk of bias table

**Bias**	**Authors' judgement**	**Support for judgement**
Random sequence generation (selection bias)	Low risk	See allocation concealment.
Allocation concealment (selection bias)	Low risk	Quote: "An independent researcher, not involved in the enrolling of participants or in the interventions, organized the allocation system used. A research assistant consecutively and randomly assigned the study participants to one of the three study arms using opaque sealed envelopes."
Blinding (performance bias and detection bias)	Low risk	Quote: "Those assessing the outcomes were blind to group assignment."
Incomplete outcome data (attrition bias)	High risk	Quote: Tthe imputation method chosen was to replace missing values with a value based on the median change of deterioration (MCD) between baseline and follow-up of all who participated at follow-up. Consequently, the MCD for an outcome measure was added to the individual value registered at baseline and imputed, substituting missing data at follow-up."
Selective reporting (reporting bias)	High risk	Several outcomes in study protocol are not reported in this paper (ClinicalTrials.gov NCT00877058), such as falls, fall self-efficacy, and functioning cognitive. Additionally, continuous outcomes (Health-Related QoL and ADL/IADL) only reported as number of people who did not deteriorate.

Hall 1992

**Methods**	**Place of recruitment**: At-home long term care programme; method of solicitation not reported<br>**Inclusion criteria**: aged 65+, recommended for personal care
**Participants**	**Number randomised**: 81 (Intervention) / 86 (Comparison)<br>**Mean age**: 78.0<br>**Sex (% female)**: 78.4%<br>**Mortality per year in the comparison group**: 7.0%<br>**Living alone (%):** 75.5%
**Interventions**	**Visitors' professional group**: Nurse<br>**Frequency and duration of visits**: Varied frequency over 36 months<br>**Fall prevention:** No<br>**Included exercise**: Yes<br>**Included multidimensional geriatric assessment:** Yes<br>**Description of intervention:** Visits involved development of personal health plan, with nurse support, reinforcement and referrals.<br>**Description of comparison**: Usual care.<br>**Implementation (fidelity and participant compliance)**: Not reported.
**Outcomes**	**Institutionalisation People:** Total number of people admitted to nursing home (12, 24, 36 months)<br>**Mortality:** Total number of deaths (12, 24, 36 months)
**Notes**	**Location**: New Westminster, British Columbia, Canada<br>**Funding Source**: British Columbia Health Research Foundation<br>**Other notes**:

Risk of bias table

**Bias**	**Authors' judgement**	**Support for judgement**
Random sequence generation (selection bias)	Low risk	Quote: "randomisation was done in blocks of four, using a table of random numbers"
Allocation concealment (selection bias)	Unclear risk	Quote: "clients who consented to participate in the study were randomly assigned"
Blinding (performance bias and detection bias)	Low risk	Quote: "independent assessor blinded with respect to subjects' group assignment"
Incomplete outcome data (attrition bias)	Low risk	Results included data from all randomised participants who had not died
Selective reporting (reporting bias)	High risk	Physical health, nutritional status, functional status, and socialisation were measured but not reported

Hebert 2001

**Methods**	**Place of recruitment**: List of patients in Quebec Health Insurance Plan; solicited by mail<br>**Inclusion criteria**: aged 75+, at risk of functional decline
**Participants**	**Number randomised**: 250 (Intervention) / 253 (Comparison)<br>**Mean age**: 80.3<br>**Sex (% female)**: 64.2%<br>**Mortality per year in the comparison group**: 7.1%<br>**Living alone (%):** Not reported
**Interventions**	**Visitors' professional group**: Nurse<br>**Frequency and duration of visits**: 1 visit over 12 months<br>**Fall prevention:** No<br>**Included exercise**: No<br>**Included multidimensional geriatric assessment:** Yes<br>**Description of intervention:** Visits involved medical and psychiatric evaluation, recommendations to general practitioner, and direct referrals to specialists<br>**Description of comparison**: Usual care<br>**Implementation (fidelity and participant compliance)**: 66.8% of intervention participants complied with recommendations.
**Outcomes**	**Functioning ADL/IADL:** Functional Autonomy Measurement System (SMAF) (12 months)<br>**Health Related QoL:** Dupuy General Well-Being (12 months)<br>**Institutionalisation People:** Total number of people admitted to nursing home (12 months)<br>**Mortality:** Total number of deaths (12 months)
**Notes**	**Location**: Sherbrooke City, Quebec, Canada<br>**Funding Source**: Quebec Health Research Fund<br>**Other notes**:

Risk of bias table

**Bias**	**Authors' judgement**	**Support for judgement**
Random sequence generation (selection bias)	Unclear risk	Quote: "the randomization lists were generated with random permutated blocks of 4-6"
Allocation concealment (selection bias)	Unclear risk	Quote: "randomized either to the study group ... or the control group"
Blinding (performance bias and detection bias)	Low risk	Quote: "interviewers were blinded to the assignment of the subjects"
Incomplete outcome data (attrition bias)	Low risk	6.8% of intervention participants and 8.7% of control participants withdrew from study. Reasons for missing data were similar between groups.
Selective reporting (reporting bias)	Unclear risk	Study does not reference a trial protocol

Hendriks 2008

**Methods**	**Place of recruitment**: A&E and General Practice Cooperative that provided after-hours emergency care; solicited by in-person contact when presenting for fall.<br>**Inclusion criteria**: aged 65+.<br>Excluded if: non-Dutch speaking, cognitively impaired, had been admitted for more than 4 weeks to a hospital or another institution, permanently wheelchair-dependent or bedridden.
**Participants**	**Number randomised**: 166 (Intervention) / 167 (Comparison)<br>**Mean age**: 74.9<br>**Sex (% female)**: 68.5%<br>**Mortality per year in the comparison group**: 0.6%<br>**Living alone (%):** Not Reported
**Interventions**	**Visitors' professional group**: Medical professionals (geriatricians, GPs, rehabilitation physicians), geriatric nurses, occupational therapists<br>**Frequency and duration of visits**: 1 visit over 1 month<br>**Fall prevention:** Yes<br>**Included exercise**: No<br>**Included multidimensional geriatric assessment:** Yes<br>**Description of intervention**: Programme began with hospital-based medical assessment comprising a comprehensive general examination of potential risk factors for new falls. An occupational therapist then visited the participants at home for a structured functional and environmental assessment. Technical aids and adaptations or additional support were directly referred to and delivered by social and community services.<br>**Description of comparison**: Usual care<br>**Implementation (fidelity and participant compliance)**: Not reported
**Outcomes**	**Falls People:** Total number of people who experienced a fall (4, 12 months)<br>**Falls Subjective:** Fear of falling (4, 12 months)<br>**Functioning ADL/IADL:** Frenchay Activity Index (4, 12 months); Groningen Activity Restriction Scale (4, 12 months)<br>**Health Related QoL:** Euroqol perceived health (4, 12 months); Medical Outcomes Study Short Form Health Survey-36 items for perceived health (4, 12 months)<br>**Injuries People:** Total number of people experiencing injury due to fall (4, 12 months)<br>**Mortality:** Total number of deaths (12 months)<br>**Psychiatric Anxiety and Depression:** Hospital Anxiety and Depression Scale (12 months)
**Notes**	**Location**: University Hospital Maastricht, Netherlands<br>**Funding Source**: Netherlands Organization for Health Research and Development Grant 945-02-053<br>**Other notes**:

Risk of bias table

Bias	Authors' judgement	Support for judgement
Random sequence generation (selection bias)	Low risk	Quote: "computerized alternate random allocation"
Allocation concealment (selection bias)	Low risk	Quote: "an external agency allocated eligible participants who signed the informed consent form and returned a completed baseline questionnaire"
Blinding (performance bias and detection bias)	Low risk	Quote: "to ensure blinding during data collection, measurements by telephone were contracted to an independent call center (Centre for Data and Information Management, MEMIC), whose operators were unaware of group allocation"
Incomplete outcome data (attrition bias)	High risk	25.3% of intervention participants and 19.8% of control participants were lost at follow-up. Reasons for missing data differed between groups
Selective reporting (reporting bias)	Unclear risk	Study does not reference a trial protocol

Hogan 2001

**Methods**	**Place of recruitment**: location of recruitment not reported; method of solicitation not reported<br>**Inclusion criteria**: aged 65+, competent to give consent, fallen within the previous 3 months, ambulatory<br>Excluded if: fall occurred (1) during high risk activities, (2) while in a treatment hospital, (3) as a result of a syncope or acute stroke, (4) and resulted in lower extremity fracture.
**Participants**	**Number randomised**: 79 (Intervention) / 84 (Comparison)<br>**Mean age**: 77.7<br>**Sex (% female)**: 71.8%<br>**Mortality per year in the comparison group**: 6.0%<br>**Living alone (%):** Not reported
**Interventions**	**Visitors' professional group**: Nurse, occupational therapist, physical therapist, or geriatric medicine specialist<br>**Frequency and duration of visits**: 2 visits over 12 months<br>**Fall prevention:** Yes<br>**Included exercise**: Yes<br>**Included multidimensional geriatric assessment:** No<br>**Description of intervention:** Visits included evaluation of participant and environmental risk, design of plan to reduce falls risk, recommendations for enacting plan and referrals, and instruction for a home exercise program.<br>**Description of comparison**: Attention-matched control<br>**Implementation (fidelity and participant compliance)**: Intervention participants adhered to 81.1% of recommendations.
**Outcomes**	**A&E People**: Total number of people admitted to an A&E department due to fall (12 months)<br>**Falls Number:** Number of falls per person (12 months)<br>**Falls People:** Total number of people who experienced a fall (12 months)<br>**Hospitalisation People:** Total number of people hospitalised due to fall (12 months)<br>**Injuries People:** Total number of people who experienced a fall-related fracture (12 months)<br>**Institutionalisation People:** Total number of people admitted to nursing home (12 months)<br>**Mortality:** Total number of deaths (12 months)
**Notes**	**Location**: Calgary, Canada<br>**Funding Source**: Health Services Research and Innovation Fund of the Alberta Heritage Foundation for Medical Research<br>**Other notes**:

Risk of bias table

Bias	Authors' judgement	Support for judgement
Random sequence generation (selection bias)	Low risk	Quote: "the allocation sequence was computer generated"
Allocation concealment (selection bias)	Unclear risk	Quote: "[the allocation sequence was] concealed (in a locked cabinet) prior to randomization"
Blinding (performance bias and detection bias)	Low risk	Quote: "the RA [research assistant] remained blinded throughout the study as to each subject’s group assignment"
Incomplete outcome data (attrition bias)	High risk	13.1% of participants lost in intervention group, and 16.5% of participants lost in control group. Reasons for missing data differed by group. Only 48 percent of calendars for self-reported falls were returned.
Selective reporting (reporting bias)	Unclear risk	Study does not reference a trial protocol

Holland 2005

**Methods**	**Place of recruitment**: General and community hospitals: method of solicitation not reported<br>**Inclusion criteria**: aged 80+, prescribed two or more drugs on discharge, admitted as emergency and discharged to home<br>Excluded if: received dialysis, participated in intensive discharge service
**Participants**	**Number randomised**: 437 (Intervention) / 435 (Comparison)<br>**Mean age**: 85.4<br>**Sex (% female)**: 62.5%<br>**Mortality per year in the comparison group**: 29.0%<br>**Living alone (%):** 62.1%
**Interventions**	**Visitors' professional group**: Pharmacist<br>**Frequency and duration of visits**: 2 visits over 6 months<br>**Fall prevention:** No<br>**Included exercise**: No<br>**Included multidimensional geriatric assessment:** No<br>**Description of intervention:** Visits involved assessment of self-medication and drug adherence, patient and carer medication education, removal of out of date drugs, report of possible interactions, and recommendations of compliance aids. Follow-up visit to reinforce original advice.<br>**Description of comparison**: Usual care<br>**Implementation (fidelity and participant compliance)**: 84.4% of intervention participants received first visits. More than 90% of first visits occurred within two weeks of recruitment (mean 7.2 days), and visits lasted a mean (*SD*) of 61 (23) minutes. 69.2% of intervention participants received second visits, which took a mean (*SD*) of 42 (19) minutes. Visits generated a total of 933 recommendations or comments to general practitioners (2.58 recommendations on average per visited participant).
**Outcomes**	**A&E Number:** Total number of admissions to an A&E department (6 months)<br>**Health Related QoL:** Euroqol Visual Analogue Scale (3, 6 months); Euroqol-5 Dimensions health status questionnaire (3, 6 months)<br>**Institutionalisation People:** Total number of people admitted to nursing or residential home (6 months)<br>**Mortality:** Total number of deaths (6 months)
**Notes**	**Location**: Norfolk and Suffolk, U.K.<br>**Funding Source**: NHS Eastern Region R&D and the Academic Pharmacy Practice Unit of the University of East Anglia<br>**Other notes**: Estimated cost of intervention was £144 per participant

Risk of bias table

**Bias**	**Authors' judgement**	**Support for judgement**
Random sequence generation (selection bias)	Low risk	Quote: "computer generated sequence in blocks of varying length"
Allocation concealment (selection bias)	Low risk	Quote: "we used third party telephone randomisation"
Blinding (performance bias and detection bias)	High risk	Quote: "the project coordinator contacted all patients by telephone at three months and six months to collect data"
Incomplete outcome data (attrition bias)	Low risk	Primary outcome data were available for 95.1% of of participants. Reasons for missing data were similar between groups
Selective reporting (reporting bias)	Low risk	The trial ISRCTN is 06813178

Huang 2004

**Methods**	**Place of recruitment**: location of recruitment not reported; method of solicitation not reported<br>**Inclusion criteria**: aged 65+
**Participants**	**Number randomised**: 60 (Intervention) / 60 (Comparison)<br>**Mean age**: 72.0<br>**Sex (% female)**: 45.8%<br>**Mortality per year in the comparison group**: Not reported<br>**Living alone (%):** Not reported
**Interventions**	**Visitors' professional group**: Community health professional<br>**Frequency and duration of visits**: 2 visits over 4 months<br>**Fall prevention:** Yes<br>**Included exercise**: No<br>**Included multidimensional geriatric assessment:** Yes<br>**Description of intervention:** Visitors assessed medical, psychosocial, and environmental falls risk factors. They also provided a falls checklist and an individualised brochure tailored to each participant based on assessment.<br>**Description of comparison**: Standardized fall-prevention information.<br>**Implementation (fidelity and participant compliance)**: Not reported.
**Outcomes**	**Falls People:** Total number of people who experienced a fall (4 months)<br>**Falls Subjective:** Falls self-efficacy (4 months)
**Notes**	**Location**: Hsin-Chu County, northwest Taiwan<br>**Funding Source**: Not reported<br>**Other notes**:

Risk of bias table

**Bias**	**Authors' judgement**	**Support for judgement**
Random sequence generation (selection bias)	Unclear risk	Quote: "half of the sample was randomly assigned as the experimental group, and the other half as the comparison group"
Allocation concealment (selection bias)	Unclear risk	Quote: "half of the sample was randomly assigned as the experimental group, and the other half as the comparison group
Blinding (performance bias and detection bias)	High risk	Quote: "the researcher completed three home visits in a 4-month period (HV1, HV2, and HV3).... During HV3, post-tests were administered to both experimental and comparison subjects"
Incomplete outcome data (attrition bias)	Low risk	Outcome data were available for 94.2% of participants
Selective reporting (reporting bias)	Unclear risk	Study does not reference a trial protocol

Kingston 2001

**Methods**	**Place of recruitment**: A&E department case records; method of solicitation not reported<br>**Inclusion criteria**: aged 65-79, female, case reference to "fall"<br>Excluded if: admitted to hospital or institutional care
**Participants**	**Number randomised**: 60 (Intervention) / 49 (Comparison)<br>**Mean age**: 71.9<br>**Sex (% female)**: 100%<br>**Mortality per year in the comparison group**: Not reported<br>**Living alone (%):** Not reported
**Interventions**	**Visitors' professional group**: Health professional<br>**Frequency and duration of visits**: 1 visit over 12 months<br>**Fall prevention:** Yes<br>**Included exercise**: Yes<br>**Included multidimensional geriatric assessment:** No<br>**Description of intervention:** Visitors provided advice for pain control and medication, how to get up after a fall, falls risk factors, diet, and exercise. Visits also included referrals to other health services.<br>**Description of comparison**: Usual care<br>**Implementation (fidelity and participant compliance)**: Not reported
**Outcomes**	**Health Related QoL:** Medical Outcomes Study Short Form Health Survey-36 items for General Health (3 months); Mental Health (3 months); Physical Health (3 months)
**Notes**	**Location**: North Staffordshire, U.K.<br>**Funding Source**: Not reported<br>**Other notes**:

Risk of bias table

**Bias**	**Authors' judgement**	**Support for judgement**
Random sequence generation (selection bias)	Unclear risk	Quote: "randomly allocated to control or intervention groups"
Allocation concealment (selection bias)	Unclear risk	Quote: "randomly allocated to control or intervention groups"
Blinding (performance bias and detection bias)	Low risk	Quote: "both groups (intervention and control) were assessed by face-to-face interview with an independent researcher"
Incomplete outcome data (attrition bias)	Unclear risk	15.0% of intervention participants and 16.3% of control participants were lost to follow-up. The authors did not provide reasons for missing data
Selective reporting (reporting bias)	Unclear risk	Study does not reference a trial protocol

Kono 2004

**Methods**	**Place of recruitment**: location of recruitment not reported; method of solicitation not reported<br>**Inclusion criteria**: aged 65+, able to walk only with assistance, went outdoors less than 3 times a week
**Participants**	**Number randomised**: 59 (Intervention) / 60 (Comparison)<br>**Mean age**: 82.7<br>**Sex (% female)**: 47.0%<br>**Mortality per year in the comparison group**: 6.7%<br>**Living alone (%):** Not reported
**Interventions**	**Visitors' professional group**: Public health nurses<br>**Frequency and duration of visits**: 4.3 visits on average over 18 months<br>**Fall prevention:** No<br>**Included exercise**: No<br>**Included multidimensional geriatric assessment:** Yes<br>**Description of intervention:** Visits included comprehensive in-home assessments, specific care recommendations, and the offering of care services if needed.<br>**Description of comparison**: Usual care<br>**Implementation (fidelity and participant compliance)**: Not reported
**Outcomes**	**Falls Subjective:** Falls self-efficacy (18 months)<br>**Functioning ADL/IADL:** Barthal Activities of Daily Living Index (18 months); TMIG Index of Competence (18 months)<br>**Hospitalisation People:** Total number of people hospitalised (18 months)<br>**Institutionalisation People:** Total number of people admitted to nursing home (18 months)<br>**Mortality:** Total number of deaths (18 months)<br>**Psychiatric Anxiety and Depression:** Geriatric Depression Scale (18 months)
**Notes**	**Location**: Saku City, Japan<br>**Funding Source**: Japan Ministry of Health, Labour Welfare, Kimura Foundation for Nursing Education, Mitsubishi Foundation, and Pfizer Health Research Foundation<br>**Other notes**:

Risk of bias table

Bias	Authors' judgement	Support for judgement
Random sequence generation (selection bias)	Unclear risk	Quote: "randomly assigned to intervention or control group"
Allocation concealment (selection bias)	Unclear risk	Quote: "sealed envelopes"
Blinding (performance bias and detection bias)	Unclear risk	The authors did not sufficiently describe how they assessed outcomes
Incomplete outcome data (attrition bias)	Low risk	Missing outcome data low and balanced between groups.
Selective reporting (reporting bias)	High risk	Study fails to include sufficient information for meta-analysis of relevant outcomes

Kono 2011

**Methods**	**Place of recruitment**: Long-Term Care Insurance Registries; solicited via mail.<br>**Inclusion criteria**: Aged 65+, ambulatory, living at home, and not having utilised formal long-term care services for the past 3 months, and frail.
**Participants**	**Number randomised**: 161 (Intervention) / 162 (Comparison)<br>**Mean age**: 79.9<br>**Sex (% female)**: 74.0%<br>**Mortality per year in the comparison group**: 6.2%<br>**Living alone (%):** 27.9%
**Interventions**	**Visitors' professional group**: Nurses, Care Managers, or Social Workers<br>**Frequency and duration of visits**: Visits every 6 months for 2 years (4 visits total)<br>**Fall prevention:** No<br>**Included exercise**: No<br>**Included multidimensional geriatric assessment:** Yes<br>**Description of intervention:** Visits included structured multidimensional interview-based assessments of five key elements: locomotion, daily activities, social contacts or relationships with other people, health conditions, and signs of abuse.<br>**Description of comparison**: No treatment<br>**Implementation (fidelity and participant compliance)**: See Table 3. For the majority of participants, all four visits were implemented (first visit: 87%, second: 85.7%, third: 83.9%, fourth 83.9%). A total of 13 additional home visits between the routine home visits were provided to 11 elders in the intervention group.
**Outcomes**	**Funcational ADLs/IADLs**: Barthel Index (24 months); Tokyo Metropolitan Institute of Gerontology Index of Competence (24 months)<br>Institutionalisation People: Total number of people institutionalised (24 months)<br>Mortality: Total number of deaths (24 months)<br>Psychiatric Anxiety and Depression: Geriatric Depression Scale – Japanese translation (24 months)
**Notes**	**Location**: Izumiotsu, Sennan, and Misaki, in Osaka, Japan<br>**Funding Source**: Japan Society for the Promotion of Science<br>**Other notes**:

Risk of bias table

**Bias**	**Authors' judgement**	**Support for judgement**
Random sequence generation (selection bias)	Low risk	Quote: “The remaining 323 participants were randomized to either the interven- tion group (*n* = 161) or usual care group (*n* = 162) by re- searchers using computer-generated random numbers stratified on the basis of gender, age group, and district within each community.”
Allocation concealment (selection bias)	Unclear risk	Did not report.
Blinding (performance bias and detection bias)	Low risk	Quote: “single-blind”
Incomplete outcome data (attrition bias)	Unclear risk	Quote: “All analyses were conducted by intention to treat (including participants who refused the intervention)”
Selective reporting (reporting bias)	High risk	Comment: Data at Year 1 were not reported for hospitalisation, institutionalisation, and mortality.<br>Quote: “The study protocol was registered for the UMIN clinical trials registry approved by ICMJE (no. UMIN000001113, April 07, 2008).”

Krebs 1998

**Methods**	**Place of recruitment**: Community agencies, senior centres, and senior housing sites; solicited by mail, telephone, and clinician referrals<br>**Inclusion criteria**: aged 60+, reported one or more functional limitations, had no medical history hindering exercise, were not receiving rehabilitation services, ambulatory with or without assistive devices, willingness to participate
**Participants**	**Number randomised**: 54 (Intervention) / 66 (Comparison)<br>**Mean age**: 74.3<br>**Sex (% female)**: 74.2%<br>**Mortality per year in the comparison group**: Not reported<br>**Living alone (%):** Did not report
**Interventions**	**Visitors' professional group**: Physical therapist<br>**Frequency and duration of visits**: 2 visits over 6 months<br>**Fall prevention:** Yes<br>**Included exercise**: Yes<br>**Included multidimensional geriatric assessment:** No<br>**Description of intervention:** Visits involved guidance during a 35-minute videotape programme of 11 limb-strengthing, resistance-based exercises.<br>**Description of comparison**: No-exercise treatment as usual<br>**Implementation (fidelity and participant compliance)**: Providers made average of 7.5 supplemental telephone contacts per intervention participant during the 6 months for assisting with intervention. 77.8% of intervention participants adhered to the recommended exercise frequency and level of resistance.
**Outcomes**	**No relevant outcome data reported**
**Notes**	**Location**: Boston, Massachusetts, U.S.A.<br>**Funding Source**: National Institutes of Health<br>**Other notes**:

Risk of bias table

Bias	Authors' judgement	Support for judgement
Random sequence generation (selection bias)	Unclear risk	Quote: "a prospective, single-blinded, randomized trial"
Allocation concealment (selection bias)	Unclear risk	Quote: "a prospective, single-blinded, randomized trial."
Blinding (performance bias and detection bias)	High risk	Care providers, who were not blind to participants' allocated intervention, assessed outcomes
Incomplete outcome data (attrition bias)	Unclear risk	The study did not address this item as no relevant outcome data were reported
Selective reporting (reporting bias)	High risk	The study report fails to include results for key outcomes that would be expected to have been reported for such a study

Lenaghan 2007

**Methods**	**Place of recruitment**: General Practice; solicited by mail and telephone.<br>**Inclusion criteria**: aged 80+, prescribed 4+ daily oral medications, and at least one of the following: living alone, confused mental state, vision or hearing impairment, medication-related morbidity, or 7+ regular oral medicines.<br>Excluded if: used adherence aid.
**Participants**	**Number randomised**: 68 (Intervention) / 66 (Comparison)<br>**Mean age**: 84.3<br>**Sex (% female)**: 65.7%<br>**Mortality per year in the comparison group**: 17.9%<br>**Living alone (%)**: Not reported
**Interventions**	**Visitors' professional group**: Community pharmacist<br>**Frequency and duration of visits**: 2 visits over 6-8 weeks<br>**Fall prevention:** No<br>**Included exercise**: No<br>**Included multidimensional geriatric assessment:** No<br>**Description of intervention:** Initial visit included patient education, removal of out-of-date drugs, assessment for adherence aid. Single follow-up visit was 6-8 weeks later to reinforce treatment plan and assess need for additional changes. Pharmacist and general practitioner also met to discuss medication changes.<br>**Description of comparison**: Usual care<br>**Implementation (fidelity and participant compliance)**: 94.2% of intervention participants received the intervention.
**Outcomes**	**Health Related QoL:** EQ-5d (6 months); Visual Analogue Scale (6 months)<br>**Hospitalisation Admissions:** Total number of hospital admissions (6 months)<br>**Institutionalisation People:** Total number of people admitted to nursing home (6 months)<br>**Mortality:** Total number of deaths (6 months)
**Notes**	**Location**: Norwich District, U.K.<br>**Funding Source**: NHS Executive Eastern Region research funding<br>**Other notes**: Estimated cost of intervention was £122 per participant

Risk of bias table

**Bias**	**Authors' judgement**	**Support for judgement**
Random sequence generation (selection bias)	Low risk	Quote: "randomisation was carried out by a third party, and was stratified by whether the patient lived alone"
Allocation concealment (selection bias)	Low risk	Quote: "randomisation was carried out by a third party"
Blinding (performance bias and detection bias)	Low risk	Primary outcomes obtained from hospital records. Secondary outcomes collected through telephone contact
Incomplete outcome data (attrition bias)	Low risk	Primary outcome data available for 98.5% of participants. Reasons for withdrawal for secondary outcome data were similar between groups
Selective reporting (reporting bias)	Unclear risk	Study does not reference a trial protocol

Lightbody 2002

**Methods**	**Place of recruitment**: A&E; Solicitation method not reported.<br>**Inclusion criteria**: aged 65+ years, attending A&E with a fall.<br>Excluded if: admitted to hospital as a result of the Index fall, lived in institutional care, or were out of the area.
**Participants**	**Number randomised**: 171 (Intervention) / 177 (Comparison)<br>**Mean age**: Median 75 (IQR 70-81)<br>**Sex (% female)**: 74.4%<br>**Mortality per year in the comparison group**: 8.0%<br>**Living alone (%):** 44.0%
**Interventions**	**Visitors' professional group**: Nurse<br>**Frequency and duration of visits**: 1 visit<br>**Fall prevention:** Yes<br>**Included exercise**: No<br>**Included multidimensional geriatric assessment:** Yes<br>**Description of intervention:** The intervention group was assessed for risk factors for falls at home by the falls nurse 2–4 weeks following the Index fall. Medical, functional, and environmental items were assessed. Participants were then given advice and education about safety in the home, and simple modifications were made with consent.<br>**Description of comparison**: Usual care<br>**Implementation (fidelity and participant compliance)**: 99.4% of intervention participants received the home visit.
**Outcomes**	**A&E People:** Total number of people presenting to A&E (6 months)<br>**Falls Number:** Total number of falls (6 months)<br>**Falls People:** Total number of people who experienced a fall (6 months)<br>**Functioning ADL/IADL:** Barthal index (6 months)<br>**Hospital Admissions:** Total number of falls-related hospital admissions (6 months)<br>**Hospital Days:** Total number of days spent in hospital beds (6 months)<br>**Mortality:** Total number of deaths (6 months)
**Notes**	**Location**: Liverpool, UK<br>**Funding Source**: North West Region NHS Executive; Liverpool and Wirral Research and Development Liaison Group<br>**Other notes**:

Risk of bias table

Bias	Authors' judgement	Support for judgement
Random sequence generation (selection bias)	Unclear risk	Quote: “Patients were block-randomized consecutively to groups.”
Allocation concealment (selection bias)	Unclear risk	See sequence generation.
Blinding (performance bias and detection bias)	Low risk	Outcomes obtained through records.
Incomplete outcome data (attrition bias)	High risk	Comment: Falls reported in the text do not match Table 5; based on the percentages in Table 5, denominators appear to exclude dropouts.<br>Quote: “Eighteen patients had died (11 intervention, 7 usual care), 12 had withdrawn (2 intervention, 10 usual care) and 4 were lost to follow up (3 intervention, 1 usual care). There were no significant group differences in mortality or withdrawal rates.”
Selective reporting (reporting bias)	Unclear risk	Study does not reference a trial protocol.

Lin 2007

**Methods**	**Place of recruitment**: Clinics and hospitals; method of solicitation not reported.<br>**Inclusion criteria**: aged 65+, required medical attention due to sustaining a fall in the previous 4 weeks.
**Participants**	**Number randomised**: 50 (Intervention) / 50 (Comparison)<br>**Mean age**: 76.8<br>**Sex (% female)**: 51.0%<br>**Mortality per year in the comparison group**: 0.0%<br>**Living alone (%):** 34.0.%
**Interventions**	**Visitors' professional group**: Physical therapist<br>**Frequency and duration of visits**: 8 visits over 4 months<br>**Fall prevention:** Yes<br>**Included exercise**: Yes<br>**Included multidimensional geriatric assessment:** No<br>**Description of intervention:** Visitors provided individualised physiological training consisting of stretching, muscle strengthening, and balance training at increasing levels of difficulty.<br>**Description of comparison**: Attention-matched control, with education group receiving social visits and pamphlets on fall prevention<br>**Implementation (fidelity and participant compliance)**: Not reported
**Outcomes**	**Falls People:** Total number of people who experienced a fall (8 months)<br>**Falls Subjective:** Fear of falling (6, 8 months)<br>**Functioning ADL/IADL:** Older Americans Resources and Services Multidimensional Functional Assessment Questionnaire (6, 8 months)<br>**Health Related QoL:** World Health Organisation Quality of Life Instrument Environmental (6, 8 months); Physical (6, 8 months); Psychological (6, 8 months); and Social scales (6, 8 months)<br>**Mortality:** Total number of deaths (6, 8 months)<br>**Psychiatric Anxiety and Depression:** Geriatric Depression Scale (6, 8 months)
**Notes**	**Location**: Shin-Sher township, Taiwan<br>**Funding Source**: The Bureau of Health Promotion, Department of Health, and the National Science Council, Taiwan<br>**Other notes**:

Risk of bias table

Bias	Authors' judgement	Support for judgement
Random sequence generation (selection bias)	Unclear risk	Quote: "participants were block randomized in groups of six to one of three intervention programs"
Allocation concealment (selection bias)	Unclear risk	Quote: "participants were block randomized in groups of six to one of three intervention programs"
Blinding (performance bias and detection bias)	Low risk	Quote: "two assessors, blinded to group assignment, were responsible for the evaluation of baseline and two follow-up assessments"
Incomplete outcome data (attrition bias)	Low risk	Reasons for missing data were similar between groups.
Selective reporting (reporting bias)	Unclear risk	Study does not reference a trial protocol

Liu-Ambrose 2008

**Methods**	**Place of recruitment**: Referral-based falls clinics; solicited by in-person contact at falls clinic<br>**Inclusion criteria**: aged 70+, community dwelling, could walk 3 meters, and at least one of the following: one additional fall not related to fainting in the previous year, completion of walking task, low physiological impairment.<br>Excluded if: progressive neurological condition, life expectancy of less than 12 months, high cognitive impairment.
**Participants**	**Number randomised**: 31 (Intervention) / 28 (Comparison)<br>**Mean age**: 82.2<br>**Sex (% female)**: 69.5%<br>**Mortality per year in the comparison group**: 10.5%<br>**Living alone (%):** Not reported
**Interventions**	**Visitors' professional group**: Physiotherapists<br>**Frequency and duration of visits**: 5 visits over 6 months<br>**Fall prevention:** Yes<br>**Included exercise**: Yes<br>**Included multidimensional geriatric assessment:** No<br>**Description of intervention**: Programme consisted of home-based balance and strength retraining routine. Physiotherapists prescribed a selection of exercises at the first visit, and returned every other week three additional times to make progressive adjustments to the protocol. Physiotherapists provided participants an exercise manual and encouraged them to perform the exercise programme three times per week and to walk at least twice per week. Physiotherapists made a final visit at 6 months to check and encourage continued adherence.<br>**Description of comparison**: Usual care.<br>**Implementation (fidelity and participant compliance):** 25.0% of intervention participants completed the programme 3 times per week or more, 58.3% completed the programme 2 times per week or more, and 66.7% completed the programme at least once per week.
**Outcomes**	**Falls People:** Total number of people who experienced a fall (12 months)<br>**Falls Subjective:** Psychsiological Profile Assessment Z-score (6 months)<br>**Functioning ADL/IADL:** Timed up and go test (6 months)<br>**Functioning Cognitive:** Verbal Digits Backwards Test (6 months); Stroop Color-Word Test (6 months); Trail Making Test Part B (6 months)<br>**Mortality:** Total number of deaths (6 months)
**Notes**	**Location**: Vancouver, British Columbia, Canada<br>**Funding Source**: Canadian Institutes of Health Research<br>**Other notes**:

Risk of bias table

**Bias**	**Authors' judgement**	**Support for judgement**
Random sequence generation (selection bias)	Low risk	Quote: "the randomization sequence was computer generated (www.randomization.com)"
Allocation concealment (selection bias)	Low risk	Quote: "the Family Practice Research Coordinator at the University of British Columbia held this sequence independently and remotely"
Blinding (performance bias and detection bias)	Low risk	Quote: "research assistants who administered the fall risk assessments and conducted the monthly telephone interviews were all blinded to group assignment"
Incomplete outcome data (attrition bias)	High risk	Significantly more control participants (23.7%) than intervention participants (11.1%) refused initial baseline assessment and were lost to subsequent follow-ups
Selective reporting (reporting bias)	Unclear risk	Study does not reference a trial protocol

Luker 1981

**Methods**	**Place of recruitment**: General Practice; solicited by mail<br>**Inclusion criteria**: aged 70+, no mental impairment, female, living alone
**Participants**	**Number randomised**: Appx 60 (Intervention) / Appx 60 (Comparison)<br>**Mean age**: Not reported<br>**Sex (% female)**: 100%<br>**Mortality per year in the comparison group**: Not reported<br>**Living alone (%):** 100%
**Interventions**	**Visitors' professional group**: Health rofessional<br>**Frequency and duration of visits**: 4 visits over 4 months<br>**Fall prevention:** No<br>**Included exercise**: No<br>**Included multidimensional geriatric assessment:** No<br>**Description of intervention:** Visitors identified participants' potential and actual health problems and then developed a plan of action tailored to discovered problems.<br>**Description of comparison**: Usual care<br>**Implementation (fidelity and participant compliance):** Not reported
**Outcomes**	**No relevant outcome data reported**
**Notes**	**Location**: Scotland<br>**Funding Source**: Scottish Home and Health Department<br>**Other notes**:

Risk of bias table

Bias	Authors' judgement	Support for judgement
Random sequence generation (selection bias)	Unclear risk	Quote: "the subjects were randomly allocated to either the experimental or control groups"
Allocation concealment (selection bias)	Unclear risk	Quote: "the subjects were randomly allocated to either the experimental or control groups"
Blinding (performance bias and detection bias)	High risk	The same person carried out intervention and assessment visits, and protocols were unique to each trial arm
Incomplete outcome data (attrition bias)	Low risk	No participants were lost to follow-up
Selective reporting (reporting bias)	High risk	Outcomes of interest in the review are reported insufficiently for inclusion in a meta-analysis

Luukinen 2006

**Methods**	**Place of recruitment**: City registry; solicited by mail<br>**Inclusion criteria**: aged 85+ and at least one of the following: poor cognition, 2 or more falls during preceding year, frequent feelings of loneliness, poor self-rated health, poor visual acuity, poor hearing, depression, impaired balance
**Participants**	**Number randomised**: 243 (Intervention) / 243 (Comparison)<br>**Mean age**: 88.0<br>**Sex (% female)**: 79.0%<br>**Mortality per year in the comparison group**: 15.4%<br>**Living alone (%):** Not reported
**Interventions**	**Visitors' professional group**: Occupational therapist, physical therapist<br>**Frequency and duration of visits**: 3 visits on average over 3 months<br>**Fall prevention:** Yes<br>**Included exercise**: Yes<br>**Included multidimensional geriatric assessment:** No<br>**Description of intervention:** Visits involved a physiological assessment and physiotherapy treatment with a problem solving approach at home or in out-patient rehabilitation centre.<br>**Description of comparison**: Usual care<br>**Implementation (fidelity and participant compliance)**: Not reported
**Outcomes**	**A&E People**: Total number of people seeking first aid in hospital (16 months)<br>**Falls Number:** Total number of falls (16 months)<br>**Falls People:** Total number of people who experienced a fall (16 months)<br>**Functioning ADL/IADL:** Severe restriction (16 months)<br>**Functioning Cognitive:** Mini-Mental State Examination (16 months)<br>**Health Related QoL:** Poor self-related health (16 months)<br>**Injuries Number:** Number of injurious falls per person (16 months)<br>**Injuries People:** Total number of people experiencing injury due to fall (16 months)<br>**Mortality:** Total number of deaths (16 months)<br>**Psychiatric Anxiety and Depression:** Geriatric Depression Scale (16 months)
**Notes**	**Location**: Oulu, Finland<br>**Funding Source**: Ministry of Health and Social Affairs of Finland<br>**Other notes**:

Risk of bias table

Bias	Authors' judgement	Support for judgement
Random sequence generation (selection bias)	Low risk	Quote: "randomization was done by the study statistician using a random numbers table"
Allocation concealment (selection bias)	Unclear risk	Quote: "randomization was done by the study statistician using a random numbers table"
Blinding (performance bias and detection bias)	Low risk	Quote: "the research nurse was blinded to the randomization"
Incomplete outcome data (attrition bias)	Low risk	Reasons for missing data were similar between groups
Selective reporting (reporting bias)	Unclear risk	Study does not reference a trial protocol

Markle-Reid 2006

**Methods**	**Place of recruitment**: Community Care Access Centre registry; method of solicitation not reported.<br>**Inclusion criteria**: aged 75+, require assistance with personal care.<br>Excluded if: refused to give informed consent, poor cognitive or physical functioning, deemed eligible for nursing services, unable to understand English.
**Participants**	**Number randomised**: 144 (Intervention) / 144 (Comparison)<br>**Mean age**: 83.8<br>**Sex (% female)**: 76.9%<br>**Mortality per year in the comparison group**: Not reported<br>**Living alone (%):** Not reported
**Interventions**	**Visitors' professional group**: Registered nurse<br>**Frequency and duration of visits**: Median of 5 visits over 6 months<br>**Fall prevention:** No<br>**Included exercise**: No<br>**Included multidimensional geriatric assessment:** Yes<br>**Description of intervention:** Vistis involved usual care plus a proactive nursing health promotion intervention that included an initial health assessment, health education, identification of risk factors for functional decline, and bolstering of environmental support.<br>**Description of comparison**: Usual home care services<br>**Implementation (fidelity and participant compliance)**: 84.7 % of intervention participants received the intervention.
**Outcomes**	**Health Related QoL:** Medical Outcomes Study Short Form Health Survey-36 items for Mental Health (6 months) and Physical Health (6 months)<br>**Psychiatric Anxiety and Depression:** Center for Epidemiologic Studies Depression Scale (CES - D) (6 months)
**Notes**	**Location**: Ontario, Canada<br>**Funding Source**: Canadian Health Services Research Foundation, Ontario Ministry of Health and Long-term Care, Community Care Access Centre of Halton, McMaster University, System-linked Research Unit on Health & Social Services Utilisation<br>**Other notes**:

Risk of bias table

Bias	Authors' judgement	Support for judgement
Random sequence generation (selection bias)	Low risk	Quote: "eligible and consenting participants were randomized ... using a computerized randomization schedule"
Allocation concealment (selection bias)	Unclear risk	Quote: "eligible and consenting participants were randomized ... using a computerized randomization schedule"
Blinding (performance bias and detection bias)	Low risk	Quote: "trained interviewers, blinded to the purpose of the study and the treatment assignment, obtained baseline (prerandomization) and follow-up outcome assessments at 6 months from the participants"
Incomplete outcome data (attrition bias)	Unclear risk	16.7% of intervention participants and 15.3% of control participants were lost to follow-up. The authors did not report reasons for missing data by group
Selective reporting (reporting bias)	Unclear risk	Study did not reference a trial protocol

Markle-Reid 2010

**Methods**	**Place of recruitment**: Community Care Access Centre registry; method of solicitation not reported.<br>**Inclusion criteria**: aged 75+, low cognitive impairment, at risk for falls, required assistance for personal care, competent in English or with a translator available.
**Participants**	**Number randomised**: 55 (Intervention) / 54 (Comparison)<br>**Mean age**: Not reported<br>**Sex (% female)**: 71.7%<br>**Mortality per year in the comparison group**: 14.5%<br>**Living alone (%):** 43.0%
**Interventions**	**Visitors' professional group**: Case manager, registered nurse, occupational therapist, physiotherapist, registered dietitian<br>**Frequency and duration of visits**: Minimum of 6 visits over 6 months<br>**Fall prevention:** Yes<br>**Included exercise**: No<br>**Included multidimensional geriatric assessment:** Yes<br>**Description of intervention:** Visits involved assessment of known risk factors for falls and other factors influencing health, management of modifiable fall risk factors, and support and education about falls prevention.<br>**Description of comparison**: Usual care<br>**Implementation (fidelity and participant compliance)**: 100% of intervention participants received the intervention.
**Outcomes**	**Falls Number:** Number of falls per person (6 months)<br>**Falls Subjective:** Modified Falls Efficacy Scale (6 months)<br>**Functioning Cognitive:** Short Mini-Mental State Examination (6 months)<br>**Health Related QoL:** Medical Outcomes Study Short Form Health Survey-36 items for Mental Health (6 months) and Physical Health (6 months)<br>**Hospitalisation Days:** Number of acute hospital days for fall per person (6 months)<br>**Mortality**: Total number of deaths (6 months)<br>**Psychiatric Anxiety and Depression:** Center for Epidemiologic Studies Depression Scale (CES - D) (6 months)
**Notes**	**Location**: Ontario, Canada<br>**Funding Source**: Canadian Patient Safety Institute (CPSI – Grant Number RFAAA0506164), Community Care Access Centre of Halton, McMaster University System-Linked Research Unit on Health and Social Services Utilization, and Ontario Ministry of Health and Long-Term Care<br>**Other notes**:

Risk of bias table

**Bias**	**Authors' judgement**	**Support for judgement**
Random sequence generation (selection bias)	Low risk	Quote: "randomly generated numbers constructed by a biostatistician who was not involved in the recruitment process"
Allocation concealment (selection bias)	Low risk	Quote: "randomization was achieved using consecutively numbered, sealed, opaque envelopes"
Blinding (performance bias and detection bias)	Low risk	Quote: "trained interviewers, blinded to the purpose of the study and group assignment, assessed participants at baseline and six months through a structured in-home interview"
Incomplete outcome data (attrition bias)	High risk	Significantly more control participants (14.5%) than intervention participants (3.7%) refused follow-up
Selective reporting (reporting bias)	High risk	Trial Registration: clinicaltrials.gov identifier: NCT00463658<br>Outcomes from protocol not reported in study

McEwan 1990

**Methods**	**Place of recruitment**: General Practice; method of solicitation not reported.<br>**Inclusion criteria**: aged 75+, registered with the General Practice in 1986<br>Excluded if: "too ill" or in hospital
**Participants**	**Number randomised**: 151 (Intervention) / 145 (Comparison)<br>**Mean age**: Not reported<br>**Sex (% female)**: Not reported<br>**Mortality per year in the comparison group**: 9.5%<br>**Living alone (%):** Not reported
**Interventions**	**Visitors' professional group**: Registered nurse<br>**Frequency and duration of visits**: 1 visit over 20 months<br>**Fall prevention:** No<br>**Included exercise**: No<br>**Included multidimensional geriatric assessment:** Yes<br>**Description of intervention:** Visits involved special medical screening and assessment, health information provision, and referals and/or advice based on assessment results.<br>**Description of comparison**: Usual care<br>**Implementation (fidelity and participant compliance)**: Not reported
**Outcomes**	**Hospitalisation People:** Total number of people in hospital or too ill to be contacted at end of study (20 months)<br>**Mortality:** Total number of deaths (20 months)
**Notes**	**Location**: Newcastle, England<br>**Funding Source**: Newcastle Health Authority<br>**Other notes**:

Risk of bias table

**Bias**	**Authors' judgement**	**Support for judgement**
Random sequence generation (selection bias)	Unclear risk	Quote: "elderly people were stratified into the age-sex groups ..., then randomly allocated to the test ... and control groups"
Allocation concealment (selection bias)	Unclear risk	Quote: "elderly people were stratified into the age-sex groups ..., then randomly allocated to the test ... and control groups"
Blinding (performance bias and detection bias)	Low risk	Quote: "evaluated by an independent interviewer (a community nurse trained in interviewing techniques and who had no previous connection with the care plan team)"
Incomplete outcome data (attrition bias)	High risk	Reasons for missing outcome data differed considerably by group, namely for refusing allocated intervention and follow-up evaluation
Selective reporting (reporting bias)	Unclear risk	Study did not reference trial protocol number

Nelson 2004

**Methods**	**Place of recruitment**: greater Boston area; solicited by newspaper and radio advertisements, and community presentations.<br>**Inclusion criteria**: aged 70+, not currently exercising more than 1 day per week, self-reported at least two functional limitations, low physical performance<br>Excluded if: unstable cardiovascular disease, psychiatric disorders, neurological or muscular disease, terminal illness, or cognitive impairment
**Participants**	**Number randomised**: 34 (Intervention) / 38 (Comparison)<br>**Mean age**: 77.8<br>**Sex (% female)**: 79.2%<br>**Mortality per year in the comparison group**: 0.0%<br>**Living alone (%):** Not reported
**Interventions**	**Visitors' professional group**: Exercise physiologist<br>**Frequency and duration of visits**: 11 visits over 6 months<br>**Fall prevention:** No<br>**Included exercise**: Yes<br>**Included multidimensional geriatric assessment:** No<br>**Description of intervention:** Visits involved exercise programme focusing on strength, balance, and encouragement to increase overall activity. Visitors gave participants a detailed booklet, dumbbells, and adjustable ankle weights. Physiologist also asked about new medical conditions and falls history.<br>**Description of comparison**: Attention-matched control given nutritional education<br>**Implementation (fidelity and participant compliance)**: Intervention participants completed an average of 82.0% of their prescribed exercise sessions. Comparison participants complied with 75% of their nutrition programme
**Outcomes**	**Functioning ADL/IADL:** Physical Performance Test (6 months)<br>**Mortality:** Total number of deaths (6 months)
**Notes**	**Location**: Greater Boston area, Massachusetts, U.S.A.<br>**Funding Source**: Brookdale Foundation, U.S. Dept of Agriculture; National Institute of Health, USDA Food and Agriculture Sciences Graduate Fellowship, National Institute on Health Research Training programme in Nutrition and Aging<br>**Other notes**: There were two adverse events during the study. One participant in the exercise group fell while doing the tandem walk at home, which resulted in bruises to both arms and one knee, and one participant in the attention-control group had an episode of food poisoning. Both participants were able to continue in the study once they recovered.

Risk of bias table

Bias	Authors' judgement	Support for judgement
Random sequence generation (selection bias)	Unclear risk	Quote: "participants were randomly assigned to either group in blocks of four by gender and age (70–79 years/80 years or older)"
Allocation concealment (selection bias)	Unclear risk	Quote: "participants were randomly assigned to either group in blocks of four by gender and age (70–79 years/80 years or older)"
Blinding (performance bias and detection bias)	Low risk	Quote: "a single assessor (A.N.), who was blinded to participants' group assignment, carried out all baseline and final testing"
Incomplete outcome data (attrition bias)	Low risk	Outcome data were available for 97.2% of participants.
Selective reporting (reporting bias)	Unclear risk	Study did not reference trial protocol

Newbury 2001

**Methods**	**Place of recruitment**: Databases of six General Practices; solicitation by contacting every 20th name on the register<br>**Inclusion criteria**: aged 75+, living independently in community
**Participants**	**Number randomised**: 50 (Intervention) / 50 (Comparison)<br>**Mean age**: 79.2<br>**Sex (% female)**: 63.0%<br>**Mortality per year in the comparison group**: 10.0%<br>**Living alone (%):** Not reported
**Interventions**	**Visitors' professional group**: Registered nurse<br>**Frequency and duration of visits**: 1 visit over 6 months<br>**Fall prevention:** Yes<br>**Included exercise**: No<br>**Included multidimensional geriatric assessment:** Yes<br>**Description of intervention:** Visits involved in-home health check with emphasis on functional ability. Visitors counted participants' number of health problems and reported them to participants' general practitioners.<br>**Description of comparison**: Usual care<br>**Implementation (fidelity and participant compliance)**: 96.0% of intervention participants received the intervention.
**Outcomes**	**Falls People**: Total number of people who experienced a fall (12 months)<br>**Functioning ADL/IADL:** Barthal Activities of Daily Living Index (12 months)<br>**Functioning Cognitive:** Mini-Mental State Examination (12 months)<br>**Health Related QoL:** Medical Outcomes Study Short Form Health Survey-36 items for Bodily Pain (12 months); General Health (12 months); Mental Health (12 months); Physical Health (12 months); Role Emotional (12 months); Role Physical (12 months); Social Functioning (12 months); and Vitality (12 months)<br>**Institutionalisation People:** Total number of people admitted to nursing home (12 months)<br>**Mortality:** Total number of deaths (12 months)
**Notes**	**Location**: Adelaide Western Division of General Practice, Australia<br>**Funding Source**: General Practice Evaluation Programme, Commonweath Department of Health and Aged Care<br>**Other notes**:

Risk of bias table

**Bias**	**Authors' judgement**	**Support for judgement**
Random sequence generation (selection bias)	Low risk	Quote: "randomisation was by random numbers contained in sequentially numbered, sealed envelopes"
Allocation concealment (selection bias)	Low risk	Quote: "randomisation was by random numbers contained in sequentially numbered, sealed envelopes"
Blinding (performance bias and detection bias)	Low risk	Quote: "the research team was blind to randomisation when reporting problems to the GPs"
Incomplete outcome data (attrition bias)	Low risk	Reasons for missing outcome data were similar between groups
Selective reporting (reporting bias)	Unclear risk	Study did not reference trial protocol number

Pathy 1992

**Methods**	**Place of recruitment**: General Practice; method of solicitation not reported.<br>**Inclusion criteria**: aged 65+, living in domestic accommodation, registered with the practice
**Participants**	**Number randomised**: 369 (Intervention) / 356 (Comparison)<br>**Mean age**: 73.3<br>**Sex (% female)**: 59.9%<br>**Mortality per year in the comparison group**: 8.43%<br>**Living alone (%):** 33.5%
**Interventions**	**Visitors' professional group**: Health professional<br>**Frequency and duration of visits**: 9 visits<br>**Fall prevention:** No<br>**Included exercise**: No<br>**Included multidimensional geriatric assessment:** Yes<br>**Description of intervention:** The research team sent screening questionnaires were sent to eligible patients annually. If the questionnaire indicated a problem, a health professional visited the patient at home and provided advice, health education, and referrals to their general practitioners or community services. The visitor was not directly involved in patient care.<br>**Description of comparison**: Usual care<br>**Implementation (fidelity and participant compliance)**: 60.0% of intervention participants received a visit.
**Outcomes**	**Health Related QoL:** Self-rated health (36 months)<br>**Hospitalisation Admissions:** Total number of admissions to hospital (36 months)<br>**Hospitalisation Days:** Total number of days in hospital (36 months)<br>**Institutionalisation People:** Total number of people admitted to nursing home (36 months)<br>**Mortality:** Total number of deaths (36 months)
**Notes**	**Location**: Cardiff, Wales<br>**Funding Source**: Nuffield Provincial Hospital Trust<br>**Other notes**:

Risk of bias table

Bias	Authors' judgement	Support for judgement
Random sequence generation (selection bias)	Unclear risk	Quote: "patients were randomly allocated to intervention or control"
Allocation concealment (selection bias)	Unclear risk	Quote: "patients were randomly allocated to intervention or control"
Blinding (performance bias and detection bias)	Low risk	Quote: "staff blind to the purpose and design of the study kept an independent record of all deaths, hospital admissions, and admissions to permanent residential care"
Incomplete outcome data (attrition bias)	Unclear risk	Insufficient reporting of attrition and reasons for exclusions to permit judgement
Selective reporting (reporting bias)	High risk	Some outcomes (e.g., quality of Life) were measured and not fully reported

Pighills 2011

**Methods**	**Place of recruitment**: GP registrars; solicited via mail.<br>**Inclusion criteria**: aged 70+ years, history of falls.<br>Excluded if: living in nursing or residential homes, currently receiving occupational therapy, or those who had received a falls-specific OT intervention in the preceding year.
**Participants**	**Number randomised**: 87 (Intervention) / 78 (Comparison)<br>**Mean age**: 78.9<br>**Sex (% female)**: 69.1%<br>**Mortality per year in the comparison group**: 7.7%<br>**Living alone (%):** Did not report
**Interventions**	**Visitors' professional group**: Occupational therapist<br>**Frequency and duration of visits**: 1 visit lasting 1.5 to 2 hours<br>**Fall prevention:** Yes<br>**Included exercise**: No<br>**Included multidimensional geriatric assessment:** No<br>**Description of intervention:** The visit consisted of an environmental assessment and modification of the home by OT.<br>**Description of comparison**: Usual care.<br>**Implementation (fidelity and participant compliance)**: 3.5% of intervention participants refused assessment. 60% participants partially adhered to recommendations for home modification, while 18% fully adhered to recommendations.
**Outcomes**	**Falls Number:** Total number of falls (12 months)<br>**Falls People:** Total number of people who experienced a fall (12 months)<br>**Falls Subjective:** Falls Efficacy Scale International Version (12 months)<br>**Functional ADL/IADL:** Barthal Index (12 months)<br>**Health-Related QoL:** EuroQol (12 months); SF-12 Physical (12 months); SF-12 Mental (12 months)<br>**Mortality:** Total number of deaths (12 months)
**Notes**	**Location**: Yorkshire, UK<br>**Funding Source**: Institute for Health Research National Coordinating Centre for Research Capacity Development; Department of Health Research Capacity Development Programme<br>**Other notes**: We did not extract data from a third group that received an intervention delivered by an unqualified trained assessor.

Risk of bias table

Bias	Authors' judgement	Support for judgement
Random sequence generation (selection bias)	Low risk	Quote: "The York Trials Unit independently and remotely conducted simple Web-based randomization because the anticipated sample size of the trial was to exceed 100."
Allocation concealment (selection bias)	Low risk	Quote: "The computer-generated outcome of randomization was automatically e-mailed to an independent person who passed the participant's case notes on to the contact person for the group to which they had been randomized."
Blinding (performance bias and detection bias)	Low risk	Quote: "All reported falls were followed up with a blinded, structured telephone interview to investigate the circumstances and consequences."<br>Quote: "Staff at the York Trials Unit inputted questionnaire data, which was checked independently twice for accuracy. Those assessing the outcome of the trial were blinded to group assignment."
Incomplete outcome data (attrition bias)	High risk	Comment: 217 / 238 participants provided full outcome data. It is not clear how means were calculated or the denominator used for dichotomous outcomes. Most outcomes are rounded to nearest whole number.]<br>Quote: "The primary analysis was performed on an intention to treat (ITT) basis."<br>Quote: "Six people died or withdrew without providing any follow-up data; 10 died having provided some follow-up data; three withdrew having provided 1, 3, and 7 months of falls data but did not return follow-up questionnaires; and two were lost to follow-up in Month 12. Attrition at 12 months was 4%, excluding deaths (Figure 1)."
Selective reporting (reporting bias)	Unclear risk	Study does not reference a trial protocol.

Ploeg 2010

**Methods**	**Place of recruitment**: Primary care networks; solicited by postal screening of patients from selected family physicians.<br>**Inclusion criteria**: aged 75+, spoke English.<br>Excluded if: needed palliative care, had major surgery scheduled in next year, planned to leave country for more than 1 month in next year.
**Participants**	**Number randomised**: 361 (Intervention) / 358 (Comparison)<br>**Mean age**: 81.1<br>**Sex (% female)**: 53.1%<br>**Mortality per year in the comparison group**: 2.8%<br>**Living alone (%):** 33.9%
**Interventions**	**Visitors' professional group**: Home care nurse<br>**Frequency and duration of visits**: Average of 3.03 visits over 12 months<br>**Fall prevention:** No<br>**Included exercise**: No<br>**Included multidimensional geriatric assessment:** Yes<br>**Description of intervention:** Visits involved a comprehensive initial assessment, collaborative care planning, health promotion, and referral to community health and social support service. Follow-up visits were performed as necessary to encourage adherence.<br>**Description of comparison**: Usual care<br>**Implementation (fidelity and participant compliance)**: 100% of intervention participants received the intervention. Intervention participants received a mean of 1.17 telephone calls from the nurse in addition to the home visits over the year.
**Outcomes**	**Functioning ADL/IADL:** Older Americans Resources and Services Multidimensional Functional Assessment Questionnaire (12 months)<br>**Health Related QoL:** Quality adjusted life years (12 months); Self-rated health (12 months)<br>**Mortality:** Total number of deaths (12 months)
**Notes**	**Location**: Hamilton, Ontario, Canada<br>**Funding Source**: Ontario Ministry of Health and Long Term Care, Primary Health Care Transition Fund<br>**Other notes**:

Risk of bias table

**Bias**	**Authors' judgement**	**Support for judgement**
Random sequence generation (selection bias)	Low risk	Quote: "the random numbers used to assign the block size and choice of allocation within blocks came from the Rand tables of random digits"
Allocation concealment (selection bias)	Low risk	Quote: "the allocation sequence was kept in the locked office of the statistician and was inaccessible to staff making decisions about patients' eligibility"
Blinding (performance bias and detection bias)	Low risk	Quote: "research assistants were thus blinded to group assignment"
Incomplete outcome data (attrition bias)	Low risk	Reasons for missing outcome data were similar between groups
Selective reporting (reporting bias)	High risk	Outcome data was collected 6 months but the authors only reported data at 12 months

Robertson 2001a

**Methods**	**Place of recruitment**: Computerised registers at 17 General Practices; solicited by mail from doctor.<br>**Inclusion criteria**: aged 75+<br>Excluded if: unable to walk around own residence, receiving physiotherapy, not able to understand the requirements of the trial
**Participants**	**Number randomised**: 121 (Intervention) / 119 (Comparison)<br>**Mean age**: 80.8<br>**Sex (% female)**: 67.5%<br>**Mortality per year in the comparison group**: 5.0%<br>**Living alone (%):** 53.0%
**Interventions**	**Visitors' professional group**: Registered nurse<br>**Frequency and duration of visits**: 4 visits over 8 weeks, with 1 additional visit at 6 months<br>**Fall prevention:** Yes<br>**Included exercise**: Yes<br>**Included multidimensional geriatric assessment:** No<br>**Description of intervention:** Visitors provided individually presecribed sets of muscle strengthening and balance retraining exercises of progressive difficulty, in addition to a walking plan. Visitors expected participants to exercise at least three times a week and to walk twice a week for a year. Nurses called participants to maintain motivation and discuss any problems in months where they did not visit.<br>**Description of comparison**: Usual care.<br>**Implementation (fidelity and participant compliance)**: 82.6% of intervention participants that received intervention. 43.4% of intervention participants carried out their prescribed exercise programme three or more times a week, whereas 66.6% of carried it out at least 2 times per week. 66.1% of intervention participants walked at least 2 times per week at 1 year follow up.
**Outcomes**	**A&E Number:** Number of fall-related visits to an A&E department per person (12 months)<br>**Falls Number:** Number of falls per person (12 months)<br>**Injuries Number:** Number of injurious falls per person (12 months)<br>**Mortality:** Total number of deaths (12 months)
**Notes**	**Location**: West Auckland, New Zealand<br>**Funding Source**: Health Funding Authority Northern Division, Accident Rehabilitation and Compensation Insurance Corporation of New Zealand, Trustbank Otago Community Trust research fellowship<br>**Other notes**: Estimated cost of intervention was NZ$432 per participant

Risk of bias table

**Bias**	**Authors' judgement**	**Support for judgement**
Random sequence generation (selection bias)	Low risk	Quote: "a statistician developed the schedule for group allocation using random numbers"
Allocation concealment (selection bias)	Low risk	Quote: "a statistician developed the schedule for group allocation using random numbers, and this was held at another centre. Participants were then informed of their group allocation by telephone"
Blinding (performance bias and detection bias)	Low risk	Quote: "the independent assessor telephoned participants to record the circumstances of the falls and any injuries or resource use as a result of the falls. She remained blind to group allocation"
Incomplete outcome data (attrition bias)	High risk	9.9% of intervention participants were lost to follow-up, and 18.5% of control participants were lost to follow-up. Reasons for missing data differed between groups.
Selective reporting (reporting bias)	Unclear risk	Study did not reference trial protocol

Sahlen 2006

**Methods**	**Place of recruitment**: Nordmaling residents; method of solicitation not reported.<br>**Inclusion criteria**: 75+, living independently without any home help or home-nursing care
**Participants**	**Number randomised**: 248 (Intervention)/ 346 (Control)<br>**Mean age**: 79.4<br>**Sex (% female)**: 55.1%<br>**Mortality per year in the Comparison Group**: 11.8%<br>**% Living Alone:** Not reported
**Interventions**	**Visitors' professional group**: Registered nurse; care visitor<br>**Frequency and duration of visits**: 4 visits over 2 years<br>**Fall Prevention:** Yes<br>**Included exercise**: No<br>**Included Multidimensional Geriatric Assessment:** Yes<br>**Description of intervention:**<br>The nurse and care visitor each received half of the participants in the intervention group. They shared knowledge with each other, as they had different professional backgrounds. Visits lasted 1.5-3 hours and followed structured programme on general health information and risks for falling, with each visit focusing on a different topic. Visitors used a structured questionnaire as an interview guide and evaluation tool. Visitors followed-up with needed services before the next home visit.<br>**Description of comparison**: Usual care<br>**Implementation (fidelity and participant compliance)**:<br>78.7% (196/249) of randomised participants that received intervention
**Outcomes**	**Mortality:** Mortality (24, 58 months)
**Notes**	**Location**: Nordmaling, Sweden<br>**Funding Source**: Swedish National Institute of Public Health<br>**Other notes**:

Risk of bias table

Bias	Authors' judgement	Support for judgement
Random sequence generation (selection bias)	High risk	Used birth date for randomisation. Quote: "those whose birth date was divisible by 3 were assigned to the intervention."
Allocation concealment (selection bias)	High risk	Used birth date for randomisation. Quote: "those whose birth date was divisible by 3 were assigned to the intervention."
Blinding (performance bias and detection bias)	Low risk	Quote: "all mortality data came from the Swedish national registration."
Incomplete outcome data (attrition bias)	High risk	Excluded randomised participants from analysis that did not receive the intervention (used n = 196 for intervention group).
Selective reporting (reporting bias)	High risk	Do not discuss outcomes found from interview questionnaire. Quote: "the questionnaire functioned as an interview guide as well as an evaluation instrument."

Shapiro 2002

**Methods**	**Place of recruitment**: State of Florida's Community Care for Elderly programme; solicited by telephone call from case managers<br>**Inclusion criteria**: Older adults on waiting list to receive social services and considered "moderate risk"
**Participants**	**Number randomised**: 40 (Intervention) / 65 (Comparison)<br>**Mean age**: 77.3<br>**Sex (% female)**: 80.0%<br>**Mortality per year in the comparison group**: 4.1%<br>**Living alone (%):** Not reported
**Interventions**	**Visitors' professional group**: Registered nurse<br>**Frequency and duration of visits**: 1 initial visit and then varied follow-up visits over 18 months<br>**Fall prevention:** No<br>**Included exercise**: No<br>**Included multidimensional geriatric assessment:** Yes<br>**Description of intervention:** Visits involved in-home geriatric assessment, with tailored services provided to address specific needs and problems. Services included homemaking, home delivered meals, help with chores, emergency alert response system, personal care, consumable medical supplies, medical transportation, and respite.<br>**Description of comparison**: Wait-list control<br>**Implementation (fidelity and participant compliance)**: 93.0% of intervention participants received the intervention.
**Outcomes**	**Health Related QoL:** Life Satisfaction (18 months)<br>**Institutionalisation People:** Total number of people admitted to nursing home (18 months)<br>**Mortality:** Total number of deaths (18 months)<br>**Psychiatric Anxiety and Depression:** Center for Epidemiologic Studies Depression Scale (CES - D) (18 months)
**Notes**	**Location**: Florida, U.S.A.<br>**Funding Source**: Borchard Center Foundation on Law and Aging, the United Way of Northeast Florida, and Baptist and St. Vincent's Hospitals<br>**Other notes**: Estimated cost of intervention was US$2300 per participant

Risk of bias table

**Bias**	**Authors' judgement**	**Support for judgement**
Random sequence generation (selection bias)	High risk	Quote: "randomly assigned using a random number generator" and "three members of the intervention group were removed from the study after baseline ... and were replaced by three members of the comparison group"
Allocation concealment (selection bias)	High risk	Quote: "three members of the intervention group were removed from the study after baseline ... and were replaced by three members of the comparison group"
Blinding (performance bias and detection bias)	High risk	Same unblinded case manager providing the intervention made the follow-up assessments at each of seven data points
Incomplete outcome data (attrition bias)	High risk	67.7% of control participants withdrew, with significantly more withdrawals due to institutionalisation and refusal to continue programme
Selective reporting (reporting bias)	Unclear risk	Study did not reference trial protocol

Sommers 2000

**Methods**	**Place of recruitment**: Patients from 18 primary care physician registers; solicited by mailed baseline questionnaire and cover letter.<br>**Inclusion criteria**: aged 65+, one or more visits to primary care physician in previous 3 months, did not require 24 hour care, low daily functioning, under treatment for at least 2 chronic conditions, spoke proficient English, living independently, not under therapy for cognitive decline
**Participants**	**Number randomised**: 383 (Intervention) / 351 (Comparison)<br>**Mean age**: 77.5<br>**Sex (% female)**: 68.5%<br>**Mortality per year in the comparison group**: 3.7%<br>**Living alone (%):** 48.6%
**Interventions**	**Visitors' professional group**: Registered nurse, social worker<br>**Frequency and duration of visits**: 1 initial visit and then varied follow-up visits over 18 months<br>**Fall prevention:** No<br>**Included exercise**: No<br>**Included multidimensional geriatric assessment:** Yes<br>**Description of intervention:** Visitors listened to health concerns, took vital signs and health histories, and completed a functional assessment and home safety check. Visitors then generated frailty and health risk scores, and drafted a risk reduction plan to set target objectives and plan self management strategies. Visitors also monitored health status between office visits every six weeks and revised care plan on basis of feedback.<br>**Description of comparison**: Usual care<br>**Implementation (fidelity and participant compliance)**: Intervention participants averaged 34 nurse or social contacts (range 1 – 176). 7% of intervention participants received less than 10 contacts, 47% received 10-29 contacts, and 46% received 30 to 176 contacts. Contacts lasted an average of 22 minutes per intervention participant and occurred every 21 days.
**Outcomes**	**A&E People**: Total number of people admitted to an A&E department (12, 24 months)<br>**Functioning ADL/IADL:** Health Activities Questionnaire (24 months)<br>**Health Related QoL:** Medical Outcomes Study Short Form Health Survey-36 (24 months); Symptom Scale (24 months)<br>**Hospitalisation Admissions:** Total number of hospital admissions (12, 124 months)<br>**Institutionalisation People:** Total number of people admitted to nursing home (12, 24 months)<br>**Mortality:** Total number of deaths (24 months)<br>**Psychiatric Anxiety and Depression:** Geriatric Depression Scale (24 months)
**Notes**	**Location**: San Francisco, California, U.S.A.<br>**Funding Source**: John A Hartford Foundation.<br>**Other notes**:

Risk of bias table

Bias	Authors' judgement	Support for judgement
Random sequence generation (selection bias)	Low risk	Quote: "the 13 internists and 5 family physicians were randomized, by means of a random number table"
Allocation concealment (selection bias)	Unclear risk	Quote: "the 13 internists and 5 family physicians were randomized, by means of a random number table"
Blinding (performance bias and detection bias)	Low risk	Quote: "Data were available for the assessments. . .from both HCFA and the Medicare HMOs"
Incomplete outcome data (attrition bias)	Unclear risk	30% of control partcipants and 28% of intervention participants were lost to follow-up. Methods of imputation were unclear
Selective reporting (reporting bias)	Unclear risk	Study did not reference trial protocol

Sorensen 1988

**Methods**	**Place of recruitment**: Central National Register for the general population of Copenhagen; solicited by visit from a social worker and physician<br>**Inclusion criteria**: aged 75+, registered in the Copenhagen Central National Register
**Participants**	**Number randomised**: 778 (Intervention) / 777 (Comparison)<br>**Mean age**: 79.8<br>**Sex (% female)**: 48.2%<br>**Mortality per year in the comparison group**: Not reported<br>**Living alone (%):** Not reported
**Interventions**	**Visitors' professional group**: Physician, social worker<br>**Frequency and duration of visits**: 1 initial 2-hour visit over 1 year<br>**Fall prevention:** No<br>**Included exercise**: No<br>**Included multidimensional geriatric assessment:** Yes<br>**Description of intervention:** Physician carried out general medical examination to establish need for health intervention. Social worker asked questions about housing conditions, economy, social support, and social network. If any unmet social needs were disclosed, social services were informed to request support. Participants' general practitioners were informed via mail of the social and medical conclusions made at the visit.<br>**Description of comparison**: Usual care<br>**Implementation (fidelity and participant compliance)**: Not reported
**Outcomes**	**Falls People:** Total number of people who experienced a fall (30 months)<br>**Health Related QoL:** Subjective health (30 months)<br>**Hospitalisation People:** Total number of people hospitalised (30 months)<br>**Institutionalisation People:** Total number of people admitted to nursing home (30 months)
**Notes**	**Location**: Copenhagen, Denmark<br>**Funding Source**: The Danish Health Insurance Foundation<br>**Other notes**:

Risk of bias table

Bias	Authors' judgement	Support for judgement
Random sequence generation (selection bias)	Unclear risk	Quote: "stratified random sampling among 75-, 80- and 85-year-old citizens"
Allocation concealment (selection bias)	Unclear risk	Quote: "stratified random sampling among 75-, 80- and 85-year-old citizens"
Blinding (performance bias and detection bias)	Low risk	The researchers obtained primary health outcome data from hospital registers.
Incomplete outcome data (attrition bias)	High risk	The authors did not report data for N = 190 participants who were assigned to the intervention but did not receive it.
Selective reporting (reporting bias)	High risk	Authors did not adequately report all outcomes (e.g., death) to allow for meta-analysis

Stevens 2001

**Methods**	**Place of recruitment**: State electoral roll; solicited by mail and telephone<br>**Inclusion criteria**: cognitively intact, able to speak English, didn’t plan to move, able to modify their homes, had no ramps or grab rails
**Participants**	**Number randomised**: 635 (Intervention) / 1244 (Comparison)<br>**Mean age**: 76.9<br>**Sex (% female)**: 52.3%<br>**Mortality per year in the comparison group**: Not reported<br>**Living alone (%):** Not reported
**Interventions**	**Visitors' professional group**: Research nurse<br>**Frequency and duration of visits**: 1 visit<br>**Fall prevention:** Yes<br>**Included exercise**: No<br>**Included multidimensional geriatric assessment:** No<br>**Description of intervention:** Before the visit, participants received information about the intervention and fall reduction strategies. Visits began with an educational session about recognising a fall. Then, visitors offered participants a home hazard assessment, installed safety devices, and provided more education about fall hazards.<br>**Description of comparison**: Attention-matched control<br>**Implementation (fidelity and participant compliance)**: 89.8% of intervention participants received the intervention.
**Outcomes**	**A&E Number:** Number of fall-related visits to an A&E department per person (12 months)<br>**Falls Number:** Number of falls per person (12 months)<br>**Falls People:** Total number of people who experienced a fall (12 months)<br>**Injuries Number:** Number of injurious falls per person (12 months)
**Notes**	**Location**: Perth metropolitan area, Western Australia<br>**Funding Source**: Western Australian Health Promotion Federation, Lotteries Commission of Western Australia, and Health Department of Western Australia<br>**Other notes**:

Risk of bias table

**Bias**	**Authors' judgement**	**Support for judgement**
Random sequence generation (selection bias)	Unclear risk	Quote: "index recruits were allocated in a 2:1 ratio to either the control (C) or the intervention (I) group"
Allocation concealment (selection bias)	Low risk	Quote: "Recruitment and allocation were by different recruitment officers, who were unaware of each others' activities, and therefore the allocation of subjects was concealed before their agreement to participate and determining their experimental status"
Blinding (performance bias and detection bias)	Unclear risk	Questionnaires were mailed to and filled out by participants. No information was provided on blinding status of researchers entering data
Incomplete outcome data (attrition bias)	Low risk	Quote: "All questionnaires were returned and less than 10% of data was missing"
Selective reporting (reporting bias)	Unclear risk	Study did not reference trial protocol

Stuck 1995

**Methods**	**Place of recruitment**: Voter-registration list; solicited by phone, mail, and personal contact.<br>**Inclusion criteria**: aged 75+, living at home<br>Excluded if: severe cognitive impairment, language problems, plans to move to a nursing home, plans to move away, self-reported terminal disease, participation in another randomised trial, and severe functional impairment
**Participants**	**Number randomised**: 215 (Intervention) / 199 (Comparison)<br>**Mean age**: 81.2<br>**Sex (% female)**: 70.0%<br>**Mortality per year in the comparison group**: 4.4%<br>**Living alone (%):** 64.0%
**Interventions**	**Visitors' professional group**: Gerontologic nurse practitioner<br>**Frequency and duration of visits**: 10.9 visits on average over 3 years<br>**Fall prevention:** No<br>**Included exercise**: No<br>**Included multidimensional geriatric assessment:** Yes<br>**Description of intervention:** Visits involved in-home asssessment of medical history, physical and mental health status, medications, quality of social support, home hazards, and other falls risk factors. Practitioners discussed each case with geriatricians, developed rank-order recommendations, and conducted in-home visits every 3 months to monitor implementation of recommendations, make additional recommendations, and facilitate compliance. Telephone contact was also available.<br>**Description of comparison**: Usual care<br>**Implementation (fidelity and participant compliance)**: 6.0% of intervention participants were never seen. Of the remaining 202 intervention participants, 24.3% did not complete the programme. Each year, intervention participants received an average of 5.9 recommendations about self-care, 3.3 recommendations to discuss new problems and treatement with physicians, and 2.3 recommendations about community services. Intervention participants adhered to 47.0% of all recommendations, did not adhere to 39.0%, and partly adhered to 14.0%.
**Outcomes**	**Functioning ADL/IADL:** Activities of Daily Living Composite (36 months); Instrumental Activities of Daily Living Composite (36 months)<br>**Hospitalisation Days:** Number of days in hospital per person (36 months)<br>**Hospitalisation People:** Total number of people hospitalised (12, 36 months)<br>**Institutionalisation Days:** Number of days in a nursing home per person (36 months)<br>**Institutionalisation People:** Total number of people admitted to nursing home (36 months)<br>**Mortality:** Total number of deaths (36 months)
**Notes**	**Location**: Santa Monica, California, U.S.A.<br>**Funding Source**: W.K. Kellogg Foundation, Swiss National Science Foundation, Senior Health and Peer Counseling, and the Swiss Foundation for Biological and Medical Grants<br>**Other notes**:

Risk of bias table

**Bias**	**Authors' judgement**	**Support for judgement**
Random sequence generation (selection bias)	Unclear risk	Quote: "randomization was performed with sealed envelopes containing random numbers"

Allocation concealment (selection bias)	Low risk	Quote: "randomization was performed with sealed envelopes containing random numbers"
Blinding (performance bias and detection bias)	Low risk	Quote: "trained interviewers not involved in the intervention who used a structured interview format"<br>Comment: Probably done, because outcome data was collected from hospital records or during interviews conducted by independent interviewers
Incomplete outcome data (attrition bias)	Low risk	Reasons for missing data were balanced between groups
Selective reporting (reporting bias)	Unclear risk	Study did not reference trial protocol

Stuck 2000

**Methods**	**Place of recruitment**: Health insurance list of community-residing subjects; method of solicitation not reported.<br>**Inclusion criteria**: aged 75+; living in 3 eligible zip code areas in Bern<br>Excluded if: living in a nursing home or board and care facility, did not speak German, had a terminal disease
**Participants**	**Number randomised**: 264 (Intervention) / 527 (Comparison)<br>**Mean age**: 81.7<br>**Sex (% female)**: 73.1%<br>**Mortality per year in the comparison group**: 4.2%<br>**Living alone (%):** 55.0%
**Interventions**	**Visitors' professional group**: Certified registered nurses with an additional degree in public health nursing<br>**Frequency and duration of visits**: 8 visits over 24 months<br>**Fall prevention:** No<br>**Included exercise**: No<br>**Included multidimensional geriatric assessment:** Yes<br>**Description of intervention:** Visits involved multidimensional geriatric assessments in participant homes. Nurses prepared a falls problem list, discussed it with the project geriatrician, developed recommendations, and conducted follow-up visits with participants every 3 months to encourage/monitor compliance and provide education. If medical problems were urgent, the project geriatrician could call participants' physicians. A physical therapist, occupational therapist, and social worker discussed complex psychosocail problems.<br>**Description of comparison**: Usual care<br>**Implementation (fidelity and participant compliance)**: 2.7% of intervention participants refused visits. Of the remaining 249 intervention participants, each participants received an average of 8.5 visits during the 24 months of the programme. Greater than 90% of intervention participants' primary care general practitioners were contacted during the intervention by a project geriatrician.
**Outcomes**	**Functioning ADL/IADL:** Total number of people dependent in basic ADL (36 months); total number of people dependent in basic IADL (36 months)<br>**Institutionalisation People:** Total number of people admitted to nursing home (36 months)<br>**Mortality:** Total number of deaths (36 months)
**Notes**	**Location**: Bern, Switzerland<br>**Funding Source**: Swiss National Science Foundation, Cantonal Department of Health and Social Affairs, W.K. Kellogg Foundation, Novartis Foundation for Gerontological Research, and Visana Health Insurance Company<br>**Other notes**: Estimated cost of intervention was US$276 per participant

Risk of bias table

Bias	Authors' judgement	Support for judgement
Random sequence generation (selection bias)	Low risk	Quote: "an independent test center calculated the treatment assignment based on a random letter table and informed the project team"
Allocation concealment (selection bias)	Low risk	Quote: "an independent test center calculated the treatment assignment based on a random letter table and informed the project team"
Blinding (performance bias and detection bias)	Low risk	Quote: "trained telephone interviewers independent of the intervention and blinded to the treatment assignment of study subjects collected information on ADL at 3 years"
Incomplete outcome data (attrition bias)	Unclear risk	Quote: "All analyses were by intention to treat, including subjects who refused intervention."<br>17.8% of intervention participants and 12.9% of control participants did not provide ADL/IADL data. Intention-to-treat procedure was not reported
Selective reporting (reporting bias)	Unclear risk	Study did not reference trial protocol number

Thomas 2007

**Methods**	**Place of recruitment**: Households in eastern Newfoundland phone book; solicitation by random-digit dialing of all households<br>**Inclusion criteria**: aged 75+, participant and caregiver both mentally competent, not receiving formal home care, able to identify an informal caregiver, English-speaking
**Participants**	**Number randomised**: 175 (Intervention Group 1), 170 (Intervention Group 2) / 175 (Comparison)<br>**Mean age**: 80.6<br>**Sex (% female)**: 67.5%<br>**Mortality per year in the comparison group**: 7.0%<br>**Living alone (%):** 46.3%
**Interventions**	**Visitors' professional group**: Nurse<br>**Frequency and duration of visits**: 4 visits over 48 months<br>**Fall prevention**: No<br>**Included exercise**: No<br>**Included multidimensional geriatric assessment:** Yes<br>**Description of intervention:**<br>Group 1: Visits involved a comprehensive assessment. Visitors shared results with participants and their caregivers.<br>Group 2: Same as Group 1, except that visitors also referred participants to health and social services.<br>**Description of comparison**: Usual care<br>**Implementation (fidelity and participant compliance)**: 100% of intervention participants received the intervention.
**Outcomes**	**Functioning, Cognitive:** Mini-Mental State Examination (12, 24, 36, 48 months)<br>**Health Related QoL:** Household Survey of Canada's National Health Population Survey (12, 24, 36, 48 months)<br>**Institutionalisation People:** Total number of people admitted to nursing home (48 months)<br>**Mortality:** Total number of deaths (48 months)
**Notes**	**Location**: Newfoundland, Canada<br>**Funding Source**: Not reported<br>**Other notes**:

Risk of bias table

**Bias**	**Authors' judgement**	**Support for judgement**
Random sequence generation (selection bias)	Low risk	Quote: "computer generated random numbers"
Allocation concealment (selection bias)	Low risk	Quote: "allocation was concealed from the RNs until they began working with individual clients"
Blinding (performance bias and detection bias)	High risk	Quote: "the RNs administering the intervention also measured outcomes; they were not blinded"
Incomplete outcome data (attrition bias)	Low risk	The authors analysed data for all randomised participants
Selective reporting (reporting bias)	Unclear risk	Quote: "Trial registration: Canadian Institutes of Health Research # 10576"<br>Comment: CIHR utilises the ISRCTN Register. Multiple searches of the registration number and protect keywords did not locate protocol.

Tinetti 1994

**Methods**	**Place of recruitment**: Health Maintenance Organization in Southern Connecticut; solicited by in-person contact at physicians' practices.<br>**Inclusion criteria**: aged 70+, ambulatory, had at least 1 risk factor for falling, not demented, no participation in vigorous sports or walking, not enrolled in another study of ageing
**Participants**	**Number randomised**: 153 (Intervention) / 148 (Comparison)<br>**Mean age**: 77.9<br>**Sex (% female)**: 69.1%<br>**Mortality per year in the comparison group**: 3.4%<br>**Living alone (%):** Not reported
**Interventions**	**Visitors' professional group**: Initial visits by nurse practitioner, then physical therapist<br>**Frequency and duration of visits**: 7.8 visits on average over 3 months<br>**Fall prevention:** Yes<br>**Included exercise**: Yes<br>**Included multidimensional geriatric assessment:** Yes<br>**Description of intervention:** Visitors instructed participants to perform strength exercises twice a day for 15-20 minutes per session. Visits also included behavioural modification instructions and medication adjustment later discussed with a general practitioner.<br>**Description of comparison**: Attention-matched control<br>**Implementation (fidelity and participant compliance)**: 94.8% of intervention participants received the intervention. Intervention participants reported that they completed 73.0% of the recommended exercise sessions.
**Outcomes**	**A&E Number:** Number of fall-related visits to an A&E department per person (12 months)<br>**A&E People**: Total number of people admitted to an A&E department due to fall (12 months)<br>**Falls Number:** Number of falls per person (12 months)<br>**Falls People:** Total number of people who experienced a fall (3, 6, 9, 12 months)<br>**Falls Subjective:** Falls self-efficacy (12 months)<br>**Hospitalisation Admissions:** Total number of hospital admissions (12 months)<br>**Hospitalisation Days:** Total number of days in hospital (12 months)<br>**Hospitalisation People:** Total number of people hospitalised (12 months)<br>**Injuries Number**: Total number of fall-related injuries (12 moths)<br>**Injuries People:** Total number of people with a serious injury due to a fall (12 months)<br>**Mortality:** Total number of deaths (12 months)
**Notes**	**Location**: Southern Connecticut, U.S.A.<br>**Funding Source**: National Institute on Aging<br>**Other notes**: Estimated cost of intervention was US$891 per participant

Risk of bias table

**Bias**	**Authors' judgement**	**Support for judgement**
Random sequence generation (selection bias)	Low risk	Quote: "random number generator on SAS software"
Allocation concealment (selection bias)	Low risk	Quote: "random number generator on SAS software"
Blinding (performance bias and detection bias)	Low risk	Quote: "the subjects were contacted by an interviewer who was blinded to their group assignments"
Incomplete outcome data (attrition bias)	Low risk	Reasons for missing data were balanced between groups
Selective reporting (reporting bias)	High risk	Data for some pre-specified measures were not reported

van Haastregt 2000

**Methods**	**Place of recruitment**: General Practices; method of solicitation not reported<br>**Inclusion criteria**: aged 70+, ambulatory, moderate impairments in mobility or a recent history of falls.<br>Excluded if: fully dependent on a wheelchair, on waiting list for a nursing home, or already receiving regular visits from health professional
**Participants**	**Number randomised**: 159 (Intervention) / 157 (Comparison)<br>**Mean age**: 77.2<br>**Sex (% female)**: 66.1%<br>**Mortality per year in the comparison group**: 5.9%<br>**Living alone (%):** 50.0%
**Interventions**	**Visitors' professional group**: Community nurse<br>**Frequency and duration of visits**: 5 visits over 12 months<br>**Fall prevention:** Yes<br>**Included exercise**: No<br>**Included multidimensional geriatric assessment:** Yes<br>**Description of intervention:** Visits included screening for medical, environmental, and behavioural risks for falls. Nurses followed-up the assessment with advice, referrals, and other actions aimed at reducing falls hazards. Nurses also assessed social and physical functioning, and completed a checklist for home safety.<br>**Description of comparison**: Usual care<br>**Implementation (fidelity and participant compliance)**: 86.8% of intervention participants received the intervention. Intervention participants complied with 46.0% of the advice given by nurses.
**Outcomes**	**A&E Number:** Number of fall-related visits to an A&E department per person (12, 18 months)<br>**Falls People:** Total number of people who experienced a fall (12, 18 months)<br>**Falls Subjective:** Fear of falling (12, 18 months)<br>**Functioning ADL/IADL:** Mobility Control (12, 18 months); Frenchay Activities Index (12, 18 months)<br>**Health Related QoL:** Medical Outcomes Study Short Form Health Survey-36 items for Mental Health (12, 18 months) and Physical Health (12, 18 months)<br>**Injuries Number:** Total number of injurious falls (12, 18 months)<br>**Mortality:** Total number of deaths (18 months)
**Notes**	**Location**: Hoensbroek, the Netherlands<br>**Funding Source**: Zorg Onderzoek Nederland and Stichting Onderzoek en Ontwikkeling Maatchappelijke Gezondheidzorg<br>**Other notes**:

Risk of bias table

Bias	Authors' judgement	Support for judgement
Random sequence generation (selection bias)	Low risk	Quote: "eligible patients were randomised to the home visit group or usual care group by computer generated random numbers directly after screening"
Allocation concealment (selection bias)	Low risk	Quote: "eligible patients were randomised to the home visit group or usual care group by computer generated random numbers directly after screening"
Blinding (performance bias and detection bias)	Unclear risk	Questionnaires were mailed to and filled out by participants. No information was provided on blinding status of researchers entering data
Incomplete outcome data (attrition bias)	Low risk	Reasons for missing data were balanced between groups
Selective reporting (reporting bias)	Unclear risk	Study did not reference trial protocol

van Hout 2010

**Methods**	**Place of recruitment**: Primary care practices; solicited by mail<br>**Inclusion criteria**: aged 75+, self-reported frailty<br>Excluded if: terminally ill, demented, participating in another research project
**Participants**	**Number randomised**: 331 (Intervention) / 320 (Comparison)<br>**Mean age**: 81.4<br>**Sex (% female)**: 70.5%<br>**Mortality per year in the comparison group**: 6.5%<br>**Living alone (%):** 54.8%
**Interventions**	**Visitors' professional group**: Nurse<br>**Frequency and duration of visits**: Minimum of 4 visits over 12 months<br>**Fall prevention:** Yes<br>**Included exercise**: Yes<br>**Included multidimensional geriatric assessment:** Yes<br>**Description of intervention:** Visits involved assessement of care needs, identification of health risks, determination of care priorities, and design and execution of individually tailored interventions. Follow-ups by telephone and additional home visits were provided as needed.<br>**Description of comparison**: Usual care<br>**Implementation (fidelity and participant compliance)**:<br>90.0% of intervention participants received the intervention. 67.1% of intervention participants met the protocol requirement of four or more visits.
**Outcomes**	**A&E People:** Total number of people admitted to an A&E department at least once (18 months)<br>**Functioning ADL/IADL:** Groningen Activity Restriction Scale (18 months)<br>**Health Related QoL:** Medical Outcomes Study Short Form Health Survey-36 items for Mental Health (6, 18 months) and Physical Health (6, 18 months)<br>**Hospitalisation People:**<br>Total number of people admitted to hospital (18 months)<br>**Institutionalisation People:** Total number of people admitted to nursing home (18 months)<br>**Mortality:** Total number of deaths (18 months)
**Notes**	**Location**: Netherlands<br>**Funding Source**: Netherlands Organization for Health Research<br>**Other notes**:

Risk of bias table

Bias	Authors' judgement	Support for judgement
Random sequence generation (selection bias)	Low risk	Quote: "we used Pocock's random number table and assigned up to 10 blocks per practice"
Allocation concealment (selection bias)	Low risk	Quote: "An independent statistician kept the assignment lists and assigned individuals to the intervention or control groups"
Blinding (performance bias and detection bias)	Unclear risk	Questionnaires were mailed to and filled out by participants. No information was provided on blinding status of researchers entering data
Incomplete outcome data (attrition bias)	Unclear risk	Quote: "Both "intention-to-treat" (ITT) and per protocol (PP) analyses were performed"<br>34.7% of intervention participants and 35.0% of control participants lost to follow-up. Intention-to-treat procedure was unclear
Selective reporting (reporting bias)	Unclear risk	Study did not reference trial protocol

van Rossum 1993

**Methods**	**Place of recruitment**: residents in regional area of Weert; solicited by postal questionnaire<br>**Inclusion criteria**: aged 75-84.<br>Excluded if: receiving regular home nursing care
**Participants**	**Number randomised**: 292 (Intervention) / 288 (Comparison)<br>**Mean age**: 78.7<br>**Sex (% female)**: 57.8%<br>**Mortality per year in the comparison group**: 5.8%<br>**Living alone (%):** 39.0%
**Interventions**	**Visitors' professional group**: Public health nurses<br>**Frequency and duration of visits**: Minimum of 4 visits per year over 3 years<br>**Fall prevention:** No<br>**Included exercise**: No<br>**Included multidimensional geriatric assessment:** Yes<br>**Description of intervention:** Nurses used a standardised checklist containing questions about functional state, medication, social contacts, and housing conditions. Participants could also phone the nurse to ask questions or ask for a visit. Visits focused on improving quality of life and included discussion of health topics and referrals as needed.<br>**Description of comparison**: Usual care<br>**Implementation (fidelity and participant compliance)**: 97.6% of intervention participants received the intervention. 2.4% of intervention participants refused visits. 32.9% of intervention participants received a total of 174 extra visits. 52.7% of intervention participants were advised to contact other services.
**Outcomes**	**Functioning ADL/IADL:** Activities of Daily Living Disabilities (36 months); Household disabilities (36 months)<br>**Health Related QoL:** Self-rated health (18, 36 months)<br>**Hospitalisation Admissions:** Total number of hospital admissions (12, 24, 36 months)<br>**Hospitalisation Days:** Total number of days in hospital (12, 24, 36 months)<br>**Hospitalisation People:**<br>Total number of people hospitalised (12, 24, 36 months)<br>**Institutionalisation Days:** Total number of days in a nursing home (36 months)<br>**Institutionalisation People:** Total number of people admitted to nursing home (36 months)<br>**Mortality:** Total number of deaths (12, 24, 36 months)
**Notes**	**Location**: Weert, Netherlands<br>**Funding Source**: Not reported<br>**Other notes**:

Risk of bias table

**Bias**	**Authors' judgement**	**Support for judgement**
Random sequence generation (selection bias)	Low risk	Quote: "randomisation was performed within each stratum by means of random numbers generated by computer"
Allocation concealment (selection bias)	Low risk	Quote: "randomisation was performed within each stratum by means of random numbers generated by computer"
Blinding (performance bias and detection bias)	Low risk	Quote: "the interviews were conducted by trained interviewers, who were unaware of whether a participant had been regularly visited by a nurse or not"
Incomplete outcome data (attrition bias)	Low risk	Reasons for missing data were balanced between groups
Selective reporting (reporting bias)	Unclear risk	Study did not reference trial protocol

Vetter 1984a

**Methods**	**Place of recruitment**: General Practices; solicited by in-person contact at participants' homes<br>**Inclusion criteria**: born before 1909, living at home
**Participants**	**Number randomised**: 281 (Intervention) / 273 (Comparison)<br>**Mean age**: 77.5<br>**Sex (% female)**: Not reported<br>**Mortality per year in the comparison group**: 8.2%<br>**Living alone (%):** Not reported
**Interventions**	**Visitors' professional group**: Health visitor<br>**Frequency and duration of visits**: 1 visit per year over 2 years<br>**Fall prevention:** No<br>**Included exercise**: No<br>**Included multidimensional geriatric assessment:** Yes<br>**Description of intervention:** Visits involved an in-home, semi-structured interview regarding participants' physical, mental, and social characteristics. Visits also included health education and prevention, referrals to a range of services, and follow-up of those services. Participants completed a problems sheet and treatment procedure form at each interview.<br>**Description of comparison**: Usual care<br>**Implementation (fidelity and participant compliance)**: Intervention participants received a total of 528 visits
**Outcomes**	**Functioning ADL/IADL:** Townsend's disability scale (24 months)<br>**Health Related QoL:** Subjective view of life overall (24 months)<br>**Mortality:** Total number of deaths (24 months)<br>**Psychiatric Anxiety and Depression:** Anxiety (24 months)
**Notes**	**Location**: Powys, Wales<br>**Funding Source**: Welsh Office and the Department of Health and Social Security (Office of the Chief Scientist)<br>**Other notes**:

Risk of bias table

Bias	Authors' judgement	Support for judgement
Random sequence generation (selection bias)	Unclear risk	Quote: "The people in the study were then randomly allocated by household to a group receiving intervention and a control group."
Allocation concealment (selection bias)	Unclear risk	Quote: "randomly selected from the age-sex register"
Blinding (performance bias and detection bias)	Low risk	Quote: "interview was repeated by the independent interviewer after two years"
Incomplete outcome data (attrition bias)	Unclear risk	Reasons for missing data were not reported by group
Selective reporting (reporting bias)	Unclear risk	Study did not reference trial protocol

Vetter 1984b

**Methods**	**Place of recruitment**: General Practices; solicited by in-person contact at participants' homes<br>**Inclusion criteria**: born before 1909, living at home
**Participants**	**Number randomised**: 296 (Intervention) / 298 (Comparison)<br>**Mean age**: 76.8<br>**Sex (% female)**: Not reported<br>**Mortality per year in the comparison group**: 10.1%<br>**Living alone (%):** Not reported
**Interventions**	**Visitors' professional group**: Health visitor<br>**Frequency and duration of visits**: 1 visit per year over 2 years<br>**Fall prevention:** No<br>**Included exercise**: No<br>**Included multidimensional geriatric assessment:** Yes<br>**Description of intervention:** Visits involved an in-home, semi-structured interview regarding participants' physical, mental, and social characteristics. Visits also included health education and prevention, referrals to a range of services, and follow-up of those services. Participants completed a problems sheet and treatment procedure form at each interview.<br>**Description of comparison**: Usual care<br>**Implementation (fidelity and participant compliance)**: Intervention participants received a total of 528 visits.
**Outcomes**	**Functioning ADL/IADL:** Townsend's disability scale (24 months)<br>**Health Related QoL:** Subjective view of life overall (24 months)<br>**Mortality:** Total number of deaths (24 months)<br>**Psychiatric Anxiety and Depression:** Anxiety (24 months)
**Notes**	**Location**: Gwent, Wales<br>**Funding Source**: Welsh Office and the Department of Health and Social Security (Office of the Chief Scientist)<br>**Other notes**:

Risk of bias table

**Bias**	**Authors' judgement**	**Support for judgement**
Random sequence generation (selection bias)	Unclear risk	Quote: "The people in the study were then randomly allocated by household to a group receiving intervention and a control group."
Allocation concealment (selection bias)	Unclear risk	Quote: "randomly selected from the age-sex register"<br>Comment: The authors did not report any other information
Blinding (performance bias and detection bias)	Low risk	Quote: "interview was repeated by the independent interviewer after two years"
Incomplete outcome data (attrition bias)	Unclear risk	Reasons for missing data were not reported by group
Selective reporting (reporting bias)	Unclear risk	Study did not reference trial protocol

Vetter 1992

**Methods**	**Place of recruitment**: General Practice; solicited by in-person contact at participants' homes.<br>**Inclusion criteria**: aged 70+
**Participants**	**Number randomised**: 350 (Intervention) / 324 (Comparison)<br>**Mean age**: 76.8<br>**Sex (% female)**: Not reported<br>**Mortality per year in the comparison group**: 8.2%<br>**Living alone (%):** Not reported
**Interventions**	**Visitors' professional group**: Health visitor<br>**Frequency and duration of visits**: Minimum of 1 visit per year over 4 years<br>**Fall prevention:** Yes<br>**Included exercise**: Yes<br>**Included multidimensional geriatric assessment:** Yes<br>**Description of intervention:** Visitors obtained a history of illness and focused on four factors: nutrition, medical conditions, environment, and muscle tone. Local voluntary agencies were recruited to fix environmental hazards. A part-time physiotherapist helped with fitness classes for moderately disabled people. Visitors referred participants to other professionals as needed.<br>**Description of comparison**: Usual care<br>**Implementation (fidelity and participant compliance)**: Not reported
**Outcomes**	**A&E People**: Total number of people admitted to an A&E department due to fall (48 months)<br>**Falls People:** Total number of people who experienced a fall (48 months)<br>**Injuries People:** Total number of people who experienced a fracture due to a fall (48 months)<br>**Mortality:** Total number of deaths (48 months)
**Notes**	**Location**: Wales<br>**Funding Source**: Grand Charity and Welsh Office<br>**Other notes**:

Risk of bias table

Bias	Authors' judgement	Support for judgement
Random sequence generation (selection bias)	Low risk	Quote: "the randomisation was carried out ... using random number tables"
Allocation concealment (selection bias)	Unclear risk	Quote: "the randomisation was carried out ... using random number tables"
Blinding (performance bias and detection bias)	High risk	The outcome assessor was a research assistant not blinded to allocation
Incomplete outcome data (attrition bias)	Unclear risk	Reasons for missing data were not reported by group
Selective reporting (reporting bias)	Unclear risk	Study did not reference trial protocol

Wyman 2007

**Methods**	**Place of recruitment**: Health Care Financing Administration list of female Medicare beneficiaries; solicited by mailing a random sample of the list.<br>**Inclusion criteria**: aged 70+, female, Medicare beneficiary, living independently, postural instabilty, low cognitive impairment, at least 1 injurious fall factor, physician clearance for exercise, available for follow-up appointments, proficiency in English.<br>Excluded if: unable to walk 30 feet without stopping or assistive device, involved in regular exercise, unstable health conditions preventing safe, independent exercise, terminal illness.
**Participants**	**Number randomised**: 137 (Intervention) / 135 (Comparison)<br>**Mean age**: 78.8<br>**Sex (% female)**: 100.0%<br>**Mortality per year in the comparison group**: 0.0%<br>**Living alone (%):** 48.9%
**Interventions**	**Visitors' professional group**: Registered nurse<br>**Frequency and duration of visits**: 1 visit every other week over 12 weeks<br>**Fall prevention:** Yes<br>**Included exercise**: Yes<br>**Included multidimensional geriatric assessment:** No<br>**Description of intervention:** The intervention involved a multidimensional, 12-week programme alternating between home visits and phone calls. The programme included an exercise programme (Weeks 1-3), an individualised fall risk profile including environmental hazards (Week 5), fall and home safety education and evaluation (Weeks 5-13), two nightlights, individualised counseling, and referrals to other services as needed.<br>**Description of comparison**: Education about topics unrelated to falls prevention<br>**Implementation (fidelity and participant compliance)**: 91.9% of intervention participants received the full allocated intervention. 5.1% received a partial version of the allocated intervention. 97.0% of comparison participants received the comparison treatment. The intervention participants received an average of 6.6 home visits and 5.5 telephone calls. Comparison participants received a mean of 6.7 home visits and 5.4 telephone calls. 62.0% of intervention participants set at least one goal for correcting an environmental hazard, 7.3% refused to make an action plan to reduce their fall risk factors, and 34.3% actually made one or more home modifications by follow-up.
**Outcomes**	**Falls Number:** Total number of falls (12 months)<br>**Falls People:** Total number of people who experienced a fall (12 months)<br>**Mortality:** Total number of deaths (12 months)
**Notes**	**Location**: Twin Cities Metropolitan Area, Minnesota, U.S.A.<br>**Funding Source**: National Institute of Nursing Research and the Office of Research on Women's Health, National Institutes of Health<br>**Other notes**:

Risk of bias table

Bias	Authors' judgement	Support for judgement
Random sequence generation (selection bias)	Unclear risk	Quote: "participants were stratified according to age group ... and randomised using a permuted block design"
Allocation concealment (selection bias)	Unclear risk	Quote: "participants were stratified according to age group ... and randomised using a permuted block design"
Blinding (performance bias and detection bias)	Low risk	Quote: "The same nurse practitioner (MM) evaluated environmental hazards at baseline and immediately after a 12-week, home- based intervention. This individual was blinded to treatment group status, and study participants were reminded not to reveal their treatment assignment during their follow-up visit"
Incomplete outcome data (attrition bias)	Low risk	Study had less than 10.0% attrition, and reasons for missing data were similar between groups
Selective reporting (reporting bias)	Unclear risk	Study did not reference a trial protocol

Yamada 2003

**Methods**	**Place of recruitment**: Voter registration list of two geographic areas; solicited by mail<br>**Inclusion criteria**: aged 65+, community-dwelling, dependent in instrumental activities of daily living, independent in activities of daily living<br>Excluded if: dependent on mobility or personal care
**Participants**	**Number randomised**: 184 (Intervention) / 184 (Comparison)<br>**Mean age**: 78.7<br>**Sex (% female)**: 63.3%<br>**Mortality per year in the comparison group**: 5.4%<br>**Living alone (%):** 9.2%
**Interventions**	**Visitors' professional group**: Public health nurses<br>**Frequency and duration of visits**: 5.1 visits on average over 18 months<br>**Fall prevention:** Yes<br>**Included exercise**: No<br>**Included multidimensional geriatric assessment:** Yes<br>**Description of intervention:** Visits were human-interaction focused and involved flexible approach. Initial visits involved multidimensional geriatric assessments, which informed further assessments and treatment recommendations. Visits stopped if a participant was institutionalised and were restarted after hospitalisation.<br>**Description of comparison**: Usual care<br>**Implementation (fidelity and participant compliance)**: 100% of intervention participants received the intervention.
**Outcomes**	**Health Related QoL:** Euroqol-5 Dimensions Weighted Index (18 months)<br>**Institutionalisation People:** Total number of people in nursing home at end of study (18 months)<br>**Mortality:** Total number of deaths (18 months)
**Notes**	**Location**: Sapporo city and Takahata town, Japan<br>**Funding Source**: Ministry of Health and Welfare in Japan<br>**Other notes**:

Risk of bias table

**Bias**	**Authors' judgement**	**Support for judgement**
Random sequence generation (selection bias)	Low risk	Quote: "computerised central randomisation scheme generated by one of the research officers"
Allocation concealment (selection bias)	Low risk	Quote: "computerised central randomisation scheme generated by one of the research officers"
Blinding (performance bias and detection bias)	Unclear risk	Outcomes self-reported by participants. The authors did not report information on the blinding status of researchers entering data
Incomplete outcome data (attrition bias)	High risk	Significantly more control participants (6.0%) than intervention participants (0.5%) refused to respond to the final questionnaire
Selective reporting (reporting bias)	Unclear risk	Study did not reference trial protocol
