# Supplementary material for: Preventive Home Visits for Mortality, Morbidity, and Institutionalization in Older Adults: A Systematic Review and Meta-Analysis
Source: PLoS One. 2014 Mar 12;9(3):e89257. doi: 10.1371/journal.pone.0089257 (PMC3951196; doi:10.1371/journal.pone.0089257)
Supplement: Table S1 — Summary of findings table. (DOCX) [file pone.0089257.s007.docx]

# Table S1: Summary of findings table

| **Home Visits versus Controls for prevention of impairment and death in older adults** | | | | | |
| --- | --- | --- | --- | --- | --- |
| **Outcomes** | **Illustrative comparative risks* (95% CI)** | | **Relative effect (95% CI)** | **No of Participants (studies)** | **Quality of the evidence (GRADE)** |
|  | **Assumed risk** | **Corresponding risk** |  | |  |
|  | **Control** | **Home Visits versus Controls** |  | |  |
| Mortality | Study population | | RR 0.93  (0.87 to 0.99) | 24198  (55 studies) | ⊕⊕⊕⊕ high |
|  | 139 per 1000 | 130 per 1000 (121 to 138) |  | | |
|  | Low | |  |  |  |
|  | 50 per 1000 | 47 per 1000 (44 to 50) |  |  |  |
|  | High | |  |  |  |
|  | 200 per 1000 | 186 per 1000 (174 to 198) |  |  |  |
| Institutionalisation (people admitted) | Study population | | RR 1.02  (0.88 to 1.18) | 16459  (27 studies) | ⊕⊕⊕⊝ moderate ^a,b^ |
|  | 85 per 1000 | 87 per 1000 (75 to 100) |  | | |
|  | Low | |  |  |  |
|  | 40 per 1000 | 41 per 1000 (35 to 47) |  |  |  |
|  | High | |  |  |  |
|  | 120 per 1000 | 122 per 1000 (106 to 142) |  |  |  |
| Falls  (people who fell) | Low | | OR 0.86  (0.73 to 1.01) | 7455  (23 studies) | ⊕⊕⊕⊝ moderate ^c^ |
|  | 200 per 1000 | 177 per 1000 (154 to 202) |  | | |
|  | High | |  |  |  |
|  | 600 per 1000 | 563 per 1000 (523 to 602) |  |  |  |
| Hospitalisation  (people admitted) | Study population | | RR 0.96  (0.91 to 1.01) | 6288  (15 studies) | ⊕⊕⊕⊝ moderate^d^ |
|  | 410 per 1000 | 394 per 1000 (373 to 414) |  | | |
|  | Low | |  |  |  |
|  | 200 per 1000 | 192 per 1000 (182 to 202) |  |  |  |
|  | High | |  |  |  |
|  | 600 per 1000 | 576 per 1000 (546 to 606) |  |  |  |
| Functioning (ADL/IADL) | The mean functioning (ADL/IADL) in the intervention groups was 0.10 standard deviations better (0.17 to 0.03 better) | |  | 8769  (27 studies) | ⊕⊝⊝⊝ very low ^e,f,g^ |
| Health Related  Quality of Life | The mean health related quality of life in the intervention groups was 0.06 standard deviations better (0.11 to 0.01 better) | |  | 9892  (29 studies) | ⊕⊕⊝⊝ low^5, 7^ |
| *The basis for the assumed risk (e.g. the median control group risk across studies) is provided in footnotes. The corresponding risk (and its 95% confidence interval) is based on the assumed risk in the comparison group and the relative effect of the intervention (and its 95% CI). CI: Confidence interval; RR: Risk ratio; OR: Odds ratio; | | | | | |
| GRADE Working Group grades of evidence High quality: Further research is very unlikely to change our confidence in the estimate of effect.  Moderate quality: Further research is likely to have an important impact on our confidence in the estimate of effect and may change the estimate. Low quality: Further research is very likely to have an important impact on our confidence in the estimate of effect and is likely to change the estimate. Very low quality: We are very uncertain about the estimate. | | | | | |
| a. Institutionalisation people was recorded and reported in several different ways within and across studies b. This is a main outcome for this intervention and this review, but only 57% of participants and 42% of studies are included in this analysis. c. Heterogeneity was significant for this outcome (Chi²=43.59, df=22, p=0.004; I²=50%).  d. Trim and fill analysis imputed 6 studies and adjusted effect RR = 0.98 (0.91 - 1.06) e. Lack of blinding of outcome assessors was a significant risk of bias for this outcome. f. Heterogeneity was important and statistically significant for this outcome (Chi²=55.40, df=26, p=0.0007; I²=53%).  g. This is a main outcome for this intervention, yet only a minority of participants and studies were included in this analysis. We concluded there is  a high risk that the effect is overestimated as a result of selective outcome reporting. | | | | | |
