# Supplementary material for: Preventive Home Visits for Mortality, Morbidity, and Institutionalization in Older Adults: A Systematic Review and Meta-Analysis
Source: PLoS One. 2014 Mar 12;9(3):e89257. doi: 10.1371/journal.pone.0089257 (PMC3951196; doi:10.1371/journal.pone.0089257)
Supplement: Table S2 — Table of included studies. (DOCX) [file pone.0089257.s008.docx]

# Table S2: Table of included studies

| **Study ID** | **Recruitment** | **Country** | **No.^a^** | **Age (*SD*) ^b^** | **Primary Outcome ^c^** | **Visitor** | **Mean**  **Visits** | **Length (Months)** |
| --- | --- | --- | --- | --- | --- | --- | --- | --- |
| ***Falls prevention*** | | | | | | | | |
| Campbell 1999 [1,2] | N/R | NZ | 233 | 84 (*4.6*) | FN; InjN | Other | 4 | 12 |
| Campbell 2005 [3,4] | 2002 – 2003 | NZ | 194 | 84 (*4.8*) | *FN; *InjN | Combined | 6 | 12 |
| Chandler 1998 [5] | N/R | US | 100 | 78 (*7.6*) | GAD; QoL; PP | Other | 30 | 3 |
| Elley 2008 [6,7] | 2005 – 2006 | NZ | 312 | 81 (*5.0*) | *FN | Nurse | N/R | 12 |
| Gallagher 1996 [8] | N/R | CA | 100 | 75 | FN; FS; QoL; HSU; Soc | Nurse | 3 | 6 |
| Green 2002 [9] | 1995 – 1997 | UK | 170 | 73 (*11.5*) | Mobility | Other | 3 | 3 |
| Gustafsson 2012 [10,11] | 2007 –2008 | SE | 288 | 86; range 80–97 | *ADL; *Cog; *FS; *PP; *PSY | Combined | 1 | 1 |
| Hogan 2001 [12] | N/R | CA | 163 | 78 (*9.1*) | FN; FP | Combined | 2 | 12 |
| Kingston 2001 [13] | N/R | UK | 109 | 72 | QoL | Other | 1 | 12 |
| Krebs 1998 [14] | N/R | US | 120 | 74 (*6.5*) | PP | Other | 2 | 6 |
| Lin 2007 [15] | 2003 - 2004 | TW | 100 | 77 | QoL | Other | 8 | 4 |
| Liu-Ambrose 2008 [16] | 2004 –2006 | CA | 59 | 82 (*6.3*) | Falls Risks Factors | Other | 5 | 6 |
| Luukinen 2006 [17,18] | 2000 –2001 | FI | 486 | 88 (*4.2*) | FN; FP | Combined | 3 | 3 |
| Pighills 2011 [19] | 2006 –2006 | UK | 165 | 79 (*6.0*) | *FS | Other | 1 | 1 |
| Robertson 2001a [20,21] | 1998 | NZ | 240 | 81 (*5.8*) | FN | Nurse | 5 | 6 |
| Stevens 2001 [22,23] | 1995 | AU | 1879 | 77 | FN | Nurse | 1 | 1 |
| Wyman 2007 [24] | N/R | US | 272 | 79 (*5.6*) | Home Hazards | Nurse | 6 | 3 |
| **Multidimensional geriatric assessment** | | | | | | | | |
| Balaban 1988 [25] | 1981 – 1982 | US | 198 | 69; range 17–99 | ADL; HSU; QoL; PSY | Combined | N/R | N/R |
| Bernabei 1998 [26] | 1995 | IT | 200 | 81 (*10*.*0*) | ADL; Cog; HSU; InsP | Other | 6 | 12 |
| Bouman 2008 [27,28,29,30] | 2002 – 2003 | NL | 330 | 76 (*3.8*) | ADL; Cog; QoL; Soc | Nurse | 6.9 | 18 |
| Byles 2004 [31] | 1997 – 1998 | AU | 1569 | 77 | HA; InsP; QoL; Mort | Combined | 4.5 | 36 |
| Caplan 2004 [32] | N/R | AU | 739 | 82 (*8.1*) | HA | Nurse | 2.29 | 1 |
| Counsell 2007 [33,34,35] | 2002 – 2004 | US | 951 | 72 (*7.8*) | ADL; ER-N; QoL | Combined | 13 | 24 |
| Dalby 2000 [36] | N/R | CA | 142 | 79 (*5.6*) | Mort; InsP | Nurse | 3 | 14 |
| Gunner-Svensson 1984 [37] | 1972 | DK | 3743 | N/R | InsP | Nurse | N/R | 60 |
| Hall 1992 [38] | 1986 – 1987 | CA | 167 | 78 | Insp | Nurse | N/R | 36 |
| Hebert 2001 [39] | N/R | CA | 503 | 80 (*4.4*) | ADL | Nurse | 1 | 12 |
| Kono 2004 [40] | 2000 | JP | 119 | 83 | ADL; Cog; FS; PSY; Soc | Nurse | 4.3 | 18 |
| Kono 2011 [41,42] | 2007 – 2008 | JP | 323 | 80 (*6.3*) | ADL; InsP; Mort; PSY; Soc | Combined | 4 | 24 |
| Markle-Reid 2006 [43] | 2001 – 2002 | CA | 288 | 84 (*5.4*) | HSU; QoL; PSY; Soc | Nurse | 5 | 6 |
| McEwan 1990 [44] | 1986 | UK | 296 | N/R | ADL; HP; QoL; Mort; PP | Nurse | 1 | 20 |
| Pathy 1992 [45] | N/R | UK | 725 | 73 (*6.4*) | HA; HD; QoL; InsP; Mort | Other | 9 | N/R |
| Ploeg 2010 [46] | 2003 – 2005 | CA | 719 | 81 (*4.3*) | QoL | Nurse | 3.03 | 12 |
| Shapiro 2002 [47] | 1998 | US | 105 | 77 (*N/R*) | QoL | Nurse | N/R | 18 |
| **Study ID** | **Recruitment** | **Country** | **No.^a^** | **Age (*SD*) ^b^** | **Primary Outcome ^c^** | **Visitor** | **Mean**  **Visits** | **Length (Months)** |
| **Multidimensional geriatric assessment** | | | | | | | | |
| Sommers 2000 [48] | 1992 – 1993 | US | 734 | 76 (*6.7*) | ADL; ER-P; HA; QoL; HSU; InsP; Mort; PSY | Combined | 10+ | 18 |
| Sorensen 1988 [49] | 1978 – 1979 | DK | 1555 | 80 (*N/R*) | FP; QoL; HP; InsP | Combined | 1 | 12 |
| Stuck 1995 [50,51,52,53] | 1988 – 1990 | US | 414 | 81 (*4.1*) | ADL | Nurse | 10.9 | 36 |
| Stuck 2000 [54] | 1998 | CH | 791 | 82 (*4.6*) | ADL | Nurse | 8 | 24 |
| Thomas 2007 [55] | N/R | CA | 520 | 81 (*4.4*) | Cog; QoL; InsP; Mort | Nurse | 4 | 48 |
| van Rossum 1993 [56] | N/R | NL | 580 | 79 | ADL; QoL; HA; HD; HP; InsD; InsP; Mort | Nurse | 12 | 36 |
| Vetter 1984a [57] | N/R | UK | 554 | 78 | ADL; QoL; Mort; PSY | Other | 2 | 24 |
| Vetter 1984b [57] | N/R | UK | 594 | 77 | ADL; QoL; Mort; PSY | Other | 2 | 24 |
| ***Both falls prevention and* multidimensional geriatric assessment** | | | | | | | | |
| Ciaschini 2009 [58,59] | 2003 – 2005 | CA | 201 | 72 (*11.26*) | Falls risk assessment | Nurse | N/R | N/R |
| Close 1999 [60] | 1995 – 1996 | UK | 397 | 78 (*7.5*) | FN | Other | 1 | 1 |
| Davison 2005 [61] | 1998 – 1999 | UK | 313 | 77 (*7.0*) | FN; FP | Combined | N/R | N/R |
| Fabacher 1994 [62] | N/R | US | 254 | 73 (*5.8*) | ADL; FP; HP; InsD; InsP; Mort | Combined | 4 | 12 |
| Gitlin 2006 [63,64,65] | 2000 – 2003 | US | 319 | 79 (*5.9*) | ADL; FS | Combined | 6 | 12 |
| Hendriks 2008 [66] | 2003 – 2004 | NL | 333 | 75 (*6.4*) | ADL; FN | Combined | 1 | 1 |
| Huang 2004 [67] | N/R | TW | 120 | 72 (*5.7*) | FP; FS | Other | 2 | 4 |
| Lightbody 2002 [68] | 1997 | UK | 348 | 75: IQR 70–81 | ADL; ER-P; FN; FP; HA; HD; Mort | Nurse | 1 | 1 |
| Markle-Reid 2010 [69] | 2006 – 2007 | CA | 109 | N/R | *FN; *InjN | Combined | 6 | 6 |
| Newbury 2001 [70,71] | 1998 – 1999 | AU | 100 | 79; range 75–88 | Mort; “Problems” | Nurse | 1 | 6 |
| Sahlen 2006 [72] | 1999 | SE | 594 | 79 (*4.1*) | Mort | Combined | 4 | 24 |
| Tinetti 1994 [73,74] | 1990 – 1992 | US | 301 | 78 (*5.3*) | FN | Combined | 7.8 | 3 |
| van Haagstregt 2000 [75] | 1997 – 1997 | NL | 316 | 77 (*5.1*) | FP; InjP | Nurse | 5 | 12 |
| van Hout 2010 [76] | N/R | NL | 651 | 81 | ADL; QoL | Nurse | 4 | 12 |
| Vetter 1992 [77] | N/R | UK | 674 | 77 | InjN | Other | 4 | 48 |
| Yamada 2003 [78] | N/R | JP | 368 | 79 (*7.8*) | QoL | Nurse | 5.1 | 18 |
| ***Neither falls prevention nor* multidimensional geriatric assessment** | | | | | | | | |
| Ciechanowski 2004 [79] | 2000 – 2003 | US | 138 | 73 (*8.5*) | PSY; QoL | Other | 6.6 | 4 |
| Crawford-Shearer 2010 [80] | 2007 – 2008 | US | 59 | 78 (*9.3*) | QoL | Nurse | 6 | 3 |
| Holland 2005 [81,82,83] | 2000 – 2002 | UK | 872 | 85 (*4.0*) | ER-N | Other | 2 | 6 |
| Lenaghan 2007 [84] | N/R | UK | 134 | 84 | HA | Other | 2 | 2 |
| Luker 1981 [85] | N/R | UK | 120 | N/R | Health problems; Life satisfaction | Other | 4 | 4 |
| Nelson 2004 [86] | N/R | US | 72 | 78 (*5.3*) | ADL; QoL; Mort; PSY; PP | Other | 11 | 6 |

a. No. (number of participants in the study)

b. Age (mean age and standard deviation; range or inter-quartile range (IQR) is given if the standard deviation was not reported)

c. Primary Outcome (As reported by the authors. Prospectively registered outcomes are marked an asterisk.)

ADL = Physical functioning; Cog = Cognitive functioning; ER-N = ER, number; ER-P = ER, people; FN = Falls, number; FP = Falls, people; FS = Falls, subjective; HA = Hospital admissions; HD = Hospital days; HP = Hospitalised people; QoL = Health-Related quality of life; HSU = Health Service Use; InjN = Injuries, number; InjP = Injuries, people; InsD = Institutionalisation days; InsP = Institutionalised people; Mort = Mortality; PSY = Anxiety and depression; PP = Physical performance indicators; Soc = Social Functioning.

**Table S2 References**

1. Campbell AJ, Robertson MC, Gardner MM, Norton RN, Buchner DM (1999) Falls prevention over 2 years: A randomized controlled trial in women 80 years and older. Age Ageing 28: 513-518.

2. Campbell AJ, Robertson MC, Gardner MM, Norton RN, Tilyard MW, et al. (1997) Randomised controlled trial of a general practice programme of home based exercise to prevent falls in elderly women. BMJ 315: 1065-1069.

3. Campbell AJ, Robertson MC, La Grow SJ, et al. (2005) Randomised controlled trial of prevention of falls in people aged > or =75 with severe visual impairment: The VIP trial. BMJ 331: 817.

4. La Grow SJ, Robertson MC, Campbell AJ, Clarke GA, Kerse NM (2006) Reducing hazard related falls in people 75 years and older with significant visual impairment: How did a successful program work? Inj Prev 12: 296-301.

5. Chandler JM, Duncan PW, Kochersberger G, Studenski S (1998) Is lower extremity strength gain associated with improvement in physical performance and disability in frail, community-dwelling elders? Arch Phys Med Rehabil 79: 24-30.

6. Elley CR, Robertson MC, Garrett S, et al. (2008) Effectiveness of a falls-and-fracture nurse coordinator to reduce falls: A randomized, controlled trial of at-risk older adults. J Am Geriatr Soc 56: 1383-1389.

7. Elley CR, Robertson MC, Kerse NM, et al. (2007) Falls Assessment Clinical Trial (FACT): Design, interventions, recruitment strategies and participant characteristics. BMC Public Health 7: 185.

8. Gallagher E, Brunt H (1996) Head over heels: Impact of a health promotion program to reduce falls in the elderly. Can J Aging 15: 84-96.

9. Green J, Forster A, Bogle S, Young J (2002) Physiotherapy for patients with mobility problems more than l year after stroke: A randomised controlled trial. Lancet 359: 199-203.

10. Dahlin-Ivanoff S, Gosman--Hedström G, Edberg AK, et al. (2010) Elderly persons in the risk zone. Design of amultidimensional, health-promoting, randomised three-armed controlled trial for "prefrail" people of 80+ years living at home. BMC Geriatr 10: 27.

11. Gustafsson S, Wilhelmson K, Eklund K, et al. (2012) Health-promoting interventions for persons aged 80 and older are successful in the short term—Results from the randomized and three-armed elderly persons in the risk zone study. J Am Geriatr Soc 60: 447-454.

12. Hogan DB, MacDonald FA, Betts J, et al. (2001) A randomized controlled trial of a community-based consultation service to prevent falls. CMAJ 165: 537-543.

13. Kingston P, Jones M, Lally F, Crome P (2001) Older people and falls: A randomized controlled trial of a health visitor (HV) intervention. Rev Clin Gerontol 11: 209-214.

14. Krebs DE, Jette AM, Assmann SF (1998) Moderate exercise improves gait stability in disabled elders. Arch Phys Med Rehabil 79: 1489-1495.

15. Lin MR, Wolf SL, Hwang HF, Gong SY, Chen CY (2007) A randomized, controlled trial of fall prevention programs and quality of life in older fallers. J Am Geriatr Soc 55: 499-506.

16. Liu-Ambrose T, Donaldson MG, Ahamed Y, et al. (2008) Otago home-based strength and balance retraining improves executive functioning in older fallers: A randomized controlled trial. J Am Geriatr Soc 56: 1821-1830.

17. Luukinen H, Lehtola S, Jokelainen J, Väänänen-Sainio R, Lotvonen S, et al. (2006) Prevention of disability by exercise among the elderly: A population-based, randomized, controlled trial. Scand J Prim Health Care 24: 199-205.

18. Luukinen H, Lehtola S, Jokelainen J, Väänänen-Sainio R, Lotvonen S, et al. (2007) Pragmatic exercise-oriented prevention of falls among the elderly: A population-based, randomized, controlled trial. Preventive Medicine 44: 265-271.

19. Pighills AC, Torgerson DJ, Sheldon TA, Drummond AE, Bland JM (2011) Environmental assessment and modification to prevent falls in older people. J Am Geriatr Soc 59: 26-33.

20. Robertson MC, Devlin N, Gardner MM, Campbell AJ (2001) Effectiveness and economic evaluation of a nurse delivered home exercise programme to prevent falls. 1: Randomised controlled trial. BMJ 322: 697-701.

21. Robertson MC, Devlin N, Scuffham P, Gardner MM, Buchner DM, et al. (2001) Economic evaluation of a community based exercie programme to prevent falls. J Epidemiol Community Health 55: 600-606.

22. Stevens M, Holman CDA, Bennett N (2001) Preventing falls in older people: Impact of an intervention to reduce environmental hazards in the home. J Am Geriatr Soc 49: 1442-1447.

23. Stevens M, Holman CDA, Bennett N, de Klerk N (2001) Preventing falls in older people: Outcome evaluation of a randomized controlled trial. J Am Geriatr Soc 49: 1455.

24. Wyman JF, Croghan CF, Nachreiner NM, et al. (2007) Effectiveness of education and individualized counseling in reducing environmental hazards in the homes of community-dwelling older women. J Am Geriatr Soc 55: 1548-1556.

25. Balaban DJ, Goldfarb NI, Perkel RL, Carlson BL (1988) Follow-up study of an urban family medicine home visit program. J Fam Pract 26: 307-312.

26. Bernabei R, Landi F, Gambassi G, et al. (1998) Randomised trial of impact of model of integrated care and case management for older people living in the community. BMJ 316: 1348-1351.

27. Bouman A, Van Rossum E, Ambergen T, Kempen G, Knipschild P (2008) Effects of a home visiting program for older people with poor health status: A randomized, clinical trial in the Netherlands. . J Am Geriatr Soc 56: 397-404.

28. Bouman A, Van Rossum E, Evers SM, Ambergen T, Kempen G, et al. (2008) Effects on health care use and associated cost of a home visiting program for older people with poor health status: A randomized clinical trial in the Netherlands. J Gerontol A Biol Sci Med Sci 63A: 291-297.

29. Nicolaides-Bouman A, van Rossum E, Habets H, Kempen GI, Knipschild P (2007) Home visiting programme for older people with health problems: Process evaluation. J Adv Nurs 58: 425-435.

30. Nicolaides-Bouman A, van Rossum E, Kempen GI, Knipschild P (2004) Effects of home visits by home nurses to elderly people with health problems: Design of a randomised clinical trial in the Netherlands. BMC Health Serv Res 4: 35.

31. Byles JE, Tavener M, O'Connell RL, et al. (2004) Randomised controlled trial of health assessments for older Australian veterans and war widows. Med J Aust 181: 186-190.

32. Caplan GA, Williams AJ, Daly B, Abraham K (2004) A randomized, controlled trial of comprehensive geriatric assessment and multidisciplinary intervention after discharge of elderly from the emergency department--The DEED II Study. J Am Geriatr Soc 52: 1417-1423.

33. Counsell SR, Callahan CM, Buttar AB, Clark DO, Frank KI (2006) Geriatric Resources for Assessment and Care of Elders (GRACE): A new model of primary care for low-income seniors. J Am Geriatr Soc 54: 1136-1141.

34. Counsell SR, Callahan CM, Clark D, et al. (2007) Geriatric care management for low-income seniors: A randomized controlled trial. JAMA 298: 2623-2633.

35. Counsell SR, Callahan CM, Tu W, Stump TE, Arling GW (2009) Cost analysis of the geriatric resources for assessment and care of elders care management intervention. J Am Geriatr Soc 57: 1420-1426.

36. Dalby DM, Sellors JW, Fraser FD, Fraser C, van Ineveld C, et al. (2000) Effect of preventive home visits by a nurse on the outcomes of frail elderly people in the community: A randomized controlled trial. CMAJ 162: 497-500.

37. Gunner-Svensson F, Ipsen J, Olsen J, Waldstrom B (1984) Prevention of relocation of the aged in nursing homes. Scand J Prim Health Care 2: 49-56.

38. Hall N, De BP, Johnson D, Mackinnon K, Gutman G, et al. (1992) Randomized trial of a health promotion program for frail elders. Can J Aging 11: 72-91.

39. Hebert R, Robichaud L, Roy PM, Bravo G, Voyer L (2001) Efficacy of a nurse-led multidimensional preventive programme for older people at risk of functional decline. A randomized controlled trial. Age Ageing 30: 147-153.

40. Kono A, Kai I, Sakato C, Harker JO, Rubenstein LZ (2004) Effect of preventive home visits for ambulatory housebound elders in Japan: A pilot study. Aging Clin Exp Res 16: 293-299.

41. Kono A, Fujita T, Tsumura C, Kondo T, Kushiyama K, et al. (2009) Preventive home visit model targeted to specific care needs of ambulatory frail elders: Preliminary report of a randomized trial design. Aging Clin Exp Res 21: 167-173.

42. Kono A, Kanaya Y, Fujita T, et al. (2011) Effects of a preventive home visit program in ambulatory frail older people: A randomized controlled trial. J Gerontol A Biol Sci Med Sci 67A: 302-309.

43. Markle-Reid M, Weir R, Browne G, Roberts J, Gafni A, et al. (2006) Health promotion for frail older home care clients. J Adv Nurs 54: 381-395.

44. McEwan RT, Davison N, Forster DP, Pearson P, Stirling E (1990) Screening elderly people in primary care: A randomized controlled trial. Br J Gen Pract 40: 94-97.

45. Pathy MS, Bayer A, Harding K, Dibble A (1992) Randomised trial of case finding and surveillance of elderly people at home. Lancet 340: 890-893.

46. Ploeg J, Brazil K, Hutchison B, et al. (2010) Effect of preventive primary care outreach on health related quality of life among older adults at risk of functional decline: Randomised controlled trial. BMJ 340: c1480.

47. Shapiro A, Taylor K (2002) Effects of a community-based early intervention program on the subjective well-being, institutionalization, and mortality of low-income elders. Gerontologist 42: 334-341.

48. Sommers LS, Marton KI, Barbaccia JC, Randolph J (2000) Physician, nurse, and social worker collaboration in primary care for chronically ill seniors. Arch Intern Med: 1825-1833.

49. Sorensen KH, Silvertsen J (1988) Follow-up three years after intervention to relieve unmet medical and social needs of old people. Compr Gerontol B 2: 85-89.

50. Alessi CA, Stuck AE, Aronow HU, et al. (1997) The process of care in preventive in-home comprehensive geriatric assessment. J Am Geriatr Soc 45: 1044-1050.

51. Büla CJ, Alessi CA, Aronow HU, et al. (1995) Community physicians' co-operation with a program of in-home comprehensive geriatric assessment. J Am Geriatr Soc 43: 1016-1020.

52. Rubenstein LZ, Aronow HU, Schloe M, et al. (1994) A home-based geriatric assessment, follow-up and health promotion program: Design, methods, and baseline findings from a 3-year randomized clinical trial. Aging Clin Exp Res 6: 105-120.

53. Stuck AE, Aronow HU, Steiner A, et al. (1995) A trial of annual in-home comprehensive geriatric assessments for elderly people living in the community. N Engl J Med 333: 1184-1189.

54. Stuck AE, Minder CE, Peter-Wuest I, et al. (2000) A randomized trial of in-home visits for disability prevention in community-dwelling older people at low and high risk for nursing home admission. Arch Intern Med 160: 977-986.

55. Thomas R, Worrall G, Elgar F, Knight J (2007) Can they keep going on their own? A four-year randomized trial of functional assessments of community residents. Can J Aging 26: 379-389.

56. van Rossum E, Frederiks CM, Philipsen H, Portengen K, Wiskerke J, et al. (1993) Effects of preventive home visits to elderly people. BMJ 307: 27.

57. Vetter NJ, Jones DA, Victor CR (1984) Effect of health visitors working with elderly patients in general practice: A randomised controlled trial. BMJ 288: 369-372.

58. Ciaschini PM, Straus SE, Dolovich LR, et al. (2008) Management of patients at risk for falls and osteoporosis: A randomized trial. Trials 9: 62.

59. Ciaschini PM, Straus SE, Dolovich LR, et al. (2009) Community-based intervention to optimise falls risk management: A randomised controlled trial. Age Ageing 38: 724-730.

60. Close J, Ellis M, Hooper R, Glucksman E, Jackson S, et al. (1999) Prevention of falls in the elderly trial (PROFET): A randomised controlled trial. Lancet 353: 93-97.

61. Davison J, Bond J, Dawson P, Steen IN, Kenny RA (2005) Patients with recurrent falls attending Accident & Emergency benefit from multifactorial intervention - a randomised controlled trial. Age Ageing 34: 162-168.

62. Fabacher D, Josephson K, Pietruszka F, Linderborn K, Morley JE, et al. (1994) An in-home preventive assessment program for independent older adults: A randomized controlled trial. J Am Geriatr Soc 42: 630-638.

63. Gitlin LN, Hauck WW, Dennis MP, Winter L, Hodgson N, et al. (2009) Long-term effect on mortality of a home intervention that reduces functional difficulties in older adults: Results from a randomized trial. J Am Geriatr Soc 57: 476-481.

64. Gitlin LN, Hauck WW, Winter L, Dennis MP, Schulz R (2006) Effect of an in-home occupational and physical therapy intervention on reducing mortality in functionally vulnerable older people: Preliminary findings. J Am Geriatr Soc 54: 950-955.

65. Gitlin LN, Winter L, Dennis MP, Corcoran M, Schinfeld S, et al. (2006) A randomized trial of a multicomponent home intervention to reduce functional difficulties in older adults. J Am Geriatr Soc 54: 809-816.

66. Hendriks MR, Bleijlevens MH, van Haastreght JC, et al. (2008) Lack of effectiveness of a multidisciplinary fall-prevention program in elderly people at risk: A randomized, controlled trial. J Am Geriatr Soc 56: 1390-1397.

67. Huang T, Acton GJ (2004) Effectiveness of home visit falls prevention strategy for Taiwanese community-dwelling elders: Randomized trial. Public Health Nurs 21: 247-256.

68. Lightbody E, Watkins C, Leathley M, Sharma A, Lye M (2002) Evaluation of a nurse-led fallsprevention programme versus usual care: A randomized controlled trial. Age Ageing 31: 203-210.

69. Markle-Reid M, Browne G, Gafni A, et al. (2010) The effects and costs of a multifactorial and interdisciplinary team approach to falls prevention for older home care clients 'at risk' for falling: A randomized controlled trial. Can J Aging 29: 139-161.

70. Newbury J (2001) 75+ health assessments: A randomised controlled trial: Adelaide University.

71. Newbury JW, Marley JE, Beilby JJ (2001) A randomised controlled trial of the outcome of health assessment of people aged 75 years and over. Med J Aust 175: 104-107.

72. Sahlen KG, Dahlgren L, Hellner BM, Stenlund H, Lindholm L (2006) Preventive home visits postpone mortality--A controlled trial with time-limited results. BMC Public Health 6: 220.

73. Tinetti ME, Baker DI, Garrett PA, Gottschalk M, Koch ML, et al. (1993) Yale FICSIT: Risk factor abatement strategy for fall prevention. J Am Geriatr Soc 41: 315-320.

74. Tinetti ME, Baker DI, McAvay G, et al. (1994) A multifactorial intervention to reduce the risk of falling among elderly people living in the community. N Engl J Med 331: 821-827.

75. van Haastregt JC, Diederiks JP, van Rossum E, de Witte LP, Voorhoeve PM, et al. (2000) Effects of a programme of multifactorial home visits on falls and mobility impairments in elderly people at risk: Randomised controlled trial. BMJ 321: 994-998.

76. van Hout HP, Jansen AP, van Marwijk HW, Pronk M, Frijters DF, et al. (2010) Prevention of adverse health trajectories in a vulnerable elderly population through nurse home visits: A randomized controlled trial. J Gerontol A Biol Sci Med Sci 65: 734-742.

77. Vetter NJ, Lewis PA, Ford D (1992) Can health visitors prevent fractures in elderly people? BMJ 304: 888-890.

78. Yamada Y, Ikegami N (2003) Preventive home visits for community-dwelling frail elderly people based on Minimum Data Set-Home Care: Randomised controlled trial. Geriatr Gerontol Int 3: 236-242.

79. Ciechanowski P, Wagner, E., Schmaling, K., Schwartz, S., Williams, B., Diehr, P., et al. (2004) Community-integrated home-based depression treatment in older adults: A randomized controlled trial. JAMA 291: 1569-1577.

80. Crawford Shearer NB, Fleury JD, Belyea M (2010) Randomized control trial of the Health Empowerment Intervention: Feasibility and impact. Nurs Res 59: 203-211.

81. Holland R, Lenaghan E, Harvey I, et al. (2005) Does home based medication review keep older people out of hospital? The HOMER randomised controlled trial. BMJ 330: 393-395.

82. Holland R, Lenaghan E, Smith R, et al. (2006) Delivering a home- based medication review, process measures from the HOMER randomised controlled trial. Int J Pharm Pract 14: 71-79.

83. Pacini M, Smith RD, Wilson EC, Holland R (2007) Home-based medication review in older people: Is it cost effective? Pharmacoeconomics 25: 171-180.

84. Lenaghan E, Holland R, Brooks A (2007) Home-based medication review in a high risk elderly population in primary care--The POLYMED randomised controlled trial. Age Ageing 36: 292-297.

85. Luker KA (1981) Health visiting and the elderly. Nurs Times 77: 137-140.

86. Nelson ME, Layne JE, Bernstein MJ, et al. (2004) The effects of multidimensional home-based exercise on functional performance in elderly people. J Gerontol A Biol Sci Med Sci 59A: 154-160.
