# Supplementary material for: Preventive Home Visits for Mortality, Morbidity, and Institutionalization in Older Adults: A Systematic Review and Meta-Analysis
Source: PLoS One. 2014 Mar 12;9(3):e89257. doi: 10.1371/journal.pone.0089257 (PMC3951196; doi:10.1371/journal.pone.0089257)
Supplement: Table S3 — Table of excluded studies. (DOCX) [file pone.0089257.s009.docx]

# Table S3: Table of excluded studies

| **Study ID** | **Reason for exclusion** |
| --- | --- |
| Archbold 1995 [1] | Intervention for families, not for the elderly themselves. |
| Carpenter 1990 [2] | Visits not conducted by health professional. |
| Clarke 1992 [3] | Visits not conducted by health professional. |
| Clemson 2004 [4] | Intervention not a home visit: intervention conducted in community venue with follow-up home visit. |
| Comans 2010 [5] | Ineligible comparison: both intervention and control groups received home visits. |
| Dapp 2011 [6,7,8] | Only 8.8% of intervention participants opted for home visits. |
| Day 2002 [9] | Intervention not a home visit. All arms of intervention (including controls) were assessed at home by nurses. |
| de Vries 2010 [10] | Home visits not part of intervention. |
| Dunn 1994 [11] | Visit directly related to hospital discharge. |
| Engelhardt 1996 [12] | Intervention not a home visit: intervention carried out in out-patient clinic. |
| Epstein 1990 [13] | Intervention not a home visit: assessment conducted in hospital. |
| Ettinger 1997 [14,15,16,17,18] | Intervention not a home visit. |
| Fairhall 2008 [19] | Visit directly related to hospital discharge. |
| Fletcher 2004 [20] | Intervention not a home visit: less than half the assessments were conducted in participants’ homes. |
| Fordyce 1997 [21] | Intervention not a home visit: health appraisal took place during office visits. |
| Fox 2010 [22] | Only 50.0% of intervention participants opted for home visits. |
| German 1995 [23] | Intervention not a home visit: preventive visits took place at physician’s office. |
| Gill 2002 [24,25] | Ineligible comparison: both intervention and control groups received home visits. |
| Graham 2006 [26] | Home visits not carried out by health professionals. Quasi experimental design. |
| Hansen 1992 [27] | Visit directly related to hospital discharge. |
| Hay 1998 [28] | Intervention not a home visit: screening took place in primary care practice. |
| Hendriksen 1984 [29] | Not a randomised controlled trial: study refusers did not have equal chance of being assigned to either group. Informed consent followed randomisation. |
| Hornbrook 1994 [30] | Visits not conducted by health professional |
| June 2009 [31] | Not a randomised controlled trial |
| Karatay 2012 [32] | Visits not conducted by health professional – students. |
| Kerse 1999 [33] | Intervention targeted general practitioners |
| Kronborg 2006 [34] | Intervention targeted visitors |
| Legault 2011 [35] | Intervention not a home visit: centre-based intervention. |
| Leveille 1998 [36] | Intervention not a home visit: intervention carried out at senior centre. |
| Mahoney 2007 [37] | Ineligible comparison: both intervention and control groups received home visits. |
| Mann 1999 [38] | Visits directly related to rehabilitation and delivered to non-independent elderly. |
| Matzen 2007a [39] | The intervention group received an initial assessment at home with follow-up home visits, and the control group was assessed in hospital and received follow-up home visits. As both groups received home visits as part of their allocated intervention, the comparison was ineligible. |
| Matzen 2007b [40] | The intervention group received an initial assessment at home with follow-up home visits, and the control group was assessed in hospital and received follow-up home visits. As both groups received home visits as part of their allocated intervention, the comparison was ineligible. |
| McMurdo 1995 [41] | Ineligible comparison: control group received home visits. |
| Melin 1992 [42] | Visit directly related to hospital discharge. |
| Melis 2005 [43] | Some participants were living in a home for the aged. |
| Melis 2008 [44] | Some participants were living in a home for the aged. |
| Miller 1996 [45] | Visit directly related to hospital discharge. Not a randomised controlled trial. |
| Moore 1997 [46] | Intervention not a home visit. |
| Newcomer 2004 [47] | Intervention not a home visit: only 1% of visits were conducted in participant’s homes. |
| Oktay 1990 [48] | Not a randomised controlled trial: intervention and control groups recruited over different time period. |
| Parsons 2012 [49] | Ineligible comparison: control group received home visits. |
| Peeters 2007 [50] | Home visits not part of intervention. |
| Poulsen 2007 [51] | Intervention focused on providers. |
| Poulstrup 2000 [52] | Not a randomised controlled trial: quasi-experimental design. |
| Robertson 2001b [53] | Not a randomised controlled trial. |
| Robichaud 2000 [54] | Not a randomised controlled trial. |
| Rosie 2007 [55] | Intervention not a home visit. |
| Salminen 2008 [56] | Intervention not a home visit. |
| Salminen 2009a [57] | Intervention not a home visit. |
| Salminen 2009b [58] | Intervention not a home visit. |
| Schraeder 2001 [59] | Not all participants required to have home visits as part of intervention. |
| Scogin 2007 [60] | Intervention was not a multidimensional geriatric assessment nor usual health visiting practice, but an advanced, home-delivered cognitive-behavioural therapy. |
| Silverman 1995 [61] | Intervention not a home visit: geriatric assessment took place in outpatient clinic. |
| Sjosten 2007a [62] | Intervention not a home visit. |
| Sjosten 2007b [63] | Intervention not a home visit. |
| Sjosten 2008 [64] | Intervention not a home visit. |
| Spice 2009 [65] | Intervention not preventative and not a home visit. |
| Steinberg 2000 [66] | Visits not conducted by health professional - student OTs. |
| Stewart 2005 [67,68] | Ineligible comparison: both intervention and control groups received home visits. |
| Theander 2005 [69] | Not a randomised controlled trial: no control group. |
| Toseland 1996 [70] | Intervention not a home visit. |
| Townsend 1988 [71] | Visit directly related to hospital discharge. |
| Tulloch 1979 [72] | Intervention not a home visit. |
| Vaapio 2007 [73] | Intervention not a home visit. |
| Vass 2005 [74] | Intervention targeted visitors. |
| von Renteln 2003 [75] | Not a randomised controlled trial. |
| Wagner 1994 [76] | Intervention not a home visit. |
| Wallace 1998 [77] | Intervention not a home visit. |
| Wasson 1999 [78] | Intervention not a home visit. |
| Whitehead 2003 [79] | Visit directly related to hospital discharge. |
| Williams 1992 [80] | Directly related to hospital discharge. |
| Williams 2002 [81] | Participants resided in retirement communities. |
| Wolf 2001 [82] | Intervention not a home visit. |
| Yates 2001 [83] | Visit not conducted by a health professional - masters students. |
| Zimmer 1985 [84] | Intervention not a preventive home visit: visits to homebound, chronically or terminally ill patients. |

**Table S3 References**

1. Archbold PG, Stewart BJ, Miller LL, et al. (1995) The PREP system of nursing interventions: A pilot test with families caring for older members. Res Nurs Health 18: 3-16.

2. Carpenter GI, Demopoulos GR (1990) Screening the elderly in the community: Controlled trial of dependency surveillance using a questionnaire administered by volunteers. BMJ 300: 1253-1256.

3. Clarke M, Clarke SJ, Jagger C (1992) Social intervention and the elderly: A randomized controlled trial. Am J Epidemiol 136: 1517-1523.

4. Clemson L, Cumming RG, Kendig H, Swann M, Heard R, et al. (2004) The effectiveness of a community-based program for reducing the incidence of falls in the elderly: A randomized trial. J Am Geriatr Soc 52: 1487-1494.

5. Comans TA, Brauer SG, Haines TP (2010) Randomized trial of domiciliary versus center-based rehabilitation: Which is more effective in reducing falls and improving quality of life in older fallers? J Gerontol A Biol Sci Med Sci 65: 672-679.

6. Dapp U, Anders J, von Renteln-Kruse W, Meier-Baumgartner HP (2005) Active health promotion in old age: Methodology of a preventive intervention programme provided by an interdisciplinary health advisory team for independent older people. J Public Health (Oxf) 13: 122-127.

7. Dapp U, Anders JAM, von Renteln-Kruse W, et al. (2011) A randomized trial of effects of health risk appraisal combined with group sessions or home visits on preventive behaviors in older adults. J Gerontol A Biol Sci Med Sci 66A: 591-598.

8. Stuck AE, Kharicha K, Dapp U, et al. (2007) The PRO-AGE study: An international randomised controlled study of health risk appraisal for older persons based in general practice. BMC Med Res Methodol 7: 2.

9. Day L, Fildes B, Gordon I, Fitzharris M, Flamer H, et al. (2002) Randomised factorial trial of falls prevention among older people living in their own homes. BMJ 325: 1-6.

10. de Vries OJ, Peeters GM, Elders PJ, et al. (2010) Multifactorial intervention to reduce falls in older people at high risk of recurrent falls: A randomized controlled trial. Arch Intern Med 170: 1110-1117.

11. Dunn RB, Lewis PA, Vetter NJ, Guy PM, Hardman CS, et al. (1994) Health visitor intervention to reduce days of unplanned hospital re-admission in patients recently discharged from geriatric wards: The results of a randomised controlled study. Arch Gerontol Geriatr 18: 15-23.

12. Engelhardt JB, Toseland RW, O'Donnell JC, Richie JT, Jue D, et al. (1996) The effectiveness and efficiency of outpatient geriatric evaluation and management. J Am Geriatr Soc 44: 847-856.

13. Epstein AM, Hall JA, Fretwell M, et al. (1990) Consultative geriatric assessment for ambulatory patients. A randomized trial in a health maintenance organization. JAMA 263: 538-544.

14. Ettinger WH, Burns R, Messier SP, et al. (1997) A randomized trial comparing aerobic exercise and resistance exercise with a health education program in older adults with knee osteoarthritis. The Fitness Arthritis and Seniors Trial (FAST). JAMA 277: 25-31.

15. Messier SP, Royer TD, Craven TE, O'Toole ML, Burns R, et al. (2000) Long-term exercise and its effect on balance in older, osteoarthritic adults: Results from the Fitness, Arthritis, and Senior Trial (FAST). J Am Geriatr Soc 48: 131-138.

16. Penninx BW, Rejeski WJ, Pandya J, et al. (2002) Exercise and depressive symptoms: a comparison of aerobic and resistance exercise effects on emotional and physical function in older persons with high and low depressive symptomatology. J Gerontol B Psychol Sci Soc Sci 57: 124-132.

17. Rejeski WJ, Brawley LR, Ettinger W, Morgan T, Thompson C (1997) Compliance to exercise therapy in older participants with knee osteoarthritis: Implications for treating disability. Med Sci Sports Exerc 29: 977-985.

18. Sevick MA, Bradham DD, Muender M, et al. (2000) Cost-effectiveness of aerobic and resistance exercise in seniors with knee osteoarthritis. Med Sci Sports Exerc 32: 1534-1540.

19. Fairhall N, Aggar C, Kurrle SE, et al. (2008) Frailty intervention trial (FIT). BMC Geriatr 8: 27.

20. Fletcher AE, Price GM, Ng ESW, et al. (2004) Population-based multidimensional assessment of older people in UK general practice: A cluster-randomised factorial trial. Lancet 364: 1667-1677.

21. Fordyce M, Bardole D, Romer L, Soghikian K, Fireman B (1997) Senior Team Assessment and Referral Program--STAR. J Am Board Fam Pract 10: 398-406.

22. Fox PJ, Vazquez L, Tonner C, Stevens JA, Fineman N, et al. (2010) A randomized trial of a multifaceted intervention to reduce falls among community-dwelling adults. Health Educ Behav 37: 831-848.

23. German PS, Burton LC, Shapiro S, et al. (1995) Extended coverage for preventive services for the elderly: Response and results in a demonstration population. Am J Public Health 85: 379-386.

24. Gill TM, Baker DI, Gottschalk M, Peduzzi PN, Allore H, et al. (2002) A program to prevent functional decline in physically frail, elderly persons who live at home. N Engl J Med 347: 1068-1074.

25. Gill TM, Baker DI, Gottschalk M, Peduzzi PN, Allore H, et al. (2004) A prehabilitation program for the prevention of functional decline: Effect on higher-level physical function. Arch Phys Med Rehabil 85: 1043-1049.

26. Graham SA (2006) Effects of a home-based physical activity program implemented by a trained caregiver on the physical function of community-dwelling older adults: The University of Texas.

27. Hansen FR, Spedtsberg K, Schroll M (1992) Geriatric follow-up by home visits after discharge from hospital: A randomized controlled trial. Age Ageing 21: 445-450.

28. Hay WI, Van Ineveld C, Browne G, et al. (1998) Prospective care of elderly patients in family practice. Is screening effective? Can Fam Physician 44: 2677-2687.

29. Hendriksen C, Lund E, Stromgard E (1984) Consequences of assessment and intervention among elderly people: A three year randomised controlled trial. BMJ 289: 1522-1524.

30. Hornbrook MC, Stevens VJ, Wingfield DJ, Hollis JF, Greenlick MR, et al. (1994) Preventing falls among community-dwelling older persons: Results from a randomized trial. Gerontologist 34: 16-23.

31. June KJ, Lee JY, Yoon JL (2009) Effects of case management using Resident Assessment Instrument-Home Care (RAI-HC) in home health services for older people. J Korean Acad Nurs 39: 366-375.

32. Karatay G, Akkus Y (2012) Effectiveness of a multistimulant home-based program on cognitive function of older adults. West J Nurs Res 34: 883-901.

33. Kerse NM, Flicker L, Jolley D, Arroll B, Young D (1999) Improving the health behaviours of elderly people: Randomised controlled trial of a general practice education programme. BMJ 319: 683-687.

34. Kronborg C, Vass M, Lauridsen J, Avlund K (2006) Cost effectiveness of preventive home visits to the elderly: Economic evaluation alongside randomized controlled study. Eur J Health Econ 7: 238-246.

35. Legault C, Jennings JM, Katula JA, et al. (2011) Designing clinical trials for assessing the effectsof cognitive training and physical activityinterventions on cognitive outcomes: The Seniors Health and Activity Research Program Pilot (SHARP-P) Study, a randomized controlled trial. BMC Geriatr 11: 27.

36. Leveille SG, Wagner EH, Davis C, et al. (1998) Preventing disability and managing chronic illness in frail older adults: A randomized trial of a community-based partnership with primary care. J Am Geriatr Soc 46: 1191-1198.

37. Mahoney JE, Shea TA, Przybelski R, et al. (2007) Kenosha County falls prevention study: A randomized, controlled trial of an intermediate-intensity, community-based multifactorial falls intervention. J Am Geriatr Soc 55: 489-498.

38. Mann WC, Ottenbacher KJ, Fraas L, Tomita M, Granger CV (1999) Effectiveness of assistive technology and environmental interventions in maintaining independence and reducing home care costs for the frail elderly. Arch Fam Med 8: 210-217.

39. Matzen LE, Foged L, Pedersen P, Wengle K, Andersen-Ranberg K (2007) Geriatric home visits can prevent hospitalisation of subacute patients but is timeconsuming--A randomised study. [Geriatrisk teambesog kan forebygge indlaeggelse af subakut henvistepatienter, men er et tidskraevende tilbud: En randomiseret undersogelse]. Ugeskr Laeger 169: 2113-2118.

40. Matzen LE, Foged L, Pedersen P, Wengler K, Andersen-Ranberg K (2007) Primary visitation of elective referred geriatric patients--a randomised study of home visits compared to day hospital visits. [Primaer visitation af elektive geriatriske patienter. Enrandomiseret undersogelse af hjemmebesog kontra ambulantbesog]. Ugeskr Laeger 169: 2109-2113.

41. McMurdo MET, Johnstone R (1995) A randomized controlled trial of a home exercise programme for elderly people with poor mobility. Age Ageing 24: 425-428.

42. Melin AL, Bygren LO (1992) Efficacy of the rehabilitation of elderly primary health care patients after short-stay hospital treatment. Med Care 30: 1004-1015.

43. Melis RJ, van Eijken MI, Borm GF, et al. (2005) The design of the Dutch EASYcare study: A randomised controlled trial on the effectiveness of a problem-based community intervention model for frail elderly people. BMC Health Serv Res 5: 65.

44. Melis RJ, van Eijken MI, Teerenstra S, et al. (2008) A randomized study of a multidisciplinary program to intervene on geriatric syndromes in vulnerable older people who live at home (Dutch EASYcare Study). J Gerontol 63A: 283-290.

45. Miller DK, Lewis LM, Nork MJ (1996) Controlled trial of a geriatric case-finding and liaison service in an emergency department. J Am Geriatr Soc 44: 513-520.

46. Moore AA, Siu AL, Partridge JM, Hays RD, Adams J (1997) A randomized trial of office-based screening for common problems in older persons. Am J Med 102: 371-378.

47. Newcomer R, Maravilla V, Faculjak P, Graves M (2004) Outcomes of preventive case management among high-risk elderly in three medical groups. Eval Health Prof 27: 323-348.

48. Oktay JS, Volland PJ (1990) Post-hospital support program for the frail elderly and their caregivers: A quasi-experimental evaluation. Am J Public Health 80: 39-46.

49. Parsons J, Rouse P, Robinson EM, Sheridan N, Connolly MJ (2012) Goal setting as a feature of homecare services for older people: Does it make a difference? Age Ageing 41: 24-29.

50. Peeters GM, de Vries OJ, Elders PJ, Pluijm SM, Bouter LM, et al. (2007) Prevention of fall incidents in patients with a high risk of falling: Design of a randomised controlled trial with an economic evaluation of the effect of multidisciplinary transmural care. BMC Geriatr 7: 15.

51. Poulsen T, Elkjaer E, Vass M, Hendriksen C, Avlund K (2007) Promoting physical activity in older adults by education of home visitors. Eur J Ageing 4: 115-124.

52. Poulstrup A, Jeune B (2000) Prevention of fall injuries requiring hospital treatment among community-dwelling elderly. Eur J Public Health 10: 45-50.

53. Robertson MC, Gardner MM, Devlin N, McGee R, Campbell AJ (2001) Effectiveness and economic evaluation of a nurse delivered home exercise programme to prevent falls. 2: Controlled trial in multiple centres. BMJ 322: 701-704.

54. Robichaud L, Hebert, R., Roy, P., & Roy, C. (2000) A preventive program for community-dwelling elderly at risk of functional decline: A pilot study. Arch Gerontol Geriatr 30: 73-84.

55. Rosie J, Taylor D (2007) Sit-to-stand as home exercise for mobility-limited adults over 80 years of age—GrandStand System may keep you standing? Age Ageing 36: 555-562.

56. Salminen M, Vahlberg T, Sihvonen S, et al. (2008) Effects of risk-based multifactorial fall prevention program on maximal isometric muscle strength in community-dwelling aged: A randomized controlled trial. Aging Clin Exp Res 20: 487-493.

57. Salminen M, Vahlberg T, Sihvonen S, et al. (2009) Effects of risk-based multifactorial fall prevention on postural balance in the community-dwelling aged: A randomized controlled trial. Arch Gerontol Geriatr 48: 22-27.

58. Salminen MJ, Vahlberg TJ, Salonoja MT, Aarnio PTT, Kivela SL (2009) Effect of a risk-based multifactorial fall prevention program on the incidence of falls. J Am Geriatr Soc 57: 612-619.

59. Schraeder C, Shelton P, Sager M (2001) The effects of a collaborative model of primary care on the mortality and hospital use of community-dwelling older adults. J Gerontol A Biol Sci Med Sci 56A: M106-M112.

60. Scogin F, Morthland M, Kaufman A, Burgio L, Chaplin W, et al. (2007) Improving quality of life in diverse rural older adults: A randomized trial of a psychological treatment. Psychol Aging 22: 657-665.

61. Silverman M, Musa D, Martin DC, Lave JR, Adams J, et al. (1995) Evaluation of outpatient geriatric assessment: A randomized multi-site trial. J Am Geriatr Soc 43: 733-740.

62. Sjosten NM, Salonoja M, Piirtola M, et al. (2007) A multifactorial fall prevention programme in the community-dwelling aged: Predictors of adherence. Eur J Public Health 17: 464-470.

63. Sjosten NM, Salonoja M, Piirtola M, et al. (2007) A multifactorial fall prevention programme in home-dwelling elderly people: A randomized-controlled trial. Public Health 121: 308-318.

64. Sjosten NM, Vahlberg TJ, Kivela SL (2008) The effects of multifactorial fall prevention on depressive symptoms among the aged at increased risk of falling. Int J Geriatr Psychiatry 23: 504-510.

65. Spice CL, Morotti W, George S, et al. (2009) The Winchester falls project: A randomised controlled trial of secondary prevention of falls in older people. Age Ageing 38.

66. Steinberg M, Cartwright C, Peel N, Williams G (2000) A sustainable programme to prevent falls and near falls in community dwelling older people: Results of a randomised trial. J Epidemiol Community Health 54: 227-232.

67. Flood C, Mugford M, Stewart S, Harvey I, Poland F, et al. (2005) Occupational therapy compared with social work assessment for older people. An economic evaluation alongside the CAMELOT randomised controlled trial. Age Ageing 34: 47-52.

68. Stewart S, Harvey I, Poland F, Lloyd-Smith W, Mugford M, et al. (2005) Are occupational therapists more effectvie than social workers when assessing frail older people? Results of CAMELOT, a randomised controlled trial. Age Ageing 34: 41-46.

69. Theander E, Edberg AK (2005) Preventive home visits to older people in Southern Sweden. Scand J Prim Health Care 33: 392-400.

70. Toseland RW, O'Donnell JC, Engelhardt JB, Hendler J, Thomley J, et al. (1996) Outpatient geriatric evaluation and management: Results of a randomized trial. Med Care 34: 624-640.

71. Townsend J, Piper M, Frank AO, Dyer S, North WR, et al. (1988) Reduction in hospital readmission stay of elderly patients by a community based hospital discharge scheme: A randomised controlled trial. BMJ 297: 544-547.

72. Tulloch AJ, Moore V (1979) A randomized controlled trial of geriatric screening and surveillance in general practice. J R Coll Gen Pract 29: 733-742.

73. Vaapio S, Salminen M, Vahlberg T, et al. (2007) Effects of risk-based multifactorial fall prevention on health-related quality of life among the community-dwelling aged: A randomized controlled trial. Health Qual Life Outcomes 5: 20.

74. Vass M, Avlund K, Lauridsen J, Hendriksen C (2005) Feasible model for prevention of functional decline in older people: Municipality-randomized, controlled trial. J Am Geriatr Soc 53: 568.

75. von Renteln KW, Anders J, Dapp U, Meier-Baumgartner HP (2003) Preventative home visits by a specially trained nurse for 60-year olds and elderly in Hamburg. Z Gerontol Geriatr 36: 378-391.

76. Wagner EH, LaCroix AZ, Grothaus L, et al. (1994) Preventing disability and falls in older adults: A population-based randomized trial. Am J Public Health 84: 1800-1806.

77. Wallace JI, Buchner DM, Grothaus L, et al. (1998) Implementation and effectiveness of a community-based health promotion program for older adults. J Gerontol A Biol Sci Med Sci 53: M301-M306.

78. Wasson JH, Stukel TA, Weiss JE (1999) A randomized trial of the use of patient self-assessment data to improve community practices. Eff Clin Pract 2: 1-10.

79. Whitehead C, Wundke R, Crotty M, Finucane P (2003) Evidence-based clinical practice in falls prevention: A randomised controlled trial of a falls prevention service. Aust Health Rev 26: 88-97.

80. Williams EI, Greenwell J, Groom LM (1992) The care of people over 75 years old after discharge from hospital: An evaluation of timetabled visiting by Health Visitor Assistants. J Public Health Med 14: 138-144.

81. Williams K, Mustian K, Kovacs C (2002) A home-based intervention to improve balance, gait and self-confidence in older adults. Act Adapt Aging 27: 1-16.

82. Wolf B, Feys H, De Weerdt W, van der Meer J, Aufdemkampe G (2001) Effect of a physical therapeutic intervention for balance problems in the elderly: A single-blind, randomized controlled multicentre trial. Clin Rehabil 15: 624-636.

83. Yates SM, Dunnagan TA (2001) Evaluating the effectiveness of a home-based fall risk reduction program for rural community-dwelling older adults. J Gerontol A Biol Sci Med Sci 56A: M226-M230.

84. Zimmer JG, Groth-Juncker A, McKusker J (1985) A randomized controlled study of a home health care team. Am J Public Health 75: 134-141.
