# Supplementary material for: Preventive Home Visits for Mortality, Morbidity, and Institutionalization in Older Adults: A Systematic Review and Meta-Analysis
Source: PLoS One. 2014 Mar 12;9(3):e89257. doi: 10.1371/journal.pone.0089257 (PMC3951196; doi:10.1371/journal.pone.0089257)
Supplement: Text S1 — Complete search strategies. (DOCX) [file pone.0089257.s010.docx]

# Text S1: Complete search strategies

## Databases included

## Database 1: Ovid MEDLINE(R)

## Database 2: Ovid EMBASE

## Database 3: Ovid PsycINFO

## Database 4: Central (Cochrane Central Register of Trials)

## Database 5: IBSS (International Bibliography of the Social Sciences)

## Database 6: Sociological Abstracts

## Database 7: C2-SPECTR

## Database 8: OVID Cinahl

## Database 9: Ovid British Nursing Index and Archive

## Database 10: Ovid Nursing Full Text Plus

## Database 1: Ovid MEDLINE(R)

1 House Calls/

2 ((home$ or in-home$ or domiciliary) adj2 (visit$ or support$ or care$ or service$)).tw.

3 (visit$ adj2 (nurse$ or doctor$ or physician$ or volunteer$ or health)).tw.

4 (preventive adj2 (program$ or visit$)).tw.

5 (health promotion$ or health education or health screening$ or geriatric assessment$ or preventive assessment$).mp.

6 or/1-5

7 exp Aged/

8 (((elderly or aged or old$) adj2 (person$ or people$ or man or men or woman or women or patient$)) or geriatric$ or senior citizen$).tw.

9 or/7-8

10 6 and 9

11 clinical trial.pt.

12 randomized controlled trial.pt.

13 controlled clinical trial.pt.

14 randomi?ed.ab.

15 placebo.ab.

16 randomly.ab.

17 trial.ti.

18 "clinical trials".mp.

19 or/11-18

20 Animals/

21 Humans/

22 20 not (20 and 21)

23 19 not 22

24 23 and 10

## Database 2: Ovid EMBASE

1 ((home$ or in-home$ or domiciliary) adj2 (visit$ or support$ or care$ or service$)).tw.

2 (visit$ adj2 (nurse$ or doctor$ or physician$ or volunteer$ or health)).tw.

3 (preventive adj2 (program$ or visit$)).tw.

4 (health promotion$ or health education or health screening$ or geriatric assessment$ or preventive assessment$).mp.

5 exp Aged/

6 (((elderly or aged or old$) adj2 (person$ or people$ or man or men or woman or women or patient$)) or geriatric$ or senior citizen$).tw.

7 or/5-6

8 or/1-4

9 7 and 8

10 Clinical Trial/

11 Randomized Controlled Trial/

12 Randomization/

13 Double Blind Procedure/

14 Single Blind Procedure/

15 Crossover Procedure/

16 PLACEBO/

17 placebo$.tw.

18 randomi?ed controlled trial$.tw.

19 rct.tw.

20 random allocation.tw.

21 randomly allocated.tw.

22 allocated randomly.tw.

23 (allocated adj2 random).tw.

24 single blind$.tw.

25 double blind$.tw.

26 ((treble or triple) adj blind$).tw.

27 Prospective study/

28 or/10-27

29 Case study/

30 case report.tw.

31 Abstract report/

32 Letter/

33 Editorial/

34 Note/

35 Human/

36 Nonhuman/

37 ANIMAL/

38 Animal Experiment/

39 36 or 37 or 38

40 39 not (35 and 39)

41 or/29-34,40

42 28 not 41

43 42 and 9

## Database 3: Ovid PsycINFO

1 exp Home Visiting Programs/

2 ((home$ or in-home$ or domiciliary) adj2 (visit$ or support$ or care$ or service$)).tw.

3 (visit$ adj2 (nurse$ or doctor$ or physician$ or volunteer$ or health)).tw.

4 (preventive adj2 (program$ or visit$)).tw.

5 (health promotion$ or health education or health screening$ or geriatric assessment$ or preventive assessment$).mp.

6 or/1-5

7 exp Aged/

8 (((elderly or aged or old$) adj2 (person$ or people$ or man or men or woman or women or patient$)) or geriatric$ or senior citizen$).tw.

9 or/7-8

10 6 and 9

11 methodology/

12 data collection/

13 empirical methods/

14 Experimental methods/

15 Quasi experimental methods/

16 experimental design/

17 between groups design/

18 followup studies/

19 exp longitudinal studies/

20 repeated measures/

21 experimental subjects/

22 experiment controls/

23 experimental replication/

24 exp "sampling (experimental)"/

25 placebo/

26 clinical trials/

27 exp treatment outcomes/

28 treatment effectiveness evaluation/

29 empirical study.md.

30 experimental replication.md.

31 followup study.md.

32 longitudinal study.md.

33 meta analysis.md.

34 prospective study.md.

35 retrospective study.md.

36 treatment outcome clinical trial.md.

37 placebo$.tw.

38 randomi?ed controlled trial$.tw.

39 rct.tw.

40 random allocation.tw.

41 (randomly adj1 allocated).tw.

42 (allocated adj2 random).tw.

43 ((singl$ or doubl$ or treb$ or tripl$) adj (blind$3 or mask$3)).tw.

44 (clinic$ adj (trial? or stud$3)).tw.

45 or/11-44

46 comment reply.dt.

47 editorial.dt.

48 letter.dt.

49 clinical case study.md.

50 nonclinical case study.md.

51 animal.po.

52 human.po.

53 51 not (51 and 52)

54 or/46-50,53

55 45 not 54

56 10 and 55

## Database 4: Central (Cochrane Central Register of Trials)

1 MeSH descriptor House Calls explode all trees

2 (home* or in-home* or domiciliary) near (visit* or support* or care* or service*):ti,ab,kw

3 ("health promotion"* or "health education" or "health screening*" or "geriatric assessment*" or "preventive assessment*"):ti,ab,kw

4 (visit* near (nurse* or doctor* or physician* or volunteer* or health)):ti,ab,kw

5 (preventive near (program* or visit*)):ti,ab,kw

6 (#1 OR #2 OR #3 OR #4 OR #5)

7 MeSH descriptor Aged explode all trees

8 ((elderly or aged or old*) near/2 (person* or people* or man or men or woman or women or patient*)) or geriatric* or "senior citizen*":ti,ab,kw

9 (#7 OR #8)

10 (#6 AND #9)

11 from 2006 to 2008

12 (#10 AND #11)

## Database 5: IBSS (International Bibliography of the Social Sciences)

1. KW=(house call*) or TI=(house call*) or AB=(house call*)

2. KW=((home* or in-home* or domiciliary) within 2 (visit* or support* or care* or service*)) or AB=((home* or in-home* or domiciliary) within 2 (visit* or support* or care* or service*)) or TI=((home* or in-home* or domiciliary) within 2 (visit* or support* or care* or service*))

3. KW=(visit* within 2 (nurse* or doctor* or physician* or volunteer* or health)) or TI=(visit* within 2 (nurse* or doctor* or physician* or volunteer* or health)) or AB=(visit* within 2 (nurse* or doctor* or physician* or volunteer* or health))

4. (TI=preventive within 2 (program* or visit*)) or (AB=preventive within 2 (program* or visit*)) or (KW=preventive within 2 (program* or visit*))

5. (TI=health promotion* or health education or health screening* or geriatric assessment* or preventive assessment*) or (KW=health promotion* or health education or health screening* or geriatric assessment* or preventive assessment*) or (AB=health promotion* or health education or health screening* or geriatric assessment* or preventive assessment*)

6. Or/1-5

7. DE="elderly"

8. KW=(((elderly or aged or old*) within 2 (person* or people* or man or men or woman or women or patient*)) or geriatric* or senior citizen*) or TI=(((elderly or aged or old*) within 2 (person* or people* or man or men or woman or women or patient*)) or geriatric* or senior citizen*) or AB=(((elderly or aged or old*) within 2 (person* or people* or man or men or woman or women or patient*)) or geriatric* or senior citizen*)

9. 7 or 8

10. (random* or control* or blind* or double-blind* or trial* or experiment* or RCT*

## Database 6: Sociological Abstracts

1. KW=(house call*) or TI=(house call*) or AB=(house call*)

2. KW=((home* or in-home* or domiciliary) within 2 (visit* or support* or care* or service*)) or AB=((home* or in-home* or domiciliary) within 2 (visit* or support* or care* or service*)) or TI=((home* or in-home* or domiciliary) within 2 (visit* or support* or care* or service*))

3. KW=(visit* within 2 (nurse* or doctor* or physician* or volunteer* or health)) or TI=(visit* within 2 (nurse* or doctor* or physician* or volunteer* or health)) or AB=(visit* within 2 (nurse* or doctor* or physician* or volunteer* or health))

4. (TI=preventive within 2 (program* or visit*)) or (AB=preventive within 2 (program* or visit*)) or (KW=preventive within 2 (program* or visit*))

5. (TI=health promotion* or health education or health screening* or geriatric assessment* or preventive assessment*) or (KW=health promotion* or health education or health screening* or geriatric assessment* or preventive assessment*) or (AB=health promotion* or health education or health screening* or geriatric assessment* or preventive assessment*)

6. Or/1-5

7. DE="elderly"

8. KW=(((elderly or aged or old*) within 2 (person* or people* or man or men or woman or women or patient*)) or geriatric* or senior citizen*) or TI=(((elderly or aged or old*) within 2 (person* or people* or man or men or woman or women or patient*)) or geriatric* or senior citizen*) or AB=(((elderly or aged or old*) within 2 (person* or people* or man or men or woman or women or patient*)) or geriatric* or senior citizen*)

9. 7 or 8

10. (random* or control* or blind* or double-blind* or trial* or experiment* or RCT*)

## Database 7: C2-SPECTR

1. house call*

2. (home* or in-home* or domiciliary*) and (visit* or support* or care* or service*)

3. visit* and (nurse* or doctor* or physician* or volunteer$ or health)

4. preventive and (program* or visit*)

5. health promotion* or health education or health screening* or geriatric assessment* or preventive assessment*

6. or/1-5

## Database 8: OVID Cinahl

1. House Calls/

2. ((home$ or in-home$ or domiciliary) adj2 (visit$ or support$ or care$ or service$)).tw.

3. (visit$ adj2 (nurse$ or doctor$ or physician$ or volunteer$ or health)).tw.

4. (preventive adj2 (program$ or visit$)).tw.

5. (health promotion$ or health education or health screening$ or geriatric assessment$ or preventive assessment$).mp.

6. or/1-5

7. exp Aged/

8. (((elderly or aged or old$) adj2 (person$ or people$ or man or men or woman or women or patient$)) or geriatric$ or senior citizen$).tw.

9. or/7-8

10. 6 and 9

11. clinical trial.pt.

12. randomized controlled trial.pt.

13. controlled clinical trial.pt.

14. randomized.ab.

15. placebo.ab.

16. Clinical Trials/

17. randomly.ab.

18. trial.ti.

19. or/11-18

20. Animals/

21. Humans/

22. 20 not (20 and 21)

23. 19 not 22

24. 23 and 10

## Database 9: Ovid British Nursing Index and Archive

1 ((home$ or in-home$ or domiciliary) adj2 (visit$ or support$ or care$ or service$)).tw.

2 (visit$ adj2 (nurse$ or doctor$ or physician$ or volunteer$ or health)).tw.

3 (preventive adj2 (program$ or visit$)).tw.

4 (health promotion$ or health education or health screening$ or geriatric assessment$ or preventive assessment$).mp.

5 or/1-4

6 (((elderly or aged or old$) adj2 (person$ or people$ or man or men or woman or women or patient$)) or geriatric$ or senior citizen$).tw.

7 (older patients or older people).sh.

8 (elderly or elderly nursing or elderly services).sh.

9 or/6-8

10 5 and 9

## Database 10: Ovid Nursing Full Text Plus

1 exp House Calls/

2 ((home$ or in-home$ or domiciliary) adj2 (visit$ or support$ or care$ or service$)).tw.

3 (visit$ adj2 (nurse$ or doctor$ or physician$ or volunteer$ or health)).tw.

4 (preventive adj2 (program$ or visit$)).tw.

5 (health promotion$ or health education or health screening$ or geriatric assessment$ or preventive assessment$).mp.

6 or/1-5

7 exp Aged/

8 (((elderly or aged or old$) adj2 (person$ or people$ or man or men or woman or women or patient$)) or geriatric$ or senior citizen$).tw.

9 or/7-8

10 6 and 9

11 exp "clinical trial [publication type]"/

12 randomized controlled trial.pt.

13 controlled clinical trial.pt.

14 randomized.ab.

15 placebo.ab.

16 Clinical Trials/

17 randomly.ab.

18 trial.ti.

19 or/11-18

20 Animals/

21 Humans/

22 20 not (20 and 21)

23 19 not 22

24 23 and 10
